# Proteomic analysis of quail calcified eggshell matrix: a comparison to chicken and turkey eggshell proteomes

**DOI:** 10.1186/s12953-015-0078-1

**Published:** 2015-08-27

**Authors:** Karlheinz Mann, Matthias Mann

**Affiliations:** Max-Planck-Institut für Biochemie, Abteilung Proteomics und Signaltransduktion, Am Klopferspitz 18, D-82152 Martinsried, Germany

## Abstract

**Background:**

Eggshell mineralization in commercially important species such as chicken, turkey or quail is of interest as a general model of calcium carbonate biomineralization. Knowledge of proteins and molecular mechanisms in eggshell assembly may also pave the way to manipulation of thickness of the calcified layer or other features. Comparison of eggshell matrix proteomes of different species may contribute to a better understanding of the mineralization process. The recent publication of the quail genome sequence now enables the proteomic analysis of the quail shell matrix and this comparison with those of chicken and turkey.

**Results:**

The quail eggshell proteome comprised 622 identified proteins, 311 of which were shared with chicken and turkey eggshell proteomes. Forty-eight major proteins (iBAQ-derived abundance higher than 0.1 % of total identified proteome) together covered 94 % of total proteome mass. Fifteen of these are also among the most abundant proteins in chicken and turkey eggshell matrix. Only three proteins with a percentage higher than 1.0 % of the total had not previously been identified as eggshell matrix proteins. These were an uncharacterized member of the latexin family, an uncharacterized protease inhibitor containing a Kunitz domain, and gastric intrinsic factor. The most abundant proteins were ovocleidin-116, ovalbumin and ovocalyxin-36 representing approximately 31, 13 and 8 % of the total identified proteome, respectively. The major phosphoproteins were ovocleidin-116 and osteopontin. While osteopontin phosphorylation sites were predominantly conserved between chicken and quail sequences, conservation was less in ovocleidin-116.

**Conclusions:**

Ovocleidin-116 and ovocalyxin-36 are among the most abundant eggshell matrix proteins in all three species of the family Phasianidae analyzed so far, indicating that their presently unknown function is essential for eggshell mineralization. Evidence for other chicken eggshell-specific proteins in quail was inconclusive. Therefore measurement of additional eggshell proteomes, especially from species of different families and preferentially from outside the order Galliformes, will be necessary.

**Electronic supplementary material:**

The online version of this article (doi:10.1186/s12953-015-0078-1) contains supplementary material, which is available to authorized users.

## Background

The biological function of the avian eggshell is to provide protection against mechanical impact, to form a first line of defense against microbial infection, to regulate water and gas exchange and to provide calcium for the developing embryo [1]. The shell is formed in the eggshell gland (uterus) and consists to approximately 95 % of calcium carbonate in the form of calcite. The calcified layer is pervaded by an organic matrix consisting of proteins and proteoglycans, which is thought to provide a 3-dimensional network guiding and controlling the mineralization process [2]. The most studied avian eggshell is that of the chicken, probably because of commercial importance and easy accessibility. Among the first identified chicken eggshell matrix proteins were major egg white proteins, such as ovalbumin [3], lysozyme [4], and ovotransferrin [5]. These proteins were shown by immunohistochemical methods to be part of the calcified matrix rather than surface contaminants. Egg white proteins are produced and secreted predominantly in the magnum section of the oviduct [2]. Soluble remnants of egg the white assembly may migrate with the egg into the eggshell gland, where they are eventually incorporated into the mineralizing matrix. In addition, messages for lysozyme and ovotransferrin are also present at much lower concentration in white isthmus [4, 5]. These messages can even be detected in red isthmus and uterus after extensive amplification [4, 5]. At present it is unknown what percentage of these egg white proteins found in the eggshell matrix may be contributed by these tissues. Other proteins are produced by eggshell gland epithelial cells but apparently not in other sections of the oviduct and not in other selected tissues analyzed. The first of these so-called eggshell-specific proteins was ovocleidin-17 (OC17) [6], subsequently shown to belong to the C-type lectin-like family of proteins [7]. This was followed by cloning and characterization of ovocleidin-116 (OC116) [8–10], which was first identified by its N-terminal sequence in a dermatan sulfate proteoglycan preparation from chicken eggshell [11]. It was subsequently also detected in chicken bone [12, 13] and is thus not strictly eggshell-specific. Other members of this group with possible eggshell-specific distribution are ovocalyxin-32 (OCX32), a member of the latexin family of carboxypeptidase inhibitors [14], and ovocalyxin-36 (OCX36) [15], a member of the BPI/LBP/PLUNC family of anti-microbial proteins [16]. Other ovocalyxins occasionally mentioned in publications but poorly characterized are ovocalyxins-25 and −21. OCX-25 contains protease inhibitor domains and OCX-21 is apparently identical to gastrokine-2, a constituent of the gastric secretome [17]. Two other eggshell matrix proteins of widespread distribution, osteopontin and glypican-4, are induced in eggshell gland epithelia by the mechanical strain exerted upon entry of the egg into the gland [18, 19]. The exact role of these proteins remains unknown at present, but *in vitro* studies have shown that uterine fluid as well as isolated eggshell components may influence calcite crystallization *in vitro* and thus may also control eggshell mineralization *in vivo* [20–24]. Other matrix proteins may participate in the egg’s anti-microbial defense during and after egg production [25, 26].

The almost complete sequencing of the chicken genome [27] provided the possibility to identify more eggshell matrix proteins using high-throughput mass-spectrometry-based proteomics. The first compartment of the egg to be analyzed by such methods was the acid-soluble fraction of the chicken eggshell calcified layer [28, 29], leading to identification of 528 proteins in a wide abundance range. Measurement of the acid-insoluble matrix [30, 31], the cuticle [32, 33], and the soluble fraction of the eggshell membranes in conjunction with the innermost eggshell calcified layer (mammillary cones) [34] yielded several additional protein identifications. Soluble eggshell membrane proteins were also analyzed at different stages of chicken embryo development [35]. In addition, the proteome of the uterine fluid bathing the egg during shell mineralization was compared to the proteome of the calcified shell [36]. In that study a total of 577 proteins were identified in uterine fluid and 466 in eggshell, with an overlap of 244. The proteomes of uterine fluid at different stages of eggshell mineralization has also been compared [37]. In sum, these studies identified a total of 675 eggshell proteins [37] and yielded important information on the distribution of shell proteins in different compartments and on the temporal sequence of their appearance in the uterine fluid. Proteomic studies were complemented by transcriptomic studies aiming at identifying genes expressed in uterus with possible importance for eggshell production [38–42].

Compared to chicken, the eggshell proteomes of other species were less well explored. This was due to the lack of comprehensive sequence databases, still a prerequisite for high-throughput proteomics. The publication of the almost complete turkey genome sequence [43] provided the possibility to compare the eggshell proteomes of chicken and turkey [44]. The turkey eggshell matrix yielded 697 proteins. The overlap with the chicken eggshell proteome was 52 %. However, if only turkey proteins with an abundance of >0.01 % of the total were compared, the overlap increased to 95 %. This indicated that most of the major proteins were conserved between species, but that there were also a few potentially important differences.

In the present report, we use the recently published genome-derived sequence database of Japanese quail [45] to compare another eggshell proteome of the same avian family, the Phasianidae, to the preceding ones. Previously Western blotting analysis of eggshell matrices of several avian species using antibodies against known hen eggshell matrix proteins identified ovotransferrin, osteopontin and ovalbumin as components of the quail eggshell [46]. In addition, ovomucoid and lysozyme were identified by N-terminal sequence analysis of electrophoretically separated matrix proteins [47]. More recently, an unknown 32 kDa protein of the cuticle was characterized by a short N-terminal sequence [48], and the calcified matrix was shown to contain a protein with sequence similarity to ovocleidin-116 [49]. Ovocleidin-116, ovocleidin-17, ovocalyxin-32, clusterin, cystatin, lysozyme, osteopontin, ovalbumin, ovoinhibitor, ovomucoid and ovotransferrin were also identified in quail eggshell matrix in a previous proteomic study using the chicken sequences for protein identification [50]. Here we show that the number of quail eggshell matrix proteins is similar to that of chicken and turkey and explore similarities and differences among the major matrix proteins.

## Materials and methods

### Matrix and peptide preparation

Fresh quail eggs were bought at a local market. The broken shells were emptied, cleaned under a jet of water, and washed with 5 % EDTA at 6 °C for 30 min to facilitate mechanical removal of the cuticle and the membranes. The cuticles were then removed by brushing under a jet of de-ionized water, and pieces of calcified shell were stripped off the wet membranes. The dried pieces of calcified eggshell were demineralized in 50 % acetic acid (20 ml/g of shell) at 4–8 °C for 15 h with constant stirring. The turbid mixture was dialyzed (Spectra Por 6, cut off 2000; Spectrum Europe/Carl Roth GmbH, Karlsruhe, Germany) against 2 × 10 vol. of 10 % acetic acid and 2 × 10 vol. of 5 % acetic acid, and freeze dried.

SDS-PAGE was done using pre-cast 4–12 % Novex Bis-Tris gels in the MOPS buffer system using reagents and protocols supplied by the manufacturer (Invitrogen, Carlsbad, CA). The kit sample buffer was modified by adding β-mercaptoethanol to a final concentration of 2 %, and the sample was suspended in 30 μl sample buffer/100 μg of organic matrix and heated to 70 °C for 10 min. Samples were centrifuged to sediment insoluble material and gels were loaded with the dissolved proteins of 100 μg of matrix per lane and stained with colloidal Coomassie (Invitrogen) after electrophoresis. Gels were cut into 20 slices for in-gel reduction, carbamidomethylation and digestion with trypsin [51]. Peptides were cleaned with C18 Stage-Tips [52] before mass spectrometric analysis.

### LC-MS and data analysis

Peptide mixtures were analyzed by in-line nanoflow liquid chromatography using the EASY-nLC system (Proxeon Biosystems, Odense, Denmark; now part of Thermo Fisher Scientific) with 15 cm capillary columns of an internal diameter of 75 μm filled with 3 μm Reprosil-Pur C18-AQ resin (Dr. Maisch GmbH, Ammerbuch-Entringen, Germany). The gradient consisted of 5–30 % acetonitrile in 0.5 % acetic acid at a flow rate of 250 nl/min for 85 min, 30–60 % acetonitrile in 0.5 % acetic acid at a flow rate of 250 nl/min for 5 min and 60-80 % acetonitrile in 0.5 % acetic acid at a flow rate of 250 nl/min for 7 min. The eluate was electrosprayed into an LTQ Orbitrap Velos (Thermo Fisher Scientific, Bremen, Germany) through a Proxeon nanoelectrospray ion source. The Orbitrap Velos was operated in an HCD top 10 mode essentially as described [53] at a resolution of 60,000 for full scans and of 7500 for fragment measurement (both specified at m/z 400). The dynamic exclusion time was 120 s.

Raw files were processed using the Andromeda search engine-based version 1.5.1.6 of MaxQuant (http://www.maxquant.org/) [54–56] with enabled second peptide identification, iBAQ quantification and match between runs (match time window 0.5 min; alignment time window 20 min) options. We used the predicted gene database of *Coturnix japonica* [45] (http://www.nodai-genome.org/japanese_quail.html? lang = en; 30810 entries; downloaded November 2014) combined with a *Coturnix* subset of the UniProtKB database (Release 2014_9, 813 entries). In some trial searches, the sequences of chicken ovocleidins and ovocalyxins were added. The corresponding reversed databases and the sequences of common contaminants possibly introduced during sample preparation and handling were appended to the database. Carbamidomethylation was set as a fixed modification. Variable modifications were oxidation (M), N-acetyl (protein), pyro-Glu/Gln (N-term), and phosphorylation (Phospho (STY)). In some trial searches, hydroxyproline was added as a variable modification. Initial peptide mass tolerance and allowed MS/MS mass deviation were 20 ppm. Two missed cleavages were allowed and the minimal length required for a peptide identification was seven amino acids. The protein false discovery rate (FDR) and PSM (peptide spectral match) FDR were set to 0.01. The minimal peptide score for unmodified and modified peptides was set to 60. Identifications with less than three sequence-unique peptides were in each case validated using the MaxQuant Expert System software [57] considering the assignment of major peaks, occurrence of uninterrupted y- or b-ion series of at least four consecutive amino acids, preferred cleavages N-terminal to proline bonds, the possible presence of a2/b2 ion pairs and immonium ions, and mass accuracy. We only accepted protein identifications with at least two sequence-unique peptides occurring in at least two replicates with a total of three peptides. Exceptions were protein identifications sharing peptides with very similar proteins and fragments of proteins obviously belonging together. The iBAQ (intensity-based absolute quantification) [58] option of MaxQuant was used to calculate, based on the sum of peak intensities, the approximate share of each protein in the total identified proteome mass, and thus discerning major from minor proteins.

Sequence homology searches were performed with FASTA (http://www.ebi.ac.uk/Tools/sss/fasta/) [59] against current releases of Uniprot Knowledgebase (UniProtKB). We also searched the quail sequence databases for some proteins of particular interest using the Local Blast function [60] of BioEdit Sequence Alignment Editor version 7.2.5 from http://www.mbio.ncsu.edu/bioedit/bioedit.html and with the TBLASTN function of the BLAST site provided by the NODAI Genome Research Center (http://www.nodai-genome.org/blast/Japanesequail_scaf_1000/blast/). We also used Kalign (http://www.ebi.ac.uk/Tools/msa/kalign/) [59] and Clustal Omega for sequence alignments (http://www.ebi.ac.uk/Tools/msa/clustalo/) [61], InterPro (http://www.ebi.ac.uk/interpro/) [62] for domain predictions, and the Venn diagram plotter (http://omics.pnl.gov/software/VennDiagramPlotter.php) for preparing Venn diagrams. Kinase motifs apart from FAM20C consensus phosphorylation motifs were predicted using NetPhosK (http://www.cbs.dtu.dk/services/NetPhosK/) [63].

## Results and discussion

**Fig. 1:**
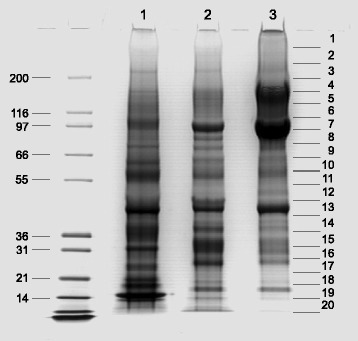
PAGE separation of eggshell matrices. Lane 1, acid-soluble chicken eggshell matrix. Lane 2, acid-soluble quail eggshell matrix. Lane 3, total quail eggshell matrix. 100 μg of matrix were separated per lane. Molecular weight of markers is shown on the left in kDa. Gel sections used for in-gel digestion and analysis are indicated on the right

In this study, we used the total PAGE sample buffer-soluble matrix of quail eggshell without separation of acid-soluble and acid-insoluble fractions because only approximately 10 % of the quail eggshell matrix is acid-soluble. Comparison of this total matrix fraction to the quail acid-soluble fraction alone showed that the band pattern remained the same but that some higher molecular weight bands were enriched in the total matrix (Fig. 1, lanes 2 and 3). In addition lane 1 of Fig. 1 shows the chicken eggshell acid-soluble matrix for comparison with the quail acid-soluble quail matrix (Fig. 1, lane 2). Both matrices showed similar complexity but low-molecular weight bands below 21 kDa were less prominent in quail than in chicken. In chicken this mobility range includes major matrix proteins, such as ovocleidin-17 and lysozyme.

To ensure we obtained a representative average shell proteome, we pooled the calcified layers of eighteen washed eggshells (12.85 g) and performed demineralization and proteome analysis in triplicate. The yield of quail eggshell matrix was 37 mg/g of air-dried eggshell calcified layer. Proteins were separated by SDS-PAGE and the gels were sliced into 20 sections (Fig. 1) for in-gel reduction, carbamidomethylation and digestion with trypsin.

**Fig. 2:**
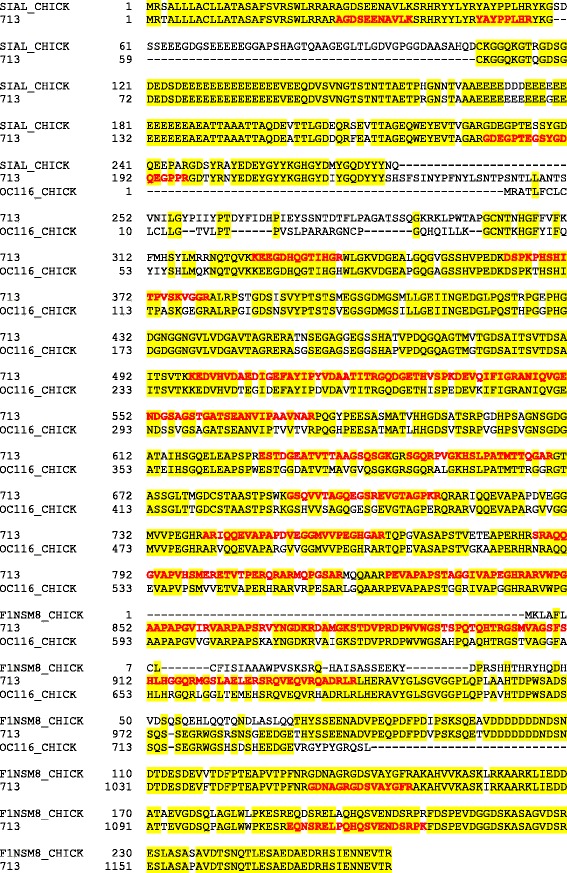
Analysis of accession 713 amino acid sequence. The sequence of quail accession no. 713 is aligned to chicken bone sialoprotein 2 (SIAL_CHICK), chicken ovocleidin-116 (OC116_CHICK) and chicken osteopontin (F1NSM8_CHICK). Identical amino acids are shaded *yellow*. Sequence regions covered by identified peptides are shown in *bold red* letters

**Fig. 3:**
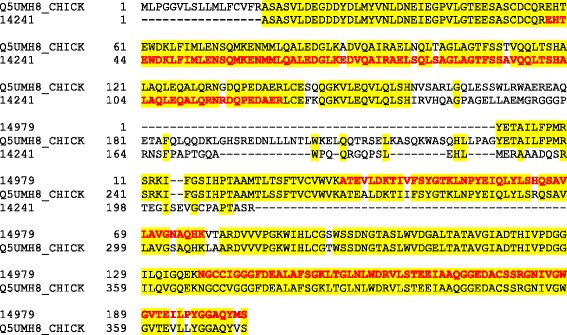
Alignment of pentraxin fragments. Quail entries 14241 and 14979 matched to different sequence regions of pentraxin (Q5UMH8_CHICK) strongly indicating that both were fragments of the same protein. Identical amino acids are shaded *yellow*. Sequence regions covered by identified peptides are shown in *bold red* letters

**Fig. 4:**
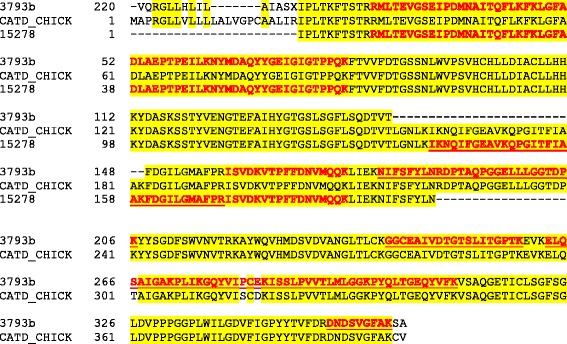
Alignment of cathepsin D fragments. Quail database entry 15278 and part of 3793 (3793b) contained overlapping sequences and were both highly similar to chicken cathepsin D. Sequences confirmed by MS/MS sequencing are shown in *red*. Peptides identified only in one of the entries and not in the other one are underlined. Peptides matching only entry 3793b align to a gap in the alignment of this entry to chicken cathepsin D, and peptides matching only entry 15278 align to gaps in 3793b. Because of the very high sequence identity of both entries to chicken cathepsin D and the occurrence of several sequenced peptides shared by both entries we suggest that both entries are incomplete and likely belong to the same protein. Identical amino acid positions are shaded *yellow*

Processing the resulting 60 raw-files with MaxQuant yielded 1173 identified protein groups (Additional file 1: Table S1). Protein groups collect protein entries that cannot be distinguished by the obtained MS evidence (however, both proteins and protein groups are used interchangeably in the manuscript). The corresponding peptide data are shown in Additional file 2: Table S2. The amino acid sequences of identified accessions were compared to sequences contained in the UniProt Knowledgebase using FASTA. The results indicated that the NODAI quail sequence database obviously contained singles entries comprising several unrelated proteins as well as different entries likely containing fragments of the same protein. The first possibility is illustrated by entry 713 (Fig. 2). The sequence of its first 230 amino acids is nearly identical to the sequence of bone sialoprotein 2. This sequence region comprised three identified sequence-unique peptides. The sequence of amino acids 231–991 was very similar to chicken ovocleidin-116 and contained 45 identified sequence-unique peptides. The remainder of the sequence of 713 was very similar to osteopontin and the four peptides identified in this part of the entry (Fig. 2) were also contained in the *Coturnix japonica* osteopontin entry Q9I832_COTJA. An example of different entries containing fragments of the same protein is illustrated using accessions 14241 and 14979, which both matched to different regions of pentraxin (Q5UMH8) without overlap in matching regions (Fig. 3). Finally, entries 15278 and part of 3793 (3793b) are an example of entries already indicating their origin from one and the same protein by partially shared sets of identified peptides (Fig. 4). Both quail database entries were highly similar to cathepsin D (CATD_CHICK).

**Fig. 5:**
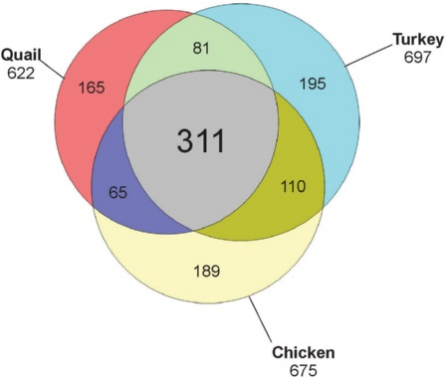
Quantitative comparison of eggshell proteomes. The number of chicken proteins derived from different reports and fractions was taken from [37]. The overlap between turkey and chicken proteomes [44] was updated to include new data [34, 36]

After elimination of identifications not matching the criteria detailed above (Materials and methods, LC-MS and data analysis) and tentatively grouping together protein fragments matching identical UniProt Knowledgebase accessions, we accepted 622 identified protein groups (Additional file 3: Table S3). However, this number remained tentative, because fragment assembly and grouping may contain errors and must be confirmed by determination of complete gene or mRNA sequences. Furthermore, proteins appear to be missing from the genome-derived database as indicated by identification of quail Uniprot protein entries not contained in the genome-derived database. The number of identified proteins is also lower than that of chicken (675; [37]) and turkey (697; [44]. Furthermore, identities between quail sequences and similar chicken or turkey sequences were frequently much lower than expected (Additional file 3: Table S3) and in almost all instances this was due to gaps in the respective sequence alignments caused by shorter quail sequences. The overlap of the quail shell proteome with turkey and chicken shell proteomes was approximately 63 and 60 %. Approximately 50 % of the proteins/protein groups were identified in all three species (Fig. 5).

As before [44, 64] we used intensity-based absolute quantification (iBAQ) as implemented in MaxQuant to discern major from minor proteins (Additional file 3: Table S3). To obtain more correct abundances for entries obviously containing sequences from different proteins, these were dissected into different database entries distinguished by small letters following the original accession number (Additional file 4: Figure S1). We also joined entries tentatively identified as fragments of the same protein to form a single accession according to the differentially shaded groups in Additional file 3: Table S3. This table contains abundances before and after database modification. Using threshold of ≥ 0.1 % of the total identified proteome to differentiate between minor and major proteins, we obtained 48 major proteins that together covered 94 % of the proteome (Table 1).

**Table 1:** Major proteins (≥0.1 %) of the quail eggshell calcified layer

Protein	Accession (possible turkey and chicken homologs in brackets)^1^	iBAQ %	iBAQ % Turkey	Chick abundance (emPAI)
Ovocleidin-116^a g^	2898a;713b (G1N6E1;OC116)	46.40	31.30	High (65.3)^2a^
Ovalbumin^i^	20482;Q6V115;20852 (G1MYL1;G1MYK6;OVAL)	15.97	12.52	High (85.6)^2a^
Ovocalyxin-36^a g, t^	21806 (G1N6M8;Q53HW8)	7.80	6.00	High (23.2)^2a^
Uncharacterized member of the latexin family	11366a	6.32	-	-
Uncharacterized proteinase inhibitor (I1)	23208	3.47	-	-
Avidin/avidin-related protein^i^	29257 (G1MSZ9;AVID)	1.95	2.67	Intermediate (2.2)^2a^
EGF-like repeat and discoidin I-like domain-containing protein 3 (EDIL3)^a i^	25038;28103;20199 (G3UT43; F1NCN3)	1.65	3.70	High (83.3)^2a^
Tsukushin^a ^ ^g, t^	7859 (G1NRF2;F1NDH7)	1.51	0.25	Intermediate (3.9)^2a^
Alpha-2-antiplasmin (SERPINF2)^a^	5112;6916 (G1N0Z7;F1NAR5)	0.60	0.80	Low-intermediate^2b,c,d^
Gastric intrinsic factor	8574 (G1MW98;R4GLQ4)	0.42	-	-
Serum albumin^g, t^	16345;19800;4290a (G1NCR2;ALBU;F2Z4L6)	0.39	2.57	High (113.5)^2a^
Lactadherin (MFGE8)^i^	9870;11245;14016 (G1N944;E1C0K5)	0.36	1.25	High (15.9)^2a^
Glutathione peroxidase (GPX3)^i, t^	19686 (G1N365;F1NPJ8)	0.36	0.04	Intermediate (2.2)^2a^
Actin(s)^i^	7084 (G1NS52;ACT5;ACTG1))	0.36	-	High (9.0)^2a^
Uncharacterized/similar to mucin(−5 AC)	4106;10751;6295;6355 (G1N8Z1;E1C037)	0.35	0.15	Low (1.4)^2a^
Uncharacterized/Ovostatin-like (OVSTL)	15509;25086;455a;9460 (G1NK51;F1NEW8)	0.34	0.05	Low (1.5)^2a^
Prostate stem cell antigen (PSCA)^a^ ^t^	20430 (G1NJY7;F1NXM7)	0.34	0.18	Low (0.8)^2a^
Extracellular fatty acid-binding protein (EXFAB)	Q9I9P7 (G1MQ16;E1C0K1)	0.34	3.82	High (26.8)^2a^
Pigment epithelium-derived factor/SERPINF1^i^	5860 (G1N131;E1C7H6)	0.31	0.64	Intermediate (6.0)^2a^
Clusterin^a i^	6174;P14018 (G1NMV6;Q9YGP0)	0.31	0.31	High (42.7)^2a^
Serpin E2/Glia-derived nexin	6327 (G3URX3;E1BWU2)	0.31	0.18	High (11.1)^2a^
Osteopontin^a^	Q9I832;713c (G1N6D8;G1N6D8)	0.28	0.15	High (1.89)^2a^
Apolipoprotein D (APOD)	28649 (G1N591;Q5G8Y9)	0.27	0.04	High (18.3)^2a^
Glypican-4^a^	6640 (G1MVZ0;F1NAU1)	0.23	0.12	Intermediate (6.3)^2a^
Semaphorin-3G^g, t^	621a (G1MXI6;F1NQ93)	0.22	0.15	Low (1.7)^2a^
Similar to Bactericidal/ permeability-increasing protein-like 2/BPI fold-containing family C protein^a i, t^	28084;20375 (IPI_CHICK:00571823)	0.21	-	High (16.8)^2a^
Pentraxin (PTX3/PPTX)	14241;14979 (G1NDX8;Q5UMH8)	0.21	0.01	Low (0.6)^2a^
Ovalbumin-related protein X^i^	30155 (G1MZH2;R9TNA6)	0.21	<0.01	Intermediate (3.8)^2a^
Ovotransferrin (TRFE)^i^	2596;18629;24968 (G1MVV5;E1BQC2;Q4ADJ7)	0.19	2.22	High (22.9)^2a^
Cathepsin D	15278;3793b (G1N8P0;CATD)	0.18	0.22	Intermediate (5.3)^2a^
Cathepsin B	8924;17826 (G1NN37;F1N9D8)	0.18	0.09	High (24.8)^2a^
Sulfhydryl oxidase 1 (QSOX1)^i^	2299;5467 (G1MV84;QSOX1;F1NYK2)	0.18	1.08	Intermediate (4.7)^2a^
Apolipoprotein A-I^a g, t^	12041;P32918 (G1MVX1;APOA1)	0.17	0.05	Intermediate (2.2)^2a^
Lysozyme C^g^	P00701 (LYSC;LYSC)	0.14	0.64	High (128.2)^2a^
Fibronectin (FN1)	141;A0A060PIY8 (G1MWJ4;F1NJT3;F1NJT4)	0.13	0.13	High (18.9)^2a^
IGFBP5	Q9DGI2 (−;F1ND88)	0.13	-	Low (0.6)^2a^
FAM20C kinase/DMP4^a^	21781;14673 (G1MSD1;E1C4X0)	0.12	0.27	Intermediate (2.3)^2a^
Proactivator polypeptide (PSAP)	2306;1756 (G1MZV1;E1BSP1)	0.12	0.06	High (9.0)^2a^
Polyubiquitin/ubiquitin^t^	11531;2058 (G1NR52;Q91021;UBB)	0.12	0.12	High (9.0)^2a^
Regenerating islet-derived protein 4 (REG4)	8715a (G1MZE6;E1BZV4)	0.11	0.38	Low (0.7)^2a^
Cystatin^t^	14227 (G1N522;CYT)	0.11	1.67	High (45.4)^2a^
Ovomucoid^i^	P01003;15709 (IOVO;IOVO)	0.11	0.05	Intermediate (2.2)^2a^
UDP-glucuronic acid decarboxylase 1	11592 (G1NPP3;E1BV28)	0.10	0.07	Intermediate (2.2)^2a^
Uncharacterized/similar to mucin	5306;6950;1527 (G1N931;E1C037)	0.10	0.15	Low (1.4)^2a^
Insulin-like growth factor-binding protein 7 (IGFBP7)	18344 (G1N8M9;F1NVP4)	0.10	0.07	Intermediate (3.6)^2a^
Ganglioside GM2 activator^a ^ ^g, t^	24235 (G1N3B0;Q5ZK02)	0.10	-	High (12.9)^2a^
Synthenin-1 (SDCBP)^t^	24420 (G1NEY6;Q5ZHM8)	0.10	-	Intermediate (2.8)^2a^
Similar to procollagen C-endopeptidase enhancer	30796 (G1N443;F1NH70)	0.10	-	-

^1^in this order; turkey entries start with G. ^2a^[28]; ^2b^[31]; ^2c^[32]; ^2d^[36]. ^a^gene expression up-regulated in chicken uterus upon mineralization [42] or upon sexual maturation of the hen [38]. Highest abundance in chicken uterus fluid during ^i^initial phase, ^g^growth phase, ^t^terminal phase [37]

### Eggshell-specific proteins

A group of major eggshell proteins generally referred to as eggshell-specific proteins was once thought to occur specifically in the uterus because they were neither found in other sections of the oviduct nor in a few selected other tissues. These are the ovocleidins and ovocalyxins and they will be discussed in this section.

The first eggshell-specific protein to be detected in the chicken eggshell as a major matrix component was ovocleidin-17 (OC17) [6]. Sequence information of this C-type lectin-like protein was not contained in the published genome sequence of chicken but was known from direct sequence analysis of the isolated protein [7]. A full length clone containing the OC17 sequence was isolated and characterized only very recently and its mRNA expression level was found to correlate negatively with eggshell strength [65]. Furthermore, the message was found to be expressed in uterus and, at a lower level, in the preceding oviduct section, the isthmus, but not in 13 other tissues tested. However, its possible function in eggshell mineralization remains ill-defined at present and may include antimicrobial activity [25] and direct interaction with calcite surfaces or carbonate ions [22, 23, 66]. We could not identify turkey OC17 in our previous study [44], however, identification of a few peptides after addition of the chicken sequence to the searched database and previous detection of a protein in turkey eggshell matrix in Western blotting analysis using anti-chicken OC17 antiserum [46] indicated its presence in that eggshell matrix, but not in the genome sequence database. Homology searching of the quail sequence database with the chicken sequence did not yield a significant match and addition of the chicken sequence to the database searched by MaxQuant did not identify any matching peptide. This contradicts the previous identification of OC17 in quail eggshell using such a trans-species approach [50], but agrees with Western blotting results [46]. Very thick eggshells, such as the shells of ostrich [67], emu and rhea [68] all have two major proteins of this family in their shell matrix. Chicken and goose with their much thinner shell both only have one of these proteins [28, 69]. The apparent absence of an OC17 homolog in the even thinner shell of quail may thus indicate a connection between number or concentration of these proteins and eggshell thickness.

Ovoclein-116 was by far the most abundant protein of the quail and turkey eggshell matrix (Table 1), and also one of the most abundant chicken eggshell matrix components [28]. Most of the quail protein sequence was contained in the middle region of accession 713 (713b; Fig. 2). However, a second entry, accession 2298, was even more similar to chicken OC-116 than accession 713. However, we did not identify any peptides shared between these sequences. Closer examination showed that two overlapping, very abundant, peptides contained in 2298 (168 times identified; Additional file 3: Table S3) were located in the first 40 amino acids, preceded by a predicted secretion signal peptide. The N-terminus of the predicted mature protein was similar, but not identical, to a short N-terminal sequence published previously [49] (accession 2298 in Additional file 4: Figure S1). The remainder of the amino acid sequence of accession 2298 contained only two identified low-abundance peptides identified just four times altogether. In summary, the evidence indicated that accession 2298 contained the N-terminus of OC-116 missing in accession 713. We consequently combined this part of the sequence (aa1-40; 2298a) into one entry termed 713b to obtain a better estimate of OC-116 abundance. OC116 was mainly localized to the palisade layer [8], in agreement with its predominant secretion during the growth phase of eggshell mineralization [37]. The protein was also identified in chicken bone [12, 13], thus establishing a link between these two different biomineralization systems in this species. OC116 was suggested to have a mammalian counterpart, matrix extracellular phosphoprotein (MEPE), belonging to the secretory calcium-binding phosphoprotein (SCPP) group of proteins [70] or small integrin-binding ligand N-linked glycoproteins (SIBLING), a group of proteins including dentin matrix protein, osteopontin, dentin sialoprotein and other proteins important for skeletal and dental mineralization and remodeling [71, 72]. In fact, chicken OC116 is N-glycosylated [10] and phosphorylated [29]. While the phosphorylation status of the turkey protein was not determined, quail OC116 was also a major phosphoprotein of the eggshell matrix (see below).

Ovocalyxin-36 (OCX36) is related to lipopolysaccharide-binding proteins (LPS), bactericidal permeability-increasing proteins (BPIP) and Plunc family proteins [15]. Immunofluorescence staining indicated a distribution of the protein throughout the eggshell and the membrane with highest intensity at the interface between membranes and mammillary cones [15]. Its expression was greatest in the uterus during eggshell mineralization and its distribution was restricted to isthmus and uterus [15]. Its similarity to the LPS/BPI/PLUNC family of proteins and its ability to bind to bacterial lipopolysaccharide suggested a function in egg antimicrobial defense [15, 16, 73, 74]. A sequence with approximately 70 % identity to chicken OCX36 was among the most abundant proteins of the quail eggshell proteome (Additional file 3: Table S3). Thus OCX36 is a major component of the eggshell proteomes of all three species analyzed (Table 1), indicating an essential role in eggshell production.

Ovocalyxin-32 (OCX32) is a member of the latexin family of carboxypeptidase inhibitors [14], with unknown function in the eggshell matrix. It was most abundant in uterine fluid during the initial phase of mineralization [37]. We found OCX32 neither in turkey eggshell matrix [44] nor in quail matrix, despite the addition of the chicken sequence to databases for possible cross-species identification with peptides of identical sequence. This is in contrast to the previously reported presence of OCX32 in quail eggshell using the chicken sequence [50]. However, we identified a highly abundant quail database entry (accession 11366; Additional file 3: Table S3) with high similarity to latexin. Closer examination of the sequence and the distribution of identified peptides indicated that the highly abundant peptides all matched to the N-terminal 71 amino acids of this entry (11366a), which was not very similar to latexin but showed a weak similarity to chicken OCX32 (C7G541_CHICK; Additional file 3: Table S3; accession 11366 in Additional file 4: Figure S1) and contained a predicted proteinase inhibitor I47 (latexin) domain. The remainder of the sequence of this entry with high similarity to latexin (11366b) was represented by only one peptide identified only once. In conclusion, evidence for the presence of OCX32 in the quail eggshell matrix was inconclusive, but we unequivocally identified a highly abundant fragment of a protein belonging to the latexin family (Table 1) that may have a function similar to that of OCX32.

**Fig. 6:**
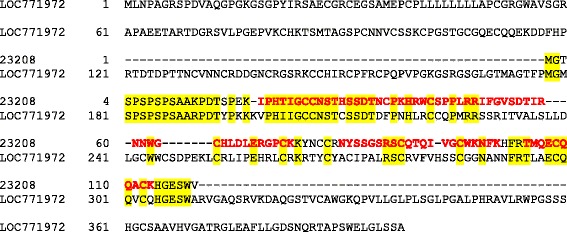
Alignment of quail accession 23208 to LOC771972 (OCX25). The isoform if LOC771972 shown is X3 (XP_004947248.1). Identical amino acids are shaded *yellow*. Sequence regions covered by identified peptides are shown in *bold red*

Two less characterized so-called eggshell-specific proteins occasionally appearing in the literature are ovocalyxin-25 (OCX25) and ovocalyxin-21 (OCX21). OCX25 was described as a protease inhibitor with WAP and Kunitz domains [37]. A Kunitz protease inhibitor-type domain was also predicted for the very abundant uncharacterized protein in quail accession 23208 (Table 1). The gene id given for chicken OCX25 [37], LOC771972, was linked to five chicken protein sequences of different length that all showed blocks of almost complete sequence identity separated by gaps in the alignments to the uncharacterized chicken protein F1NPR2. The N-terminal 50 amino acids of accession 23208 (length 120aa) also showed a weak similarity to the same chicken protein. This was also the accession retrieved by searching the quail sequence database with the sequence of LOC771972 and accession 23208 was 45 % identical to aa178-311 of LOC771972 (isoform X3, XP_004947248.1; Fig. 6). However, we are not sure whether this is sufficient to establish the protein encoded in entry 23208 as a homolog of OCX25.

OCX21 was among the most abundant proteins of the chicken eggshell matrix (IPI00574331.1) [28]. This protein is identical to gastrokine-2 (E1C2G7_CHICK) [32, 37], a protein of the gastric mucosal secretome and is therefore not truly eggshell-specific. We did not identify this protein in quail eggshell, although the quail sequence database contained two accessions (29506, 30653) with more than 90 % sequence identity to chicken and turkey OCX21/gastrokine-2 sequences. Therefore, this major chicken eggshell protein is not part of the quail eggshell proteome. Remarkably, however, all three species contain among their major shell matrix proteins a protein of the gastric secretome (see below).

### Other major proteins

Egg white proteins, such as ovalbumin, lysozyme C and ovotransferrin were among the first eggshell proteins identified [3–5] and were invariably found among the major proteins of eggshell matrices [28, 44] (Table 1). Among its major components quail eggshell matrix contained ovalbumin, avidin, ovostatin-like protein, ovalbumin-related protein X, ovotransferrin, lysozyme C, cystatin and ovomucoid. All of these were also identified in chicken and turkey eggshell matrices at different abundances (Table 1), but ovalbumin was always among the most abundant eggshell matrix components, in agreement with its very high abundance in egg white. No specific function in the mineralization process at the molecular level has been reported for these proteins so far. Some of these proteins show antimicrobial activity mediated by different mechanisms, such as degradation of bacterial cell walls, inhibition of microbial proteases or sequestration of components essential for microbial growth [75]. Therefore, these proteins are thought to contribute to general egg defense against microbial contamination, which can also happen during shell growth in the uterus. However, because several of these proteins were also shown to influence calcium carbonate mineralization *in vitro*, a direct effect on eggshell mineralization cannot be excluded at present. Many of these egg white proteins are very abundant and therefore even weak binding of calcite could affect mineralization. Egg white proteins are predominantly produced in the magnum section of the oviduct and not in uterus [38, 42] and may migrate together with the unfinished egg from the site of their synthesis to the uterus. However, the messages for lysozyme and ovotransferrin [4, 5] were also detected at much lower levels in white isthmus, red isthmus and uterus tissues. At present it is not known whether these messages are translated into protein, and, if they are, what percentage of these abundant shell matrix proteins is derived from these alternative sources.

Major proteins that we identified only in quail eggshell matrix were an uncharacterized member of the latexin family (accession 11366a) and an uncharacterized proteinase inhibitor containing a predicted Kunitz domain (accession 23208). While the first may have a similar function as OCX32, the latter may be functionally related to OCX25. Another major new eggshell matrix protein was gastric intrinsic factor (GIF). GIF is part of the gastric mucosal secretome and binds cobalamin (vitamin B12). Interestingly each of the three proteomes considered in this report contained among its most abundant proteins a protein of gastric origin that was not identified in the respective other species’ eggshell proteome. In chicken this was gastrokine-2/OCX21 [28] and in turkey this was trefoil factor 2 (TFF2) [44]. The role of these gastric proteins in eggshell production and the origin of the eggshell matrix forms remain unknown at present.

EGF-like repeats and discoidin I-like domains 3 protein (EDIL3/Del1) and tsukushin were among the most abundant proteins with an iBAQ of more than 1 % (Table 1) in all three species analyzed. EDIL3 is a widespread extracellular calcium-binding protein that was most abundant in chicken uterine fluid during the initial phase of eggshell mineralization [37]. No connection of this protein to mineralization or eggshell production is known at present. This is also true for tsukushin, a member of the small leucine-rich proteoglycan family [76]. This protein was highly abundant in quail and turkey eggshell matrix (Table 1) but comparatively less abundant in chicken eggshell matrix. In chicken uterus fluid it reached the highest abundance during growth and terminal phase [37]. The expression of both genes was up-regulated when an unfinished egg was present in the uterus [42] indicating a specific, but presently unknown, function.

The list of major proteins (Table 1) contains two proteins that were previously shown to be induced and secreted in the uterus upon entry of the egg before the advent of large scale transcriptomic studies, and may be supposed to have some specific function in shell production. These are osteopontin and glypican-4 [18, 19]. Osteopontin is a member of the SIBLING family of mineralization-related secreted phosphoproteins [71, 72]. It was identified as a major protein of the shell matrix of all three phasianid species [28, 29, 44] (Table 1). The involvement of this multifunctional protein in mineralization processes is reviewed in [72, 77, 78]. Its activity is generally inhibitory and depends on phosphorylation. The localization of osteopontin predominantly at the surface of mammillary cones and eggshell pores and at the margins of calcite columns of the palisade layer supports an inhibitory function in eggshell mineralization by binding to selected crystal surfaces [24]. Glypican-4 is one of several related cell surface heparane sulfate proteoglycans with a GPI anchor and may be released from the surface by Notum protein (present in the quail eggshell matrix as a minor protein; Additional file 3: Table S3). Glypicans have been implicated in various regulatory processes at the cell surface [79], but no connection to mineralization events has been reported so far. Glypican-4 was identified in all three species analyzed, but was a major protein only in turkey and quail eggshell matrix (Table 1).

Also among the most abundant eggshell proteins in all three species were serum albumin, lactadherin (milk fat globule membrane protein 8), extracellular fatty acid-binding protein, the extracellular chaperone clusterin, extracellular serpin E2, the extracellular matrix protein fibronectin, and ubiquitin. Nothing is known about their specific role, if any, in eggshell production. Lactadherin and clusterin were most abundant in chicken uterus fluid during the initial phase of mineralization, while ubiquitin was most abundant in the terminal phase and albumin in the growth phase [37].

### Phosphoproteins

We showed previously that higher energy collisional dissociation (HCD) fragmentation, also used in the present report, is well suited to determine peptide phosphorylation sites [80]. Applied to low-complexity proteomes such as those of biomineral matrices, this technique can yield useful information about major phosphoproteins and their phosphorylation sites without prior enrichment of modified peptides [64]. Because phosphorylation was reported previously to potentially affect biomineralization processes (reviewed in [81–83]) we included it among the variable modifications used for MaxQuant search of our raw files.

**Fig. 7:**
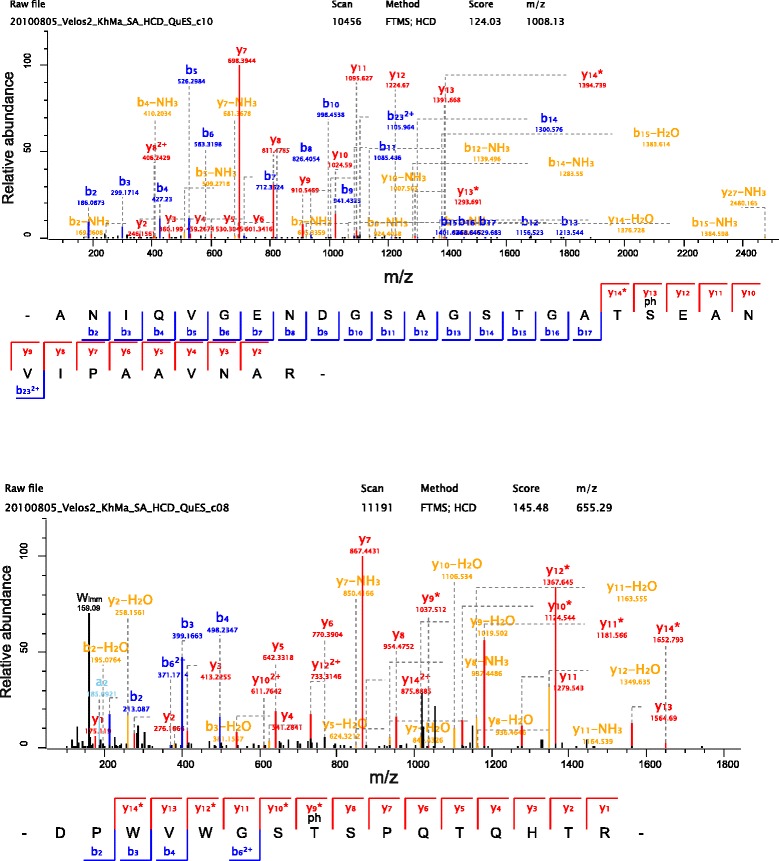
Representative spectra of ovocleidin-116 phosphopeptides. HCD spectra of two selected ovocleidin-116 phosphopeptides (compare Table 2). Y-ions are shown in *red*, b-ions in *blue*, and water or ammonia losses in *orange*. *indicates loss of H_3_PO_4_ from a phosphorylated amino acid. Such losses can occur only C-terminal to the phosphorylated amino acid. In both spectra y7 is the most intense ion, due to the frequently observed preferential cleavage N-terminal to a proline. A tryptophane immonium ion (m/z 159.0922) in the lower spectrum is labeled W_imm_

In this way, we identified 21 phosphoproteins with a total of 56 different phosphorylation sites each with a localization probability in the sequence of >0.75 [84] (Table 2; only highest probability shown). This was less than in a previous study using phosphopeptide enrichment prior to analysis of the chicken eggshell phosphoproteome, which enables the identification of even traces of phosphorylation [29]. However, such sites may be less important for protein function, at least in proteins without catalytic or regulatory function. We compared the number of repeatedly identified phosphorylated versions of peptides to the number of non-phosphorylated forms of these peptides. This indicated that most sites were phosphorylated only partially (Table 2). The highest site occupancy, close to 100 %, was found for Ser_346_ of ovalbumin. This site and a second one (S_69_), with an estimated site occupancy of approximately 25 %, were already detected previously in several avian species including chicken [85]. In addition, we detected three previously unknown phosphorylation sites at Thr_76_, Ser_77_ and Thr_266_ of ovalbumin with very low site occupancy (Table 2). Ser_80_ in cystatin [86], corresponding to Ser_103_ in accession 14227 is a previously known phosphorylation site in a quail protein. However, we could not confirm the complete site occupancy reported previously (Table 2). As in chicken [29], the major phosphoproteins of the quail eggshell were OC116 with 10 different phosphosites (Fig. 7) and site occupancies up to approximately 80 %, and osteopontin with 14 different phosphosites and occupancy of up to 75 %.

**Table 2:** Phosphoproteins and phosphosites

Accession	Peptide	Number of P-sites	Proba-bility^2^	Mod/unmod
**10704/** ***17712*** (Golgi phosphoprotein 4)^1a^	_232_IQVS **pS**HEESQAPGLLLSDLK_251_	1	0.806	3/3
	_8_ *VNAQMDDVQVH* ***pS*** *YPK*_22_	1	1	1/7
**11366** (latexin family)	_51_IMFEH**pS**REDLSYNVAQVK_68_	1	0.998	1/48
**13580** (HSPA13)^1a^	_96_NSGDA**pS**DEENKFVK_109_	1	1	2/7
**14227** (cystatin)^1a^	_97_SSADLQ**pS**CEFHDEPEMAK_114_	1	1	6/11
**18232** (PPIB)	_59_GDGTGGK**pS**IYGDRFPDENFK_78_	1	0.989	1/1^3^
**188** (VIT1; not phosvitin domain)	_1721_ESVLSD**pS**GVSEYEKDNIK_1738_	1	0.97	3/5
**2265** (β-amyloid 751)^1a^	_411_AVIQHFQEKVE**pS**LEQEAANER_431_	1	1	1/1
	_420_VE**pS**LEQEAANER_431_	1	1	2/6
**3917** (secretogranin-2)^1b^	_115_MPLGHYED**p[SS]**RDSPFK_130_	1	[0.89]	1/1
	_483_RVPVPASE**pS**DLQEDEQLEQAIR_504_	1	0.961	4/10
**5112;** ***6916*** (SERPINF2)^1a^	_24_LADTWETYGTSPSIST**pS**PETGDEESPGDK_52_	1	0.95	1/20
	_53_ATAGAV**pS**CHEQEPSGK_68_	1	1	1/17
	_284_YPLSWF**pT**LESQDIQVAK_300_	1	0.958	1/115
	_55_ *IS* ***pS*** *EEGEGEEK*_65_	1	1	1/3
	_55_ *IS* ***pS*** *EEGEGEEKNCDLTWK*_72_	1	1	7/16
	_55_ *IS* ***pS*** *EEGEGEEKNCDLTWKK*_73_	1	1	1/4
**5116** (Fibroleukin)	_110_D**p[SS]**NEFLPPNAETPAEIQDNR_131_	1	[1]	1/1
	_102_LQADDNQERD**p[SS]**NEFLPPNAETPAEIQD NR_131_	1	[1]	2/2
**5306** (uncharacterized mucin)	_170_QIECQAEDYPEI**pS**IEQVGQVVQCDVHYG LVCK_200_	1	1	21/51
**5467** (QSQX1)^1a^	_259_EHFSPGNLLLDYAIPIT **pS**GEEAAASAR_286_	1	0.931	9/23
	_311_EGETGRPG**pS** SELR_323_	1	0.968	3/4
**5586** (DNAJC3)	_212_QIE**pS**AEEFIR_221_	1	1	2/30
**621** (Semaphorin-3G)^1a^	_197_VVEAD**pS**REHTIVSR_210_	1	0.993	5/8
	_553_DILHLAS**pS**ADR_563_	1	0.976	23/53
	_553_DILHLA**p[SS]**ADRQR_565_	1	[1]	2/2
**713** (713a;bone sialoprotein 2)^1b^	_28_AGD**pS**EENAVLK_38_	1	1	3/3
**713** (713b; OC116^1a^)	_498_KEDVHVDAEDIGEFA**pY**IPYVDAATITR_524_		0.94	14/655
	_499_EDVHVDAEDIGEFA**pY**IPYVDAATITR_524_	1	0.98	48/512
	_545_ANIQVGENDGSAG**pSpT**GATSEANVIPAVNAR_575_	2	0.993; 0.829	23/1111
	_545_ANIQVGENDGSAGSTGAT **pS**EANVIPAVNAR_575_	1	0.814	240/1111
	_649_SGQRPVGKH**pS**LPATMTTQGAR_669_	1	0.984	2/13
	_657_H**pS**LPATMTTQGAR_669_	1	1	2/308
	_692_GSQVVTAGQEG**pS**REVGTAGPK_712_	1	0.916	3/3
	_787_SRAQQGVAPVH**pS**MER_801_	1	1	1/22
	_787_SRAQQGVAPVH**pS**MERETVTPER_808_	1	0.998	18/29
	_789_AQQGVAPVH**pS**MER_801_	1	1	125/251
	_789_AQQGVAPVH**pS**MERETVTPER_808_	1	0.999	202/240
	_881_ STDVPRDPWVWG**pS** TSPQTQHTR_902_	1	0.915	43/499
	_887_DPWVWG**pS** TSPQTQHTR_902_	1	0.948	15/224
	_903_GSMVAG**pS**FSHLHGGQR_918_	1	0.977	112/538
	_903_GSMVAG**pS**F**pS**HLHGGQR_918_	2	1; 1	21/538
	_919_MG**pS**LAELER_927_	1	1	46/353
	_919_MGSLAELER**pS**RQVEQVR_935_	1	1	8/8
**7172** (Nucleobindin-2)^1a^	_101_AKMD**pS**VQDTGIDHQALLK_118_	1	1	1/7
**7717** (β-amyloid 695)^1a^	_215_AVIQHFQEKVE**pS**LEQEAANER_235_	1	1	1/1
	_224_VE**pS**LEQEAANER_235_	1	1	2/5
**Q6V115** (Ovalbumin)^1a^	_57_VVHFDKLPGFGD**pS**IEAQCGTSANVHSSLR_84_	1	1	122/487
	_57_VVHFDKLPGFGD**pS**IEAQCG**pT** SANVHSSLR_84_	2	1;0.875	8/487
	_57_VVHFDKLPGFGD**pS**IEAQCGTSANVHSSLRDILNQITK_93_	1	1	3/20
	_62_LPGFGD**pS**IEAQCGTSANVHSSLR_84_	1	1	300/733
	_62_LPGFGD**pS**IEAQCGT **pS**ANVHSSLR_84_	2	1;0.976	41/733
	_265_L**pT**EWTSSIMEER277	1	1	1/614
	_341_DVVG**pS**AEAGVDATEEFR_357_	1	1	1415/1419
	_341_DVVG**pS**AEAGVDATEEFRADHPFLFCVK_367_	1	1	66/66
**Q9DGI2** (IGFBP5)^1b^	_74_EHEEPT**pT** SEMTEETYPPK_91_	1	0.809	1/6
	_74_EHEEPT**p[TS]**EMTEETYPPK_91_	1	[0.962]	2/6
	_150_HMEASLQELK**pS** SQR_163_	1	0.802	4/5
	_206_LPGTDYLSGDLQCHAFD**pS** SNVE_227_	1	0.806	3/4
**Q9I832** (Osteopontin)^1a^	_22_SKQHAISA**p[SS]**EEKYDPR_38_	1	[0.958]	1/5
	_22_SKQHAISA**pSpS**EEKYDPR_38_	2	0.913; 0.913	4/5
	_24_QHAISA**p[SS]**EEKYDPR_38_	1	[1]	21/45
	_24_QHAISAS **pS**EEKYDPR_38_	1	0.973	10/45
	_24_QHAI**pS**AS **pS**EEKYDPR_38_	2	1; 0.951	3/45
	_24_QHAISA**pSpS**EEKYDPR_38_	2	1;1	7/45
	_24_QHAISA**pSpS**EEK**pY**DPR_38_	3	0.967;0.967;0.967	1/45
	_24_QHAI**pS**A**pSpS**EEKYDPR_38_	3	1;1;1	3/45
	_133_GDNAGRGD**pS**VAYGFR_147_	1	1	5/33
	_139_GD**pS**VAYGFR_147_	1	1	1/54
	_164_KLIEDDA**pT** TEDGDSQPAGLWWPK_186_	1	0.779	1/4
	_164_KLIEDDA**p[TT** **]**EDGD**pS**QPAGLWWPK_186_	2	[1];1	1/4
	_164_KLIEDDATTEDGDSQPAGLWWPKE**pS**R_189_	1	1	5/10
	_164_KLIEDDA**pT** TEDGDSQPAGLWWPKE**pS**R_189_	2	0.912;1	1/10
	_164_KLIEDDA**p[TT]**EDGDSQPAGLWWPKE**pS**R_189_	2	[0.875];1	1/10
	_164_KLIEDDA**p[TT]**EDGDpSQPAGLWWPKE**pS**R_189_	3	[1];1;1	2/10
	_164_KLIEDDA**pT** TEDGD**pS**QPAGLWWPKE**pS**R_189_	3	0.927;1;1	1/10
	_165_LIEDDA**pT** TEDGDSQPAGLWWPK_186_	1	0.838	1/1
	_165_LIEDDA**pT** TEDGDSQPAGLWWPKE**pS**R_189_	2	0.946;1	3/4
	_165_LIEDDA**pT** TEDGD**pS**QPAGLWWPKE**pS**R_189_	3	0.819; 0.795;1	1/4
	_190_EQN**pS**RELPQHQ**pS**VEND**pS**RPK_209_	3	1;1;1	2/3
	_190_EQN**pS**RELPQHQSVEND**pS**RPK_209_	2	0.996; 0.998	1/3
	_195_ELPQHQ**pS**VEND**pS**RPK_209_	2	1;1	35/49
	_195_ELPQHQ**pS**VENDSRPK_209_	1	1	4/49
	_195_ELPQHQSVEND**pS**RPK_209_	1	1	8/49
	_210_FD**pS**REVDGGDSK_221_	1	1	1/4
	_210_FD**pS**REVDGGD**pS**K_221_	2	1;1	3/4
	_214_EVDGGD**pS**KASAGVD**pS**R_229_	2	1;0.998	3/4
	_214_EVDGGDSKA**pS**AGVD**pS**R_229_	2	0.988;1	1/4
	_230_ESQQGSVPAVDASNQ**pT**LESAEDAEDR_254_	1	0.955	1/7

^1^identified as phosphoprotein before in quail or other species; ^1a^chicken or quail, ^1b^other species. ^2^only the highest localization probability in the sequence is shown. ^3^S_66_ also occurred 9 times unmodified in the cleavage product _66_SIYGDRFPDENFK_78_. Underlined amino acids in the peptides shown represent other possible phosphorylation sites with lower probabilities or lower frequency. The number of total phosphorylated peptides always includes peptides containing sites with lower localization probabilities. Square brackets delimit neighboring amino acids if no unequivocal localization of the phosphorylation site(s) was possible

**Fig. 8:**
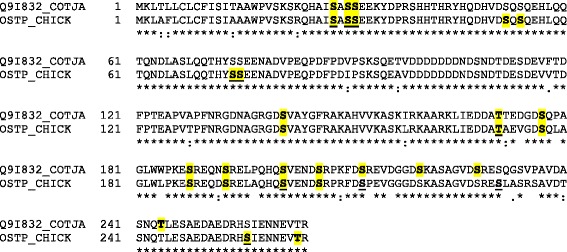
Comparison of phosphorylation sites in quail and chicken osteopontin. Phosphorylated amino acid residues of eggshell osteopontin are highlighted by *yellow* shading. Phosphorylation sites identified in metabolically ^32^P-labeled chicken osteoblast osteopontin [92] are *underlined*

While nothing is known about the specific function of OC116 phosphorylation, osteopontin is one of the few matrix proteins better characterized in this regard. This multifunctional glycosylated phosphoprotein generally inhibits mineralization in a phosphorylation-dependent way. This was shown by comparison of phosphorylated osteopontin to enzymatically de-phosphorylated or un-phosphorylated recombinant osteopontin in *in vitro* crystallization assays using different minerals, such as calcium phosphate [87], calcium oxalate [88], or calcium carbonate [89]. Calcification of cultured human smooth muscle cells, used as a model of vascular calcification, was also inhibited by phosphorylated osteopontin, but not by an enzymatically de-phosphorylated form [90]. The extent of phosphorylation may also play a role. Moderately phosphorylated osteopontin, such as bone osteopontin, inhibited mineral formation in a hydroxyapatite crystallization assay, while the more heavily phosphorylated milk osteopontin rather promoted mineralization [91]. Eleven of fourteen quail osteopontin phosphorylation sites were previously identified in chicken eggshell osteopontin by mass spectrometric analysis after phosphopeptide enrichment [29] or by Edman degradation of peptides isolated from metabolically ^32^P-labeled cultured chicken osteoblast osteopontin [92] (Fig. 8). Differences may be species specific, tissue specific, or due to differences in isolation and detection methods.

**Fig. 9:**
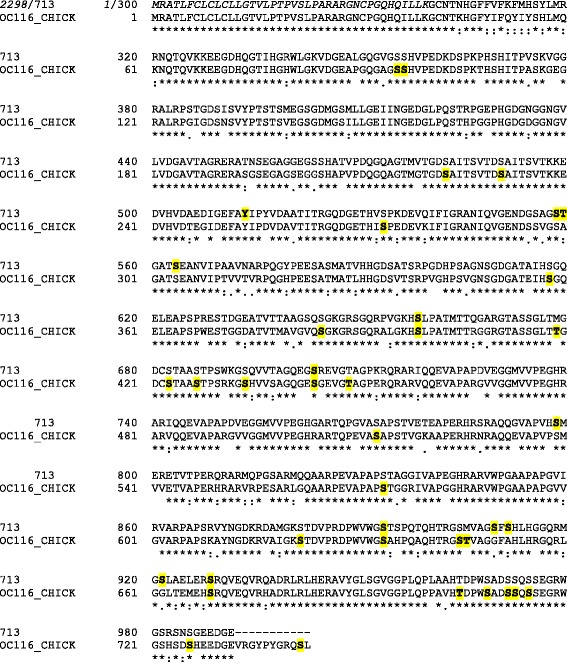
Comparison of phosphorylation sites in quail and chicken ovocleidin-116. Phosphorylation sites are highlighted by *yellow* shading. Only phosphorylation sites with a site localization probability of >0.75 [84] are shown

Comparison between quail and chicken ovocleidin-116 phosphorylation sites provided a different result (Fig. 9). Only four of the twelve quail OC116 phosphorylation sites were shared between the species, although eight of the amino acids phosphorylated in the quail protein were conserved in the chicken sequence. The higher number of phosphorylation sites in chicken OC116 may be due to phosphopeptide enrichment prior to analysis, enabling the identification of traces of phosphopeptides, possibly with no functional importance [93]. Overall, phosphorylation sites in OC116 were less well conserved than osteopontin phosphorylatin sites. It is possible that the overall extent of phosphorylation is more important in OC116 than conservation of exact positions.

A total of 12 of the phosphoproteins were previously identified as such in chicken [29], and three in mammals (Table 2). More than half of the phosphorylation sites (29 sites) agree with the consensus motif for phosphorylation by the secreted kinase FAM20C (S/T-X-[D,E,pS]), a kinase that appears to be identical with Golgi casein kinase and is known to modify many biomineralization-related extracellular proteins, such as the members of the SIBLING cluster [94, 95]. We also identified this kinase among the major quail eggshell matrix proteins (Table 1). Other kinase motifs detected in phosphopeptides were those recognized by casein kinase 2 (CH-II, four sites), protein kinase A (PKA, three sites), protein kinase C (PKC, two sites), pyruvate kinase (PK1, one site), ribosomal S6 kinase (RSK9, one site), and casein kinase 1 (CK1, one site). The phosphorylation site data, such as best score, mass error, and site probability as determined by MaxQuant are in Additional file 5: Table S4, which also contains data of peptides with lower site probability than 0.75 that were not included into Table 2.

## Conclusions

The quail eggshell matrix proteome shares 50 % of its 622 identified proteins with the shell proteomes of chicken and turkey. Nevertheless we did not find the quail homologs of several major proteins of chicken and turkey eggshell matrix, including members of the so-called eggshell-specific group of proteins. Only ovocleidin-116 and ovocalyxin-36 were unequivocally identified as major eggshell proteins in all three species analyzed and these may therefore be essential for eggshell mineralization in general (Table 1). Osteopontin, like OC116 a member of the SIBLING cluster of biomineralization-related proteins, and a major phosphoprotein in all three eggshell matrices (Table 1), may also be essential. For almost all major proteins no specific function in eggshell mineralization is known. However, there are many suggestions for possible functions based on known enzymatic or binding activities of isolated proteins, results of *in vitro* crystallization experiments, immunohistochemical localization, transcriptomic studies relating message expression level to different shell mineralization stages or eggshell properties, and most recently, proteomic analysis of the shell itself at different mineralization stages [96]. It remains to be seen how this accumulated knowledge can help to elucidate specific functions of particular proteins at the molecular level. Proteins of general importance for eggshell matrix assembly and mineralization should be conserved in other species. Therefore proteomic analysis of other eggshell matrices, especially those of species not belonging to the order Galliformes, may provide further clues concerning distribution and importance of particular matrix proteins. However, our study also emphasizes the need for better, more comprehensive, and less redundant sequence databases to facilitate such comparative studies.

## Additional files

Additional file 1: Table S1. This table is derived from the MaxQuant output table *ProteinGroups* and contains the complete list of identified proteins/protein groups including the ones that we did not accept after application of criteria described in Materials and Methods. This table also contains additional data such as the complete set of accession numbers forming one group, the distribution of peptides among the 20 PAGE sections analyzed separately, the calculated molecular weight of each entry, the iBAQ intensity and the percentages calculated from it. (XLSX 265 kb) Additional file 2: Table S2. Table S2, derived from MaxQuant output table *Peptides*, contains all identified peptide sequences belonging to the proteins of Table S1 in alphabetic order starting with the first amino acid: It also contains other relevant data such as scores, charge states, and mass accuracy. (XLSX 2472 kb) Additional file 3: Table S3. All accepted identifications with annotations derived from FASTA searches. Proteins and protein groups predicted to belong together are indicated by alternate white and grey background. The table also contains UniProt accession numbers of similar chicken and turkey proteins with FASTA-derived percentages of sequence identity and e-values, number of peptides and iBAQ-derived abundances. (DOCX 419 kb) Additional file 4: Figure S1. Alignments of quail accessions predicted to contain sequences (or partial sequences) of different proteins with the most similar chicken or turkey protein sequences. Also included is the location of identified peptides. (DOCX 143 kb) Additional file 5: Table S4. Curated version of the MaxQuant output file Phospho (STY) sites with phosphopeptide sequences, localization probabilities, scores, mass errors and other relevant data. Also included are phosphopeptides with site probabilities lower than 0.75, which were not included into Table 2. (XLS 99 kb)

## Abbreviations

Aa: Amino acid
FDR: False discovery rate
HCD: Higher-energy collision-induced dissociation
iBAQ: Intensity-based absolute quantification
OC: Ovocleidin
OCX: Ovocalyxin
PAGE: Polyacrylamide gel electrophoresis
SIBLING: Small integrin-binding ligand N-linked glycoproteins
