# Supplementary material for: Proteomic analysis of quail calcified eggshell matrix: a comparison to chicken and turkey eggshell proteomes
Source: Proteome Sci. 2015 Aug 27;13:22. doi: 10.1186/s12953-015-0078-1 (PMC4550075; doi:10.1186/s12953-015-0078-1)
Supplement: Additional file 3: Table S3. — All accepted identifications with annotations derived from FASTA searches. Proteins and protein groups predicted to belong together are indicated by alternate white and grey background. The table also contains UniProt accession numbers of similar chicken and turkey proteins with FASTA-derived percentages of sequence identity and e-values, number of peptides and iBAQ-derived abundances. (DOCX 419 kb) [file 12953_2015_78_MOESM3_ESM.docx]

**Table S3**

**Proteins of the quail calcified eggshell matrix**

|  |  |  |  |  |  |  |  |  |  |
| --- | --- | --- | --- | --- | --- | --- | --- | --- | --- |
| **NODAI**  **gene no**  **or**  **UniProtKB accession** | **Protein** | **Corresponding turkey and chicken proteins (UniProt)** | **% identity** | **e-value** | **Unique plus razor peptides** | **Repli-cate** | **Total**  **peptides** | **% of total (iBAQ)** | **% of total (iBAQ)**  **MOD** |
|  |  |  |  |  |  |  |  |  |  |
| **1**  (aa1-17627)  (aa16777-17627) | Similar to titin (Gga.55993) | R4GLP6_CHICK  (aa8961-30622)  G1NB19_MELGA  (aa14-1021) | 77.4  68.8 | 0e0  2.5e-142 | 15 | a,b,c | 18 | <0.01 | **<0.01** |
| **10000**  (aa1-286)  (aa1-286) | Serpin D1/heparin cofactor II **^1,2^** | G1MQH6_MELGA  (aa1-286)  F1NJ16_CHICK  (aa1-286) | 97.9  97.9 | 1.0e-116  5.9e-116 | 6 | a,b,c | 9 | <0.01 | **<0.01** |
| **10928**  (aa1-272)  (aa1-272)  **10010** | Teneurin-4 (TEN4) **^1,2^** | F1NVT4_CHICK  (aa1443-1679)  G1N4G9_MELGA  (aa1242-1478) | 84.6  59.9 | 2.5e-70  2.4e-51 | 1 | a | 2 | <0.01 | **<0.01** |
| **11869**  (aa1-221)  (aa1-220) | Teneurin-4 | F1NVT4_CHICK  (aa1037-1257)  G1NQR9_MELGA  (aa1079-1298) | 100.0  100.0 | 8.9e-96  9.3e-96 | 3 | a,b,c | 18 | <0.01 |  |
| **11888**  (aa1-227)  (aa1-227) | Teneurin-4  (F1NVT4_CHICK aa2501-2684) | G3USY2_MELGA  (aa49-275)  Q9DEQ8_CHICK  (aa52-278) | 99.6  99.6 | 6.1e-93  1.4e-92 | 14 | a,b,c | 48 | 0.01 |  |
| **25584**  (aa9-101)  (aa9-101) | Teneurin-4 | G1NQR9_MELGA  (aa986-1078)  F1NVT4_CHICK  (aa944-1036) | 95.7  94.6 | 7.9e-38  2.0e-37 | 3 | a,c | 5 | <0.01 |  |
| **26279**  (aa1-84) | Teneurin-4 | F1NVT4_CHICK  (aa1738-1821) | 95.2 | 5.8e-30 | 2 | a,b,c | 12 | <0.01 |  |
| **3116**  (aa1-637) | Teneurin-4 | F1NVT4_CHICK  (aa1816-2452) | 99.7 | 0e0 | 24 | a,b,c | 105 | 0.01 |  |
| **10919**  (aa1-246)  **10055**  (aa40-241) | Similar to endothelial lipase (LIPG) | F1NFW7_CHICK  (aa265-562)  G1MRF8_MELGA  (aa143-391) | 80.5  58.3 | 9.3e-60  2.8e-39 | 2 | a,b,c | 6 | <0.01 | **<0.01** |
| **10068**  (aa13-241)  (aa35-241) | Similar to beta-hexosaminidase (RCJMB04_30g17/LOC100544348) **^1,2^**; shares 6 peptides with 7139 | Q5ZI46_CHICK  (aa80-368)  G1MRV8_MELGA  (aa18-288) | 60.7  63.7 | 3.0e-55  6.5e-55 | 1 | b | 1 | <0.01 | **<0.01** |
| **7139**  (aa1-376)  (aa1-376) | Beta-hexosaminidase (HEXA/ LOC100544348); shares 6 peptides with 10068 | F1NEX5_CHICK  (aa166-526)  G1MRV8_MELGA  (aa36-446) | 87.6  87.8 | 2.2e-155  4.7e-155 | 13 | a,b,c | 38 | 0.01 |  |
| **10096**  (aa5-279)  (aa5-279) | Lysyl oxidase homolog 2 (LOXL2) **^1,2^**; shares 3 peptides with 20794 | G1MRH0_MELGA  (aa54-321)  LOXL2_CHICK  (aa54-321) | 81.0  80.7 | 1.5e-87  5.8e-87 | 8 | a,b,c | 103 | 0.04 | **0.04** |
| **12356**  (aa1-243)  (aa1-243) | Lysyl oxidase homolog 2 | LOXL2_CHICK  (aa384-626)  G1MRH0_MELGA  (aa384-626) | 99.6  98.8 | 1.2e-118  2.4e-118 | 7 | a,b,c | 60 | 0.03 |  |
| **20794**  (aa1-72)  (aa1-72) | Lysyl oxidase homolog 2; shares 3 peptides with 10096 | G1MRH0_MELGA  (aa177-248)  LOXL2_CHICK  (aa177-248) | 97.2  97.2 | 1.1e-23  1.1e-23 | 3 | a,b,c | 51 | 0.03 |  |
| **10098**  (aa1-292)  (aa1-292) | Similar to alpha-amylase (AMY1A) **^1,2^**; shares 2 peptides with 13022 | F1NW02_CHICK  (aa117-512)  G3UQ12_MELGA  (aa117-512) | 67.7  64.6 | 4.8e-62  2.3e-56 | 11 | a,b,c | 57 | 0.02 | **0.02** |
| **10440**  (aa1-283)  (aa1-283)  **10175** | Uncharacterized/Trans-aconitate 3-methyltransferase (LOC100550906/LOC424014) | G1MW72_MELGA  (aa1-274)  R4GL55_CHICK  (aa1-271) | 89.4  90.1 | 1.7e-65  6.4e-65 | 3 | a,b | 3 | <0.01 | **<0.01** |
| **10263**  (aa16-279)  (aa16-279) | Laminin subunit beta-1 (LAMB1) **^1,2^**, fragment; shares 2 peptides with 6802 | F1NJ23_CHICK  (aa1005-1269)  G1NC71_MELGA  (aa1194-1457) | 97.3  97.0 | 8.6e-85  1.7e-84 | 9 | a,b,c | 29 | <0.01 | **<0.01** |
| **10417**  (aa1-197)  (aa1-283) | Laminin subunit beta-1, fragment; shares 2 peptides with 227 | LAMB1_CHICK  (aa80-303)  G1NC71_MELGA  (aa84-399) | 83.0  77.2 | 3.0e-60  8.8e-60 | 6 | a,b,c | 28 | <0.01 |  |
| **17484**  (aa1-170)  (aa1-170) | Laminin subunit beta-1 (LAMB1) | F1NJ23_CHICK  (aa284-453)  G1NC71_MELGA  (aa471-641) | 97.1  97.1 | 3.9e-73  5.6e-72 | 2 | a,b,c | 8 | <0.01 |  |
| **22820**  (aa1-123)  (aa1-123) | Laminin subunit beta-1 (LAMB1) | F1NJ23_CHICK  (aa882-1004)  G1NC71_MELGA  (aa1071-1193) | 95.1  92.7 | 4.2e-48  2.2e-47 | 4 | a,c | 4 | <0.01 |  |
| **6802**  (aa1-390)  (aa1-390) | Laminin subunit beta-1 (LAMB1); shares 2 peptides with 10263 | F1NJ23_CHICK  (aa1147-1536)  G1NC71_MELGA  (aa1336-1728) | 97.2  96.4 | 6.0e-96  7.3e-95 | 7 | a,b,c | 15 | <0.01 |  |
| **10369**  (aa1-272)  (aa1-272) | Protein O-linked-mannose beta-1,2-N-acetylglucosaminyltransferase 1-like (POMGNT1) **^1,2^** | G1NDR4_MELGA  (aa38-288)  E1C532_CHICK  (aa38-288) | 91.5  91.5 | 1.8e-92  2.4e-92 | 5 | a,b,c | 10 | <0.01 | **<0.01** |
| **16520**  (aa1-158)  (aa1-158) | Protein O-linked-mannose beta-1,2-N-acetylglucosaminyltransferase 1 (POMGNT1); shares peptide with 21990 | G1NDR4_MELGA  (aa312-511)  E1C532_CHICK  (aa312-511) | 77.0  77.5 | 1.6e-60  1.6e-60 | 1 | a,b | 2 | <0.01 |  |
| **21990**  (aa1-130)  (aa1-130) | Protein O-linked-mannose beta-1,2-N-acetylglucosaminyltransferase 1 (POMGNT1); shares 1 peptide with 16520 | G1NDR4_MELGA  (aa471-589)  E1C532_CHICK  (aa471-589) | 78.6  78.6 | 1.8e-22  1.8e-22 | 3 | a,c | 5 | <0.01 |  |
| **10397**  (aa7-285)  (aa7-285) | Uncharacterized/similar to alpha-2-macroglobulin (A2ML3/A2M) **^1^** | E1C544_CHICK  (aa1183-1461)  G1NMH1_MELGA  (aa1191-1469) | 91.4  91.0 | 1.4e-111  2.3e-111 | 11 | a,b,c | 198 | 0.11 | **0.05** |
| **19799**  (aa1-140)  (aa1-140) | Uncharacterized/similar to alpha-2-macroglobulin (A2M/A2ML3) | G1NMH1_MELGA  (aa1060-1225)  E1C544_CHICK  (aa1052-1217) | 66.3  65.1 | 3.2e-16  2.4e-15 | 10 | a,b,c | 129 | 0.08 |  |
| **25258**  (aa1-104)  (aa1-102) | Uncharacterized/similar to alpha-2-macroglobulin (A2ML3/A2M) | E1C544_CHICK  (aa919-1022)  G1NMH1_MELGA  (aa927-1028) | 95.2  95.1 | 3.4e-41  4.5e-40 | 4 | a,b,c | 55 | 0.09 |  |
| **4167**  (aa55-516)  (aa55-516) | Uncharacterized/similar to alpha-2-macroglobulin (A2M/A2ML3) | G1NMF6_MELGA  (aa29-576)  E1C544_CHICK  (aa29-574) | 74.7  72.7 | 2.6e-107  1.4e-102 | 2 | b,c | 3 | <0.01 |  |
| **5368b**  (aa371-439)  (aa371-442) | Similar to alpha-2-macroglobulin (A2ML3)  G1NMH1_MELGA: 88.7; 1.0e-19; aa22-83 | E1C544_CHICK  (aa16-90)  G1NMF6_MELGA  (aa21-92) | 73.3  73.7 | 4.9e-18  5.1e-20 | 1 | a,b,c | 20 | ? |  |
| **7052**  (aa1-318)  (aa1-318) | Similar to alpha-2-macroglobulin (A2M)  E1C544_CHICK: 78.8%; 2.4e-33; aa616-922 | G1NMH1_MELGA  (aa617-930)  F1NF29_CHICK  (aa617-912) | 60.7  67.3 | 3.3e-75  1.9e-60 | 6 | a,b,c | 66 | 0.02 |  |
| **9582**  (aa1-300)  (aa1-304) | Similar to alpha-2-macroglobulin (A2M) | F1NF29_CHICK  (aa317-674)  G1NMH1_MELGA  (aa315-679) | 82.4  75.1 | 4.5e-100  1.9e-95 | 9 | a,b,c | 68 | 0.03 |  |
| **10413**  (aa59-284) | Calcyphosin-like protein (CAPSL) | E1BV18_CHICK  (aa3-232) | 91.7 | 9.6e-79 | 2 | a,c | 4 | <0.01 | **<0.01** |
| **10423**  (aa21-284)  (aa21-283) | Tiarin-like/Olfactomedin-4 (OLFM4) **^1,2^** | G1NQ14_MELGA  (aa226-489)  F1NXH9_CHICK  (aa206-468) | 94.3  93.2 | 1.7e-115  5.0e-114 | 10 | a,b,c | 46 | 0.02 | **0.02** |
| **16761**  (aa1-167)  (aa1-167) | Similar to tiarin-like/Olfactomedin-4 | F1NXH9_CHICK  (aa84-246)  G1NQ14_MELGA  (aa104-266) | 76.6  76.0 | 2.9e-42  1.1e-41 | 5 | a,b,c | 21 | 0.02 |  |
| **10477**  (aa1-283)  (aa2-283) | Carbohydrate sulfotransferase 11 (CHST11/LOC100857771) **^2^** | G1NJV5_MELGA  (aa29-311)  R4GMJ5_CHICK  (aa68-349) | 99.3  99.6 | 2.4e-125  3.2e-125 | 4 | a,c | 5 | <0.01 | **<0.01** |
| **10478**  (aa46-283)  (aa46-283) | Erythrocyte band 7 integral membrane protein/stomatin (STOM) **^1,2^** | G1N774_MELGA  (aa51-287)  E1BTV1_CHICK  (aa48-284) | 95.4  93.3 | 7.1e-86  2.5e-85 | 8 | a,b,c | 34 | <0.01 | **<0.01** |
| **1055**  (aa4-997)  (aa4-997) | Uncharacterized/Similar to mucin(-5B; LOC395381)) **^1,2^**; shares 12 peptides with 5308 | F1NZY2_CHICK  (aa29-1831)  G1N988_MELGA  (aa29-1829) | 50.9  50.2 | 2.0e-80  1.3e-79 | 32 | a,b,c | 403 | 0.04 | **0.03** |
| **5308**  (aa11-461)  (aa11-461) | Uncharacterized/Similar to mucin(-5B); shares 12 peptides with 1055 | F1NZY2_CHICK  (aa636-1428)  G1N988_MELGA  (aa635-1426) | 52.7  52.3 | 3.2e-78  1.4e-77 | 4 | a,b,c | 29 | 0.01 |  |
| **1056**  (aa1-1026)  (aa1-1028) | Unconventional myosin-Ic (MYO1C) **^1,2^** | MYO1C_CHICK  (aa1-1026)  G1N0G6_MELGA  (aa1-1028) | 100.0  96.1 | 0e0  0e0 | 5 | a,b,c | 8 | <0.01 | **<0.01** |
| **10652**  (aa123-272)  (aa123-272) | Torsin-1B (TOR1B) **^1,2^**; shares 1 peptide with 16016 | F1NWW1_CHICK  (aa58-207)  G1N0L0_MELGA  (aa64-213) | 96.0  96.0 | 4.4e-57  4.5e-57 | 8 | a,b,c | 21 | 0.01 | **0.02** |
| **16016**  (aa1-189)  (aa1-184) | Torsin-1B; shares 1 peptide with 10652 | F1NWW1_CHICK  (aa150-338)  G1N0L0_MELGA  (aa156-339) | 95.8  96.7 | 3.2e-81  8.0e-80 | 7 | a,b,c | 35 | 0.03 |  |
| **10661**  (aa37-277)  (aa37-277)  **7566** | Protein kinase C and casein kinase substrate in neurons protein 2 (PACSIN2) **^4^** | F1NNZ9_CHICK  (aa21-261)  G3UQ98_MELGA  (aa21-261) | 100.0  100.0 | 1.4e-80  1.4e-80 | 5 | a,b,c | 11 | <0.01 | **<0.01** |
| **10673**  (aa1-277)  (aa1-277) | Similar to beta-site APP cleaving enzyme 2 (BACE2) | Q5QHS0_CHICK  (aa9-392)  G1NP01_MELGA  (aa33-417) | 71.4  71.7 | 3.0e-53  6.4e-53 | 4 | a,b,c | 9 | <0.01 | **<0.01** |
| **10677**  **(**aa50-215)  (aa50-215) | ADP-ribosylation factor-like protein 3 (ARL3) **^3^** | G1ND25_MELGA  (aa1-166)  F1N9A3_CHICK  (aa2-167) | 100.0  100.0 | 3.5e-51  3.7e-51 | 8 | a,b,c | 21 | <0.01 | **<0.01** |
| **10689**  (aa1-277)  (aa1-277) | Phospholipase B-like 1 (PLBD1) **^1,2^** | G1NHA1_MELGA  (aa252-517)  E1BZF7_CHICK  (aa279-544) | 94.2  94.6 | 2.4e-91  3.0e-91 | 10 | a,b,c | 45 | 0.01 | **0.02** |
| **1484**  (aa115-901)  (aa61-901)  **1069** | Sodium/potassium-transporting ATPase subunit alpha-1 (ATP1A1) **^1,2,4^** | G3URR1_MELGA  (aa138-994)  F1NSY1_CHICK  (aa1-1017) | 87.3  79.2 | 3.3e-148  3.4e-148 | 3 | b,c | 4 | <0.01 | **<0.01** |
| **10691**  (aa28-219)  (aa28-219) | Retinol-binding protein 4 (RBP4) **^1,2^** | G3URC8_MELGA  (aa1-192)  RET4_CHICK  (aa1-192) | 98.4  99.0 | 7.0e-85  9.8e-85 | 7 | a,c | 16 | <0.01 | **<0.01** |
| **10704**  (aa1-276)  (aa1-276) | Similar to Golgi phosphoprotein 4 (LOC419409) **^1,2^** | E1BU50_CHICK  (aa123-398)  G1MUR4_MELGA  (aa123-305) | 96.4  50.3 | 1.0e-86  1.1e-17 | 7 | a,b,c | 38 | 0.02 | **0.02** |
| **17712**  (aa1-170)  (aa20-124) | Similar to Golgi phosphoprotein 4 | E1BU50_CHICK  (aa400-569)  G1MUR4_MELGA  (aa337-441) | 97.1  95.2 | 3.9e-53  2.7e-28 | 4 | a,b,c | 19 | 0.01 |  |
| **10706**  (aa21-276)  (aa21-156) | Similar to polypeptide N-acetylgalactosaminyl-transferase 6 (GALNT6) **^1,2^** | Q5F4C7_CHICK  (aa164-499)  G1NE26_MELGA  (aa164-349) | 75.9  72.6 | 4.6e-50  1.2e-30 | 4 | a,b,c | 7 | <0.01 | **<0.01** |
| **18456**  (aa1-163)  (aa1-163) | Polypeptide N-acetylgalactosaminyl-transferase 6 (GALNT6); shares 1 peptide with 28817 (GALNT3) | G1NE26_MELGA  (aa1-163)  Q5F4C7_CHICK  (aa1-163) | 96.9  96.9 | 2.1e-59  3.8e-59 | 1 | a | 1 | <0.01 |  |
| **10715**  (aa4-276)  (aa11-276) | von Willebrand factor A domain-containing protein 2 (VWA2) | F1NUE2_CHICK  (aa328-596)  G1NF03_MELGA  (aa331-596) | 93.0  92.5 | 2.8e-105  8.0e-102 | 6 | a,b,c | 14 | <0.01 | **<0.01** |
| **10751**  (aa1-274)  (aa1-274) | Uncharacterized/Similar to mucin (Mucin-5AC?) **^1,2^**; shares 2 peptides with 4106 | G1N8Z1_MELGA  (aa409-728)  E1C037_CHICK  (aa393-712) | 82.5  82.8 | 2.5e-93  5.6e-93 | 10 | a,b,c | 240 | 0.38 | **0.35** |
| **4106**  (aa79-530)  (aa89-530) | Uncharacterized/similar to mucin (Mucin-5AC?); shares peptides with 6295 and 10751 | G1N8Z1_MELGA  (aa7-422)  E1C037_CHICK  (aa1-406) | 64.0  65.4 | 6.0e-116  1.2e-114 | 20 | a,b,c | 588 | 0.45 |  |
| **6295**  (aa1-412)  (aa1-411) | Uncharacterized/Similar to mucin (Mucin-5AC?); shares peptides with 4106 | E1C037_CHICK  (aa731-1180)  G1N8Z1_MELGA  (aa747-1195) | 89.6  88.4 | 1.9e-115  2.2e-115 | 8 | a,b,c | 114 | 0.15 |  |
| **6355**  (aa1-328)  (aa1-252) | Uncharacterized/Similar to mucin (Mucin-5AC?) | E1C037_CHICK  (aa1264-1591)  G1N8Z1_MELGA  (aa1320-1567) | 89.9  88.9 | 1.4e-113  4.7e-41 | 20 | a,b,c | 216 | 0.31 |  |
| **10776**  (aa1-275)  (aa1-275) | Arrestin domain-containing protein 1 (ARRDC1) **^1,2^** | Q5ZKV5_CHICK  (aa78-421)  G1MTX5_MELGA  (aa39-382) | 79.1  79.4 | 2.5e-74  4.8e-75 | 5 | a,b,c | 83 | 0.02 | **0.03** |
| **108**  (aa25-1895)  (aa25-2213) | Similar to desmoplakin (DSP) **^1,2^**; shares 10 peptides with 234 | G3UTZ5_MELGA  (aa20-2064)  E1BWI0_CHICK  (aa78-2772) | 70.2  76.4 | 7.2e-149  2.6e-148 | 13 | a,b,c | 35 | <0.01 | **<0.01** |
| **234**  (aa1-1763)  (aa1-1763) | Desmoplakin; shares 10 peptides with 108 | G1MXH6_MELGA  (aa871-2697)  E1BWI0_CHICK  (aa794-2620) | 94.9  94.8 | 0e0  0e0 | 1 | a,b,c | 4 | <0.01 |  |
| **11547**  (aa13-245)  (aa86-245) | Desmoplakin | G1MXH6_MELGA  (aa1-237)  E1BWI0_CHICK  (aa1-160) | 80.2  99.4 | 2.2e-76  3.8e-66 | 2 | a,b,c | 24 | <0.01 |  |
| **10820**  (aa1-274)  (aa1-274) | Rab-GDP dissociation inhibitor (GDI2) **^1,2^**; shares 6 peptides with 13389 | G1MWF4_MELGA  (aa131-448)  O93382_CHICK  (aa131-448) | 85.8  85.8 | 1.9e-85  1.9e-85 | 4 | a,b,c | 8 | <0.01 | **<0.01** |
| **13389**  (aa1-225)  (aa1-225) | Similar to Rab-GDP dissociation inhibitor (GDI2); shares 6 peptides with 10820 | F1NCZ2_CHICK  (aa66-379)  G1MWF4_MELGA  (aa66-379) | 71.7  71.7 | 1.7e-54  1.7e-54 | 11 | a,b,c | 25 | <0.01 |  |
| **10868**  (aa19-259)  (aa106-259) | Isochorismatase domain-containing protein 1 (ISOC1) **^2^** | F1NS88_CHICK  (aa26-271)  G1MY70_MELGA  (aa1-156) | 82.9  85.3 | 3.2e-80  4.2e-48 | 3 | a,b,c | 5 | <0.01 | **<0.01** |
| **10869**  (aa1-270)  (aa1-270) | Similar to alpha-mannosidase (MAN2A2) **^1,2^**; shares 1 peptide with 6255 | F1NXU9_CHICK  (aa544-860)  G1NE21_MELGA  (aa506-823) | 64.8  64.3 | 3.5e-55  6.3e-55 | 1 | a | 1 | <0.01 | **<0.01** |
| **6255**  (aa1-409)  (aa1-409) | Alpha-mannosidase (MAN2A2); shares 1 peptide with 10869 | G1NE21_MELGA  (aa91-584)  F1NXU9_CHICK  (aa129-622) | 81.0  80.6 | 8.9e-124  3.5e-123 | 3 | a,c | 8 | <0.01 |  |
| **10880**  (aa8-273)  (aa10-273)  **8489** | Angiotensinogen (AGT) **^1,2^** | G1NHN9_MELGA  (aa1-266)  F1NDH2_CHICK  (aa1-264) | 91.7  92.4 | 9.7e-104  1.6e-101 | 5 | a,b,c | 18 | <0.01 | **0.01** |
| **10961**  (aa27-271)  (aa12-271) | Follistatin-related protein 1 (FSTL1) **^3,4^**; shares 1 peptide with 30760 | F1NM70_CHICK  (aa99-316)  G1NN97_MELGA  (aa86-307) | 85.7  66.2 | 1.7e-66  1.7e-44 | 5 | a,b,c | 20 | 0.02 | **0.03** |
| **30760**  (aa1-44) | Follistatin-related protein 1 (FSTL1); shares 1 peptide with 10961 | F1NM70_CHICK  (aa137-180) | 100.0 | 6.8e-13 | 1 | a,b,c | 7 | 0.01 |  |
| **15819**  (aa5-191)  (aa5-191)  **10983** | Similar to increased sodium tolerance 1 homolog (IST1) | E1C062_CHICK  (aa1-150)  G1N9K7_MELGA  (aa1-150) | 78.6  76.5 | 2.0e-31  1.1e-29 | 3 | a,b,c | 6 | <0.01 | **<0.01** |
| **1099**  (aa181-848)  (aa257-1013) | Similar to C3 and PZP-like alpha-2-macroglobulin domain-containing protein 8 (CPAMD8) **^1,2^**; shares 10 peptides with 5166 and 4 with 2615 | G1N1H6_MELGA  (aa27-1280)  F1NN85_CHICK  (aa135-1697) | 51.8  45.9 | 1.5e-63  3.7e-63 | 17 | a,b,c | 50 | <0.01 | **<0.01** |
| **2615**  (aa1-690)  (aa1-609) | Similar to C3 and PZP-like alpha-2-macroglobulin domain-containing protein 8 (CPAMD8); shares 4 peptides with 1099 | F1NN85_CHICK  (aa734-1397)  G1N1H6_MELGA  (aa887-1698) | 70.3 | 6.3e-89 | 10 | a,b,c | 50 | <0.01 |  |
| **5166**  (aa1-470)  (aa97-470) | Similar to C3 and PZP-like alpha-2-macroglobulin domain-containing protein 8 (CPAMD8); shares 10 peptides with 1099 | G1N1H6_MELGA  (aa29-634)  F1NN85_CHICK  (aa64-482) | 66.5  72.2 | 5.6e-84  1.0e-83 | 6 | a,b,c | 23 | <0.01 |  |
| **11062**  (aa1-230) | Carbohydrate sulfotransferase 14 (CHST14) | R4GH42_CHICK  (aa91-343) | 87.4 | 1.4e-62 | 7 | a,b,c | 24 | 0.01 | **0.01** |
| **11075**  (aa1-268)  (aa1-268) | Glycoprotein-N-acetylgalactosamine 3-beta-galactosyltransferase 1 (C1GALT1) | C1GLT_CHICK  (aa30-297)  G1NBQ6_MELGA  (aa30-297) | 97.8  98.5 | 8.6e-127  1.0e-126 | 5 | a,b,c | 13 | <0.01 | **<0.01** |
| **11117**  (aa45-252)  (aa45**-**252) | Beta-galactoside alpha-2,6-sialyltransferase 1 (ST6GAL1) **^1,2,4^** | G3US73_MELGA  (aa1-208)  SIAT1_CHICK  (aa1-208) | 93.8  98.1 | 2.8e-82  1.2e-80 | 2 | a,b,c | 8 | <0.01 | **0.01** |
| **21067**  (aa1-138)  (aa1-138) | Beta-galactoside alpha-2,6-sialyltransferase 1 (ST6GAL1), shares 1 peptide with 21963 | G3US73_MELGA  (aa276-413)  SIAT1_CHICK  (aa276-413) | 96.4  95.7 | 2.4e-66  1.2e-65 | 5 | a,b,c | 36 | 0.02 |  |
| **21963**  (aa1-130)  (aa1-130) | Beta-galactoside alpha-2,6-sialyltransferase 1 (ST6GAL1); shares 1 peptide with 21067 | G3US73_MELGA  (aa210-339)  SIAT1_CHICK  (aa210-339) | 93.1  93.1 | 3.9e-56  5.7e-56 | 1 | a,b,c | 8 | 0.01 |  |
| **11133**  (aa28-243)  (aa29-243) | Similar to annexin A5 (ANXA5) **^1,2^** | ANXA5_CHICK  (aa3-301)  G1NDY6_MELGA  (aa1-298) | 69.9  69.1 | 6.5e-64  2.6e-63 | 12 | a,b,c | 40 | 0.01 | **0.01** |
| **1123**  (aa1-1002)  (aa51-1002) | Hypoxia up-regulated protein 1 (HYOU1) **^1,2,4^**; shares 15 peptides with 1302 | HYOU1_CHICK  (aa1-1002)  G3UTS6_MELGA  (aa1-952) | 100.0  97.3 | 0e0  0e0 | 1 | a,b,c | 13 | <0.01 | **<0.01** |
| **1302**  (aa1-937)  (aa1-938) | Hypoxia up-regulated protein 1 (HYOU1); shares 15 peptides with 1123 | G1MZ54_MELGA  (aa4-900)  HYOU1_CHICK  (aa54-955) | 93.2  91.9 | 93.2e-175  8.2e-172 | 18 | a,b,c | 54 | <0.01 |  |
| **11231**  (aa1-265)  (aa1-265) | Uncharacterized/Neuronal pentraxin receptor (NPTXR) | G1NL88_MELGA  (aa338-602)  E1BUW1_CHICK  (aa389-653) | 99.2  97.7 | 1.2e-116  9.3e-115 | 2 | a,b,c | 4 | <0.01 | **<0.01** |
| **11245**  (aa1-265)  (aa1-265) | Lactadherin/Milk fat globule-EGF factor 8 protein (MFGE8) **^1,2; i^**; shares 10 peptides with 9870, and 15 with 14016 | E1C0K5_CHICK  (aa148-468)  G1N944_MELGA  (aa147-473) | 79.4  76.8 | 2.7e-69  2.7e-69 | 25 | a,b,c | 708 | 0.64 | **0.36** |
| **9870**  (aa25-223)  (aa1-223) | Lactadherin (MFGE8); shares 10 peptides with 11245 | G1N944_MELGA  (aa66-267)  E1C0K5_CHICK  (aa1-268) | 91.1  77.2 | 1.6e-77  8.0e-77 | 3 | a,b,c | 47 | 0.02 |  |
| **14016**  (aa1-216)  (aa1-216) | Lactadherin (MFGE8); shares 15 peptides with 11245 | E1COK5_CHICK  (aa260-468)  G1N944_MELGA  (aa259-473) | 93.1  89.6 | 9.3e-93  8.3e-91 | 3 | a,b,c | 199 | 0.15 |  |
| **11279**  (aa3-182)  (aa3-182) | DJ-1 **^3^**/Putative Parkinson disease autosomal recessive early onset 7 variant 1 (PARK7) | G1MQE7_MELGA  (aa1-180)  D5M8S2_CHICK  (aa1-180) | 97.8  97.8 | 7.8e-69  7.8e-69 | 3 | a,b,c | 8 | <0.01 | **<0.01** |
| **11294**  (aa14-264)  (aa14-264) | Voltage-dependent anion-selective channel protein 2 (VDAC2) **^1^** | Q9I9D1_CHICK  (aa1-283)  G3X8N2_MELGA  (aa1-282) | 86.9  81.6 | 1.2e-81  3.3e-81 | 4 | a,b | 6 | <0.01 | **<0.01** |
| **11307**  (aa1-264)  (aa1-264) | Atrial natriuretic peptide clearance receptor (NPR3) **^1^** | E1C1V5_CHICK  (aa239-521)  G1MVA6_MELGA  (aa2-283) | 82.3  60.8 | 3.3e-81  4.3e-46 | 3 | a,b,c | 13 | <0.01 | **0.07** |
| **12094**  (aa1-237) | Atrial natriuretic peptide receptor 3 (NPR3) | E1C1V5_CHICK  (aa1-237) | 99.2 | 3.0e-103 | 11 | a,b,c | 143 | 0.10 |  |
| **1133**  (aa225-687)  (aa90-1002) | Similar to laminin subunit gamma-1 (LAMC1) **^1,2^**; shares 13 peptides with 1462 | Q90ZN3_CHICK  (aa1-1007)  G1MSJ8_MELGA  (aa1-1473) | 42.8  56.5 | 1.4e-59  5.7e-58 | 17 | a,b,c | 77 | <0.01 | **<0.01** |
| **1462**  (aa1-908)  (aa1-551) | Similar to laminin gamma-1 (LAMC1); shares 13 peptides with 1133 | G1MSJ8_MELGA  (aa387-1473)  Q90ZN3_CHICK  (aa278-1007) | 81.6  73.4 | 3.2e-161  9.2e-69 | 1 | a,b | 3 | <0.01 |  |
| **11361**  (aa1-261)  (aa1-261) | Hyaluronidase (HYAL1) **^1,2^** | G1MRN3_MELGA  (aa152-444)  H9KYW7_CHICK  (152-444) | 79.0  76.8 | 7.7e-62  1.1e-60 | 7 | a,b,c | 18 | 0.01 | **0.01** |
| **11366a**  (aa14-71) | Uncharacterized; domain: proteinase_inhibitor_I47, latexin (aa14-72); | C7G541_CHICK  (aa143-200) | 44.8 | 9.5e-5 | 5 | a,b,c | 374 | 1.05 | **6.32** |
| **11366b**  (aa73-230) | Latexin (LXN) | F1P0G7_CHICK  (aa45-192) | 90.5 | 9.2e-57 | 1 | c | 1 | - | **<0.01** |
| **11387**  (aa1-250)  (aa1-250) | Thrombospondin type-1 domain-containing protein 4 (THSD4) **^1,2^** | G1N0M4_MELGA  (aa187-436)  F1NVQ5_CHICK  (aa564-813) | 98.0  98.0 | 6.1e-119  1.7e-118 | 5 | a,b,c | 15 | <0.01 | **0.01** |
| **15969b**  (aa112-181)  (aa113-181) | Thrombospondin type-1 domain-containing protein 4 (THSD4) | G1N0M4_MELGA  (aa24-93)  F1NVQ5_CHICK  (aa403-471) | 100.0  98.6 | 1.7e-27  1.7e-26 | 2 | a,b,c | 25 | <0.01 |  |
| **11463**  (aa99-251)  (aa47-250) | Similar to uroplakin-3b-like protein (UPK3BL) **^3^** | F1NF48_CHICK  (aa1-152)  G3UUI3_MELGA  (aa47-250) | 75.8  54.4 | 5.5e-48  1.4e-33 | 2 | a,b,c | 7 | <0.01 | **<0.01** |
| **11484**  (aa1-260)  (aa1-260) | Myocilin (MYOC) **^1^** | G1MYB1_MELGA  (aa100-359)  F1NJM9_CHICK  (aa216-475) | 98.5  98.1 | 1.9e-116  1.2e-115 | 9 | a,b,c | 27 | <0.01 | **<0.01** |
| **11515**  (aa8-247)  (aa8-247) | Similar to transmembrane protease serine 4 (TMPRSS4) **^1,2^** | F1N9M8_CHICK  (aa100-379)  G1MXR5_MELGA  (aa102-381) | 61.6  61.6 | 4.3e-27  3.2e-26 | 6 | a,b,c | 19 | 0.01 | **0.01** |
| **1153**  (aa220-882)  (aa220-882) | Similar to suppressor of tumorigenicity protein 14 (ST14) **^1,2^** | G1MR46_MELGA  (aa18-794)  F1NLW7_CHICK  (aa16-792) | 81.0  79.0 | 2.1e-101  8.6e-100 | 8 | a,b,c | 38 | <0.01 | **<0.01** |
| **11531**  (aa3-259) **2058**  (aa699-783) | Polyubiquitin (UBB)/ubiquitin **^1; t^** | UBB_CHICK  (aa1-299)  Q91021_CHICK  (aa5-89) | 82.6  100.0 | 8.7e-81  3.0e-27 | 6 | a,b,c | 225 | 0.08 | **0.12** |
| **11559**  (aa1-228)  (aa1-259) | Guanine nucleotide-binding protein subunit alpha-11 (GNA11) **^1^** | H9H1U8_MELGA  (aa2-243)  H9KZ66_CHICK  (aa2-314) | 86.4  82.7 | 1.6e-83  2.1e-83 | 5 | a,b,c | 14 | <0.01 | **0.01** |
| **11563**  (aa1-217)  (aa1-217) | Follistatin-related protein 4 (FSTL4); shares 2 peptides with 1775 and 1 with 9150 | F1NWP0_CHICK  (aa600-816)  G1N8T8_MELGA  (aa600-816) | 94.0  93.5 | 6.0e-87  1.4e-86 | 1 | b | 1 | <0.01 | **<0.01** |
| **1775**  (aa1-831)  (aa1-831) | SPIG-1B/FSTL4; shares 3 peptides with 9150, and 2 with 11563 | Q71SY9_CHICK  (aa1-183)  G1N8T8_MELGA  (aa1-831) | 100.0  96.4 | 0e0  0e0 | 8 | a,b,c | 22 | <0.01 |  |
| **9150**  (aa3-288)  (aa3-280) | Follistatin-related protein 4 (FSTL4); shares 1 peptide with 11563 and 3 with 1775 | G1N8T8_MELGA  (aa332-623)  F1NWP0_CHICK  (aa388-701) | 80.3  79.9 | 3.9e-51  1.8e-49 | 1 | b,c | 2 | <0.01 |  |
| **11592**  (aa20-250)  (aa20-250) | UDP-glucuronic acid decarboxylase 1 (UXS1) **^1,2^** | G1NPP3_MELGA  (aa36-266)  E1BV28_CHICK  (aa94-324) | 100.0  100.0 | 4.2e-98  5.0e-98 | 6 | a,b,c | 59 | 0.06 | **0.10** |
| **11593**  (aa1-258)  (aa1-255) | Similar to P2X purinoceptor (P2RX7) **^2^** | E1C6P3_CHICK  (aa294-573)  G3UPY6_MELGA  (aa280-560) | 75.4  68.3 | 2.0e-88  3.4e-76 | 5 | a,b,c | 18 | <0.01 | **0.01** |
| **11611**  (aa1-258)  (aa1-258) | Signal recognition particle receptor subunit alpha (SRPR) **^1,2^** | F1NXV4_CHICK  (aa387-644)  G1MPZ3_MELGA  (aa387-644) | 100.0  99.6 | 2.0e-94  2.8e-94 | 6 | a,b,c | 11 | <0.01 | **<0.01** |
| **11636**  (aa7-257)  (aa7-257) | Catalase (Gga.48383/CAT) **^2^** | F1NGJ7_CHICK  (aa152-402)  G1NCW7_MELGA  (aa193-445) | 96.0  95.3 | 9.1e-107  3.1e-106 | 3 | a,b,c | 7 | <0.01 | **<0.01** |
| **11650**  (aa30-257)  (aa30-257) | Similar to Ras-related protein Rab-1A (RAB1A) **^1,2^** | G1MZK2_MELGA  (aa30-202)  F6UGI5_CHICK  (aa33-205) | 75.9  75.9 | 4.1e-55  4.2e-55 | 3 | a,b,c | 6 | <0.01 | **<0.01** |
| **11663**  (aa1-218)  (aa1-218) | CD81 antigen/tetraspanin (CD81) | G1N786_MELGA  (aa1-218)  F1NW06_CHICK  (aa1-217) | 98.2  95.0 | 2.7e-96  6.7e-92 | 6 | a,b,c | 20 | <0.01 | **<0.01** |
| **11670**  (aa22-257)  (aa22-257) | Repulsive guidance molecule A (RGMA) **^1,2^** | RGMA_CHICK  (aa197-432)  G1NA39_MELGA  (aa181-416) | 98.7  98.3 | 3.4e-106  1.8e-105 | 6 | a,b,c | 10 | <0.01 | **<0.01** |
| **11689**  (aa1-256)  (aa1-256) | Similar to sodium-coupled monocarboxylate transporter 1 (SLC5A8) **^2^** | F1P5L0_CHICK  (aa1-607)  G1NGQ1_MELGA  (aa7-615) | 40.7  38.6 | 7.9e-35  1.0e-35 | 2 | a,c | 3 | <0.01 | **<0.01** |
| **1170**  (aa1-958)  (aa1-973) | Similar to agrin (AGRN) **^1,2^** ; shares 16 peptides with 166 and 15 with 804 | F1NWQ6_CHICK  (aa177-1385)  G1MWK4_MELGA  (aa20-1244) | 77.9  76.9 | 1.8e-123  5.8e-123 | 1 | a,c | 3 | <0.01 | **0.01** |
| **166**  (aa1-1955)  (aa40-1869) | Agrin (isoform 4; P31696-4?); shares 29 peptides with 804, 15 with 5416 and 17 with 1170 | AGRIN_CHICK  (aa119-2081)  G1MWK4_MELGA  (aa1-1833) | 99.6  97.0 | 0e0  0e0 | 44 | a,b,c | 219 | 0.01 |  |
| **23251**  (aa1-100)  (aa13-100)  **18626** | Agrin | F1NWQ6_CHICK  (aa50-149)  G1N321_MELGA  (aa1-88) | 100.0  100.0 | 1.3e-43  1.0e-37 | 4 | a,c | 16 | 0.01 |  |
| **804**  (aa1-1132)  (aa1-1107) | Similar to agrin; shares 29 peptides with 166 and 14 with 1170 | F1NWQ6_CHICK  (aa177-2065)  G1MWK4_MELGA  (aa20-1808) | 59.0  55.8 | 4.1e-103  5.1e-100 | 10 | a,b,c | 70 | 0.01 |  |
| **11716**  (aa21-220)  (aa21-256) | N-sulfoglucosamine sulfohydrolase **^2^** | F1NGI8_CHICK  (aa113-312)  G1N333_MELGA  (aa73-390) | 91.5  40.3 | 9.3e-82  1.4e-14 | 4 | a,b,c | 11 | <0.01 | **<0.01** |
| **1172**  (aa30-991)  (aa30-991) | Similar to myoferlin (MYOF) | G1N9U8_MELGA  (aa165-2019)  E1BW21_CHICK  (aa145-1997) | 48.1  49.6 | 6.7e-93  5.4e-92 | 2 | a,b,c | 3 | <0.01 | **<0.01** |
| **11745**  (aa51-240)  (aa40-240) | Torsin-2A (TOR2A) **^2^**; shares 2 peptides with 16741 | R4GIY3_CHICK  (aa16-205)  G1MXE4_MELGA  (aa1-201) | 94.7  90.6 | 6.1e-76  1.4e-74 | 2 | a,c | 2 | <0.01 | **<0.01** |
| **16741**  (aa1-182)  (aa1-182) | Torsin-2A; shares 2 peptides with 11745 | G3UPY3_MELGA  (aa69-250)  R4GIY3_CHICK  (aa104-285) | 98.4  96.7 | 1.6e-80  4.6e-79 | 4 | a,b,c | 10 | 0.01 |  |
| **11775**  (aa1-255) | Similar to alpha-N-acetylglucosaminidase; domains: alpha-N-acetylglucosaminidase  (A0A091HMN2_CALAN aa262-558, 73.1%, 2.5e-51) |  |  |  | 2 | a,c | 5 | <0.01 | **<0.01** |
| **11800**  (aa1-254)  (aa142-254) | Cartilage-associated protein (CRTAP) **^2^**; shares 2 peptide with 9604 | E1C7V3_CHICK  (aa1-254)  G1NGW5_MELGA  (aa1-113) | 98.4  100.0 | 2.5e-114  4.7e-46 | 1 | a | 1 | <0.01 | **<0.01** |
| **9604**  (aa54-303)  (aa1-303) | Cartilage-associated protein (CRTAP); shares 2 peptides with 11800 | G1NGW5_MELGA  (aa3-252)  F1NKI1_CHICK  (aa1-393) | 98.4  76.1 | 2.1e-110  8.7e-110 | 4 | a,b | 4 | <0.01 |  |
| **11810**  (aa37-254)  (aa37-254) | Similar to ovotransferrin/melanotransferrin (MFI2) **^1^**; shares 1 peptide with 2343 and 1 with 15203 | G3UQM4_MELGA  (aa67-359)  F1NVN3_CHICK  (aa67-359) | 70.6  70.6 | 4.3e-48  2.2e-47 | 2 | a | 4 | <0.01 | **<0.01** |
| **15203**  (aa1-197)  (aa1-197) | Similar to ovotransferrin/melanotransferrin (MFI2); shares 1 peptide with 11810 | G3UQM4_MELGA  (aa102-445)  F1NVN3_CHICK  (aa102-445) | 53.5  53.2 | 1.2e-53  7.9e-53 | 1 | a,c | 3 | <0.01 |  |
| **2343**  (aa1-738)  (aa1-738) | Ovotransferrin/melanotransferrin (EOS47/MFI2); shares 1 peptide with 11810 | Q92062_CHICK  (aa1-738)  G3UQM4_MELGA  (aa1-738) | 99.9  95.4 | 0e0  0e0 | 3 | a,b,c | 9 | <0.01 |  |
| **11834**  (aa22-253)  (aa18-253) | Chloride intracellular channel protein 4 (CLIC4) **^1,2^**; shares 2 peptides with 9750 (CLIC6) | G1MT68_MELGA  (aa13-244)  F1NYZ7_CHICK  (aa10-245) | 98.7  97.0 | 1.2e-96  1.7e-96 | 13 | a,b,c | 38 | <0.01 | **<0.01** |
| **11915**  (aa1-252)  (aa1-252) | Similar to Exostosin-1 (EXTL1) **^1^** | R4GFV1_CHICK  (aa94-315)  G1MUS2_MELGA  (aa91-311) | 82.9  55.2 | 6.9e-63  3.5e-40 | 6 | a,b,c | 28 | 0.01 | **0.01** |
| **11921**  (aa1-251) | Uncharacterized/Solute carrier family 25 , member 4 (SLC15A4) | Q5ZMJ6_CHICK  (aa1-298) | 81.5 | 7.2e-82 | 2 | a,b,c | 6 | <0.01 | **<0.01** |
| **11943**  (aa55-251)  (aa55-251) | Beta-1,3-N-acetylglucosaminyltransferase lunatic fringe (LNFG) | G1MUY6_MELGA  (aa19-215)  F1NZ16_CHICK  (aa57-253) | 100.0  100.0 | 4.1e-89  5.0e-89 | 5 | a,b,c | 8 | <0.01 | **<0.01** |
| **11968**  (aa1-216)  (aa2-214) | Similar to alpha-L-iduronidase (IDUA) **^2^** | Q5F366_CHICK  (aa117-457)  G1N693_MELGA  (aa78-415) | 54.5  50.0 | 2.1e-23  2.2e-20 | 1 | a,c | 6 | <0.01 | **<0.01** |
| **3233**  (aa1-630)  (aa42-630) | Alpha-L-iduronidase (IDUA); shares 5 peptides with 6000 | Q5F366_CHICK  (aa1-630)  G1N693_MELGA  (aa1-593) | 100.0  86.3 | 0e0  0e0 | 1 | a | 3 | <0.01 |  |
| **6000**  (aa1-426)  (aa1-426) | Alpha-L-iduronidase (IDUA); shares 5 peptides with 3233 | Q5F366_CHICK  (aa120-630)  G1N693_MELGA  (aa120-630) | 78.1  73.7 | 1.8e-153  1.4e-145 | 7 | a,b,c | 17 | <0.01 |  |
| **11974**  (aa1-250)  (aa1-250) | Keratin 19 (KRT19) **^1,2^**; shares 4 peptides 16665, 1 with 4638 and 6 with CON | F1NDN9_CHICK  (aa155-404)  G1MVR3_MELGA  (aa153-402) | 98.0  96.8 | 2.2e-74  6.9e-74 | 4 | a,c | 8 | <0.01 | **<0.01** |
| **16665**  (aa1-170)  (aa1-170) | Keratin, type I cytoskeletal 19 (KRT19); shares 4 peptides with 11974, 1 with 4638 and 5 with CON | F1NDN9_CHICK  (aa57-226)  G1MVR3_MELGA  (aa56-224) | 98.2  92.4 | 1.9e-52  3.9e-48 | 2 | a,b,c | 18 | <0.01 |  |
| **11995**  (aa1-232)  (aa1-231) | Protocadherin Fat 1 (FAT1) **^1,2^**; shares 2 peptides with 72 | F1NWW5_CHICK  (aa3079-3286)  G1NG27_MELGA  (aa3080-3286) | 87.5  85.7 | 11e-48  6.7e-47 | 1 | a,b,c | 5 | <0.01 | **0.01** |
| **2159**  (aa1-767)  (aa1-767) | Protocadherin Fat 1 | G1NG27_MELGA  (aa3886-4592)  F1NWW5_CHICK  (aa3884-4602) | 90.9  91.3 | 3.6e-162  4.5e-162 | 2 | a,b | 5 | <0.01 |  |
| **376**  (aa1-1427)  (aa1-1427) | Protocadherin Fat 1 | G1NG27_MELGA  (aa1-1451)  F1NWW5_CHICK  (aa1-1450) | 96.0  95.9 | 0e0  0e0 | 49 | a,b,c | 229 | 0.01 |  |
| **483**  (aa1-1355)  (aa1-1355) | Protocadherin Fat 1; shares 28 peptides with 72 | F1NWW5_CHICK  (aa1606-2960)  G1NG27_MELGA  (aa1607-2961) | 96.3  95.8 | 0e0  0e0 | 12 | a,b,c | 46 | <0.01 |  |
| **72**  (aa1-2389)  (aa1-2389) | Similar to protocadherin Fat 1; shares28 peptides with 483 and 2 with 11995 | F1NWW5_CHICK  (aa1494-4507)  G1NG27_MELGA  (aa1495-4497) | 63.3  75.8 | 0e0  75.8 | 47 | a,b,c | 174 | 0.01 |  |
| **1204b**  (aa607-983)  (aa751-983) | Alpha-(1,3)-fucosyltransferase 11 (FUT11) **^2^** | FUT11_CHICK  (aa81-456)  G1N423_MELGA  (aa1-232) | 95.2  95.3 | 3.6e-171  5.3e-101 | 5 | a,b,c | 8 | <0.01 | **<0.01** |
| **12073**  (aa1-249) | Collagen alpha-1(II) chain **^1^** (non-triple helical fragment; COL2A1) | CO2A1_CHICK  (aa121-369) | 99.6 | 2.0e-115 | 6 | a,b,c | 14 | <0.01 | **<0.01** |
| **12106**  (aa1-248)  (aa1-248) | Similar to ovoinhibitor (OIH) **^1,2,4; t^**; shares 2 peptides with 7445 | IOV7_CHICK  (aa20-399)  G1MZX1_MELGA  (aa20-401) | 55.5  55.2 | 2.3e-76  3.4e-65 | 1 | a,c | 2 | <0.01 | **0.01** |
| **7445**  (aa56-365)  (aa56-365) | Ovoinhibitor; shares 2 peptides with 12106 | F1NMN2_CHICK  (aa25-334)  G1MZX1_MELGA  (aa24-335) | 95.8  72.5 | 3.9e-120  2.5e-100 | 12 | a,b,c | 44 | 0.01 |  |
| **12114**  (aa56-248)  (aa56-248) | Rho-related GTP-binding protein RhoC (RHOC) **^1^**; shares 3 peptides with 24897 (RHOA) | RHOC_CHICK  (aa1-193)  G1N0A5_MELGA  (aa1-193) | 100.0  99.5 | 1.6e-75  3.9e-75 | 7 | a,b,c | 16 | <0.01 | **<0.01** |
| **12122**  (aa1-248)  (aa1-248) | Stanniocalcin-2 (STC2) **^1^** | G1MX28_MELGA  (aa2-249)  E1BRJ2_CHICK  (aa54-301) | 97.6  97.2 | 8.9e-109  2.2e-108 | 5 | a,b,c | 5 | <0.01 | **<0.01** |
| **12152**  (aa1-230) | Polymeric immunoglobulin receptor (PIGR) **^1,2^** | G3UTW1_MELGA  (aa357-585) | 88.7 | 2.6e-85 | 3 | a,b,c | 9 | <0.01 | **0.01** |
| **12206**  (aa1-246)  (aa1-246) | Annexin (ANXA8) **^1,2^**; shares 3 peptides with 3812 and 1 with 23558 (ANXA4) | E1C8K3_CHICK  (aa8-248)  G1N762_MELGA  (aa8-275) | 84.1  75.1 | 3.8e-75  6.2e-61 | 7 | a,b,c | 10 | <0.01 | **<0.01** |
| **3812**  (aa262-520)  (aa262-520) | Similar to annexin (ANXA8); shares 3 peptides with 12206 and 1 with 23558 (ANXA4) | G1N762_MELGA  (aa8-351)  E1C8K3_CHICK  (aa8-324) | 59.9  65.6 | 1.5e-32  2.6e-32 | 4 | a,b,c | 7 | <0.01 |  |
| **1228a**  (aa3-316)  (aa3-316) | Similar to fibrinogen gamma chain (FGG) **^1,2^**; shares 2 peptides with 1311b | E1BV78_CHICK  (aa45-433)  G1MRP5_MELGA  (aa41-430) | 76.6  75.6 | 2.2e-113  4.8e-112 | 2 | a | 2 | <0.01 | **<0.01** |
| **1311b**  (aa123-330)  (aa123-330) | Unchachacterized/similar to fibrinogen gamma chain (FGG); shares 2 peptides with 1228 | G1MRP5_MELGA  (aa133-427  E1BV78_CHICK  (aa137-430) | 63.4  64.4 | 3.7e-41  7.1e-41 | 2 | a,b,c | 4 | <0.01 |  |
| **1228b**  (aa317-976)  (aa317-976) | Fibrinogen alpha chain (FGA) **^1,2^**; shares 4 peptides with 1311c | F1P4V1_CHICK  (aa18-741)  G3UQB2_MELGA  (aa18-740) | 83.8  84.3 | 0e0  0e0 | 2 | a,c | 5 | <0.01 | **<0.01** |
| **1311c**  (aa333-951)  (aa333-951) | Fibrinogen alpha-chain; shares 4 peptides with 1228b | G3UQB2_MELGA  (aa1-618)  F1P4V1_CHICK  (aa170-741) | 85.4  85.4 | 5.1e-127  6.9e-123 | 5 | a,b,c | 11 | <0.01 |  |
| **12309**  (aa66-216)  (aa69-207) | Ras-related protein R-Ras2 (RRAS2) **^2^** | Q5ZIW8_CHICK  (aa1-151)  G1N459_MELGA  (aa2-140) | 96.6  100.0 | 5.3e-51  2.0e-50 | 3 | a,b,c | 11 | <0.01 | **<0.01** |
| **20781**  (aa1-140)  (aa1-140)  **12358** | 14-3-3 protein beta/alpha (YWHAB) **^2^**; shares 1 peptide with 14-3-3 proteins | 1433B_CHICK  (aa1-140)  G1MXY2_MELGA  (aa3-142) | 99.3  99.3 | 3.8e-50  3.9e-50 | 6 | a,b,c | 18 | <0.01 | **<0.01** |
| **1237**  (aa1-971)  (aa24-971) | Calsyntenin-1 (CLSTN1) **^2^** | E1BYQ5_CHICK  (aa1-971)  G1MXV5_MELGA  (aa1-949) | 100.0  99.6 | 0e0  0e0 | 4 | a,b,c | 12 | <0.01 | **<0.01** |
| **12381**  (aa1-243)  (aa1-243) | Alpha-1,3-mannosyl-glycoprotein 4-beta-N-acetylglucosaminyltransferase (MGAT4D) | G1MVL2_MELGA  (aa66-504)  F1NPD2_CHICK  (aa87-513) | 50.8  50.1 | 2.6e-61  1.4e-58 | 4 | a,b,c | 14 | <0.01 | **<0.01** |
| **1249**  (aa1-934)  (aa1-934) | Similar to thrombospondin-1 (THSB1) **^2^**; shares 14 peptides with 724, and 4 with CON | F1P0J8_CHICK  (aa1-1173)  G1NHK4_MELGA  (aa4-1179) | 76.1  75.6 | 2.1e-97  2.5e-96 | 19 | a,b,c | 75 | 0.01 | **<0.01** |
| **724**  (aa1-1175)  (aa1-1173) | Thrombospondin-1 (THSB1); shares 14 peptides with 1249 and 4 with CON | F1P0J8_CHICK  (aa1-1175)  G1NHK4_MELGA  (aa4-1179) | 100.0  97.0 | 0e0  0e0 | 2 | a,b,c | 19 | <0.01 |  |
| **1250**  (aa1-967) | Aminopeptidase N (ANPEP) **^1,2^** | AMPN_CHICK  (aa1-967) | 100.0 | 0e0 | 13 | a,b,c | 83 | 0.01 | **0.01** |
| **19777**  (aa30-150) | Aminopeptidase N | AMPN_CHICK  (aa84-204) | 89.3 | 4.8e-45 | 1 | a,b,c | 7 | <0.01 |  |
| **12560**  (aa1-240)  (aa1-240) | Annexin (ANXA7) | G1N4G4_MELGA  (aa174-413)  E1C1D1_CHICK  (aa142-381) | 99.2  98.8 | 1.9e-98  6.3e-98 | 2 | a,b | 3 | <0.01 | **<0.01** |
| **12566**  (aa32-239)  (aa64-239) | Uncharacterized **^2^**; domains: IG/FN3; shares 3 peptides with 3276 | G1MX70_MELGA  (aa616-829)  F1P3M4_CHICK  (aa616-829) | 72.5  76.3 | 1.9e-51  1.5e-43 | 1 | a,b,c | 7 | <0.01 | **0.01** |
| **3276**  (aa43-617)  (aa44-460) | Uncharacterized; domains: VWA, IG, collagen_triple_helical_repeats; shares 3 peptides with 12566 | F1P3M4_CHICK  (aa5-1374)  G1MX70_MELGA  (aa9-847) | 36.1  42.6 | 3.3e-27  4.9e-27 | 11 | a,b,c | 84 | 0.01 |  |
| **12571**  (aa1-239)  (aa1-239) | T-complex protein 1 subunit alpha (TCP1) | Q5ZMG9_CHICK  (aa54-327)  G1NIP3_MELGA  (aa54-327) | 85.5  85.5 | 3.5e-61  3.5e-61 | 2 | a,b,c | 3 | <0.01 | **<0.01** |
| **17561**  (aa1-172)  (aa1-172) | Similar to T-complex protein 1 subunit alpha (TCP1) | G1NIP3_MELGA  (aa434-558)  Q5ZMG9_CHICK  (aa434-558) | 72.7  72.7 | 5.5e-25  5.5e-25 | 2 | b,c | 2 | <0.01 |  |
| **12584**  (aa1-233)  (aa1-233) | Hephaestin (HEPH); shares 2 peptides with 1734 | F1NPE0_CHICK  (aa271-503)  G1MQ95_MELGA  (aa271-503) | 94.8  94.8 | 2.0e-102  3.9e-102 | 1 | a | 1 | <0.01 | **<0.01** |
| **1734**  (aa10-842)  (aa10-839) | Similar to hephaestin; shares 2 peptides with 12584 | F1NPE0_CHICK  (aa139-1158)  G1MQ95_MELGA  (aa139-1157) | 77.8  77.0 | 1.2e-152  5.2e-152 | 4 | a,b | 4 | <0.01 |  |
| **12590**  (aa1-203)  (aa1-203) | Carboxypeptidase Q (CPQ)/syndecan-2 (SDC2) **^2^** | G1NHR0_MELGA  (aa10-212)  E1BUR8_CHICK  (aa46-248) | 97.0  94.1 | 7.9e-77  3.9e-75 | 8 | a,b,c | 53 | 0.03 | **0.03** |
| **14217**  (aa9-213)  (aa9-213)  **12604** | Similar to N-acetylglucosamine-6-sulfatase (NGS) **^1,2^** | F1NI04_CHICK  (aa249-511)  G1ND08_MELGA  (aa199-461) | 69.8  69.8 | 1.3e-40  3.9e-40 | 7 | a,b,c | 37 | 0.03 | **0.03** |
| **12646**  (aa149-238)  (aa1-238) | Inositol monophosphatase domain containing 1 (IMPAD1) **^1,2,4^** | G1NEV7_MELGA  (aa140-229)  E1C648_CHICK  (aa1-356) | 100.0  65.7 | 6.7e-32  2.2e-31 | 8 | a,b,c | 24 | 0.01 | **0.01** |
| **1268**  (aa490-560)  (aa490-560) | Vesicle-associated membrane protein 3 (VAMP3) **^1^** | G1MQN9_MELGA  (aa27-97)  F1P4I3_CHICK  (aa49-119) | 98.6  98.6 | 6.3e-25  7.8e-25 | 2 | a,b | 7 | <0.01 | **<0.01** |
| **12732**  (aa46-237)  (aa46-237) | Similar to vitamin-D binding protein (GC) **^1,2; g,t^** | F1NVF3_CHICK  (aa89-467)  G1NCF1_MELGA  (aa89-467) | 47.5  47.0 | 4.4e-50  3.2e-48 | 8 | a,b,c | 60 | 0.02 | **0.02** |
| **12776**  (aa30-218)  (aa30-218) | Uncharacterized/similar to Oxygen-regulated protein 1 (RP1L1) | G1NN18_MELGA  (aa6-194)  R4GFE8_CHICK  (aa6-194) | 48.1  48.1 | 2.5e-35  2.6e-35 | 2 | a,b,c | 5 | <0.01 | **<0.01** |
| **1281**  (aa1-958)  (aa1-958) | Coiled-coil domain-containing protein 80 (CCD80) **^4^**; shares 5 peptides with 19036 | CCD80_CHICK  (aa1-958)  G1NNF1_MELGA  (aa1-958) | 100.0  98.2 | 0e0  0e0 | 15 | a,b,c | 66 | <0.01 | **<0.01** |
| **19036**  (aa1-130)  (aa1-130) | CCD80; shares 5 peptides with 1281 | F1NTI1_CHICK  (aa688-817)  G1NNF1_MELGA  (aa688-817) | 99.2  99.2 | 4.3e-52  4.9e-52 | 1 | a,b | 4 | <0.01 |  |
| **12840**  (aa1-232)  (aa1-232) | Glypican-5 (LOC429143/LOC100543530) | F1NZX2_CHICK  (aa95-326)  G1MQ74_MELGA  (aa9-240) | 95.7  94.4 | 2.5e-97  7.3e-97 | 3 | a,b,c | 11 | <0.01 | **<0.01** |
| **12865**  (aa60-234)  (aa68-234) | Similar to beta-galactosidase-1-like protein (GLB1) **^1,2^** | Q5ZLM4_CHICK  (aa11-237)  G3UTX9_MELGA (aa3-221) | 72.7  73.5 | 1.2e-45  1.2e-43 | 4 | a,b,c | 26 | 0.02 | **0.02** |
| **12923**  (aa3-179)  (aa4-179) | Signal peptide, CUB and EGF-like domain-containing protein 1 (Fragment; SCUBE1) **^1,2^** | F1NZ94_CHICK  (aa485-661)  G1NLZ2_MELGA  (aa336-511) | 98.9  99.4 | 9.5e-71  2.3e-70 | 2 | a,b,c | 26 | 0.04 | **0.05** |
| **12944**  **(**aa19-233) | Ras-related protein Rab-11B (RAB11B) **^1^**; shares 1 peptide with 21796 and 1 with 20214 (RAB11A) | Q5F3R8_CHICK  (aa8-218) | 96.3 | 2.0e-72 | 6 | a,b,c | 14 | <0.01 | **<0.01** |
| **21797**  (aa1-132) | Similar to Ras-related protein Rab-11B; shares 1 peptide with 12944 and 2 with 20214 (RAB11A) | Q5F3R8_CHICK  (aa1-218) | 59.2 | 5.9e-24 | 2 | a,b,c | 9 | 0.01 |  |
| **12994**  (aa1-232)  (aa1-232) | Heparan-sulfate 6-O-sulfotransferase 2 (HS6ST2) **^1,4^** | F1NAU6_CHICK  (aa170-401)  G1MVT1_MELGA  (aa169-400) | 99.1  98.7 | 1.0e-92  2.6e-92 | 3 | a,b | 4 | <0.01 | **<0.01** |
| **13**  (aa1-4071)  (aa308-4071) | Basement membrane-specific heparan sulfate proteoglycan core protein (HSPG/HSPG2) **^1,2^**; shares 10 peptides with 2044, 9 with 1980, 8 with 5189, and 2 with18735 | Q6KDZ1_CHICK  (aa1-4071)  G1N5Z4_MELGA  (aa1-2858) | 100.0  63.4 | 0e0  3.6e-198 | 56 | a,b,c | 301 | <0.01 | **<0.01** |
| **18735**  (aa17-160) | Basement membrane-specific heparan sulfate proteoglycan core protein (HSPG); shares 2 peptides with 13 | Q6KDZ1_CHICK  (aa2671-2814) | 95.8 | 1.6e-53 | 1 | c | 3 | <0.01 |  |
| **1980**  (aa6-787)  (aa71-729) | Similar to basement membrane-specific heparan sulfate proteoglycan core protein (HSPG/HSPG2); shares 9 peptides with 13 | Q6KDZ1_CHICK  (aa432-2243)  G1N5Z4_MELGA  (aa149-1893) | 39.4  37.8 | 1.6e-79  7.3e-65 | 6 | a,b,c | 36 | <0.01 |  |
| **2044**  (aa1-764)  (aa2-764) | Similar to basement membrane-specific heparan sulfate proteoglycan core protein (HSPG/HSPG2); shares peptides with 13 | Q6KDZ1_CHICK  (aa2529-3744)  G3US27_MELGA  (aa10-432) | 58.4  80.3 | 6.3e-140  7.6e-124 | 9 | a,b,c | 23 | <0.01 |  |
| **13022**  (aa1-232)  (aa1-232) | Alpha-amylase (AMYA2) **^1,2^**; shares 2 peptides with 10098 | G3UQ12_MELGA  (aa281-512)  F1NF53_CHICK  (aa281-512) | 94.0  93.5 | 6.0e-110  5.7e-109 | 4 | a,b,c | 13 | 0.01 | **0.01** |
| **17747**  (aa1-171)  (aa1-171) | Alpha-amylase (AMY2A) | F1NF53_CHICK  (aa1-171)  G3UQ12_MELGA  (aa1-171) | 97.1  95.9 | 1.8e-80  5.9e-79 | 2 | a,b,c | 9 | <0.01 |  |
| **1309**  (aa153-952)  (aa149-952) | Similar to villin (VILL) **^1,2^**; shares 1 peptide with 13976 | G1MWE6_MELGA  (aa1-856)  E1C5U6_CHICK  (aa1-860) | 71.5  72.1 | 6.7e-76  6.8e-76 | 3 | a,b,c | 12 | <0.01 | **<0.01** |
| **13976**  (aa1-199)  (aa1-199) | Villin; shares 1 peptide with 1309 | E1C5U6_CHICK  (aa115-313)  G1MWE6_MELGA  (aa111-309) | 99.5  99.0 | 9.9e-82  3.1e-81 | 1 | a,b,c | 3 | <0.01 |  |
| **1311a**  (aa26-81)  (aa26-81 | EGF-like-domain 7 (EGFL7) **^2^** | E1C7R1_CHICK  (aa213-268)  G1N698_MELGA  (aa213-268) | 98.2  98.2 | 8.6e-17  8.6e-17 | 3 | a,b,c | 16 | <0.01 | **<0.01** |
| **13115**  (aa9-225)  (aa2-232) | Dipeptidyl peptidase (DPP7) **^1,2^** | G1MQD8_MELGA  (aa2-232)  E1BZ81_CHICK  (aa55-292) | 90.5  87.8 | 8.8e-50  1.0e-48 | 9 | a,b,c | 43 | 0.02 | **0.02** |
| **13133**  (aa1-230)  (aa1-230) | Neuroendocrine convertase 2(PCSK2) **^1,2^**; shares 5 peptides with 7094 | F1NUG0_CHICK  (aa415-644)  G1MYW7_MELGA  (aa335-564) | 99.1  98.7 | 1.3e-103  1.3e-103 | 7 | a,b,c | 43 | 0.02 | **0.01** |
| **7094**  (aa99-378)  (aa99-378) | Similar to proprotein convertase subtilisin/kexin type 2 (PCSK2); shares 5 peptides with 13133 | G1MYW7_MELGA  (aa163-564)  F1NUG0_CHICK  (aa243-644) | 68.4  68.7 | 3.1e-63  3.1e-63 | 2 | a,b,c | 9 | <0.01 |  |
| **13142**  (aa52-230) | Similar to alpha-mannosidase; domains: Glyco_hydro_38/57_N, Glyco_hydro_38_C  (A0A091GF09_9AVES aa367-556, 52.1%, 3.1e-33) |  |  |  | 3 | a,b,c | 13 | 0.01 | **0.02** |
| **13275**  (aa1-214)  (aa1-214) | Netrin-G1 (NTNG1) | G1N3N2_MELGA  (aa83-196)  E1BWU4_CHICK  (aa83-296) | 100.0  99.5 | 1.7e-99  5.8e-99 | 3 | a,b,c | 4 | <0.01 | **<0.01** |
| **13315**  (1-124) | Similar to claudin-23 (CLDN23) | G1NAK5_MELGA  (aa11-248) | 73.2 | 9.8e-68 | 2 | a,b,c | 5 | <0.01 | **<0.01** |
| **13355**  (aa1-226)  (aa1-226) | Ectonucleoside triphosphate diphosphohydrolase 8 (ENTPD8) | G1MRQ1_MELGA  (aa263-495)  F1NJE8_CHICK  (aa261-493) | 89.7  89.7 | 7.3e-99  1.2e-98 | 1 | a,b,c | 8 | 0.01 | **0.01** |
| **22102**  (aa1-129)  (aa1-129) | Ectonucleoside triphosphate diphosphohydrolase 8 (ENTPD8) | ENTP8_CHICK  (aa1-129)  G1MRQ1_MELGA  (aa3-131) | 91.5  90.7 | 1.4e-48  3.9e-48 | 1 | a,c | 4 | <0.01 |  |
| **13480**  (aa37-217)  (aa37-224) | Similar to peptidyl-glycine alpha-amidating monooxygenase (PAM) | G1N537_MELGA  (aa123-332)  F1NQN1_CHICK  (aa268-478) | 60.4  87.2 | 2.1e-39  3.0e-39 | 1 | a,b,c | 6 | <0.01 | **0.01** |
| **14851**  (aa1-205)  (aa57-205) | Similar to peptidyl-glycine alpha-amidating monooxygenase (PAM) | F1NQN1_CHICK  (aa491-970)  G1N537_MELGA  (aa463-833) | 37.3  38.0 | 9.4e-26  8.4e-25 | 4 | a,b,c | 33 | 0.01 |  |
| **13499**  (aa1-224)  (aa1-224) | Iduronate 2-sulfatase (IDS); shares 1 peptide with 5974 | F1NFI0_CHICK  (aa378-601)  G1N835_MELGA  (aa301-524) | 90.2  87.9 | 2.0e-94  1.8e-93 | 1 | b | 1 | <0.01 | **<0.01** |
| **5974**  (aa16-428)  (aa14-428) | Similar to iduronate 2-sulfatase (IDS); shares 1 peptide with 13499 | G1N835_MELGA  (aa1-524)  F1NFI0_CHICK  (aa76-601) | 75.0  75.3 | 7.6e-96  6.0e-94 | 5 | a,c | 7 | <0.01 |  |
| **13512**  (aa3-223)  (aa16-223) | Phosphoglycerate mutase 1 (PGAM1) **^1,2^** | PGAM1_CHICK  (aa33-254)  G1NBW3_MELGA  (aa5-212) | 93.7  97.1 | 2.6e-88  1.8e-87 | 5 | a,b,c | 9 | <0.01 | **<0.01** |
| **13580**  (aa1-222)  (aa1-222) | Heat shock 70 kDa protein 13 (HSPA13) **^1,2,4^**; shares 6 peptides with 16304 | F1ND59_CHICK  (aa251-472)  G1NNS3_MELGA  (aa251-472) | 96.4  93.7 | 1.1e-80  6.5e-77 | 11 | a,b,c | 45 | 0.04 | **0.03** |
| **16304**  (aa1-165)  (aa1-165) | Similar to HSP70-13 (HSPA13); shares 6 peptides with 13580 | Q5F497_CHICK  (aa191-457)  G1NNS3_MELGA  (aa195-461) | 58.4  58.1 | 2.7e-33  3.2e-33 | 2 | a,b,c | 12 | 0.02 |  |
| **13582**  (aa13-209)  (aa35-209) | Plasma protease C1 inhibitor (SERPING1) **^1,2^** | G1NBE9_MELGA  (aa67-263)  F1NA58_CHICK  (aa107-283) | 86.8  84.7 | 4.2e-70  3.5e-58 | 5 | a,b,c | 26 | 0.02 | **0.03** |
| **26678**  (aa11-93)  (aa1-93)  **24662** | Plasma protease C1 inhibitor (SERPING1) | F1NA58_CHICK  (aa405-487)  G1NBE9_MELGA  (aa375-467) | 92.8  83.9 | 4.0e-30  7.8e-30 | 3 | a,b,c | 6 | 0.03 |  |
| **13673**  (aa1-221)  (aa1-220) | Similar to gonadotropin releasing hormone receptor 2 (GNRHR1/III) **^1^** | B5G4W1_CHICK  (aa184-354)  G1NDI1_MELGA  (aa50-219 | 71.9  71.9 | 2.2e-56  2.8e-53 | 2 | a,b,c | 12 | 0.01 | **0.01** |
| **13677**  (aa2-216)  (aa1-221) | Similar to transmembrane BAX inhibitor motif-containing protein 1-like protein (TMBIM1) | G1NG94_MELGA  (aa55-282)  E1BTK0_CHICK  (aa61-311) | 68.1  63.5 | 1.5e-23  2.1e-22 | 2 | a,b | 4 | <0.01 | **<0.01** |
| **13681**  (aa1-221)  (aa1-221) | Similar to FRAS1-related extracellular matrix protein 2 (FREM2) **^1,2^**; shares 1 peptide with 25199 | G1NQ83_MELGA  (aa309-656)  F1P4P1_CHICK  (aa2067-2414) | 62.6  62.4 | 4.4e-34  1.2e-33 | 5 | a,b,c | 17 | <0.01 | **0.01** |
| **13902**  (aa1-218)  (aa1-218) | FRAS1-related extracellular matrix protein 2 (FREM2) | G1NQ83_MELGA  (aa759-976)  F1P4P1_CHICK  (aa2518-2736) | 99.1  99.1 | 1.5e-97  1.1e-96 | 7 | a,b,c | 52 | 0.02 |  |
| **14827**  (aa1-202)  (aa1-202) | FRAS1-related extracellular matrix protein 2 (FREM2) | F1P4P1_CHICK  (aa1814-2015)  G1NQ83_MELGA  (aa56-257) | 99.0  97.0 | 1.2e-82  4.6-82 | 4 | a,b,c | 26 | 0.01 |  |
| **19277**  (aa1-154)  (aa1-154) | FRAS1-related extracellular matrix protein 2 (FREM2) | F1P4P1_CHICK  (aa2362-2515)  G1NQ83_MELGA  (aa604-757 | 100.0  99.4 | 4.5e-67  4.9e-67 | 4 | a,b,c | 18 | 0.01 |  |
| **24541**  (aa1-108)  (aa1-108) | FRAS1-related extracellular matrix protein 2 (FREM2) | G1NQ83_MELGA  (aa984-1101)  F1P4P1_CHICK  (aa2744-2861) | 88.1  89.0 | 3.6e-33  1.7e-32 | 3 | a,c | 9 | <0.01 |  |
| **25199**  (aa1-105)  (aa1-105) | FRAS1-related extracellular matrix protein 2 (FREM2); shares 1 peptide with 13681 | G1NQ83_MELGA  (aa455-559)  F1P4P1_CHICK  (aa2213-2317) | 95.2  97.1 | 3.0e-39  3.7e-39 | 4 | a,b | 11 | <0.01 |  |
| **264**  (aa1-1720)  (aa351-1711) | FRAS1-related extracellular matrix protein 2 (FREM2); shares 8 peptides with 6042 | F1P4P1_CHICK  (aa15-1733)  G1NQ88_MELGA  (aa2-1342) | 97.3  95.3 | 0e0  0e0 | 48 | a,b,c | 208 | 0.01 |  |
| **6042**  (aa1-418)  (aa1-418) | FRAS1-related extracellular matrix protein 2 (FREM2); shares 8 peptides with 264 | F1P4P1_CHICK  (aa366-825)  G1NQ88_MELGA  (aa4-443) | 89.1  84.5 | 4.3e-134  1.1e-83 | 1 | a | 1 | <0.01 |  |
| **13685**  (aa1-221)  (aa1-221) | Slit-like protein 2 protein (SLIT2) **^1,2,4^**; shares 1 peptide with 350 (Slit-3), 5459, 2305 | G1NIV1_MELGA  (aa359-579)  F1NR89_CHICK  (aa530-750) | 100.0  100.0 | 2.4e-92  2.7e-92 | 3 | a,b,c | 11 | 0.01 | **0.01** |
| **16772**  (aa1-181)  (aa1-181) | Similar to Slit-2 | Q90Z44_CHICK  (aa653-950)  G1NIV1_MELGA  (aa1052-1350) | 60.1  59.5 | 2.4e-39  5.7e-39 | 5 | a,b,c | 23 | <0.01 |  |
| **23949**  (aa1-114)  (aa1-114) | Slit-2 | Q90XG2_CHICK  (aa575-688)  G1NIV1_MELGA  (aa937-1050) | 100.0  100.0 | 3.8e-46  6.8e-46 | 3 | a,b,c | 23 | 0.01 |  |
| **13761**  (aa13-219)  (aa13-219) | Proto-oncogene tyrosine-protein kinase ROS (ROS1) **^1,2,4^** | G1NKX7_MELGA  (aa529-771)  ROS1_CHICK  (aa529-770) | 69.8  70.9 | 2.8e-49  5.0e-48 | 1 | a,b | 5 | <0.01 | **<0.01** |
| **13831**  (aa1-214)  (aa1-214) | Tyrosine-protein kinase receptor (ROS1); shares 1 peptide with 94 | F1NQL9_CHICK  (aa1309-1522)  G1NKX7_MELGA  (aa1310-1523) | 91.1  90.2 | 9.7e-89  6.4e-88 | 1 | a,b,c | 4 | <0.01 |  |
| **4325**  (aa4-529)  (aa4-529) | Proto-oncogene tyrosine-protein kinase ROS (ROS1); shares 2 peptides with 94 | ROS1_CHICK  (aa431-957)  G1NKX7_MELGA  (aa431-958) | 91.5  89.8 | 0e0  8.6e-211 | 1 | a,b | 4 | <0.01 |  |
| **94**  (aa1-2311)  (aa1-2311) | Tyrosine-protein kinase receptor (ROS1); shares 2 peptides with 4325 and 1 with 13831 | F1NQL9_CHICK  (aa1-2311)  G1NKX7_MELGA  (aa1-2312) | 100.0  94.2 | 0e0  0e0 | 7 | a,b,c | 18 | <0.01 |  |
| **13766**  (aa1-222)  (aa1-220) | EH domain-containing protein 4 (EHD4) **^2^**; shares 2 peptides with 14498 (EHD3) and 4 with 6467 | G1NFT2_MELGA  (aa11-230)  F1NV47_CHICK  (aa24-243) | 100.0  100.0 | 1.0e-93  1.0e-93 | 8 | a,b,c | 43 | 0.03 | **0.01** |
| **6467**  (aa20-405)  (aa20-405) | EH domain-containing protein 4 (EDH4), shares 1 peptide with 14498 and and 4 with 13766 | F1NV47_CHICK  (aa107-476)  G1NFT2_MELGA  (aa94-463) | 82.2  83.2 | 1-0e-75  2.1e-75 | 3 | a,b,c | 12 | <0.01 |  |
| **13801**  (aa1-94)  (aa1-86) | Mesothelin (MSLN) **^1,2; t^** | F1NZV7_CHICK  (aa458-551)  G1N1K4_MELGA  (aa514-599) | 84.0  84.9 | 3.5e-28  2.0e-25 | 3 | a,c | 3 | <0.01 | **<0.01** |
| **2480**  (aa201-625)  (aa201-625) | Mesothelin (MSLN); shares 7 peptides with 5817, and 3 with 18107 | F1NZV7_CHICK  (aa18-430)  G1N1K4_MELGA  (aa59-486) | 84.9  84.7 | 8.6e-154  2.2e-141 | 13 | a,b,c | 30 | <0.01 |  |
| **5817**  (aa43-434)  (aa43-434) | Mesothelin; shares 7 peptides with 2480 | G1N1K4_MELGA  (aa59-513)  F1NZV7_CHICK  (aa18-457) | 78.5  79.6 | 8.5e-122  5.5e-118 | 1 | a | 1 | <0.01 |  |
| **18107** | Uncharacterized/similar to mesothelin; domain: stereocilin_related; shares 3 peptides with 2480 |  |  |  | 2 | a,b,c | 8 | <0.01 |  |
| **13818**  (aa2-219) | Similar to hepatocyte growth factor-like protein (MST1) **^3^**; shares 2 peptides with 16425 | G1MVV6_MELGA  (aa108-318) | 62.4 | 3.0e-44 | 1 | c | 1 | <0.01 | **<0.01** |
| **16425**  (aa2-185)  (aa30-182) | Similar to hepatocyte growth factor-like/macrophage stimulating protein (MST1); shares 2 peptides with 13818 | G1MVV6_MELGA  (aa108-261)  Q90865_CHICK  (aa141-268) | 68.3  79.1 | 1.5e-39  4.5e-38 | 3 | a,b,c | 4 | <0.01 |  |
| **21844**  (aa4-94)  (aa4-94)  **18763** | Hepatocyte growth factor-like/macrophage stimulating protein (MST1); shares 2 peptides with 2601 | G1MVV6_MELGA  (aa514-603)  Q90865_CHICK  (aa519-608) | 95.6  92.3 | 9.3e-36  1.7e-34 | 2 | a,c | 6 | 0.01 |  |
| **2601**  (aa1-704)  (aa5-704) | Hepatocyte growth factor/macrophage stimulating protein 1(MST1); shares 2 peptides with 21844 and 18763 | Q90865_CHICK  (aa1-704)  G3USW9_MELGA  (aa1-699) | 100.0  93.7 | 0e0  0e0 | 11 | a,b,c | 23 | <0.01 |  |
| **13827**  (aa1-218)  (aa57-218) | Transmembrane emp24 domain-containing protein 7 (TMED7) **^2^** | Q5ZLF6_CHICK  (aa1-219)  G1N9P0_MELGA  (aa4-165) | 96.3  98.8 | 3.0e-97  2.0e-71 | 4 | a,b,c | 9 | <0.01 | **<0.01** |
| **13834**  (aa45-218)  (aa48-218) | UMP-CMP kinase (CMPK) | KCY_CHICK  (aa23-196)  G1NEB5_MELGA  (aa2-172) | 97.7  99.4 | 7.8e-70  9.3e-70 | 4 | a,b,c | 6 | <0.01 | **<0.01** |
| **13864**  (aa4-209)  (aa4-209) | Similar to Collagen alpha-1(VII) chain (COL7A1) **^1,2^**; domains: VWA, FN3; shares 1 peptide with 2456 | F1NBA6_CHICK  (aa116-321)  G1N5G9_MELGA  (aa116-322) | 87.4  82.7 | 1.3e-64  1.3e-64 | 2 | a,b,c | 6 | <0.01 | **<0.01** |
| **2456**  (aa1-682)  (aa1-680) | Similar to COL7A1; shares 6 peptides with 4678, and 1 with 13864 | F1NBA6_CHICK  (aa1-1233)  G1N5G9_MELGA  (aa1-1269) | 50.0  48.5 | 5.5e-79  8.8e-75 | 11 | a,b,c | 35 | <0.01 |  |
| **4678**  (aa17-506)  (aa17-467) | Uncharacterized (similar to COL7A1); shares 6 peptides with 2456 | G1N5G9_MELGA  (aa412-1268)  F1NBA6_CHICK  (aa411-1214) | 48.6  51.4 | 2.0e-76  2.7e-75 | 3 | a,b,c | 5 | <0.01 |  |
| **13883**  (aa1-218)  (aa1-218) | 14-3-3 protein gamma (YWHAG) **^3^**; shares 3 peptides with 20781 and other 14-3-3 proteins | F1NMY1_CHICK  (aa1-218)  G1MX53_MELGA  (aa4-221) | 100.0  100.0 | 5.2e-89  5.3e-89 | 2 | a,c | 4 | <0.01 | **<0.01** |
| **13891**  (aa1-218) | 14-3-3 protein epsilon (YWHAE) **^1,2^**; shares 2 peptides with other 14-3-3 proteins | F1P2P9_CHICK  (aa2-219) | 99.5 | 2.2e-95 | 14 | a,b,c | 56 | 0.01 | **0.01** |
| **139**  (aa15-2032)  (aa111-2034) | Similar to laminin subunit alpha-5 (LAMA5) **^1,2^**; shares 27 peptides with 458, 2 with 6755 and 1 18709 | G1N210_MELGA  (aa48-3598)  F1NZZ2_CHICK  (aa1-3451) | 51.6  51.3 | 2.9e-194  8.3e-194 | 40 | a,b,c | 160 | <0.01 | **<0.01** |
| **458**  (aa37-1376)  (aa43-1378) | Similar to laminin subunit alpha-5 (LAMA5); shares 27 peptides with 139 | G1N210_MELGA  (aa1993-3598)  F1NZZ2_CHICK  (aa1636-3451) | 73.2  67.8 | 2.6e-201  7.8e-201 | 2 | a,b,c | 13 | <0.01 |  |
| **6755**  (aa20-388)  (aa20-388) | Similar to laminin subunit alpha-5 (LAMA5); shares 2 peptides with 139 | F1NZZ2_CHICK  (aa1048-1602)  G1N210_MELGA  (aa1192-1758) | 63.8  62.6 | 6.3e-78  2.1e74 | 3 | a,b,c | 21 | <0.01 |  |
| **7314**  (aa5-358)  (aa5-358) | Similar to laminin subunit alpha-5 (LAMA5) | G1N210_MELGA  (aa629-1086)  F1NZZ2_CHICK  (aa485-942) | 71.5  70.8 | 1.2e-53  2.5e-53 | 2 | a,c | 5 | <0.01 |  |
| **13912**  (aa2-135)  (aa1-210) | Calcium-activated nucleotidase 1 (CANT1) **^1,2^**; domain: apyrase | F1NLP1_CHICK  (aa141-274)  G1N2S5_MELGA  (aa48-442) | 97.8  90.5 | 2.9e-57  2.3e-49 | 5 | a,b,c | 15 | 0.01 | **0.01** |
| **14001**  (aa11-175)  (aa54-175) | Chondroitin sulfate synthase 1 (CHSY1) | G1NAX3_MELGA  (aa2-166)  R4GL94_CHICK  (aa1-122) | 100.0  100.0 | 5.6e-75  4.8e-53 | 3 | b,c | 3 | <0.01 | **<0.01** |
| **4361**  (aa1-530)  (aa1-530) | Chondroitin sulfate synthase 1 (CHSY1) | R4GL94_CHICK  (aa123-652)  G1NAX3_MELGA  (aa167-696) | 99.6  99.4 | 0e0  0e0 | 12 | a,b,c | 46 | <0.01 |  |
| **14051**  (aa1-214)  (aa30-214) | Tetraspanin (TSPAN6) **^1,2,4^** | E1C857_CHICK  (aa40-241)  G1MYM6_MELGA  (aa2-174) | 92.1  89.7 | 1.2e-51  1.3e-38 | 4 | a,b,c | 17 | <0.01 | **0.01** |
| **14057**  (aa1-216)  (aa1-216) | Ras-related protein Rab-5C (RAB5C) **^1,2^** | G1MWE3_MELGA  (aa1-216)  RAB5C_CHICK  (aa1-216) | 100.0  100.0 | 1.2e-86  1.2e-86 | 5 | a,b,c | 10 | <0.01 | **<0.01** |
| **14075**  (aa1-212) | [Protein ADP-ribosylarginine]  Hydrolase (ADPRH) **^1,2^** | G1NNQ0_MELGA  (aa143-354) | 91.5 | 9.1e-88 | 5 | a,b,c | 24 | 0.04 | **0.08** |
| **16934**  (aa1-150)  (aa29-135) | [Protein ADP-ribosylarginine] hydrolase (ADPRH) | Q5ZMJ1_CHICK  (aa1-150)  G1NNQ0_MELGA  (aa1-107) | 92.0  93.5 | 3.8e-60  9.8e-41 | 4 | a,b,c | 41 | 0.12 |  |
| **141**  (aa21-2019) | Fibronectin (FN1) **^1,2^**; shares 80 peptides with A0A060PIY8, 68 with 76 and 34 with 441 | F1NJT4_CHICK  (aa184-2314) | 82.8 | 0e0 | 2 | a,b | 3 | <0.01 | **0.13** |
| **A0A060PIY8**  (aa1-2484)  (aa1179-2383)  **76; 441** | Fibronectin 1; shares 80 peptides with 141 | F1NJT3_CHICK  (aa1-2484)  G1MWJ4_MELGA  (aa52-1256) | 99.0  98.7 | 0e0  0e0 | 116 | a,b,c | 1598 | 0.21 |  |
| **14104**  (aa1-172)  (aa1-172) | Receptor protein tyrosine phosphatase LAR (PTPRF) **^1,2^**; shares 1 peptide with 1577 | G3UTB7_MELGA  (aa523-694)  A3FB57_CHICK  (aa523-694) | 97.7  97.7 | 2.8e-68  4.5e-68 | 4 | a,b,c | 15 | 0.01 | **0.01** |
| **4319**  (aa1-480)  (aa1-480) | Receptor-type tyrosine-protein phosphatase F (PTPRF); shares 11 peptides with 6809 | G3UTB7_MELGA  (aa46-527)  F1N897_CHICK  (aa129-610) | 99.2  99.2 | 2.9e-199  3.1e-199 | 17 | a,b,c | 148 | 0.02 |  |
| **6084**  (aa1-423)  (aa1-423) | Receptor-type tyrosine-protein phosphatase F (PTPRF); shares 3 peptides with 7499 | G3UTB7_MELGA  (aa743-1148)  F1N897_CHICK  (aa817-1222) | 93.6  93.4 | 4.8e-121  1.4e-120 | 20 | a,b,c | 79 | 0.01 |  |
| **6809**  (aa1-390)  (aa1-390) | Similar to receptor-type tyrosine-protein phosphatase F (PTPRF); shares 11 peptides with 4319 | G3UTB7_MELGA  (aa46-521)  F1N897_CHICK  (aa129-604) | 55.3  55.3 | 2.7e-55  3.5e-55 | 1 | a,b | 2 | <0.01 |  |
| **7499**  (aa1-327)  (aa1-327) | Receptor-type tyrosine-protein phosphatase F (PTPRF); shares 3 peptides with 6084 | F1N897_CHICK  (aa1122-1448)  G1NBY3_MELGA  (aa1020-1344) | 99.4  87.6 | 2.7e-141  7.2e-121 | 2 | a,b | 4 | <0.01 |  |
| **22668**  (aa35-125)  (aa35-125)  **14111** | Exostosin-2 (EXT2) **^1,2^** | F1NR24_CHICK  (aa556-646)  G1NDI8_MELGA  (aa469-559) | 100.0  100.0 | 7.3e-41  8.5e-41 | 3 | a,c | 4 | <0.01 | **0.01** |
| **3947**  (aa1-567)  (aa1-567) | Exostosin-2 (EXT2); G1NDI8_MELGA (aa2-719; 78.6%; 0e0) | Q5F3S5_CHICK  (aa1-567)  G3UPY9_MELGA  (aa1-567) | 100.0  99.3 | 0e0  0e0 | 22 | a,b,c | 65 | 0.01 |  |
| **14128**  (aa1-215) | Beta-hexosaminidase subunit beta (HEXB **^1,2^**; shares 2 peptides with 8970 | F1NTQ2_CHICK  (aa262-476) | 94.9 | 1.9e-92 | 2 | a,b,c | 7 | 0.01 | **<0.01** |
| **8970**  (aa1-290)  (aa37-312) | Similar to beta-hexosaminidase subunit beta (HEXB); shares 2 peptides with 14128 | F1NTQ2_CHICK  (aa5-507)  G1MRV8_MELGA  (aa138-413) | 52.9  54.3 | 7.2e-81  2.2e-61 | 6 | a,b,c | 13 | <0.01 |  |
| **14179**  (aa1-214) | Sphingomyelin phosphodiesterase-like protein (SMPD1) **^1^** | R4GHJ3_CHICK  (aa193-440) | 83.5 | 2.2e-81 | 7 | a,b,c | 15 | 0.01 | **0.01** |
| **14227**  (aa1-139)  (aa1-139) | Cystatin (P81061; CYT_COTJA) **^1,2; t^** | CYT_CHICK  (aa1-139)  G1N522_MELGA  (aa1-139) | 88.5  87.8 | 7.7e-52  3.1e-51 | 9 | a,b,c | 88 | 0.15 | **0.11** |
| **14241**  (aa1-141)  (aa1-141) | Pentraxin (PTX3/PPTX) **^1,2^** | G1NDX8_MELGA  (aa18-158)  Q5UMH8_CHICK  (aa18-158) | 93.6  94.3 | 8.1e-46  2.0e-45 | 10 | a,b,c | 119 | 0.13 | **0.21** |
| **14979**  (aa1-203)  (aa1-203) | Pentraxin (PTX3/PPTX) | G1NDX8_MELGA  (aa231-433)  Q5UMH8_CHICK  (aa231-433) | 93.1  93.1 | 5.1e-86  7.5e-85 | 7 | a,b,c | 160 | 0.27 |  |
| **14246**  (aa1-213)  (aa1-213)  **4035** | Similar to polypeptide N-acetylgalactosaminyltransferase 1 (GALNT1) | G1N4F1_MELGA  (aa288-559)  Q5ZJL1_CHICK  (aa288-559) | 77.9  77.9 | 5.9e-81  5.9e-81 | 3 | a,b,c | 5 | <0.01 | **<0.01** |
| **14285**  (aa2-198  (aa2-198) | Similar to type II alpha-keratin IIA (KRT5); shares peptide CON | Q6PVZ5_CHICK  (aa111-412)  G1NEY4_MELGA  (aa111-411) | 44.7  44.9 | 3.0e-34  1.4e-23 | 4 | a,c | 6 | <0.01 | **<0.01** |
| **14304**  (aa1-189) | Matrilin-3 (MATN3) **^2^** | MATN3_CHICK  (aa46-234) | 95.8 | 2.5e-67 | 4 | a,b | 5 | <0.01 | **<0.01** |
| **14319**  (aa1-212)  (aa1-212) | Calpain-5 (CAPN5) **^1,2^** | G1NQT4_MELGA  (aa438-641)  E1C292_CHICK  (aa438-641) | 94.3  95.8 | 3.6e-88  4.3e-86 | 4 | a,b,c | 12 | <0.01 | **<0.01** |
| **15925**  (aa1-191)  (aa1-191) | Calpain-5 (CAPN5) | G1NQT4_MELGA  (aa239-429)  E1C292_CHICK  (aa239-429) | 100.0  99.5 | 2.5e-90  7.2e-90 | 3 | a,b,c | 7 | <0.01 |  |
| **14405**  (aa67-211)  (aa67-211) | Ubiquitin-conjugating enzyme E2 L3 (UBE2L3) | F1NIZ6_CHICK  (aa4-148)  G3UQU8_MELGA  (aa4-149) | 100.0  99.3 | 2.4e-62  1.7e-61 | 2 | a,b | 4 | <0.01 | **<0.01** |
| **1444**  (aa1-912)  (aa25-912) | Cadherin-2 (CDH2) **^1,2^** | CADH2_CHICK  (aa1-912)  G1NDC6_MELGA  (aa1-888) | 100.0  99.7 | 0e0  0e0 | 8 | a,b,c | 54 | 0.01 | **0.01** |
| **14448**  (aa1-208)  (aa1-208) | Programmed cell death 6-interacting protein (PDCD6IP) **^1,2^**; shares 1 peptide with 8791 | Q5ZJ70_CHICK  (aa358-554)  G1NH54_MELGA  (aa356-554) | 92.8  91.9 | 1.3e-51  1.0e-50 | 9 | a,b,c | 67 | 0.02 | **0.02** |
| **18712**  (aa1-160)  (aa1-160) | Programmed cell death 6-interacting protein (PDCD6IP) | Q5ZJ70_CHICK  (aa552-711)  G1NH54_MELGA  (aa552-714) | 99.4  75.5 | 4.2e-49  5.6e-33 | 10 | a,b,c | 20 | <0.01 |  |
| **8791**  (aa1-324)  (aa1-324) | Programmed cell death 6-interacting protein; shares 1 peptide with 14448 | G1NH54_MELGA  (aa73-396)  Q5ZJ70_CHICK  (aa70-398) | 99.7  98.2 | 7.5e-132  1.7e-130 | 14 | a,b,c | 115 | 0.02 |  |
| **14498**  **(**aa6-209)  (aa6-209) | EH domain-containing protein (EHD3) **^2^**; shares 2 peptides with 13766 (EHD4) and 1 with 6467 (EHD4) | G1N1U5_MELGA  (aa93-285)  F1NGM0_CHICK  (aa168-360) | 94.1  94.1 | 3.6e-53  4.3e-53 | 12 | a,b,c | 105 | 0.02 | **0.01** |
| **19626**  (aa1-151)  (aa1-151) | EH domain-containing protein 3 (EHD3) | G1N1U5_MELGA  (aa310-460)  F1NGM0_CHICK  (aa385-535) | 100.0  100.0 | 7.0e-60  8.3e-60 | 4 | a,b,c | 12 | <0.01 |  |
| **29019**  (aa1-64)  (aa1-65) | EH domain-containing protein 3 (EHD3), | G1N1U5_MELGA  (aa2-66)  Q5ZM17_CHICK  (aa77-141) | 93.8  93.8 | 1.7e-22  2.0e-22 | 3 | a,b,c | 19 | 0.01 |  |
| **28794**  (aa1-76) | EH domain-containing protein 3 (EHD3) | Q5ZM17_CHICK  (aa1-76) | 98.7 | 9.0e-30 | 3 | a,b,c | 9 | <0.01 |  |
| **14502**  (aa1-209)  (aa1-209) | Malectin (MLEC) **^2^** | G1N8W9_MELGA  (aa6-214)  F1N872_CHICK  (aa73-281) | 100.0  100.0 | 1.7e-83  2.4e-83 | 5 | a,b,c | 16 | <0.01 | **<0.01** |
| **1452**  (aa59-362)  (aa59-361) | Heme oxygenase 1 (HMOX1) | F1NQF4_CHICK  (aa12-296)  G1NJD4_MELGA  (aa14-297) | 90.1  90.4 | 1.1e-92  5.0e-92 | 3 | b,c | 3 | <0.01 | **<0.01** |
| **14523**  (aa11-141)  (aa11-141) | Ubiquitin-conjugating enzyme E2 N (UBE2N) | Q5F405_CHICK  (aa9-139)  G1NFP6_MELGA  (aa9-139) | 99.2  98.5 | 4.6e-54  5.9e-54 | 2 | a,b,c | 3 | <0.01 | **<0.01** |
| **14556**  (aa1-208)  (aa1-208) | Ribosomal protein S3a **^1,2^** | F2Z4K7_CHICK  (aa57-264)  G1MYH2_MELGA  (aa57-264) | 100.0  99.5 | 4.4e-85  2.5e-84 | 3 | a,b | 3 | <0.01 | **<0.01** |
| **14603**  (aa1-208)  (aa1-208) | Ras-dva small GTPase (RAP2B) | F1P3K5_CHICK  (aa1-208)  G1NR23_MELGA  (aa1-208) | 97.6  97.6 | 1.8e-69  3.6e-69 | 4 | a,b,c | 9 | <0.01 | **<0.01** |
| **14613**  (aa1-208)  (aa18-208) | Ras-related protein Rab-35 (RAB35) **^1^** | Q5F4C5_CHICK  (aa1-201)  G1N9R6_MELGA  (aa1-184) | 96.6  96.3 | 2.3e-69  1.5e-62 | 3 | a,b,c | 18 | 0.01 | **0.01** |
| **14618**  (aa65-208)  (aa1-208) | Similar to keratin (KRT7) **^1,2^**; shares 2 peptides with CON | H9KZP5_CHICK  (aa38-222)  G1NF62_MELGA  (aa250-514) | 73.5  62.6 | 2.4e-18  2.5e-18 | 3 | a,b,c | 34 | 0.01 | **<0.01** |
| **15221**  (aa1-200)  (aa1-200) | Keratin, type II cytoskeletal cochleal (KRT7; K2CO_CHICK aa1-201); shares 4 peptides with CON | G1NF62_MELGA  (aa23-223)  H9KZP6_CHICK  (aa1-201) | 96.5  96.5 | 3.4e-62  4.1e-62 | 6 | a,b,c | 11 | <0.01 |  |
| **14651**  (aa3-207)  (aa3-207) | Integral membrane protein 2C (ITM2C) | G1MYA9_MELGA  (aa1-205)  F1NRF2_CHICK  (aa29-234) | 98.5  97.1 | 5.0e-88  2.3e-85 | 6 | a,b,c | 10 | <0.01 | **<0.01** |
| **14673**  (aa1-203)  (aa1-207) | FAM20C kinase/DMP4 **^1,2,4^** | G1MSD1_MELGA  (aa73-293)  E1C4X0_CHICK  (aa283-509) | 89.2  90.7 | 1.2e-56  2.3e-56 | 14 | a,b,c | 95 | 0.10 | **0.12** |
| **21781**  (aa1-129) | Similar to FAM20C kinase/DMP4 | E1C4X0_CHICK  (aa129-288) | 76.9 | 3.8e-33 | 9 | a,b,c | 92 | 0.14 |  |
| **14686**  (aa16-151)  (aa17-151) | Putative cation-transporting ATPase 13A5 (ATP13A5) **^1,2^** | E1C3D9_CHICK  (aa587-722)  G1N5U9_MELGA  (aa575-709) | 97.8  97.0 | 3.3e-48  1.8e-47 | 3 | a,b | 4 | <0.01 | **<0.01** |
| **1473**  (aa1-863)  (aa1-863) | Cadherin, EGF LAG seven-pass G-type receptor 1 (CELSR1) | H9L033_CHICK  (aa1-894)  G1N6Q6_MELGA  (aa15-908) | 87.6  87.6 | 1.5e-198  3.9e-198 | 1 | a,b | 3 | <0.01 | **<0.01** |
| **790**  (aa1-1140)  (aa1-1140) | Similar to cadherin, EGF LAG seven-pass G-type receptor 1 (CELSR1) | F1N812_CHICK  (aa53-1145)  G1N6Q6_MELGA  (aa15-1121) | 61.2  67.3 | 0e0  3.9e-169 | 2 | b | 3 | <0.01 |  |
| **14737**  (aa48-206)  (aa7-206) | Similar to putative phospholipase B-like 2 (PLBD2) **^1,2^** | G1NCL1_MELGA  (aa29-243)  F1P0Z3_CHICK  (aa57-314) | 71.2  60.4 | 2.4e-45  4.2e-44 | 3 | a,b,c | 5 | <0.01 | **<0.01** |
| **14782**  (aa15-205)  (aa4-205) | Creatine kinase B-type (CKB) **^1,2,4^**; shares 2 peptides with 6545 | F1NZ30_CHICK  (aa17-218)  G5E7M6_MELGA  (aa1-213) | 90.6  93.0 | 4.0e-58  4.2e-58 | 3 | b,c | 5 | <0.01 | **0.01** |
| **6545**  (aa17-402)  (aa1-402) | Creatine kinase B (CKB); shares 2 peptides with 14782 | G5E7M6_MELGA  (aa12-376)  KCRB_CHICK  (aa1-381) | 83.2  83.3 | 6.5e-94  6.6e-94 | 8 | a,b,c | 20 | 0.01 |  |
| **14790**  (aa38-205)  (aa1-205) | Triosephosphate isomerase (TPI1) **^1,2^** | G1NMV8_MELGA  (aa45-212)  TPIS_CHICK  (aa1-248) | 99.4  82.3 | 5.0e-67  6.0e-67 | 7 | a,b,c | 13 | <0.01 | **<0.01** |
| **14813**  (aa1-204)  (aa1-204) | Sortilin-related receptor (SORL1) **^1,2^**; SORL_CHICK aa721-924; 99.0%; 1.9e-90 | E1BUD4_CHICK  (aa806-1009)  G3UQY4_MELGA  (aa719-922) | 99.5  99.5 | 6.2e-91  9.3e-90 | 4 | a,b,c | 14 | <0.01 | **<0.01** |
| **3860**  (aa25-577)  (aa26-577) | Similar to sortilin-related receptor (SORL1) | G1MT79_MELGA  (aa1-723)  SORL_CHICK  (aa3-720) | 73.4  73.9 | 7.7e-165  2.9e-104 | 14 | a,b,c | 53 | <0.01 |  |
| **3936**  (aa1-570)  (aa1-570) | Sortilin-related receptor (SORL1) | G1MT79_MELGA  (aa1491-2102)  E1BUD4_CHICK  (aa1572-2183) | 91.0  90.7 | 0e0  0e0 | 4 | a,c | 11 | <0.01 |  |
| **14817**  (aa1-165)  (aa1-165) | Cilia- and flagella-associated protein 20 (CFAP20) **^2^** | G1MX21_MELGA  (aa14-248)  CFA20_CHICK  (aa29-193) | 99.4  99.4 | 1.3e-73  1.4e-73 | 6 | a,b,c | 11 | <0.01 | **<0.01** |
| **14839**  (aa1-205)  (aa1-205) | Dipeptidyl-peptidase 1 (CTSC) **^1,2^** | G1NQP8_MELGA  (aa187-435)  F1NWG2_CHICK  (aa215-463) | 80.7  80.7 | 3.5e-80  6.3e-80 | 11 | a,b,c | 37 | 0.01 | **0.01** |
| **14877**  (aa1-204)  (aa1-204) | Rho GDP-dissociation inhibitor 1 (ARHGDIA) **^1,2^** | F1P3P3_CHICK  (aa1-204)  G1N1C7_MELGA  (aa1-204) | 100.0  100.0 | 1.1e-85  1.1e-85 | 4 | a,b,c | 10 | <0.01 | **<0.01** |
| **1534**  (aa12-888)  (aa197-888)  **1475** | Alpha-actinin-1 (ACTN1) **^1,2^** | R9PXN5_CHICK  (aa2-878) G3UR37_MELGA  (aa1-692) | 99.9  99.7 | 0e0  0e0 | 9 | a,b | 9 | <0.01 | **<0.01** |
| **14911**  (aa6-183)  (aa4-183) | Ovostatin (LOC100545094/OVOST) **^1; i^**; shares 3 peptides with 389 and 1 with 5368a; OVOS_CHICK aa951-1131; 84.7%; 5.9e-64 | G1NMI3_MELGA  (aa951-1131)  F1NU63_CHICK  (aa950-1130) | 86.8  85.2 | 8.8e-66  1.4e-64 | 3 | a,b,c | 69 | 0.03 | **0.08** |
| **5368a**  (aa1-309)  (aa1-312) | Similar to ovostatin; shares 5 peptides with 389 and 1 with 14911 | G1NMI3_MELGA  (aa933-1323)  (OVOS_CHICK)  (aa933-1326) | 73.1  71.6 | 5.0e-86  3.2e-83 | 2 | a,b,c | 31 | 0.04 |  |
| **18662**  (aa1-160)  (aa1-80) | Similar to ovostatin; shares 4 peptides with 455b and 3 with 389; OVOS_CHICK aa624-914; 50.2%; 2.1e-30 | F1NU63_CHICK  (aa24-913)  G1NMI4_MELGA  (aa42-121) | 50.3  87.5 | 9.8e-31  3.2e-30 | 4 | a,b,c | 19 | 0.03 |  |
| **389**  (aa1-1454)  (aa1-1454) | Ovostatin; shares 5 peptides with 5368a, 3 with 14911, 9 with 455b, and 3 with 18662 | OVOS_CHICK  (aa20-1473)  G3URW8_MELGA  (aa20-1475) | 100.0  91.3 | 0e0  0e0 | 20 | a,b,c | 364 | 0.05 |  |
| **455b**  (aa826-1326) | Similar to ovostatin; shares 9 peptides with 389 and 4 with 18662 (OVOS_CHICK; aa16-727; 59.1%; 4.7e-108) | F1NU63_CHICK  (aa16-727)  G1NMI3_MELGA  (aa16-728) | 59.8  59.1 | 1.3e-110  2.5e-110 | 17 | a,b,c | 261 | 0.26 |  |
| **1506a**  (aa1-420)  (aa1-420) | Alpha-1-antiproteinase (SERPINA4) **^2,3^**; (aa456-895 is Serpin A10, (F1NPN3_CHICK; aa1-439) | E1BS56_CHICK  (aa1-421)  G1NKH5_MELGA  (aa2-421) | 87.4  85.2 | 6.1e-161  2.1e-157 | 17 | a,b,c | 57 | 0.01 | **0.01** |
| **15085**  (aa34-202)  (aa34-202) | Papilin (PAPLN) **^1,2^**; shares 2 peptides with 21498 | F1P2U7_CHICK  (aa110-278)  G1NGM4_MELGA  (aa117-285) | 99.4  98.2 | 8.0e-71  4.4e-285 | 10 | a,b,c | 183 | 0.12 | **0.09** |
| **19866**  (aa15-149)  (aa26-149) | Papilin; shares 10 peptides with 20205 | F1P2U7_CHICK  (aa1024-1158)  G1NGM4_MELGA  (aa1068-1191) | 91.9  96.8 | 2.2e-48  2.2e-47 | 11 | a,b,c | 158 | 0.13 |  |
| **20205**  (aa1-146)  (aa1-88) | Papilin; shares 10 peptides with 19866 | F1P2U7_CHICK  (aa1035-1275)  G1NGM4_MELGA  (aa1068-1155) | 58.5  96.6 | 2.8e-31  1.9e-30 | 1 | b | 3 | <0.01 |  |
| **21498**  (aa1-119)  (aa1-119) | Similar to papilin; shares 2 peptides with 15085 | F1P2U7_CHICK  (aa75-278)  G1NGM4_MELGA  (aa82-285) | 57.8  55.9 | 6.4e-34  4.5e-33 | 2 | a,b,c | 35 | 0.02 |  |
| **15110**  (aa1-201)  (aa1-201) | Cathepsin Z (CTSZ) **^1,2,4^** | E1C4M3_CHICK  (aa105-305)  G1N9B0_MELGA  (aa31-231) | 99.0  98.5 | 2.7e-94  4.5e-94 | 5 | a,b,c | 19 | 0.01 | **0.03** |
| **15120**  (aa54-187)  (aa56-187) | Myosin light chain (MYL1/MYL3) | F1NZG0_CHICK  (aa7-140)  Q6W5H1_MELGA  (aa52-183) | 97.8  97.0 | 3.0e-47  2.5e-46 | 4 | a,b | 5 | 0.02 | **0.02** |
| **15147**  (aa1-172)  (aa1-172) | Similar to 5'-nucleotidase (NT5E) **^1^** | G1NLS6_MELGA  (aa176-434)  F1NZT4_CHICK  (aa255-513) | 62.9  62.5 | 4.5e-44  2.0e-43 | 3 | a,b,c | 5 | <0.01 | **<0.01** |
| **15205**  (aa1-169)  (aa1-200) | Calreticulin (CALR) **^1,2^** | H9H0E9_MELGA  (aa2-170)  Q6EE32_CHICK  (aa68-353) | 100.0  69.9 | 1.5e-71  3.0e-71 | 11 | a,b,c | 40 | 0.01 | **0.01** |
| **16021**  (aa1-189)  (aa1-168)  **15232** | Polypeptide N-acetylgalactosaminyltransferase 3 (GALNT3) **^1,2^** | E1C241_CHICK  (aa230-419)  G1NE91_MELGA  (aa229-396) | 90.5  98.2 | 2.2e-76  2.4e-75 | 1 | a,b,c | 5 | <0.01 | **<0.01** |
| **28817**  (aa1-71)  (aa1-71) | Polypeptide N-acetylgalactosaminyltransferase 3 (GALNT3); shares peptide with 18456 (GALNT6) | G1NE91_MELGA  (aa103-173)  E1C241_CHICK  (aa104-174) | 97.2  95.8 | 2.4e-29  3.0e-29 | 3 | a,b,c | 4 | <0.01 |  |
| **1525**  (aa1-727)  (aa1-727) | HSP90-alpha (HSP90AA1) **^1,2^**, C7G492_COTJA; shares 3 peptides with 2075 (HSP90-beta) | G1NKX1_MELGA  (aa1-727)  HS90A_CHICK  (aa1-727) | 99.7  99.6 | 4.5e-194  1.4e-193 | 18 | a,b,c | 81 | 0.01 | **0.01** |
| **15258b**  (aa68-199)  (aa68-199) | 16 kDa beta-galactoside-binding lectin (LOC100544806) | G1NB72_MELGA  (aa3-134)  LEG6_CHICK  (aa3-134) | 96.2  95.5 | 2.3e-57  4.4e-56 | 4 | a,b,c | 9 | <0.01 | **<0.01** |
| **1526**  (aa28-892)  (aa8-892) | Similar to collagen alpha-1(XIV) chain (COL14A1); shares 3 peptides with 197, and 1 with 9826 | G1NIW5_MELGA  (aa142-1225)  COEA1_CHICK  (aa142-1226) | 76.6  76.4 | 5.9e-147  8.1e-146 | 1 | b | 1 | <0.01 | **<0.01** |
| **197**  (aa1-1888)  (aa1-1888) | Collagen alpha-1(XIV) chain (COL14A1); shares 3 peptides with 1526, and 2 with 9826 | COEA1_CHICK  (aa1-1888)  G1NIW6_MELGA  (aa1-1887) | 100.0  97.1 | 0e0  0e0 | 12 | a,b,c | 15 | <0.01 |  |
| **9826**  (aa1-296)  (aa1-296) | Collagen alpha-1(XIV) chain (COL14A1); shares 2 peptides with 197 | G1NIW3_MELGA  (aa674-1016)  COEA1_CHICK  (aa669-1009) | 84.3  83.9 | 1.4e-54  1.5e-50 | 1 | b | 1 | <0.01 |  |
| **1527**  (aa1-348)  (aa1-882) | Uncharacterized/similar to Mucin **^1,2^**; domains: VWD, VWC, cysteine_knot; shares 7 peptides with 6950 | G1N931_MELGA  (aa329-966)  E1C037_CHICK  (aa8-1194) | 48.4  29.7 | 9.2e-59  4.4e-32 | 4 | a,b,c | 36 | 0.01 | **0.10** |
| **5306**  (aa1-450)  (aa259-462) | Uncharacterized/similar to mucin; domains: WxxW (3x) | E1C037_CHICK  (aa1772-2367)  G1N931_MELGA  (aa1-203) | 56.2  81.0 | 2.6e-60  2.6e-39 | 6 | a,b,c | 71 | 0.38 |  |
| **6950**  (aa1-384) | Uncharacterized/similar to mucin; shares 7 peptides with 1527 | G1N931_MELGA  (aa329-852) | 64.7 | 3.3e-114 | 12 | a,b,c | 260 | 0.18 |  |
| **15278**  (aa1-199)  (aa1-199) | Cathepsin D (CTSD) **^1,2^**; shares 7 peptides with 3793b | CATD_CHICK  (aa24-222)  G1N8P0_MELGA  (aa31-229) | 100.0  99.0 | 1.2e-85  5.7e-85 | 6 | a,b,c | 52 | 0.06 | **0.18** |
| **3793b**  (aa222-581)  (aa235-581) | Cathepsin D; shares 7 peptides with 15278 | CATD_CHICK  (aa4-396)  G1N8P0_MELGA  (aa31-403) | 89.3  90.9 | 1.1e-90  7.6e-90 | 18 | a,b,c | 150 | 0.11 |  |
| **15298**  (aa1-199)  (aa1-199) | Similar to phosphatidylinositol 3,4,5-trisphosphate 3-phosphatase (TPTE2) | F1BUX1_CHICK  (aa154-478)  G1NQ81_MELGA  (aa154-479) | 60.3  58.9 | 6.3e-36  4.2e-35 | 2 | a,b | 3 | <0.01 | **<0.01** |
| **15308**  (aa1-199)  (aa9-199) | Similar to palmitoyl-protein thioesterase 1 (PPT1) **^1,2^** | F1NYK1_CHICK  (aa45-300)  G1N1R1_MELGA  (aa13-260) | 73.8  71.0 | 1.9e-66  5.8e-61 | 4 | a,b,c | 19 | 0.01 | **0.01** |
| **15338**  (aa1-193) | Guanine deaminase (GDA) | F1NJD6_CHICK  (aa1-193) | 95.3 | 6.7e-84 | 2 | a,b,c | 2 | <0.01 | **<0.01** |
| **24251**  (aa1-112)  (aa1-112) | Guanine deaminase (GDA) | F1NJD6_CHICK  (aa339-450)  G3USJ6_MELGA  (aa144-255) | 95.5  94.6 | 2.7e-42  4.6e-41 | 2 | a,c | 2 | <0.01 |  |
| **15394**  (aa1-151)  (aa1-151) | Similar to alpha-1-acid glycoprotein (LOC100541166/ogchi) **^1,2^** | G1MWU3_MELGA  (aa1-180)  Q8JIG5_CHICK  (aa1-180) | 76.7  76.7 | 6.4e-31  1.1e-30 | 4 | a,b,c | 31 | 0.01 | **0.02** |
| **15505**  (aa1-196)  (aa1-196) | Out at first protein homolog (OAF) **^1,2^** | OAF_CHICK  (aa75-270)  G1MTX0_MELGA  (aa84-278) | 100.0  100.0 | 1.2e-87  2.7e-87 | 9 | a,b,c | 56 | 0.03 | **0.03** |
| **1542**  (aa1-887)  (aa150-817) | Cadherin-1 (CDH1) **^1,2^**; shares 1 peptide with 3257 | CADH1_CHICK  (aa1-887)  G1N7A9_MELGA  (aa1-669) | 100.0  85.8 | 0e0  0e0 | 2 | a | 6 | <0.01 | **0.01** |
| **3257**  (aa1-629)  (aa1-559) | Cadherin-1; shares 1 peptide with 1542 | CADH1_CHICK  (aa288-887)  G1N7A9_MELGA  (aa139-669) | 89.8  84.1 | 2.2e-171  1.8e-137 | 4 | a,b,c | 33 | 0.01 |  |
| **15509**  (aa76-188)  (aa76-188) | Uncharacterized/similar to ovostatin-like (OVSTL) **^1,2^**; shares 1 peptide with 455a | F1NEW8_CHICK  (aa23-155)  G1NK51_MELGA  (aa30-142) | 93.8  92.0 | 1.5e-43  1.2e-42 | 5 | a,b,c | 82 | 0.11 | **0.34** |
| **455a**  (aa78-826)  (aa78-826) | Uncharacterized/similar to ovostatin-like (OVSTL); shares 1 peptide with 15509 and 8 with 9640 | F1NEW8_CHICK  (aa364-1441)  G1NK51_MELGA  (aa371-1439) | 60.5  57.1 | 3.0e-149  3.0e-146 | 42 | a,b,c | 835 | 0.26 |  |
| **25086**  (aa1-106)  (aa1-105) | Uncharacterized/Ovostatin-like (OVSTL) | F1NEW8_CHICK  (aa309-414)  G1NK51_MELGA  (aa316-420) | 91.5  91.4 | 8.0e-39  9.2e-38 | 7 | a,b,c | 102 | 0.49 |  |
| **9640**  (aa1-302)  (aa1-302) | Uncharacterized/Similar to ovostatin-like; (OVSTL); shares 8 peptides with 455a | F1NEW8_CHICK  (aa562-946)  G1NK51_MELGA  (aa569-942) | 71.7  64.5 | 3.2e-106  2.2e-57 | 8 | a,b,c | 265 | 0.34 |  |
| **15545**  (aa1-195)  (aa137-195) | Neuronal pentraxin-2 (NPTX2) **^1,2,4^** | E1C7S1_CHICK  (aa48-242)  G1MS71_MELGA  (aa1-59) | 98.5  100.0 | 1.1e-63  9.8e-12 | 5 | a,b,c | 19 | <0.01 | **<0.01** |
| **1555**  (aa1-885)  (aa1-885) | Similar to laminin subunit gamma-2 (LAMC2) **^1,2^**; shares 7 peptides with 3846 | E1BXK1_CHICK  (aa26-1172)  G1MSC8_MELGA  (aa26-1174) | 65.7  72.3 | 2.2e-79  2.5e-75 | 15 | a,b,c | 34 | <0.01 | **<0.01** |
| **3846**  (aa77-579)  (aa608-1174) | Similar to laminin subunit gamma-2 (LAMC2); shares 7 peptides with 1555 | G1MSC8_MELGA  (aa77-579)  E1BXK1_CHICK  (aa606-1172) | 74.5  75.2 | 3.1e-47  1.1e-45 | 2 | a,b,c | 6 | <0.01 |  |
| **1577a**  (aa1-271)  (aa1-271) | Similar to protein-tyrosine phosphatase CRYPalpha1 isoform (CRYPalpha1) **^1,2^** | Q90815_CHICK  (aa243-594)  G1N2S0_MELGA  (aa243-594) | 76.7  76.7 | 7.3e-92  7.3e-92 | 1 | b | 1 | <0.01 | **<0.01** |
| **361**  (aa1-1499)  (aa1-1499) | Protein-tyrosine phosphatase CRYPalpha1 | Q90815_CHICK  (aa1-1499)  G1N2S0_MELGA  (aa1-1500) | 100.0  99.4 | 0e0  0e0 | 3 | a,b,c | 7 | <0.01 |  |
| **577**  (aa27-1273)  (aa26-1273) | Protein-tyrosine phosphatase CRYPalpha1 isoform (PTPRS); shares 2 peptides with 1577b | G3UQ69_MELGA  (aa706-1911)  Q90815_CHICK  (aa316-1499) | 91.8  75.5 | 0e0  0e0 | 1 | a | 2 | <0.01 |  |
| **1577b**  (aa275-635)  (aa275-635) | Protein-tyrosine phosphatase (CRYPalpha2) **^1,2^**; shares 1peptide with 14104 (PTPRF) and 2 with 577 (CRYPalpha1) | Q90816_CHICK  (aa1-361)  G3UU53_MELGA  (aa1-359) | 88.7  89.2 | 8.5e-136  5.6e-134 | 2 | a,b,c | 2 | <0.01 | **<0.01** |
| **15784**  (aa1-192)  (aa9-192) | Acidic mammalian chitinase (CBPch04) **^3^** | F1NMM2_CHICK  (aa1-202)  G3UUJ2_MELGA  (aa1-184) | 90.6  94.0 | 5.2e-77  1.7e-75 | 2 | a,b,c | 5 | <0.01 | **<0.01** |
| **15813**  (aa1-175)  (aa1-175) | Hyaluronan and proteoglycan link protein 3(HAPLN3) **^1,2^** | G1N954_MELGA  (aa23-197)  E1BUN0_CHICK  (aa1-175) | 92.0  90.9 | 8.0e-69  2.9e-68 | 7 | a,b,c | 89 | 0.06 | **0.07** |
| **15996**  (aa1-190)  (aa1-177) | Tubulin beta-3 chain (TUBB3) **^1,2^**; shares 4 peptides with 5781 (TUBB2) | F1NMU4_CHICK  (aa240-397)  G3USQ3_MELGA  (aa263-406) | 81.6  79.7 | 1.2e-51  2.6e-50 | 1 | a | 1 | <0.01 | **<0.01** |
| **16030**  (aa1-189) | GTPase HRas (HRAS) | RASH_CHICK  (aa1-189) | 100.0 | 5.1e-59 | 6 | a,b,c | 19 | <0.01 | **<0.01** |
| **1604**  (aa1-870)  (aa216-870) | Carboxypeptidase D (CPD) **^1,2^**, shares 6 peptides with 1909 | E1BYS4_CHICK  (aa8-931)  G1N356_MELGA  (aa4-713) | 89.7  89.3 | 2.2e-213  4.8e-212 | 13 | a,b,c | 29 | <0.01 | **<0.01** |
| **1909**  (aa1-809)  (aa1-809) | Carboxypeptidase D (CPD); shares 6 peptides with 1604 | E1BYS4_CHICK  (aa489-1360)  G1N356_MELGA  (aa271-1141) | 89.6  88.9 | 0e0  0e0 | 3 | a,b,c | 11 | <0.01 |  |
| **16070**  (aa1-189)  (aa1-189) | Ras-related protein Rab-7a (RAB7A) **^2^** | G1N5R3_MELGA  (aa19-207)  E1C0F3_CHICK  (aa19-207) | 100.0  100.0 | 2.3e-74  2.3e-74 | 6 | a,b,c | 23 | <0.01 | **<0.01** |
| **16079**  (aa1-189)  (aa1-189) | Annexin A2 (ANXA2) **^1,2^**; shares 13 peptides with 887b | G1MZD9_MELGA  (aa151-339)  ANXA2_CHICK  (aa151-339) | 99.5  99.5 | 9.2e-76  1.5e-75 | 1 | a,b,c | 11 | 0.01 | **0.03** |
| **887b**  (aa762-1081)  (aa762-1081) | Annexin A2; shares13 peptides with 16079 | ANXA2_CHICK  (aa1-320)  G1MZD9_MELGA  (aa1-320) | 99.7  99.1 | 5.7e-118  3.7e-118 | 23 | a,b,c | 144 | 0.01 |  |
| **16134**  (aa1-188)  (aa1-188) | Delta-1 crystallin (ASL1); shares peptide with 18511 (ASL2) | ARLY1_MELGA  (aa279-466)  ARLY1_CHICK  (aa279-466) | 98.9  98.4 | 1.2e-67  1.4e-67 | 2 | a,b,c | 7 | <0.01 | **<0.01** |
| **16255**  (aa47-187)  (aa47-187)  **5263** | Transmembrane emp24 domain-containing protein 2 (TMED2) | G3UR62_MELGA  (aa3-143)  Q5ZKB0_CHICK  (aa61-201) | 100.0  100.0 | 1.0e-59  1.6e-59 | 2 | a,b | 5 | <0.01 | **<0.01** |
| **18216**  (aa21-165)  (aa21-165)  **16315** | Platelet-derived growth factor receptor-like (PDGFRL) | G1NGA0_MELGA  (aa173-317)  E1C836_CHICK  (aa168-312) | 95.9  96.6 | 4.5e-61  5.2e-61 | 2 | a,b,c | 5 | <0.01 | **<0.01** |
| **16345**  (aa6-186)  (aa6-186) | Serum albumin (ALB) **^1,2; g,t^**; shares 15 peptides with 19800 | G1NCR2_MELGA  (aa29-209)  ALBU_CHICK  (aa29-209) | 95.0  93.4 | 1.5e-77  9.7e-77 | 24 | a,b,c | 527 | 0.62 | **0.39** |
| **19800**  (aa1-149)  (aa1-148) | Serum albumin; shares 15 peptides with 16345; (ALBU_CHICK aa94-241; 88.5; 1.5e-63) | G1NCR2_MELGA  (aa94-242)  F2Z4L6_CHICK  (aa44-191) | 94.6  88.5 | 4.4e-68  6.9e-64 | 4 | a,b,c | 30 | 0.04 |  |
| **4290a**  (aa1-287)  (aa1-280) | Similar to serum albumin (ALBU_CHICK aa243-599; 66.1; 6.3e-65) | G1NCR2_MELGA  (aa243-612)  F2Z4L6_CHICK  (aa235-591) | 70.3  66.7 | 5.7e-69  7.1e-65 | 34 | a,b,c | 409 | 0.19 |  |
| **4872**  (aa7-430)  (aa7-430)  **1636** | Dynein heavy chain 9, axonemal (DNAH9) | F1NVK1_CHICK  (aa512-935)  G1MU29_MELGA  (aa977-1400) | 96.7  95.5 | 1.3e-161  1.1e-160 | 2 | b | 3 | <0.01 | **<0.01** |
| **8210**  (aa1-341)  (aa1-341) | Dynein heavy chain 9, axonemal (DNAH9); | F1NVK1_CHICK  (1190-1530)  G1MU29_MELGA  (aa1655-1997) | 97.7  97.1 | 4.2e-150  2.3e-148 | 2 | a,b,c | 5 | <0.01 |  |
| **16370**  (aa1-146)  (aa31-146) | Histidine phosphatase of the endoplasmic reticulum (HiPER1)/Multiple inositol polyphosphate phosphatase 1 (MINPP1) **^1,2^** | Q92170_CHICK  (aa174-319)  G1MS05_MELGA  (aa1-116) | 94.5  94.8 | 1.1e-60  1.8e-46 | 2 | a,b,c | 5 | <0.01 | **<0.01** |
| **1639**  (aa1-859)  (aa1-859) | Ectonucleotide pyrophosphatase/ phosphodiesterase 2, long form (ENPP2) **^2^** | E1BY59_CHICK  (aa1-859)  G1NIT9_MELGA  (aa1-886) | 100.0  96.5 | 0e0  0e0 | 11 | a,b,c | 19 | <0.01 | **<0.01** |
| **16426**  (aa1-185)  (aa1-185) | Mucin-16 (MUC16) **^2,3^** | F1NIZ7_CHICK  (aa24-230)  G1MSW6_MELGA  (aa58-247) | 82.1  56.8 | 5.1e-47  7.3e-40 | 2 | b,c | 5 | <0.01 | **<0.01** |
| **1643**  (aa1-858)  (aa2-858)  **3005** | Elongation factor 2 (EEF2) **^1,2^** | EF2_CHICK  (aa1-858)  G1NFA2_MELGA  (aa2-864) | 100.0  98.7 | 0e0  0e0 | 12 | a,b,c | 19 | <0.01 | **<0.01** |
| **16433**  (aa1-185)  (aa18-185) | Similar to beta-2-glycoprotein 1 (APOH) **^1,2; t^** | F1NYG4_CHICK  (aa8-373)  G1N5Y2_MELGA  (aa1-345) | 48.4  43.1 | 1.2e-38  1.8e-31 | 2 | a,b,c | 3 | <0.01 | **<0.01** |
| **16495**  (aa7-152)  (aa6-152) | Similar to malate dehydrogenase (MDH1) **^1,2^** | MDHC_CHICK  (aa2-263)  G1N071_MELGA  (aa1-263) | 55.0  54.8 | 1.3e-26  1.3e-26 | 2 | a,b,c | 7 | <0.01 | **<0.01** |
| **26997**  (aa1-91)  (aa1-91) | Malate dehydrogenase (MDH1) | MDHC_CHICK  (aa244-334)  G1N071_MELGA  (aa244-334) | 100.0  100.0 | 2.8e-37  2.8e-37 | 2 | a,b,c | 6 | 0.01 |  |
| **18583**  (aa1-158)  (aa1-158) | Malate dehydrogenase (MDH1) | MDHC_CHICK  (aa68-225)  G1N071_MELGA  (aa68-225) | 99.4  99.4 | 2.2e-65  2.2e-65 | 3 | a,b,c | 7 | <0.01 |  |
| **167**  (aa1-1944) | Fast myosin heavy chain isoform 2 (MYH1B); shares 11 peptides with 173, 9 with 171, 7 with 2895 (other myosins) | Q9DGM5_CHICK  (aa1-1944) | 100.0 | 0e0 | 54 | a,c | 125 | 0.04 | **0.04** |
| **16810**  (aa1-181)  (aa1-181)  **3049** | Cochlin (COCH) **^1^** | COCH_CHICK  (aa367-547)  G1NIG3_MELGA  (aa379-559) | 98.9  98.9 | 2.2e-74  2.3e-74 | 5 | a,b,c | 14 | <0.01 | **<0.01** |
| **16886**  (aa1-180)  (aa6-180)  **18449** | ADP ribosylation factor 4 (ARF4) **^1,2^**; shares 4 peptides with 18374 (ARF1) | Q5ZKR9_CHICK  (aa1-180)  G1N3Z1_MELGA  (aa6-182) | 100.0  92.1 | 2.3e-67  8.0e-60 | 5 | a,b,c | 13 | 0.01 | **0.01** |
| **169**  (aa94-1267)  (aa94-1012) | Similar to vitellogenin-2 **^1,2; g^**; shares 9 peptides with 205, 8 with 1870, and 5 with 5125 | G5E7J5_MELGA  (aa21-1208)  VIT2_CHICK  (aa21-1842) | 68.6  56.5 | 2.7e-74  1.9e-72 | 42 | a,b,c | 200 | 0.01 | **<0.01** |
| **205**  (aa1-1850)  (aa1-1850) | Vitellogenin-2 (VIT2), shares 9 peptides with 169, 2 with 5125, and 3 with Q9PUB0 | VIT2_CHICK  (aa1-1850)  G5E7Q4_MELGA  (aa1-1845) | 100.0  86.4 | 0e0  0e0 | 14 | a,b,c | 41 | <0.01 |  |
| **5125**  (aa1-356)  (aa1-473) | Similar to vitellogenin-2; shares 6 peptides with 169 and 2 with 205; VIT2_CHICK aa714-1487; 53.6%; 4-6e-38 | G5E7J5_MELGA  (aa714-1159)  Q6BCB8_CHICK  (aa628-1400) | 54.9  53.4 | 6.9e-39  3.5e-38 | 3 | a,b,c | 16 | <0.01 |  |
| **Q9PUB0**  (aa1-412)  (aa1-412) | Similar to vitellogenin-2; shares 3 peptides with 205 and 11 with 169 | G5EZJ5_MELGA  (aa80-491)  VIT2_CHICK  (aa80-491) | 89.6  86.7 | 2.1e-170  9.3e-166 | 4 | a,b,c | 40 | <0.01 |  |
| **1690**  (aa55-850)  (aa1-850) | Desmocollin (DSC2) **^1,2^** | G1NDF8_MELGA  (aa57-895)  F1NJD7_CHICK  (aa10-885) | 92.0  87.0 | 0e0  0e0 | 4 | a,b,c | 10 | <0.01 | **<0.01** |
| **1692**  (aa102-575)  (aa118-497) | von Willebrand factor A domain-containing protein 1 (VWA1) **^1,2^**; shares 5 peptides with 9912 | F1NPH3_CHICK  (aa1-486)  G1MUX6_MELGA  (aa18-375) | 93.2  88.7 | 3.2e-113  1.0e-84 | 15 | a,b,c | 70 | 0.01 | **0.01** |
| **9912**  (aa1-283)  (aa1-183) | von Willebrand factor A domain-containing protein 1 (VWA1); shares 5 peptides with 1692 | F1NPH3_CHICK  (aa214-496)  G1MUX6_MELGA  (aa214-375) | 92.2  83.6 | 1.0e-110  6.3e-52 | 2 | a,b | 9 | <0.01 |  |
| **1694**  (aa3-121)  (aa3-121)  **15717** | Myelin protein zero-like protein 2 (MPZL2) **^1^** | G1MX74_MELGA  (aa75-193)  E1C6A5_CHICK  (aa75-193) | 94.1  90.8 | 6.0e-51  5.7e-50 | 3 | a,b,c | 6 | <0.01 | **<0.01** |
| **171**  (aa1-1941)  (aa3-19419)  **252** | Myosin heavy chain (MYH7B); shares 6 peptides with 167, and 8 with 173 | Q8UWA0_CHICK  (aa1-1941)  G1MUV8_MELGA  (aa1-1941) | 100.0  99.2 | 0e0  0e0 | 3 | a | 6 | <0.01 | **<0.01** |
| **17110**  (aa3-178)  (aa3-178) | Procollagen C-endopeptidase enhancer 2 (PCOLCE2) | F1NH70_CHICK  (aa53-228)  G1N443_MELGA  (aa58-233) | 97.7  97.7 | 1.5e-80  1.8e-80 | 5 | a,b,c | 16 | 0.01 | **0.01** |
| **1719**  (aa1-843)  (aa80-843) | Alpha-1,4 glucan phosphorylase (PYGB) | Q5ZME4_CHICK  (aa1-843)  G1MWY3_MELGA  (aa1-766) | 100.0  96.4 | 0e0  0e0 | 6 | a,b,c | 18 | <0.01 | **<0.01** |
| **17243**  (aa18-156)  (aa18-156) | Calmodulin (CALM; variant 2 **^1,2^** | F2Z4K8_CHICK  (aa1-139)  G1NDB0_MELGA  (aa2-140) | 100.0  100.0 | 1.1e-45  1.1e-45 | 2 | a,b | 4 | <0.01 | **<0.01** |
| **17266** | Uncharacterized; domain: VWD |  |  |  | 3 | a,b,c | 7 | <0.01 | **<0.01** |
| **17268**  (aa13-176)  (aa13-176) | Destrin (DSTN) **^1,2^** | Z4YJB8_CHICK  (aa1-164)  G1N515_MELGA  (aa2-165) | 100.0  100.0 | 5.7e-73  5.8e-73 | 7 | a,c | 16 | <0.01 | **<0.01** |
| **17274**  (aa1-176)  (aa2-158) | Carboxylesterase (CES1) | F1NYT3_CHICK  (aa382-557)  H9H259_MELGA  (aa29-185) | 96.0  93.6 | 8.7e-75  2.2e-65 | 2 | a,b,c | 5 | <0.01 | **<0.01** |
| **173**  (aa1-1937)  (aa1-1937) | Myosin heavy chain (MYH15); shares 11 peptides with 167, 9 with 171 and 1 with 2895 | Q9IBD4_CHICK  (aa1-1937)  G1NNJ8_MELGA  (aa1-1937) | 100.0  99.3 | 0e0  0e0 | 3 | a,b,c | 5 | <0.01 | **<0.01** |
| **1730**  (aa1-841)  (aa1-841) | Transforming growth factor-beta type III receptor (TGFBR3) **^1,2^** | Q90998_CHICK  (aa1-841)  G1N8G3_MELGA  (aa1-841) | 100.0  97.1 | 0e0  0e0 | 7 | a,b,c | 29 | <0.01 | **<0.01** |
| **17332**  (aa1-175)  (aa14-175) | ADP-ribosylation factor 6 (ARF6) **^1^** | ARF6_CHICK  (aa1-175)  G1NLU5_MELGA  (aa2-163) | 99.4  96.9 | 3.8e-68  7.5e-61 | 4 | a,b,c | 11 | <0.01 | **<0.01** |
| **17386**  (aa1-162)  (aa1-162) | Cell division control protein 42 homolog (CDC42) **^1,2^** | CDC42_CHICK  (aa1-162)  G1N4S3_MELGA  (aa1-162) | 100.0  100.0 | 3.4e-66  3.4e-66 | 6 | a,b,c | 13 | <0.01 | **<0.01** |
| **17452**  (aa1-137) | Protein phosphatase 1L (PPM1L) **^1,2^** | E1BTL4_CHICK  (aa6-142) | 94.9 | 1.2e-52 | 3 | a,b,c | 20 | 0.01 | **0.01** |
| **24040**  (aa1-114)  (aa1-113) | Protein phosphatase 1L (PPM1L) | E1BTL4_CHICK  (aa247-360)  G1NDI2_MELGA  (aa133-245) | 99.1  99.1 | 1.7e-46  4.2e-46 | 6 | a,b,c | 27 | 0.01 |  |
| **28361**  (aa11-76)  (aa11-76) | Protein phosphatase 1L (PPM1L) | G1NDI2_MELGA  (aa19-84)  E1BTL4_CHICK  (aa133-198) | 89.4  90.9 | 4.3e-18  4.5e-18 | 2 | a,c | 4 | <0.01 |  |
| **17464**  (aa1-150)  (aa1-150) | Tripeptidyl peptidase 1 (TPP1) **^1,2^** | H9H1Z2_MELGA  (aa198-258)  F1NB83_CHICK  (aa120-270) | 94.0  96.0 | 2.3e63  1.9e-60 | 3 | a,b,c | 18 | 0.01 | **0.01** |
| **17502**  (aa27-173)  (aa27-173) | Tumor susceptibility gene 101 protein (TSG101) **^1,2^**; shares 3 peptides with 1890b | G1N6A3_MELGA  (aa17-163)  E1BWW2_MELGA  (aa15-161) | 99.3  98.0 | 2.5e-64  6.9e-64 | 1 | a | 1 | <0.01- | **<0.01** |
| **1890b**  (aa544-756)  (aa550-756) | Similar to tumor susceptibility 101 protein; shares 3 peptides with 17502 | E1BWW2_CHICK  (aa12-364)  G1N6A3_MELGA  (aa8-366) | 56.4  55.2 | 3.2e-40  3.2e-40 | 8 | a,b,c | 24 | <0.01 |  |
| **17523**  (aa29-172)  (aa29-172) | Similar to pyruvate kinase (PKM) **^1,2^**; shares 2 peptides with 7153 | KPYM_CHICK  (aa1-188)  G1MSA4_MELGA  (aa1-188) | 73.9  72.9 | 2.2e-29  4.2e-29 | 1 | a,b,c | 4 | <0.01 | **0.01** |
| **7153**  (aa7-371)  (aa7-371) | Pyruvate kinase (PKM); shares 2 peptides with 17523 | KPYM_CHICK  (aa1-329)  G1MSA4_MELGA  (aa1-329) | 87.9  87.4 | 3.0e-50  3.0e-50 | 11 | a,b,c | 28 | 0.01 |  |
| **1756**  (aa1-377)  (aa1-377) | Proactivator polypeptide (PSAP) **^1,2^**; shares 23 peptides with 2306 | E1BSP1_CHICK  (aa60-471)  G1MZV1_MELGA  (aa60-518) | 90.0  76.0 | 1.2e-95  3.6e-81 | 3 | a,b,c | 78 | 0.03 | **0.12** |
| **2306**  (aa9-420)  (aa21-420) | Proactivator polypeptide/saposin; shares 23 peptides with 1756 | G1MZV1_MELGA  (aa5-518)  E1BSP1_CHICK  (aa16-471) | 75.7  86.4 | 9.9e-104  1.4e-101 | 28 | a,b,c | 361 | 0.21 |  |
| **17566**  (aa1-172) | Thyroglobulin (TG) | F1NIW9_CHICK  (aa2358-2529) | 95.3 | 3.2e-66 | 3 | a,b,c | 25 | 0.01 | **<0.01** |
| **28383**  (aa1-45) | Thyroglobulin | F1NIW9_CHICK  (aa2568-2612) | 95.6 | 1.0e-12 | 1 | a,b,c | 8 | 0.01 |  |
| **4130**  (aa1-551) | Thyroglobulin; shares 10 peptides with 841 | F1NIW9_CHICK  (aa306-856) | 94.6 | 0e0 | 1 | a | 1 | <0.01 |  |
| **841**  (aa2-1090) | Similar to thyroglobulin; shares 10 peptides with 4130 | F1NIW9_CHICK  (aa289-2260) | 56.4 | 3.7e-110 | 21 | a,b,c | 92 | 0.01 |  |
| **17613**  (aa7-172)  (aa14-172) | Ras-related protein Rab-10 (RAB10) **^1,2^** | G3UR98_MELGA  (aa9-174)  F1NMT5_CHICK  (aa6-164) | 96.4  99.4 | 8.9e-65  3.0e-64 | 3 | a,b,c | 19 | <0.01 | **<0.01** |
| **18290**  (aa39-164)  (aa39-164)  **17702** | Histone H2B (H2B-VIII) | G1NRI6_MELGA  (aa1-126)  H2B8_CHICK  (aa1-126) | 100.0  100.0 | 2.8e-43  2.8e-43 | 4 | a,b,c | 28 | 0.01 | **0.01** |
| **17749**  (aa34-166)  (aa35-166) | Ceroid-lipofuscinosis neuronal protein 5 (CLN5) **^1,2^** | F1NZF1_CHICK  (aa46-178)  G1NPZ1_MELGA  (aa2-137) | 94.7  93.9 | 3.9e-63  1.6e-62 | 6 | a,b,c | 16 | 0.01 | **0.01** |
| **17881**  (aa2-169)  (aa2-169) | Ceroid-lipofuscinosis neuronal protein 5 (CLN5) | F1NZF1_CHICK  (aa180-347)  G1NPZ1_MELGA  (aa135-302) | 97.6  97.6 | 8.9e-75  1.3e-74 | 3 | a,b,c | 10 | 0.01 |  |
| **17758**  (aa1-171)  (aa1-171) | Protein-L-isoaspartate O-methyltransferase (LOC100542686/LOC423008) **^1,2; t^** | G1MVQ6_MELGA  (aa55-225)  E1BXJ0_CHICK  (aa75-245) | 93.0  93.0 | 5.8e-58  6.4e-58 | 6 | a,b,c | 15 | <0.01 | **<0.01** |
| **17778**  (aa1-148) | Uncharacterized/similar to A2ML1 **^1^**; shares 3 peptides with 30 | G1NME9_MELGA  (aa361-514) | 87.0 | 2.3e-58 | 4 | a,b,c | 21 | 0.02 | **0.01** |
| **23225**  (aa1-115)  (aa2-115) | Alpha-2-macroglobulin-like protein 1 (A2ML1); shares 1 peptide with 30 | G1NME9_MELGA  (aa27-141)  F1NTK2_CHICK  (aa21-134) | 86.1  43.5 | 9.5e-44  2.1e-14 | 1 | a,c | 4 | <0.01 |  |
| **9823**  (aa9-247) | Similar to alpha-2-macroglobulin (A2ML1) | F1NTK2_CHICK  (aa559-809) | 65.1 | 4.4e-47 | 2 | a,c | 6 | <0.01 |  |
| **17826**  (aa8-170)  (aa8-170) | Cathepsin B (CTSB) **^1,2^**; shares 8 peptides with 8924 | G1NN37_MELGA  (aa178-340)  F1N9D8_CHICK  (aa178-340) | 99.4  96.9 | 4.3e-72  1.9e-70 | 4 | a,b,c | 47 | 0.03 | **0.18** |
| **8924**  (aa1-321)  (aa1-321) | Cathepsin B (CTSB); shares 8 peptides with 17826 | F1N9D8_CHICK  (aa1-340)  G1NN37_MELGA  (aa1-340) | 76.8  77.6 | 8.5e-82  1.2e-81 | 20 | a,b,c | 263 | 0.24 |  |
| **17855**  (aa1-169) | Ribosomal protein S3 **^1,2,4^** | Q6EE58_CHICK  (aa1-169) | 100.0 | 5.0e-69 | 6 | a,b,c | 13 | <0.01 | **<0.01** |
| **1786**  (aa1-829)  (aa33-829) | Tumor necrosis factor alpha converting enzyme (ADAM17) **^2^** | Q5ZL93_CHICK  (aa1-829)  G1NMJ9_MELGA  (aa1-797) | 100.0  97.4 | 0e0  0e0 | 2 | a,b,c | 6 | <0.01 | **<0.01** |
| **17901**  (aa1-169)  (aa1-169) | Carbonic anhydrase 4 (CA4) **^1,2,4^** | E1C004_CHICK  (aa163-331)  G1N6Y5_MELGA  (aa162-337) | 87.6  84.7 | 1.1e-65  1.4e-63 | 6 | a,b,c | 49 | 0.03 | **0.03** |
| **21200**  (aa1-137)  (aa1-137)  **1811** | Cellular retinoic acid-binding protein 1 (CRABP1) **^1,2^** | B0FLN8_CHICK  (aa1-137)  H9H0C7_MELGA  (aa1-137) | 99.3  74.6 | 1.0e-61  9.6e-46 | 2 | a,c | 3 | <0.01 | **<0.01** |
| **1812**  (aa73-825)  (aa73-825) | Similar to collagen XVIII (COL18A1) **^1,2^**; shares 13 peptides with 495 | O93419_CHICK  (aa29-1344)  G1MTC0_MELGA  (aa19-1333) | 52.2  51.2 | 1.9e-93  3.3e-92 | 19 | a,b,c | 179 | 0.03 | **<0.01** |
| **495**  (aa1-1344)  (aa11-1344) | Collagen XVIII (COL18A1); shares 13 peptides with 1812 | O93419_CHICK  (aa1-1344)  G1MTC0_MELGA  (aa1-1333) | 100.0  94.5 | 0e0  0e0 | 2 | a,b,c | 19 | <0.01 |  |
| **18134**  (aa1-146)  (aa1-146) | Plakoglobin (JUP) **^1,2^** | G1NCE5_MELGA  (aa86-231)  E1C1V3_CHICK  (aa94-239) | 100.0  100.0 | 1.2e-54  3.1e-54 | 3 | a,b | 7 | <0.01 | **<0.01** |
| **18154**  (aa1-166)  (aa1-166) | Similar to isoform 2 of cAMP-dependent protein kinase type II-alpha regulatory subunit (PRKAR2A) | E1C9H8_CHICK  (aa179-400)  G1N973_MELGA  (aa83-304) | 73.4  73.0 | 1.7e-36  2.4e-36 | 2 | a,b | 4 | <0.01 | **<0.01** |
| **18232**  (aa1-165) | Peptidyl-prolyl cis-trans isomerase B (PPIB) **^1^**; shares 1 peptide with 20527 | PPIB_CHICK  (aa43-207) | 98.2 | 4.3e-69 | 11 | a,b,c | 70 | 0.05 | **0.06** |
| **18344**  (aa2-121)  (aa2-121) | Insulin-like growth factor-binding protein 7 (IGFBP7) **^1,2^** | G1N8M9_MELGA  (aa17-136)  F1NVP4_CHICK  (aa184-303) | 97.5  96.7 | 6.2e-49  4.4e-48 | 8 | a,b,c | 74 | 0.09 | **0.10** |
| **18374**  (aa1-164)  (aa1-164) | ADP-ribosylation factor 1 (ARF1) **^1,2^**; shares 4 peptides with 16886 (ARF4) and 3 with 18449 (ARF4) | F1NN08_CHICK  (aa18-181)  G1MUL7_MELGA  (aa18-181) | 100.0  100.0 | 1.0e-61  1.0e-61 | 10 | a,b,c | 34 | 0.02 | **0.02** |
| **1843**  (aa451-704)  (aa451-701) | Galactosylceramide sulfotransferase (GAL3ST1) **^2^**; shares 14 peptides with 8093 and 1 with 6555 (GAL3ST2) | G1NNM9_MELGA  (aa66-319)  F1NP56_CHICK  (aa59-312) | 94.9  94.1 | 7.1e-60  2.4e-59 | 1 | c | 3 | <0.01 | **0.01** |
| **8093**  (aa1-345)  (aa1-345) | Galactosylceramide sulfotransferase (GAL3ST1); shares 14 peptides with 1893 and 1 with 6555 (GAL3ST2) | G1NNM9_MELGA  (aa89-433)  F1NP56_CHICK  (aa82-426) | 98.0  97.4 | 4.6e-160  2.9e-159 | 15 | a,b,c | 74 | 0.02 |  |
| **18430**  (aa6-163)  (aa6-163) | GTP-binding protein SAR1b (SAR1B) **^2,4^** | R4GFN4_CHICK  (aa1-160)  G1N8D5_MELGA  (aa1-160) | 97.5  97.5 | 1.7e-53  1.7e-53 | 2 | a,b,c | 5 | <0.01 | **<0.01** |
| **1851**  (aa2-772) | Uncharacterized/similar to lipoxygenase; domains: PLAT/LH2 | G1NEP4_MELGA  (aa40-867) | 23.4 | 4.2e-13 | 5 | a,b,c | 14 | <0.01 | **<0.01** |
| **18511**  (aa22-162)  (aa22-162) | Argininosuccinate lyase (ASL2); shares 1 peptide with 16134 (ASL1) | G1MZB0_MELGA  (aa326-466)  F1NI02_CHICK  (aa325-465) | 96.5  95.7 | 2.0e-49  2.3e-49 | 3 | a,b,c | 6 | <0.01 | **<0.01** |
| **18627**  (aa1-141)  (aa46-141) | Transcobalamin-2 (TCN2) **^2^** | G1NNM7_MELGA  (aa57-197)  H9KZM9_CHICK  (aa3-98) | 89.4  91.7 | 1.7e-56  1.2e-37 | 9 | a,b,c | 49 | 0.01 | **0.01** |
| **18628**  (aa20-161)  (aa20-161) | Uncharacterized (similar to ERGIC-53/LMAN1) **^1^** | G1MR70_MELGA  (aa53-315)  Q5F3E6_CHICK  (aa117-376) | 53.2  52.7 | 7.8e-21  8.5e-20 | 3 | a,b,c | 4 | 0.01 | **<0.01** |
| **18629**  (aa31-161)  (aa34-161) | Ovotransferrin (TRFE) **^1,2; i^**; shares 5 peptides with 2596 | G1MVV5_MELGA  (aa563-693)  TRFE_CHICK  (aa566-693) | 84.7  82.0 | 1.0e-46  4.6e-44 | 11 | a,b,c | 219 | 0.22 | **0.19** |
| **24968**  (aa1-104)  (aa1-104) | Ovotransferrin (TRFE/TF/TFEW); TRFE_CHICK aa70-173; 88.5; 5.4e-38 | G1MVV5_MELGA  (aa70-173)  E1BQC2_CHICK  (aa70-173) | 92.3  90.4 | 8.5e-40  9.2e-39 | 7 | a,b,c | 137 | 0.40 |  |
| **2596**  (aa1-705)  (aa1-705) | Ovotransferrin (TFEW/TFRE); shares 5 peptides with 18629; TRFE_CHICK aa1-705; 98.9%; 0e0 | Q4ADJ7_CHICK  (aa1-705)  G1MVV5_MELGA  (aa1-705) | 99.0  90.4 | 0e0  0e0 | 19 | a,b,c | 228 | 0.11 |  |
| **1870**  (aa1-799)  (aa89-799) | Similar to vitellogenin **^1,2^**; shares 8 peptides with 169 | E1BYN5_CHICK  (aa62-1452)  G1NAD5_MELGA  (aa1-1388) | 51.0  45.5 | 1.0e-111  1.0e-74 | 4 | a,b,c | 13 | <0.01 | **<0.01** |
| **8527a**  (aa1-100)  (aa1-100) | Similar to vitellogenin | E1BYN5_CHICK  (aa1504-1640)  G1NAD5_MELGA  (aa1440-1576) | 67.9  64.2 | 5.9e-31  2.0e-29 | 1 | a,b,c | 10 | <0.01 |  |
| **18757**  (aa1-159)  (aa1-159) | L-lactate dehydrogenase A chain (LDHA) **^1,2,4; i^**; shares 1 peptide with 22431 (LDHB) | LDHA_CHICK  (aa174-332)  G1N679_MELGA  (aa174-332) | 98.1  96.9 | 1.5e-67  3.4e-67 | 1 | a,b,c | 4 | <0.01 | **<0.01** |
| **28010**  (aa1-81)  (aa1-81) | L-lactate dehydrogenase A chain (LDHA);  LDHA_CHICK aa1-81; 98.8%; 1.5e-30 | E1BTT8_CHICK  (aa1-81)  G1N679_MELGA  (aa1-81) | 100.0  97.5 | 2.5e-31  1.3e-30 | 2 | a,c | 3 | 0.01 |  |
| **18758**  (aa7-159)  (aa7-159) | Similar to EMILIN (EMILIN2); domains: complement_C1q/TNF_like | F1NBG2_CHICK  (aa899-1052)  G1NBE1_MELGA  (aa905-1058) | 39.1  39.1 | 5.3e-14  5.4e-14 | 3 | a,b,c | 11 | <0.01 | **0.01** |
| **18780**  (aa23-153)  (aa26-153) | Calcium and integrin-binding protein 1 (CIB1) **^1,2^** | F1P103_CHICK  (aa70-193)  G1NDJ9_MELGA  (aa2-122) | 87.8  85.9 | 5.0e-42  1.3e-40 | 7 | a,b,c | 22 | 0.01 | **0.01** |
| **18796**  (aa1-129)  (aa1-129) | Insulin-like growth factor-II (IGF2) **^1,2^** | Q5MGT1_MELGA  (aa7-135)  IGF2_CHICK  (aa7-135) | 99.2  98.4 | 6.6e-57  1.6e-56 | 5 | a,b,c | 54 | 0.06 | **0.07** |
| **188**  (aa1-1912)  (aa1-1912) | Vitellogenin-1 **^1,2^**; shares 8 peptides with 746 | VIT1_CHICK  (aa1-1912)  G1NAV2_MELGA  (aa1-1920) | 100.0  89.7 | 0e0  7.0e-191 | 6 | a,c | 10 | <0.01 | **0.01** |
| **746**  (aa1-1164)  (aa1-1164) | Similar to vitellogenin-1 (VTG1); shares 8 peptides with 188; VIT1_CHICK aa256-1912; 61.5%; 9.1e-139 | F1P350_CHICK  (aa156-1912)  G1NAU9_MELGA  (aa156-1810) | 61.6  65.4 | 7.0e-142  5.8e-138 | 31 | a,b,c | 216 | 0.01 |  |
| **18867**  (aa2-158)  (aa2-158) | Peroxiredoxin-6 (PRDX6) **^1,2,4^** | G1MYX6_MELGA  (aa37-194)  F1NBV0_CHICK  (aa67-224) | 91,1  90.5 | 6.0e-58  1.6e-57 | 7 | a,b,c | 19 | <0.01 | **<0.01** |
| **18915**  (aa14-158)  (aa21-158) | Calcitonin (CALCA) | G1N4N0_MELGA  (aa1-145)  B0FYW9_CHICK  (aa1-138) | 99.3  99.3 | 1.2e-61  7.2e-58 | 9 | a,b,c | 25 | 0.01 | **0.01** |
| **18974**  (aa1-157)  (aa1-157) | Coagulation factor 8 (F8) **^1,2^**; shares 4 peptides with 339 | F1NPT2_CHICK  (aa1913-2069)  G1NAE9_MELGA  (aa1913-2065) | 95.5  59.9 | 7.3e-68  4.8e-34 | 2 | a,b | 10 | 0.01 | **0.01** |
| **274**  (aa13-1695)  (aa13-1695) | Coagulation factor 8; shares 26 peptides with 339 | F1NPT2_CHICK  (aa133-1909)  G1NAE9_MELGA  (aa133-1909) | 85.9  85.7 | 0e0  0e0 | 40 | a,b,c | 268 | 0.02 |  |
| **339**  (aa45-1525)  (aa45-1466) | Similar to coagulation factor 8; shares 26 peptides with 274, and 4 with 18974 | F1NPT2_CHICK  (aa93-2018)  G1NAE9_MELGA  (aa93-1959) | 69.5  68.1 | 6.2e-129  6.3e-128 | 9 | a,b,c | 35 | <0.01 |  |
| **19048**  (aa1-156)  (aa1-156) | RAB27a **^2^** | G1N2V2_MELGA  (aa1-156)  D2D3P4_CHICK  (aa1-156) | 100.0  100.0 | 2.8e-68  2.8e-68 | 7 | a,b,c | 12 | 0.01 | **<0.01** |
| **29886**  (aa1-65)  (aa1-65) | Rab27a | D2D3P4_CHICK  (aa157-221)  G1N2V2_MELGA  (aa157-221) | 100.0  100.0 | 6.6e-28  6.6e-28 | 2 | b,c | 3 | <0.01 |  |
| **19118**  (aa13-156)  (aa13-156) | DnaJ subfamily B member 9 (DNAJB9) **^1,2,4^** | G1NAH6_MELGA  (aa73-216)  Q5ZIX7_CHICK  (aa73-216) | 100.0  98.6 | 5.2e-64  4.5e-63 | 4 | a,c | 6 | <0.01 | **<0.01** |
| **1913**  (aa1-806)  (aa101-806) | Transitional endoplasmic reticulum ATPase (VCP) **^1,2^** | Q5ZMU9_CHICK  (aa1-806)  G1MUF1_MELGA  (aa1-705) | 100.0  94.2 | 0e0  1.4e-202 | 9 | a,b,c | 14 | <0.01 | **<0.01** |
| **19142**  (aa3-131)  (aa3-131) | Meteorin-like protein (METRNL) **^1,2^** | G1MWQ7_MELGA  (aa2-130)  R4GHN2_CHICK  (aa2-130) | 100.0  100.0 | 2.3e-56  2.3e-56 | 7 | a,b,c | 26 | 0.01 | **0.01** |
| **24425**  (aa7-111)  (aa7-111) | Meteorin-like protein (METRNL) | R4GHN2_CHICK  (aa151-255)  G1MWQ7_MELGA  (aa151-255) | 99.0  97.1 | 8.4e-46  1.2e-45 | 4 | a,b,c | 14 | <0.01 |  |
| **19158**  (aa1-155)  (aa1-155) | Growth arrest-specific protein 6 (GAS6) **^1,2^** | G1NPQ8_MELGA  (aa537-691)  F1P3F0-CHICK  (aa517-671) | 96.1  92.9 | 3.4e-58  1.8e-56 | 5 | a,b,c | 22 | 0.01 | **0.01** |
| **3409**  (aa34-506)  (aa34-428) | Growth arrest-specific protein 6 (GAS6) | F1P3F0_CHICK  (aa151-621)  G1NPQ8_MELGA  (aa127-565) | 83.4  87.5 | 2.7e-156  1.7e-111 | 10 | a,b,c | 22 | <0.01 |  |
| **19295**  (aa1-154)  (aa1-153) | Tumor necrosis factor receptor superfamily member 6B (TNFRSF6B) **^1,2,4^** | G1N4U8_MELGA  (aa135-290)  F1NCY6_CHICK  (aa142-196) | 88.5  87.1 | 4.8e-53  1.1e-51 | 5 | a,b,c | 17 | 0.01 | **0.01** |
| **19315**  (aa6-154)  (aa8-154) | Transmembrane emp24 domain-containing protein 10 (TEMED10) | G1NJE1_MELGA  (aa1-149)  F1NA76_CHICK  (aa73-219) | 98.7  100.0 | 2.1e-60  4.1e-60 | 3 | a,b,c | 13 | <0.01 | **<0.01** |
| **1940**  (aa1-802)  (aa53-802) | Spondin-1 (SPON1) **^1,2,4^** | SPON1_CHICK  (aa1-802)  G1MR32_MELGA  (aa1-750) | 100.0  99.6 | 0e0  0e0 | 20 | a,b,c | 178 | 0.04 | **0.05** |
| **19409**  (aa1-153) | Alpha-galactosidase A (GLA) **^1,2^** | E1BT44_CHICK  (aa257-409) | 98.0 | 1.1e-68 | 3 | a,b | 6 | 0.01 | **0.01** |
| **19411**  (aa1-64)  (aa1-68) | Nephronectin (Gga.54550) **^1,2,4^** | E1C8K1_CHICK  (297-360)  G1N2S7_MELGA  (aa359-426) | 96.9  88.2 | 1.1e-24  3.4e-23 | 3 | a,b,c | 17 | 0.01 | **0.01** |
| **19899**  (aa1-148)  (aa26-148)  **19450** | Similar to 6-phosphogluconate dehydrogenase, decarboxylating (PGD) **^2^** | Q5ZIZ0_CHICK  (aa4-281)  G1MZ27_MELGA  (aa1-253) | 52.9  48.6 | 4.4e-29  1.4e-19 | 2 | a,b,c | 3 | <0.01 | **<0.01** |
| **19644**  (aa1-151)  (aa1-151) | Peroxiredoxin-1 (PRDX1) **^1,2^** | G1NDB7_MELGA  (aa21-171)  PRDX1_CHICK  (aa21-171) | 97.4  97.4 | 1.8e-63  4.1e-63 | 7 | a,b,c | 45 | 0.02 | **0.02** |
| **19650**  (aa1-151)  (aa1-149) | Uncharacterized/similar to FAM173A | E1BSP4_CHICK  (aa1-215)  G1NBS1_MELGA  (aa9-221) | 64.2  64.8 | 1.0e-27  1.1e-27 | 3 | a,b,c | 8 | <0.01 | **<0.01** |
| **19686**  (aa10-150)  (aa10-150) | Glutathione peroxidase (GPX3) **^1,2; i,t^** | G1N365_MELGA  (aa5-145)  F1NPJ8_CHICK  (aa81-221) | 98.6  99.3 | 5.7e-63  7.7e-63 | 10 | a,b,c | 118 | 0.32 | **0.36** |
| **1971**  (aa1-795)  (aa1-795) | Heat shock protein 90kDa beta (HSP90B1)/endoplasmin **^1,2,4^**, C7G494_COTJA; shares 3 peptides with C7G498 (HSP90AB1) | ENPL_CHICK  (aa1-795)  G1NK19_MELGA  (aa1-795) | 100.0  99.5 | 0e0  0e0 | 6 | a,b,c | 14 | <0.01 | **<0.01** |
| **1976**  (aa1-444)  (aa1-346) | ATP synthase subunit alpha (ATP5A1) **^1,2^** | F1NI22_CHICK  (aa48-527)  G1NC68_MELGA  (aa2-347) | 92.5  98.0 | 1.0e-109  1.1e-106 | 8 | a,b,c | 16 | <0.01 | **<0.01** |
| **28103**  (aa31-82)  **19762** | EGF-like repeat and discoidin I-like domain-containing protein 3 (EDIL3) **^1,2,4; i^** | F1NCN3_CHICK  (aa380-431) | 98.1 | 1.8e-18 | 6 | a,b,c | 594 | 2.35 | **1.65** |
| **20199**  (aa97-146) | EDIL3 | F1NCN3_CHICK  (aa431-480) | 96.0 | 1.4e-17 | 5 | a,b,c | 248 | 1.14 |  |
| **25038**  (aa1-100)  (aa1-100) | EDIL3 | G3UT43_MELGA  (aa3-102)  F1NCN3_CHICK  (aa218-317) | 99.0  100.0 | 1.5e-43  4.1e-43 | 19 | a,b,c | 353 | 1.41 |  |
| **19789**  (aa1-149)  (aa1-149) | Protein APCDD1 (Adenomatosis polyposis coli down-regulated 1) **^1,2^** | APCD1_CHICK  (aa367-515)  G1N9Y6_MELGA  (aa368-516) | 100.0  99.3 | 5.5e-64  3.0e-63 | 4 | a,b,c | 14 | 0.01 | **0.01** |
| **7974**  (aa1-339)  (aa6-339) | Protein APCDD1 | APCD1_CHICK  (aa1-365)  G1N9Y6_MELGA  (aa10-366) | 92.6  85.8 | 6.1e-126  2.1e-116 | 12 | a,b,c | 55 | 0.01 |  |
| **19793**  (aa70-149) | Similar to Xyloside xylosyltransferase 1 (XXYLT1) **^1,2^**  (R0K4Y8_ANAPL; 91.9%; 2.8e-67) | G1N5B2_MELGA  (aa108-187) | 95.0 | 4.6e-35 | 8 | a,b,c | 15 | 0.01 | **0.01** |
| **19859**  (aa12-149)  (aa12-149) | Immunoglobulin J chain (IGJ) **^1,2^** | E1BY98_CHICK  (aa21-158)  G1NC90_MELGA  (aa21-159) | 94.9  93.5 | 1.8e-61  7.3e-60 | 3 | a,b,c | 8 | <0.01 | **0.01** |
| **19873**  (aa8-149)  (aa8-149) | Glutathione S-transferase omega-1  (GSTO1) **^1,2^** | E1BX85_CHICK  (aa48-189)  G1NDX0_MELGA  (aa48-190) | 91.5  86.7 | 4.9e-55  2.8e-52 | 3 | a,b,c | 6 | <0.01 | **<0.01** |
| **1989b**  (aa613-794) | Similar to annexin (A11; RCJMB0_6f1) **^1,2^** | Q5ZLG6_CHICK  (aa89-347) | 68.7 | 7.0e-28 | 6 | a,b,c | 19 | <0.01 | **<0.01** |
| **19984**  (aa2-143)  (aa2-143) | Integral membrane protein 2A (ITM2A) **^1,2^**; shares 1 peptide with 21988 | F1P4X6_CHICK  (aa31-172)  G1MSA8_MELGA  (aa41-182) | 99.3  99.3 | 4.3e-63  4.5e-63 | 3 | a,b,c | 16 | <0.01 | **<0.01** |
| **21988**  (aa15-130)  (aa15-130) | Integral membrane protein 2A; shares 1 peptide with 19984 | F1P4X6_CHICK  (aa136-251)  G1MSA8_MELGA  (aa146-261) | 99.1  98.3 | 1.5e-50  4.9e-50 | 1 | a,b,c | 13 | <0.01 |  |
| **19986**  (aa1-147)  (aa1-148) | Tumor necrosis factor ligand superfamily member 10 (TNFSF10) **^2^** | G1NCC1_MELGA  (aa94-240)  Q5ZK93_CHICK  (aa145-292) | 95.9  94.6 | 6.1e-65  1.2e-64 | 5 | a,b,c | 8 | <0.01 | **<0.01** |
| **E1ABY0**  (aa1-54)  (aa21-54**)** | Tumor necrosis factor ligand superfamily member 10 (Trail; TNFSF10) | F1NHP5_CHICK  (aa30-83)  G1NCC1_MELGA  (aa2-33) | 98.1  91.2 | 1.9e-21  1.3e-8 | 2 | a,c | 3 | <0.01 |  |
| **20048**  (aa13-147)  (aa13-147) | ADP-ribose pyrophosphatase, mitochondrial (NUDT9) **^2,3^** | F1NSM1_CHICK  (aa223-357)  G1N6G8_MELGA  (aa182-316) | 97.8  97.0 | 1.1e-53  3.6e-53 | 4 | a,b,c | 8 | 0.01 | **0.01** |
| **20075**  (aa1-147) | 14-3-3 protein zeta (YWHAZ) **^1,2^**; shares 1 peptide with 13891, 13907, 13883 | 1433Z_CHICK  (aa99-254)  G1NI29_MELGA  (aa101-247) | 100.0  100.0 | 3.8e-60  3.8e-60 | 4 | a,b,c | 21 | <0.01 | **<0.01** |
| **23637**  (aa2-117)  (aa4-117) | 14-3-3 protein zeta; shares 2 peptides with 29781 and 1 with 13891 | G1NI29_MELGA  (aa1-125)  1433Z_CHICK  (aa1-123) | 83.2  83.7 | 8.7e-35  2.7e-34 | 1 | a,b,c | 12 | <0.01 |  |
| **2010**  (aa1-789)  (aa1-789) | Trans Golgi network protease furin (FURIN) **^1,2^**; shares 2 peptides with 4905 | Q91000_CHICK  (aa1-789)  G1NDT8_MELGA  (aa5-734) | 100.0  87.7 | 0e0  5.8e-178 | 6 | a,b,c | 20 | <0.01 | **<0.01** |
| **4905**  (aa1-489)  (aa1-489) | Similar to trans Golgi network protease furin ; shares 2 peptides with 2010 | Q91000_CHICK  (aa1-789)  G1NDT8_MELGA  (aa5-734) | 57.3  61.7 | 7.4e-76  1.2e-74 | 1 | a | 1 | <0.01 |  |
| **20126**  (aa1-147)  (aa1-147) | Hemoglobin beta subunit (HBB) **^1,2^** | HBB_CHICK  (aa1-147)  G1U9Q8_MELGA  (aa1-147) | 97.3  97.3 | 8.7e-63  1.0e-62- | 8 | a,b,c | 21 | <0.01 | **0.01** |
| **20161**  (aa1-101)  (aa1-106) | Parvalbumin beta (LOC427654); shares 1 peptide with 24618 (Parvalbumin, thymic) | E1BX24_CHICK  (aa18-118)  G1MRW5_MELGA  (aa1-106) | 97.0  67.9 | 3.3e-35  5.5e-25 | 2 | a,b | 2 | <0.01 | **<0.01** |
| **20214**  (aa1-90) | Ras-related protein Rab-11A (RAB11A) **^1,2^**; shares 2 peptides with 21797 and 1 with 12944 (RAB11B) | RBMA_CHICK  (aa1-90) | 100.0 | 1.2e-23 | 1 | a,c | 4 | <0.01 | **<0.01** |
| **20284**  (aa7-145)  (aa7-145) | TIMP-2 (TIMP2) **^1,2,4^** | R4GIL5_CHICK  (aa48-220)  G3UTJ0_MELGA  (aa19-190) | 78.6  79.8 | 6.2e-52  1.9e-50 | 5 | a,b,c | 24 | 0.01 | **0.02** |
| **20375**  (aa1-88) | Similar to Bactericidal/permeability-increasing protein-like 2/BPI fold-containing family C protein; A0A093BK32_  9AVES; 73.9%; 5.6e-21; IPI00571823 **^1,4; i,t^** | F1NYJ8_CHICK  (aa31-398) | 45.5% | 8.4e-5 | 2 | a,b,c | 30 | 0.12 | **0.21** |
| **28084**  (aa1-80) | Uncharacterized; domains: Bactericidal/ permeability-increasing protein 2 (aa1-81);  R0KBG4_ANAPL aa36-115; 78.8%; 2.1e-26; IPI00571823 | F1NYJ8_CHICK  (aa33-112) | 42.5 | 7.2e-12 | 5 | a,b,c | 124 | 0.32 |  |
| **20396**  (aa10-144)  (aa10-144) | Similar to nucleoside diphosphate kinase (LOC100549849) **^1,2^** | G1N8T1_MELGA  (aa9-153)  NDK_CHICK  (9-153) | 79.3  77.9 | 2.1e-44  6.9e-44 | 4 | a,b,c | 11 | <0.01 | **<0.01** |
| **20430**  (aa31-144)  (aa31-143) | Similar to prostate stem cell antigen (PSCA) **^1,2,4; t^** | F1NXM7_CHICK  (aa9-122)  G1NJY7_MELGA  (aa15-127) | 78.1  75.2 | 2.0e-37  1.4e-36 | 8 | a,b,c | 124 | 0.29 | **0.34** |
| **20482**  (aa6-143)  (aa6-143) | Ovalbumin (SERPINB14) **^1,2; i^**; shares 10 peptides with Q6V115/P19104 | G1MYL1_MELGA  (aa1-155)  OVAL_CHICK  (aa1-155) | 78.1  76.1 | 2.3e-29  8.6e-27 | 2 | a,b,c | 49 | 0.03 | **15.97** |
| **20852**  (aa9-140)  (aa9-140) | Ovalbumin; shares 7 peptides with Q6V115, P19104 | OVAL_CHICK  (aa255-386)  G1MYK6_MELGA  (aa255-386) | 88.6  97.0 | 4.7e-48  3.6e-46 | 1 | a,b,c | 527 | 4.86 |  |
| **Q6V115**  (aa1-383)  (aa1-383) | Ovalbumin; shares 9 peptides with 20852, 10 with 20482 | OVAL_CHICK  (aa1-386)  G1MYK6_MELGA  (aa1-386) | 87.0  86.3 | 9.3e-142  9.3e-142 | 42 | a,b,c | 8106 | 23.36 |  |
| **21651**  (aa16-133)  (aa17-133)  **20491** | 40S ribosomal protein S20 **^2^** | F1NH93_CHICK  (aa4-121)  G1NET0_MELGA  (aa1-117) | 100.0  99.1 | 3.9e-49  2.6e-48 | 2 | a,b,c | 4 | <0.01 | **<0.01** |
| **20527**  (aa40-143) | Peptidyl-prolyl cis-trans isomerase (PPIC) **^1^**; shares 1 peptide with 18232 | F1NZW7_CHICK  (aa126-229) | 99.0 | 1.1e-44 | 1 | a | 2 | <0.01 | **<0.01** |
| **2053**  (aa1-781)  (aa1-776) | Beta-catenin (chBcat) **^2^** | O42486_CHICK  (aa1-781)  G1NGP2_MELGA  (aa1-776) | 100.0  99.6 | 0e0  0e0 | 6 | a,b,c | 13 | <0.01 | **<0.01** |
| **20532**  (aa1-142)  (aa1-69) | NHL repeat-containing protein 3 (NHLRC3) **^1,2^** | F1P590_CHICK  (aa198-339)  G1NQ87_MELGA  (aa201-269) | 97.2  94.2 | 5.8e-63  3.3e-27 | 3 | a,b,c | 12 | 0.01 | **0.01** |
| **20553**  (aa1-143)  (aa1-143) | TIMP-3 (TIMP3) **^1,2,4^** | R4GFR4_CHICK  (aa29-171)  G1NJF8_MELGA  (aa70-212) | 100.0  100.0 | 1.6e-72  2.1e-72 | 8 | a,b,c | 19 | 0.01 | **0.01** |
| **2075**  (aa1-780)  (aa1-780) | Heat shock protein 90kDa beta (Cytosolic), class B member 1 (HSP90AB1) **^1,2^**, C7G498, C7G493; shares 3 peptides with C7G492 (HSP90_alpha) and 3 with 1971 | G1NEA9_MELGA  (aa1-725)  F1NC33_CHICK  (aa1-725) | 91.4  90.9 | 2.9e-107  4.4e-107 | 12 | b,c | 16 | <0.01 | **<0.01** |
| **2080**  (aa1-778)  (aa1-778) | Gelsolin (GSN)**^1,2; t^**; shares 20 peptides with 3462 | GELS_CHICK  (aa1-778)  G1N751_MELGA  (aa1-778) | 100.0  98.3 | 0e0  0e0 | 6 | a,b,c | 103 | 0.04 | **0.07** |
| **3462**  (aa1-611)  (aa1-611) | Gelsolin; shares 20 peptides with 2080 | GELS_CHICK  (aa49-778)  G1N751_MELGA  (aa49-778) | 82.3  82.5 | 0e0  0e0 | 27 | a,b,c | 228 | 0.08 |  |
| **20862**  (aa1-138) | Similar to Golgi membrane protein 1 (GOLM1) **^1^** | E1C3N9_CHICK  (aa192-329) | 78.3 | 2.5e-34 | 3 | a,b,c | 13 | <0.01 | **<0.01** |
| **20902**  (aa7-139)  (aa7-139) | Uncharacterized/similar to renin receptor (ATP6AP2) **^1,2,4^** | G3US21_MELGA  (aa3-178)  F1NUL3_CHICK  (aa3-178) | 67.0  67.7 | 1.8e-24  1.8e-34 | 5 | a,b,c | 39 | 0.03 | **0.03** |
| **22830**  (aa7-123)  (aa7-123) | Uncharacterized/similar to renin receptor (ATP6AP2) | G3US21_MELGA  (aa176-330)  F1NUL3_CHICK  (aa176-330) | 59.0  58.3 | 5.0e-23  2.0e-22 | 4 | a,b,c | 29 | 0.03 |  |
| **21053**  (aa1-127) | Histone H2A (H2A-VIII) **^1,2^** | Q92069_CHICK  (aa1-127) | 100.0 | 1.0e-50 | 5 | a,b,c | 22 | 0.02 | **0.02** |
| **21184**  (aa1-137) | Uncharacterized (LOC769704); domain: carboxylesterase type B | R4GIW9_CHICK  (aa25-161) | 92.7 | 3.5e-50 | 2 | a,b,c | 3 | <0.01 | **<0.01** |
| **21289**  (aa1-136)  (aa1-136) | Histone H3.2 (H3-I) **^2^** | H32_CHICK  (aa1-136)  G1N7D3_MELGA  (aa1-136) | 100.0  100.0 | 6.6e-53  6.6e-53 | 3 | a,b,c | 17 | 0.01 | **0.01** |
| **21338**  (aa23-104)  (aa7-104) | Similar to dickkopf-related protein 3 (DKK3) **^1,2^** | G1N234_MELGA  (aa2-111)  F1NRD7_CHICK  (aa44-169) | 70.9  60.3 | 1.0e-14  2.3e-13 | 2 | a,b,c | 23 | 0.07 | **0.07** |
| **21418**  (aa1-135)  (aa1-135) | Alpha-1,6-mannosylglycoprotein 6-beta-N-acetylglucosaminyltransferase A (MGST5) **^1,2^** | F1NEL5_CHICK  (aa192-326)  G1NII7_MELGA  (aa193-327) | 100.0  100.0 | 1.4e-59  1.4e-59 | 4 | a,b,c | 9 | <0.01 | **<0.01** |
| **21470**  (aa1-135)  (aa2-135) | Galectin (LGALS2) | G1NIW4_MELGA  (aa1-129)  E1C0Z4_CHICK  (aa2-129) | 84.4  86.6 | 7.5e-51  1.3e-50 | 3 | a,c | 4 | <0.01 | **<0.01** |
| **2149**  (aa1-458)  (aa1-458) | Similar to inner centromere protein (INCENP) | G1NC04_MELGA  (aa103-778)  F1NSZ0_CHICK  (aa186-868) | 63.5  62.7 | 2.8e-39  2.9e-38 | 2 | a,b | 4 | <0.01 | **<0.01** |
| **21522**  (aa1-124)  (aa1-124) | Uncharacterized (LMAN2) **^1,2^**; domain: legume_like_lectin | G1MYA6_MELGA  (aa64-187)  R4GG92_CHICK  (aa168-291) | 100.0  100.0 | 6.0e-56  9.1e-56 | 3 | a,b | 7 | <0.01 | **<0.01** |
| **21557**  (aa1-134)  (aa1-134) | Legumain (LGMN) **^1,2^**; shares 4 peptides with 9286 | G1NKC8_MELGA  (aa1-134)  E1C958_CHICK  (aa1-134) | 97.8  98.5 | 1.4e-57  1.6e-57 | 1 | a,b,c | 12 | 0.02 | **0.06** |
| **9286**  (aa6-311)  (aa6-311) | Similar to legumain (LGMN); shares 4 peptides with 21557 | E1C958_CHICK  (aa46-431)  G1NKC8_MELGA  (aa46-3431) | 64.0  63.8 | 8.9e-46  8.9e-46 | 12 | a,b,c | 128 | 0.07 |  |
| **21609**  (aa2-133)  (aa2-133) | Protease-associated domain-containing protein 1 (PRAD1) **^1,2^** | G1NKQ7_MELGA  (aa59-190)  F1NZN0_CHICK  (aa61-192) | 100.0  100.0 | 5.7e-59  5.8e-59 | 2 | a,b,c | 5 | <0.01 | **<0.01** |
| **21682**  (aa7-133)  (aa7-133) | Actin-related protein 2/3 complex subunit 4 (ARPC4) **^1,2^** | F1P010_CHICK  (aa41-167)  G1N8H3_MELGA  (aa41-167) | 100.0  100.0 | 5.4e-52  5.4e-52 | 3 | a,b,c | 11 | <0.01 | **<0.01** |
| **21806**  (aa2-129)  (aa2-129) | Similar to ovocalyxin-36 **^1,2,4; g,t^** | G1N6M8_MELGA  (aa33-163)  Q53HW8_CHICK  (aa33-163) | 72.5  70.2 | 1.0e-38  4.2e-38 | 4 | a,b,c | 454 | 7.34 | **7.80** |
| **21831**  (aa1-131)  (aa1-131) | Cytokine-like protein 1 (CYTL1) **^1^** | G1NJB0_MELGA  (aa7-137)  F1NSV3_CHICK  (aa1-131) | 96.2  95.4 | 7.2e-55  1.4e-54 | 9 | a,b,c | 71 | 0.04 | **0.05** |
| **21858**  (aa1-131) | Vacuolar protein sorting-associated protein 37B (VPS37B) | F1NN84_CHICK  (aa3-135) | 90.2 | 2.4e-39 | 3 | a,b,c | 7 | <0.01 | **<0.01** |
| **21947**  (aa1-131)  (aa1-131) | Peptidyl-prolyl cis-trans isomerase (PPIA) **^3^** | R4GHX2_CHICK  (aa17-147)  G1N1Y0_MELGA  (aa14-144) | 100.0  81.7 | 5.2e-60  1.8e-49 | 5 | a,b,c | 12 | <0.01 | **<0.01** |
| **22023**  (aa1-128) | Similar to semaphorin-3B (SEMA3B) **^2,3^** | H9L2E3_CHICK  (aa278-421) | 70.1 | 3.2e-28 | 5 | a,b,c | 15 | 0.01 | **0.01** |
| **22094**  (aa1-128)  (aa1-128) | Myelin protein zero-like 1 (MPZL1) **^3^** | G1NNN7_MELGA  (aa12-139)  E1C603_CHICK  (aa12-138) | 93.8  93.8 | 1.3e-49  7.5e-49 | 3 | a,c | 8 | 0.01 | **0.01** |
| **22183** | Uncharacterized/Alpha-1,6-mannosyl-glycoprotein 2-beta-N-acetylglucosaminyltransferase |  |  |  | 4 | a,b,c | 21 | 0.01 | **0.01** |
| **22360**  (aa1-127)  (aa1-127) | Protein FAM154B **^3^** | F1NE52_CHICK  (aa15-141)  G1N7G1_MELGA  (aa15-140) | 97.6  95.3 | 1.5e-53  1.2e-52 | 2 | a,b,c | 3 | <0.01 | **<0.01** |
| **8378**  (aa9-336)  (aa9-336) | FAM154B | F1NE52_CHICK  (aa141-468)  G1N7G1_MELGA  (aa140-467) | 92.7  90.9 | 1.5e-131  2.2e-129 | 4 | a,b,c | 6 | <0.01 |  |
| **22382**  (aa1-127)  (aa1-127) | Protein FAM132A **^2^** | F1NFM9_CHICK  (aa136-262)  G1MVZ6_MELGA  (136-262) | 100.0  100.0 | 4.4e-55  4.4e-55 | 3 | a,b,c | 5 | <0.01 | **0.01** |
| **22389**  (aa1-127)  (aa1-127) | Calcium-transporting ATPase (ATP2B2) **^1,4^**; shares 2 peptides with 2758 (ATP2B4) | G3UQV8_MELGA  (aa68-194)  F1NFC7_CHICK  (aa134-260) | 100.0  100.0 | 3.2e-49  2.4e-48 | 2 | a,b,c | 15 | 0.01 | **0.02** |
| **2239**  (aa27-753)  (aa19-753) | Amyloid-beta-like protein (APLP2) **^1,2^**; shares 1 peptide with 2256 and 7717 | F1P0A7_CHICK  (aa1-727)  G1MQW1_MELGA  (aa1-740) | 99.9  96.9 | 0e0  0e0 | 3 | a,b,c | 8 | <0.01 | **<0.01** |
| **22422**  (aa1-116)  (aa1-116) | Complement C3 (C3) **^1,2^** | H9KZT6_CHICK  (aa180-299)  G1MPR2_MELGA  (aa294-413) | 91.7  90.0 | 7.5e-45  2.3e-44 | 2 | a,b | 5 | <0.01 | **<0.01** |
| **22431**  (aa2-126)  (aa2-126) | L-lactate dehydrogenase B chain **^1,2^**; shares 1 peptide with 18757 (LDHA) | G1NLS0_MELGA  (aa210-334)  LDHB_CHICK  (aa309-333) | 88.8  87.2 | 1.6e-46  1.1e-44 | 4 | a,b,c | 17 | 0.01 | **0.01** |
| **2256**  (aa1-751)  (aa17-751) | Beta-amyloid protein 751 isoform (APP) **^1,2,4^**; shares 9 peptides with 7717 | Q9DGJ7_CHICK  (aa1-751)  G1NNT9_MELGA  (aa1-735) | 100.0  99.0 | 0e0  0e0 | 16 | a,b,c | 88 | 0.02 | **0.02** |
| **2257**  (aa1-751)  (aa1-751) | Semaphorin-3C (SEMA3C) **^1,2,4; t^**; shares 18 peptides with 4229 | SEM3C_CHICK  (aa1-751)  G3UPZ0_MELGA  (aa1-751) | 100.0  98.1 | 0e0  0e0 | 31 | a,b,c | 262 | 0.07 | **0.04** |
| **4229**  (aa26-541)  (aa26-541) | Semaphorin-3C (SEMA3C); shares 18 peptides with 2257; SEM3C_CHICK aa180-751; 81.8%; 1.2e-118 | F1P3L3_CHICK  (aa180-751)  G3UPZ0_MELGA  (aa180-751) | 82.2  82.3 | 2.2e-119  2.2e-119 | 1 | a | 1 | <0.01 |  |
| **2267**  (aa1-749)  (aa9-749) | Disintegrin metalloprotease ADAM10 **^1,2^**; shares 4 peptides with 4960 | Q8QFX0_CHICK  (aa1-749)  G1N1F6_MELGA  (aa9-749) | 100.0  98.1 | 0e0  0e0 | 6 | a,b,c | 13 | <0.01 | **<0.01** |
| **4960a**  (aa1-360)  (aa1-360) | Similar to disintegrin and metalloproteinase domain-containing protein 10 (ADAM10); shares 4 peptides with 2267 | G3URD4_MELGA  (aa93-659)  F1P1J6_CHICK  (aa92-658) | 63.5  63.1 | 4.8e-92  1.2e-91 | 1 | a,b,c | 4 | <0.01 |  |
| **227**  (aa1-1792)  (aa6-1792) | Laminin beta 2-like chain (LAMB2) **^1^**; shares 2 peptides with 10417 | O57484_CHICK  (aa1-1792)  G1N7Q3_MELGA  (aa1-1790) | 100.0  91.8 | 0e0  0e0 | 1 | a,c | 2 | <0.01 | **<0.01** |
| **22926**  (aa11-122)  (aa11-122) | Nucleoside diphosphate kinase (NME3) **^2^** | G1NBU7_MELGA  (aa62-173)  R4GM98_CHICK  (aa78-189) | 92.0  92.0 | 2.1e-45  4.0e-45 | 4 | a,b,c | 9 | <0.01 | **<0.01** |
| **2299**  (aa1-743)  (aa177-597) | Sulfhydryl oxidase 1 (QSOX1) **^1,2; i^**; shares 7 peptides with 5467 | QSOX1_CHICK  (aa1-743)  G1MV84_MELGA  (aa1-420) | 100.0  91.1 | 0e0  1.6e-146 | 7 | a,b,c | 238 | 0.01 | **0.18** |
| **5467**  (aa15-451)  (aa15-311) | Similar to sulfhydryl oxidase (QSOX1); shares 7 peptides with 2299; QS0X1_CHICK aa213-742; 77.0%; 8.8e-138 | F1NYK2_CHICK  (aa115-644)  G1MV84_MELGA  (aa37-419) | 77.0  68.9 | 6.4e-139  2.3e-76 | 17 | a,b,c | 488 | 0.38 |  |
| **23012**  (aa1-122)  (aa1-122) | Similar to ribosomal protein S7 **^1^** | G1NMH4_MELGA  (aa1-194)  F1NN16_CHICK  (aa3-196) | 62.9  62.9 | 7.2e-37  7.3e-37 | 3 | a,b | 3 | <0.01 | **<0.01** |
| **23058**  (aa1-121)  (aa1-121) | Programmed cell death protein 10  (PDCD10) **^2^** | G1NDA4_MELGA  (aa92-212)  PDCD10_CHICK  (aa92-212) | 100.0  100.0 | 6.6e-42  6.6e-42 | 2 | a,c | 2 | <0.01 | **<0.01** |
| **24762**  (aa1-108)  (aa1-108) | Similar to programmed cell death protein 10 | G1NDA4_MELGA  (aa44-186)  PDC10_CHICK  (aa44-186) | 66.4  66.4 | 3.1e-16  3.1e-16 | 2 | a,b,c | 4 | <0.01 |  |
| **2308b**  (aa606-700)  (aa618-700) | 40S ribosomal protein S13 **^1,2^** | RS13_CHICK  (aa13-107)  G1N4X8_MELGA  (aa1-83) | 90.5  100.0 | 2.7e-27  6.5e-27 | 3 | a,b | 4 | <0.01 | **<0.01** |
| **23208**  (aa7-50) | Uncharacterized; domain: proteinase_Inhibitor_I1 (Kunitz metazoan; aa62-115) | F1NPR2_CHICK  (aa72-116)  LOC771972 (X3)  (aa178-310) | 60.0  46.% | 6.6e-5  1.0e-20 | 12 | a,b,c | 520 | 3.08 | **3.47** |
| **23291**  (aa1-115)  (aa1-115) | Ras-related protein Rap-1A (RAP1A) **^1^** | F1NSA8_CHICK  (aa5-119)  G1MZG0_MELGA  (aa1-115) | 96.5  96.6 | 2.6e-39  3.0e-39 | 3 | a,b,c | 14 | 0.01 | **0.01** |
| **23368**  (aa1-119)  (aa1-119) | Lymphocyte antigen 86 (LY86) **^1,2,4^** | F1P4F3_CHICK  (aa42-160)  G1MXR9_MELGA  (aa42-160) | 94.1  92.4 | 1.9e-49  1.7e-48 | 7 | a,b,c | 19 | <0.01 | **<0.01** |
| **23513**  (aa1-118)  (aa1-118) | Putative ATPase H+ transporting V1 subunit G isoform 1 (ATP6V1G1) | G1MVG5_MELGA  (aa1-118)  E1C3C8_CHICK  (aa1-118) | 99.2  99.2 | 1.3e-32  1.3e-32 | 3 | a,b,c | 3 | <0.01 | **<0.01** |
| **2353**  (aa21-662)  (aa21-727) | Leucine-rich repeats and immunoglobulin-like domains protein 1 (LRIG1) | E1C726_CHICK  (aa50-848)  G1NAM0_MELGA  (aa1-968) | 79.3  71.9 | 4.8e-109  8.3e-109 | 3 | a,b,c | 6 | <0.01 | **<0.01** |
| **23558**  (aa12-114)  (aa14-114) | Annexin (ANXA4) **^1,2^**; shares 1 peptide with 3812 and 12206 | H9KZI4_CHICK  (aa57-159)  H9H0B3_MELGA  (aa1-101 | 93.2  93.1 | 1.6e-36  5.0e-36 | 3 | a,b | 3 | <0.01 | **<0.01** |
| **23606**  (aa11-117)  (aa11-117) | Glutathione S-transferase theta-1 (GSTT1L); shares 1 peptide with 24638 | E1BUB6_CHICK  (aa144-268)  G1N6L1_MELGA  (aa128-244) | 92.3  91.5 | 9.3e-42  5.4e-41 | 1 | a,b,c | 5 | <0.01 | **<0.01** |
| **24638**  (aa1-109)  (aa1-109) | Glutathione S-transferase theta-1 (GSTT1L); shares 1 peptide with 23606 | E1BUB6_CHICK  (aa86-194)  G1N6L1_MELGA  (aa70-178) | 90.8  89.9 | 2.1e-42  3.9e-41 | 3 | a,b,c | 4 | <0.01 |  |
| **8367c**  (aa251-336)  (aa271-336) | Glutathione_S-transferase (GSTT1L) | E1BUB6_CHICK  (aa2-84)  G1N6L1_MELGA  (aa2-68) | 81.4  92.5 | 6.3e-17  3.6e-15 | 1 | a,c | 3 | <0.01 |  |
| **23618**  (aa1-117)  (aa1-95) | Similar to annexin (ANXA1) **^1,2,4^** | Q6QAZ9_CHICK  (aa86-342)  G1N2L1_MELGA  (aa86-161) | 44.0  53.7 | 2.6e-19  4.5e-9 | 6 | a,b,c | 19 | 0.01 | **0.01** |
| **23771**  (aa40-116)  (aa42-116) | T-complex protein 1 subunit theta (CCT8) ^2^ | TCPQ_CHICK  (aa1-77)  G1NNU8_MELGA  (aa3-378) | 100.0  85.5 | 5.7e-28  2.1e-21 | 2 | a | 2 | <0.01 | **<0.01** |
| **7146**  (aa1-356)  (aa1-356) | T-complex protein 1 subunit theta (CCT8);  TCPQ_CHICK aa128-525; 56.4%; 1.5e-65 | G1NNU8_MELGA  (aa129-526)  F1NEF2_CHICK  (aa129-526) | 86.9  86.7 | 3.7e-66  6.7e-66 | 4 | a,b,c | 7 | <0.01 |  |
| **23848**  (aa1-115)  (aa1-115) | Prepronociceptin (PNOC) | G1NMY3_MELGA  (aa80-194)  E1BRK8_CHICK  (aa80-194) | 100.0  100.0 | 1.7e-46  1.7e-46 | 3 | a,b,c | 11 | <0.01 | **<0.01** |
| **2406**  (aa1-730)  (aa1-730) | Procollagen-lysine,2-oxoglutarate 5-dioxygenase 1 (PLOD1) **^1,2^**; shares 32 peptides with 2595 | PLOD1_CHICK  (aa1-730)  G1N380_MELGA  (aa1-730) | 100.0  99.0 | 0e0  0e0 | 4 | a,b,c | 56 | 0.03 | **0.08** |
| **2595**  (aa1-707)  (aa1-707) | Procollagen-lysine,2-oxoglutarate 5-dioxygenase 1; shares 32 peptides with 2406 | PLOD1_CHICK  (aa1-730)  G1N380_MELGA  (aa1-730) | 92.0  91.8 | 0e0  0e0 | 37 | a,b,c | 421 | 0.12 |  |
| **24219**  (aa27-112)  (aa1-112) | Epididymal secretory protein E1/Stathmin-3 (NPC2) **^1,2^** | G3USI2_MELGA  (aa37-122)  F1N9N4_CHICK  (aa1-148) | 98.8  74.3 | 5.9e-37  7.3e-37 | 5 | a,b,c | 16 | 0.01 | **0.02** |
| **25725**  (aa1-99)  (aa2-99)  **24220** | 40S ribosomal protein S25 **^1,2^** | F1NU56_CHICK  (aa1-105)  G1MYC4_MELGA  (aa16-119) | 93.3  93.3 | 1.2e-34  4.3e-34 | 3 | a,b,c | 6 | <0.01 | **<0.01** |
| **24235**  (aa1-112)  (aa1-112) | Ganglioside GM2 activator (RCJMB04_14a17) **^1,2,4^** | Q5ZK02_CHICK  (aa7-118)  G1N3B0_MELGA  (aa59-170) | 92.9  91.1 | 3.4e-47  2.8e-46 | 4 | a,b,c | 21 | 0.09 | **0.10** |
| **24254**  (aa17-112)  (aa17-112) | Profilin-2 (PFN2) **^3^** | F1NUH4_CHICK  (aa1-96)  G1NEP2_MELGA  (aa1-96) | 100.0  100.0 | 2.1e-41  2.1e-41 | 2 | a,b,c | 5 | <0.01 | **<0.01** |
| **24376**  (aa2-69)  (aa2-72) | Actin, cytoplasmic (ACTA1) **^1,4^**; shares 1 peptide with other actins | ACTS_CHICK  (aa260-330)  G1N059_MELGA  (aa159-332) | 90.1  86.5 | 1.1e-20  9.5e-21 | 2 | a | 4 | 0.07 | **0.07** |
| **24420**  (aa1-111)  (aa1-111) | Synthenin-1 (SDCBP) **^1,2; t^** | G1NEY6_MELGA  (aa90-200)  Q5ZHM8_CHICK  (aa78-188) | 96.4  95.5 | 1.7e-41  1.9e-41 | 4 | a,b,c | 41 | 0.09 | **0.10** |
| **24585**  (aa1-109)  (aa1-109) | Calbindin (CALB1) **^1,2,4^** | CALB1_CHICK  (aa154-262)  G1NH64_MELGA  (aa147-258) | 100.0  97.3 | 3.0e-41  4.1e-40 | 7 | a,b,c | 52 | 0.02 | **0.03** |
| **24618**  (aa1-109)  (aa1-109**)** | Parvalbumin, thymic (LOC100545341); shares 1 peptide with 20161 | PRVT_CHICK  (aa1-109)  G1MRW5_MELGA  (aa1-109) | 90.8  90.8 | 6.6e-36  6.6e-36 | 3 | a,b,c | 5 | <0.01 | **<0.01** |
| **24733**  (aa1-78)  (aa1-78) | Ras-related protein Rab-8B (RAB8B) **^2^** | G1MYD7_MELGA  (aa65-142)  E1C080_CHICK  (aa83-160) | 100.0  100.0 | 3.9e-27  4.3e-27 | 2 | a,c | 5 | <0.01 | **<0.01** |
| **24798**  (aa1-106)  (aa1-106) | Acid sphingomyelinase-like phosphodiesterase 3b (SMPDL3B) **^2,3^** | F1N9C4_CHICK  (aa127-232)  G1MR04_MELGA  (aa34-139) | 97.2  93.4 | 5.8e-47  3.6e-45 | 2 | a,b | 4 | <0.01 | **<0.01** |
| **24818**  (aa1-108)  (aa1-108) | F-actin capping protein subunit beta (CAPZB) **^1,2^** | G1N1G2_MELGA  (aa11-218)  CAPZB_CHICK  (aa111-218) | 100.0  100.0 | 6.6e-49  6.8e-49 | 2 | a,b,c | 7 | <0.01 | **<0.01** |
| **4111**  (aa1-554)  (aa1-553)  **2489** | ADAM metallopeptidase with thrombospondin type 1 motif (ADAMTS1)  **^2,4^** | F1P3T6_CHICK  (aa208-919)  G1NNU1_MELGA  (aa28-738) | 77.4  77.5 | 1.2e-157  1.3e-157 | 9 | a,b,c | 13 | <0.01 | **<0.01** |
| **24897**  (aa1-93)  (aa1-93) | Transforming protein RhoA (RCJMB0_13j3) **^1,2^**; shares 3 peptides with 12114 (RhoC) | G3UU71_MELGA  (aa1-93)  Q5ZK30_CHICK  (aa1-93) | 100.0  100.0 | 2.9e-36  2.9e-36 | 1 | a,c | 4 | <0.01 | **<0.01** |
| **25266**  (aa2-104)  (aa2-104) | Histone H4 (H4-I) **^1,2^** | H4_CHICK  (aa1-103)  G1NRJ0_MELGA  (aa1-103) | 100.0  100.0 | 1.1e-38  1.1e-38 | 8 | a,b,c | 40 | 0.03 | **0.03** |
| **25336**  (aa1-103)  (aa1-104) | Similar to Ig lambda chain C region (LOC100545541) **^2^** | LAC_CHICK  (aa1-103)  G3UR71_MELGA  (aa115-217) | 72.8  74.0 | 3.6e-35  2.2e-33 | 5 | a,b,c | 27 | 0.01 | **0.01** |
| **2565b**  (aa352-706)  (aa350-706) | Similar to N-acetylgalactosamine-6-sulfatase (GALNS) **^2^**; shares 2 peptides with 9921 | G1N6S6_MELGA  (aa21-496)  F1NW57_CHICK  (aa20-493) | 66.2  67.3 | 1.9e-58  2.3e-58 | 3 | a,b,c | 6 | <0.01 | **<0.01** |
| **9921**  (aa1-291)  (aa1-291) | N-acetylgalactosamine-6-sulfatase  (GALS); shares 2 peptides with 2565b | F1NW57_CHICK  (aa60-381)  G1N6S6_MELGA  (aa63-384) | 80.2  80.2 | 3.7e-105  4.4e-105 | 1 | a,c | 3 | <0.01 |  |
| **2574a**  (aa1-349)  (aa1-349) | Apolipoprotein AIV (APOA4) **^1,2^**; shares 11 peptides with 4336a | G1MVV8_MELGA  (aa1-349)  O93601_CHICK  (aa1-349) | 92.0  90.0 | 4.7e-78  1.6e-77 | 19 | a,b,c | 49 | 0.01 | **0.01** |
| **4336a**  (aa1-244)  (aa2-244) | Apolipoprotein A-IV (APOA4); shares 11 peptides with 2574a | G1MVV8_MELGA  (aa69-349)  O93601_CHICK  (aa69-349) | 79.8  78.3 | 1.7e-35  4.7e-35 | 1 | a | 3 | <0.01 |  |
| **25766**  (aa2-101)  (aa1-101) | Protein S100-A11 (S100A11) **^1,2^** | H9H0X9_MELGA  (aa2-101)  S10AB_CHICK  (aa1-102) | 94.0  92.1 | 6.2e-39  5.0e-39 | 2 | a,b,c | 4 | <0.01 | **<0.01** |
| **25891**  (aa1-68)  (aa1-68) | Hemoglobin subunit alpha-D (HBAD) **^3; g^**; shares 1 peptide with P30892 | HBAD_MELGA  (aa32-99)  HBAD_CHICK  (aa32-99) | 94.1  92.6 | 9.6e-22  1.8e-21 | 1 | a | 1 | <0.01 | **0.01** |
| **P30892**  (aa1-141)  (aa1-141) | Hemoglobin subunit alpha-D; shares 1 peptide with 25891 | HBAD_CHICK  (aa1-141  HBAD_MELGA  (aa1-141) | 90.8  90.1 | 4.7e-53  7.8e-53 | 3 | a,c | 5 | <0.01 |  |
| **25910**  (aa1-94)  (aa1-94) | Alpha 2,3-sialyltransferase (ST3GAL-VI) **^1,2^** | G3URG3_MELGA  (aa113-206)  Q702H9_CHICK  (aa113-206) | 100.0  100.0 | 2.6e-40  2.6e-40 | 2 | a,b,c | 3 | <0.01 | **<0.01** |
| **25983**  (aa1-87)  (aa1-87) | Laminin subunit alpha-4 (LAMA4) | F1NSZ5_CHICK  (aa275-361)  G3UV07_MELGA  (aa127-213) | 96.6  93.1 | 4.0e-31  3.4e-29 | 1 | a,b | 5 | <0.01 | **<0.01** |
| **4923**  (aa1-488)  (aa1-488) | Laminin subunit alpha-4 (LAMA4) | G1NL15_MELGA  (aa464-983)  F1NSZ5_CHICK  (aa1190-1709) | 86.5  88.2 | 4.3e-116  1.2e-115 | 3 | a,b,c | 6 | <0.01 |  |
| **2599**  (aa1-704)  (aa1-704) | Fibulin-1 (FBLN1) **^1,2^**; shares 13 peptides with 5469 and 7 with 5029 | FBLN1_CHICK  (aa1-704)  G1NM42_MELGA  (aa3-685) | 100.0  87.6 | 0e0  0e0 | 23 | a,b,c | 126 | 0.04 | **0.03** |
| **5029**  (aa1-478)  (aa92-344) | Similar to fibulin-1; shares 7 peptides with 2599 and 7 with 5469 | FBLN1_CHICK  (aa218-659)  G3UR54_MELGA  (aa1-335) | 63.8  74.9 | 5.3e-79  1.4e-66 | 5 | a,b,c | 41 | 0.02 |  |
| **5469**  (aa16-451)  (aa16-451) | Similar tof-1; shares 13 peptides with 2599 and 7 with 5029 | FBLN1_CHICK  (aa60-659)  G1NM42_MELGA  (aa36-640) | 71.8  66.4 | 3.9e-65  5.5e-51 | 2 | a,b,c | 27 | 0.01 |  |
| **26053** | Uncharacterized/similar to Ig epsilon chain C region |  |  |  | 6 | a,b,c | 32 | 0.03 | **0.03** |
| **26068** | 14-3-3 protein theta (YWHAQ) **^1,2^**; shares 3 peptides with 20781 and several other 14-3-3 proteins | G1NMK1_MELGA  (aa1-194)  1433T_CHICK  (aa1-194) | 100.0  100.0 | 2.4e-78  2.4e-78 | 2 | a,b,c | 10 | <0.01 | **<0.01** |
| **26318**  (aa1-96)  (aa1-96) | 14-3-3 protein theta | 1433T_CHICK  (aa99-194)  G1NMK1_MELGA  (aa99-194) | 100.0  100.0 | 1.1e-41  1.1e-41 | 3 | a,b,c | 7 | <0.01 |  |
| **26159**  (aa1-97)  (aa1-97) | Superoxide dismutase [Cu-Zn] (SOD1) **^3^** | F1N8Q1_CHICK  (aa58-154)  G1NNV7_MELGA  (aa38-134) | 100.0  99.0 | 1.4e-42  3.5e-42 | 2 | a,c | 3 | <0.01 | **<0.01** |
| **26235**  (aa1-97)  (aa1-97) | Neuroserpin (SERPINI1) **^1,2^**; shares 3 peptides with 9774 | NEUS_CHICK  (aa230-326)  G1ND93_MELGA  (aa238-334) | 100.0  99.0 | 2.2e-37  7.8e-37 | 5 | a,b,c | 33 | 0.02 | **0.02** |
| **9774**  (aa1-299)  (aa7-299) | Neuroserpin; shares3 peptides with 26235 | G1ND93_MELGA  (aa3-301)  NEUS_CHICK  (aa1-293) | 96.7  99.3 | 6.9e-126  1.8e-125 | 8 | a,b,c | 22 | 0.02 |  |
| **26348**  (aa1-96)  (aa1-96) | Multiple coagulation factor deficiency protein 2 (MCFD2) **^4^** | Q5ZJB5_CHICK  (aa48-143)  G1ND62_MELGA  (aa48-143) | 100.0  100.0 | 1.2e-36  1.2e-36 | 3 | a,c | 4 | <0.01 | **<0.01** |
| **2636**  (aa23-662)  (aa23-662) | Similar to beta-1,4-N-acetylgalactosaminyltransferase 3 (B4GALNT3) **^2^** | F1NN69_CHICK  (aa117-932)  G1NL13_MELGA  (aa93-892) | 74.9  76.4 | 2.7e-154  8.3e-154 | 7 | a,b,c | 24 | <0.01 | **<0.01** |
| **26413**  (aa14-74)  (aa14-74) | Alpha-enolase (ENOA/ENO1) **^1,2^**; ENOA_CHICK aa1-61; 95.1%; 4.5e-20 | Q5ZIQ4_CHICK  (aa1-61)  G1MX98_MELGA  (aa1-63) | 95.1  92.1 | 1.0e-20  2.6e-19 | 2 | a,b | 9 | 0.01 | **0.02** |
| **7773**  (aa2-354)  (aa2-354) | Alpha-enolase (ENO1); ENOA_CHICK aa82-434; 96.0%; 1.7e-137 | G1MX98_MELGA  (aa84-436)  F1NZ78_CHICK  (aa82-434) | 96.6  96.3 | 8.1e-139  1.5e-138 | 16 | a,b,c | 55 | 0.01 |  |
| **26477**  (aa1-81)  (aa1-81)  **9358** | Toll-interacting protein (TOLLIP) | F1P006_CHICK  (aa123-203)  G1N8W8_MELGA  (aa123-203) | 100.0  100.0 | 4.1e-34  4.1e-34 | 2 | a,b,c | 5 | <0.01 | **<0.01** |
| **26558**  (aa35-94) | Gamma-glutamyl hydrolase (GGH) **^1,2^** | F1NR48_CHICK  (aa40-99) | 88.3 | 3.8e-19 | 2 | a,b,c | 8 | 0.02 | **0.02** |
| **26666**  (aa1-94)  (aa4-94) | Tubulin beta-1/2 chain (TBB1B/TBB2B) **^1,2^** | TBB1_CHICK  (aa1-94)  G3X8P3_MELGA  (aa1-91) | 98.9  98.9 | 1.6e-40  5.6e-39 | 4 | a,b,c | 21 | 0.02 | **0.02** |
| **26683**  (aa1-93) | Transgelin-2 | R4GJ67_CHICK  (aa137-229) | 98.9 | 1.8e-39 | 3 | a,b,c | 6 | <0.01 | **<0.01** |
| **267**  (aa4-1706)  (aa4-1706) | Protocadherin Fat 4 (FAT4) | F1NLP0_CHICK  (aa42-1744)  G1NDK9_MELGA  (aa20-1722) | 98.3  98.3 | 0e0  0e0 | 3 | a,b | 3 | <0.01 | **<0.01** |
| **4597**  (aa1-512)  (aa1-512) | Protocadherin Fat 4; shares 1 peptide with 60 | F1NLP0_CHICK  (aa1789-2300)  G1NDK9_MELGA  (aa1767-2266) | 97.1  93.8 | 3.0e-197  3.5e-162 | 4 | a,b,c | 7 | <0.01 |  |
| **60**  (aa15-2630)  (aa15-2630) | Protocadherin Fat 4; shares 1 peptide with 4597 | F1NLP0_CHICK  (aa1743-5005)  G1NDK9_MELGA  (aa1721-4970) | 77.8  77.7 | 0e0  0e0 | 1 | a | 1 | <0.01 |  |
| **26729**  (aa2-93)  (aa2-93) | 60S ribosomal protein L22 | G1MRM5_MELGA  (aa36-127)  F1N9J4_CHICK  (aa36-127) | 97.8  97.8 | 1.2e-34  1.2e-34 | 2 | a,b,c | 3 | <0.01 | **<0.01** |
| **26854**  (aa1-92)  (aa1-92) | Similar to platelet-derived growth factor (PDGF) **^2^** | PDGFC_CHICK  (aa41-140)  G1MS78_MELGA  (aa43-142) | 72.0  72.0 | 1.2e-27  1.3e-27 | 3 | a,b,c | 5 | <0.01 | **<0.01** |
| **2706a**  (aa1-147)  (aa1-169) | Uncharacterized/similar to tubulin polymerization-promoting protein family member (TPPP3) **^3^** | E1BR14_CHICK  (aa7-153)  G1MSS7_MELGA  (aa48-216) | 99.3  68.0 | 5.4e-56  8.6e-45 | 7 | a,b,c | 13 | <0.01 | **<0.01** |
| **27206**  (aa1-89)  (aa1-89) | Programmed cell death protein 6 (PDCD6) **^1,2^** | G1N6J5_MELGA  (aa38-126)  F1NHD8_CHICK  (aa67-155) | 97.8  97.8 | 5.5e-36  6.6e-36 | 6 | a,b,c | 15 | 0.01 | **0.01** |
| **27451**  (aa1-87) | Arylsulfatase (ARSB) | F1P099_CHICK  (aa442-528) | 94.3 | 1.2e-38 | 3 | a,b | 8 | 0.02 | **0.02** |
| **2749**  (aa59-685)  (aa59-685) | Similar to ceruloplasmin (CP) **^2,3^** | G1NEW3_MELGA  (aa134-1006)  F1N9R5_CHICK  (aa134-1006) | 62.8  62.2 | 1.2e-96  3.2e-96 | 2 | a,c | 3 | <0.01 | **<0.01** |
| **2758**  (aa6-642)  (aa6-642) | Similar to calcium-transporting ATPase (ATP2B4) **^3,4^**; shares 4 peptides with 678 (ATP2B1), and 2 with 22389 (ATP2B2) | E1C784_CHICK  (aa218-1166)  G1NAR6_MELGA  (aa218-1169) | 55.0  52.3 | 6.5e-117  7.5e-117 | 12 | a,b,c | 41 | <0.01 | **<0.01** |
| **27658**  (aa1-85)  (aa1-85) | Visinin-like protein 1 (VSNL1) **^1^** | VISL1_CHICK  (aa107-191)  G1NMM0_MELGA  (aa107-191) | 100.0  100.0 | 3.8e-30  3.8e-30 | 4 | a,b,c | 8 | <0.01 | **<0.01** |
| **27687**  (aa1-74) | Signal peptidase complex subunit 3 (SPC22) | SPCS3_CHICK  (aa1-74) | 97.3 | 3.7e-28 | 2 | a,b,c | 3 | <0.01 | **<0.01** |
| **27739**  (aa1-83)  (aa1-83) | Galactocerebrosidase (GALC) **^1,2^** | G1NJZ8_MELGA  (aa63-145)  F1NJ89_CHICK  (aa63-145) | 100.0  100.0 | 4.7e-37  4.8e-37 | 2 | a,c | 4 | <0.01 | **0.01** |
| **2781**  (aa1-684)  (aa1-684) | Chondroadherin-like protein (CHADL) **^2^** | G1NHZ2_MELGA  (aa55-736)  F1NHD7_CHICK  (aa52-733) | 92.0  90.9 | 0e0  0e0 | 4 | a,c | 8 | <0.01 | **<0.01** |
| **27816**  (aa1-84)  (aa1-84) | Malate dehydrogenase (MDH2) **^1,2^** | E1BVT3_CHICK  (aa22-105)  G1MXB6_MELGA  (aa22-105) | 97.6  96.4 | 1.9e-31  3.2e-31 | 2 | a,b | 4 | <0.01 | **<0.01** |
| **2798**  (aa36-600)  (aa36-600) | Uncharacterized; domains: PLAT/LH2, cytokine_IL1_like (LOC421123) | G1NEP4_MELGA  (aa151-899)  R4GK51_CHICK  (aa166-914) | 70.7  70.8 | 3.8e-105  3.0e-68 | 4 | a,b,c | 9 | <0.01 | **<0.01** |
| **280**  (aa14-1675)  (aa1-1675) | Clathrin heavy chain (1; CLTC1) | F1NW23_CHICK  (aa1-1662)  G3UV43_MELGA  (aa11-1682) | 100.0  89.7 | 0e0  0e0 | 13 | a,b,c | 16 | <0.01 | **0.01** |
| **2800**  (aa1-680)  (aa39-680) | RGD-CAP/Transforming growth factor-beta-induced protein ig-h3 (TGFBI) **^1,2^** | O42390_CHICK  (aa1-680)  G1N7Q8_MELGA  (aa2-642) | 100.0  98.3 | 0e0  0e0 | 19 | a,b,c | 62 | 0.01 | **0.02** |
| **28044**  (aa1-61)  (aa1-61) | Protein CREG1 **^1,2^** | G1NNN9_MELGA  (aa57-117)  CREG1_CHICK  (aa132-192) | 96.7  95.1 | 1.9e-25  7.1e-25 | 4 | a,b,c | 14 | 0.01 | **0.01** |
| **28143**  (aa1-82)  (aa1-82) | SPARC-related modular calcium-binding protein 1 (SMOC1) | G1NGZ7_MELGA  (aa353-434)  F1NY60_CHICK  (aa354-435) | 100.0  100.0 | 1.7e-34  1.8e-34 | 3 | a,b,c | 7 | <0.01 | **<0.01** |
| **28365**  (aa1-80)  (aa1-80) | Ribosomal protein S16 **^1,2^** | R4GGJ0_CHICK  (aa51-146)  G1NEZ2_MELGA  (aa65-160) | 82.3  82.3 | 2.2e-15  2.5e-15 | 2 | a,b,c | 4 | <0.01 | **0.01** |
| **28649**  (aa1-77)  (aa1-77) | Apolipoprotein D (APOD) **^1,2^** | Q5G8Y9_CHICK  (aa113-189)  G1N591_MELGA  (aa116-192) | 88.3  87.0 | 4.8e-32  1.8e-31 | 5 | a,b,c | 45 | 0.21 | **0.27** |
| **28687**  (aa1-59)  (aa1-59) | Ras-related protein Ral-A (RALA) **^2^** | E1C524_CHICK  (aa109-167)  G1NI18_MELGA  (aa109-167) | 100.0  100.0 | 5.9e-19  5.9e-19 | 2 | a,c | 3 | <0.01 | **<0.01** |
| **28703**  (aa1-77)  (aa1-77) | Beta-1,4-galactosyltransferase 4  (B4GALT4) **^2^** | G1NNC1_MELGA  (aa93-169)  E1C9B0_CHICK  (aa94-170) | 93.5  93.5 | 1.2e-31  1.8e-31 | 2 | a,c | 3 | <0.01 | **<0.01** |
| **28736**  (aa2-64) | Similar to protein kinase domain-containing protein, cytoplasmic (PKDCC) | F1NEA9_CHICK  (aa88-150) | 46.0 | 6.9e-6 | 2 | b,c | 5 | <0.01 | **<0.01** |
| **4212**  (aa58-524)  (aa58-524)  **2886** | Similar to annexin (ANXA6) **^1^** | Q6B344_CHICK  (aa66-560)  G1N3Z0_MELGA  (aa66-560) | 70.8  69.7 | 8.1e-40  1.3e-39 | 2 | a,c | 3 | <0.01 | **<0.01** |
| **2895**  (aa1-548) | Myosin (MYH13); shares 7 peptides with 167 | F1NDC6_CHICK  (aa828-1457) | 85.1 | 1.1e-73 | 1 | a | 3 | <0.01 | **<0.01** |
| **29257**  (aa1-43)  (aa1-43) | Avidin (AVD)/avidin-related protein **^1,2; i^** | G1MSZ9_MELGA  (aa98-140)  AVID_CHICK  (aa99-141) | 88.4  88.4 | 4.6e-12  5.5e-12 | 3 | a,b,c | 65 | 1.42 | **1.95** |
| **29338**  (aa1-71)  (aa1-71) | Ribosomal protein S15A **^2,4^** | G1N6V8_MELGA  (aa1-71)  F1NXW3_CHICK  (aa23-93) | 100.0  100.0 | 2.1e-30  2.5e-30 | 3 | a,b,c | 8 | <0.01 | **<0.01** |
| **29768**  (aa1-67) | Similar to EH domain-containing protein 4 or 3 (EHD4/EHD3) **^2^** | F1NGM0_CHICK  (aa1-63) | 65.7 | 1.3e-13 | 4 | a,b,c | 11 | <0.01 | **0.01** |
| **29776**  (aa1-67)  (aa1-67) | Methyltransferase-like protein 9  (METTL9) **^2^** | G1N7V4_MELGA  (aa214-280)  F1NG91_CHICK  (aa246-312) | 100.0  100.0 | 4.8e-30  5.4e-30 | 3 | a,b,c | 4 | <0.01 | **<0.01** |
| **29976**  (aa1-64)  (aa1-64) | Nucleotide exchange factor SIL1 (SIL1) **^1,2^** | G1MRI3_MELGA  (aa160-223)  F1NWG7_CHICK  (aa160-223) | 100.0  100.0 | 1.8e-20  1.8e-20 | 2 | a,b,c | 17 | 0.01 | **0.01** |
| **30**  (aa21-596)  (aa2034-3198)  (aa1161-2027) | Similar to alpha-2-macroglobulin (A2ML1?) **^1,4^** | F1NTK2_CHICK  (aa20-815)  F1NEQ4_CHICK  (aa1-1271)  G1NME9_MELGA  (aa42-1346) | 64.6  63.4  48.9 | 4.6e-82  7.0e-89  5.8e-89 | 35 | a,b,c | 346 | 0.02 | **0.02** |
| **30108**  (aa9-62)  (aa9-62) | Ras-related C3 botulinum toxin substrate 1 (RAC1) **^1,2^**; shares 1 peptide with 979b | F1N8D9_CHICK  (aa84-137)  G1N850_MELGA  (aa85-138) | 98.1  98.1 | 4.5e-21  4.6e-21 | 3 | a,b,c | 15 | 0.01 | **0.01** |
| **30155**  (aa1-61**)** | Ovalbumin-related protein X (OVALX) **^1,2; i^** | R9TNA6_CHICK  (aa5-65) | 90.2 | 1.8e-22 | 2 | a,b,c | 15 | 0.02 | **0.21** |
| **9384**  (aa1-309)  (aa78-309) | Ovalbumin-related protein X; shares peptide with 8047 (Ovalbumin-related protein Y) | R9TNA6_CHICK  (aa94-402)  G1MZH2_MELGA  (aa1-232) | 90.9  88.8 | 4.1e-122  5.8e-87 | 12 | a,b,c | 217 | 0.18 |  |
| **30730**  (aa1-51)  (aa1-51) | Polypeptide N-acetylgalactosaminyltransferase 2 (GALNT2) | G1NHL9_MELGA  (aa37-87)  F1NDK8_CHICK  (aa37-87) | 100.0  100.0 | 3.0e-21  6.7e-21 | 3 | a,b,c | 8 | 0.01 | **0.02** |
| **3078**  (aa1-647)  (aa1-647) | Carboxypeptidase Z (CPZ) | CBPZ_CHICK  (aa1-647)  G1NJR1_MELGA  (aa1-647) | 100.0  97.1 | 0e0  0e0 | 20 | a,b,c | 89 | 0.02 | **0.02** |
| **30796**  (aa5-50)  (aa5-50) | Similar to procollagen C-endopeptidase enhancer (PCOLCE) | G1N443_MELGA  (aa187-232)  F1NH70_CHICK  (aa182-227) | 63.0  63.0 | 8.9e-11  8.8e-11 | 4 | a,b,c | 46 | 0.09 | **0.10** |
| **3144**  (aa3-642)  (aa11-642) | Chondroitin sulfate synthase (CHPF); shares peptide with 4709 (CHPF2) | F1P355_CHICK  (aa27-704)  G1NFF2_MELGA  (aa1-670) | 91.2  91.3 | 4.2e-148  4.7e-148 | 8 | a,b,c | 12 | <0.01 | **<0.01** |
| **3188**  (aa1-634)  (aa1-634) | HSP70**^1^**/HSPA2; B2MV58, A0PA14; shares 10 peptides with 3409, A0PA16 | HSP70_CHICK  (aa1-634)  G1NR17_MELGA  (aa1-634) | 100.0  98.4 | 0e0  0e0 | 1 | b | 1 | <0.01 | **<0.01** |
| **323b**  (aa548-1572)  (aa548-1572) | Protein KIAA1199 **^1,2,4^**; shares 23 peptides with 3694 | G1N7U1_MELGA  (aa2-1341)  F1P1G6_CHICK  (aa27-1360) | 73.3  75.3 | 2.6e-168  7.0e-168 | 59 | a,b,c | 923 | 0.07 | **0.09** |
| **3694**  (aa1-574)  (aa1-574) | KIAA1199; shares 23 peptides with 323b | G1N7U1_MELGA  (aa7-580)  F1P1G6_CHICK  (aa28-601) | 97.4  97.4 | 0e0  0e0 | 5 | a,b,c | 71 | 0.03 |  |
| **3249**  (aa1-628)  (aa1-628) | Plastin-3 (PLS3) **^2^** | Q5ZI39_CHICK  (aa1-628)  G1MUJ6_MELGA  (aa4-631) | 100.0  99.8 | 0e0  0e0 | 7 | a,b,c | 13 | <0.01 | **<0.01** |
| **3308**  (aa356-625)  (aa10-625) | Cytokine receptor-beta/Leukemia inhibitory factor receptor (LIFR) **^1,2^**; shares 2 peptides with 906 | G3UTR6_MELGA  (aa1-270)  F1NN90_CHICK  (aa308-1083) | 97.4  67.0 | 1.2e-110  7.9e-108 | 1 | a | 4 | <0.01 | **<0.01** |
| **906**  (aa1-1083)  (aa814-1083) | Cytokine receptor-beta (LIFR); shares 2 peptides with 3308 | Q8QFQ7_CHICK  (aa1-1083)  G3UTR6_MELGA  (aa1-270) | 100.0  95.9 | 0e0  3.5e-109 | 7 | a,b,c | 17 | <0.01 |  |
| **3436**  (aa1-613) | 78 kDa glucose-regulated protein (HSPA5) **^1,2,4^**; HSP70; A0ZT13, A0PA15 | GRP78_CHICK  (aa40-652) | 100.0 | 1.8e-213 | 28 | a,b,c | 285 | 0.04 | **0.06** |
| **3463**  (aa75-499)  (aa75-494) | Collagen alpha-1(XVII) chain (COL17A1) **^1,2^** | COHA1_CHICK  (aa978-1456)  G1NDW1_MELGA  (aa978-1451) | 85.4  82.9 | 1.7e-80  5.9e-76 | 8 | a,b,c | 46 | 0.02 | **0.02** |
| **3477**  (aa1-607)  (aa1-607) | Prothrombin **^1,2^** | Q91001_CHICK  (aa1-607)  G1NEM6_MELGA  (aa1-607) | 100.0  96.0 | 0e0  0e0 | 17 | a,b,c | 86 | 0.02 | **0.03** |
| **35**  (aa1-3124)  (aa1-3124) | Collagen alpha-1(XII) chain (COL12A1) | COCA1_CHICK  (aa1-3124)  G1NM18_MELGA  (aa1-3119) | 100.0  98.0 | 0e0  0e0 | 13 | a,b,c | 17 | <0.01 | **<0.01** |
| **350**  (aa1-1519)  (aa428-1519) | Slit-3 (SLIT3) **^4^**; shares 1 peptide with13685 (Slit-2) | F1NF14_CHICK  (aa1-1519)  G3UNZ8_MELGA  (aa3-1092) | 98.8  96.6 | 0e0  0e0 | 9 | a,b,c | 11 | <0.01 | **<0.01** |
| **3589**  (aa24-401)  (aa24-426) | Glypican-1 (GPC1) **^1,2,4^** | GPC1_CHICK  (aa106-523)  G1MQ88_MELGA  (aa54-494) | 89.5  84.7 | 5.6e-95  1.8e-93 | 15 | a,b,c | 102 | 0.01 | **0.02** |
| **4420**  (aa1-477)  (aa1-446)  **360** | Uncharacterized (MIA2)/Cutaneous T-cell lymphoma-associated antigen 5 **^1^** | E1C1N7_CHICK  (aa41-517)  G1NIZ7_MELGA  (aa41-489) | 82.4  81.4 | 3.5e-139  3.5e-127 | 2 | a,c | 3 | <0.01 | **<0.01** |
| **3602**  (aa14-599)  (aa28-599) | Protein disulfide-isomerase A4 (RCJMB04_13I7) **^1,2^** | Q5ZK20_CHICK  (aa1-627)  G1MRI5_MELGA  (aa6-612) | 90.0  90.5 | 1.2e-117  3.7e-114 | 5 | a,b,c | 12 | <0.01 | **<0.01** |
| **3606**  (aa48-583)  (aa48-496) | Uncharacterized/ similar to ADAM28 **^1,2^** | F1NU67_CHICK  (aa39-761)  G1MR45_MELGA  (aa38-602) | 54.6  62.4 | 1.2e-58  9.3e-58 | 5 | a,b,c | 10 | <0.01 | **<0.01** |
| **3610**  (aa1-281) | Similar to alpha-mannosidase (MAN2B2) **^1,2^** | G1NJI0_MELGA  (aa46-510) | 44.6 | 2.0e-45 | 13 | a,b,c | 84 | 0.01 | **0.02** |
| **3617b**  (aa289-587)  (aa241-597) | FAM20A (family with sequence similarity 20, member A) **^3^** | G1N536_MELGA  (aa74-392)  E1BU21_CHICK  (aa89-473) | 91.8  81.8 | 1.1e-50  1.2e-50 | 2 | a,b,c | 42 | 0.01 | **0.02** |
| **3635**  (aa1-594)  (aa13-487) | Syntaxin-binding protein 1 (STXBP1) | STXB1_CHICK  (aa1-594)  G1MUZ5_MELGA  (aa18-492) | 100.0  99.4 | 0e0  5.3e-196 | 7 | a,b,c | 11 | <0.01 | **<0.01** |
| **3797**  (aa14-583) | Polypeptide N-acetylgalactosaminyltransferase 4 (GALNT4) **^2^** | G1NRH7_MELGA  (aa1-570) | 98.1 | 0e0 | 15 | a,b,c | 45 | <0.01 | **0.01** |
| **3863**  (aa1-575)  (aa1-575) | Alpha-(1,6)-fucosyltransferase (FUT8) **^1,2^** | Q659W9_CHICK  (aa1-2102)  G1NDR5_MELGA  (aa1-1575) | 100.0  99.1 | 0e0  0e0 | 5 | a,b,c | 7 | <0.01 | **<0.01** |
| **3917**  (aa1-572)  (aa1-572) | Secretogranin-2 (SCG2) **^1,4^** | E1C6L5_CHICK  (aa58-629)  G1NRZ9_MELGA  (aa66-638) | 98.1  97.2 | 4.4e-190  3.2e-187 | 38 | a,b,c | 225 | 0.09 | **0.09** |
| **3922**  (aa1-572)  (aa1-572) | Collagen alpha-1(X) chain (COL10A1) **^1,2; i^**; all peptides in ~aa420-530 (non-triple helical, contains C1q/TNF-like domain) | G1MZ16_MELGA  (aa103-674)  F1NRH2_CHICK  (aa103-674) | 96.5  96.9 | 7.6e-119  2.3e-118 | 7 | a,b,c | 113 | 0.02 | **0.02** |
| **3951**  (aa1-568)  (aa1-568) | 60 kDa heat shock protein, mitochondrial (HSPD1) **^1,2^** | CH60_CHICK  (aa1-573)  G1N5G4_MELGA  (aa1-573) | 97.4  96.7 | 1.1e-195  1.9e-195 | 4 | b,c | 4 | <0.01 | **<0.01** |
| **4071**  (aa111-557)  (aa111-557) | Alpha-tubulin (TUBA1B) **^1^**, shares 15 peptides with other tubulins | G1NFQ5_MELGA  (aa7-455)  F1NXR6_CHICK  (12-458) | 98.7  98.0 | 4.3e-195  5.1e-195 | 21 | a,b,c | 350 | 0.08 | **0.09** |
| **4076a**  (aa11-169)  (aa11-169) | Ras-related protein Rab-3B (RAB3B) | G1NF26_MELGA  (aa3-165)  E1C8J9_CHICK  (aa3-164) | 93.3  91.4 | 2.3e-60  1.6e-164 | 3 | a,c | 10 | <0.01 | **<0.01** |
| **4131**  (aa1-552) (aa1-552) | Hyaluronan synthase 2 (HAS2) **^1,4^** | F1NN14_CHICK  (aa1-552)  G1NIX9_MELGA  (aa1-552) | 100.0  99.8 | 0e0  0e0 | 3 | a,b,c | 4 | <0.01 | **<0.01** |
| **4145**  (aa1-550)  (aa1-550) | Neuron-derived neurotrophic factor (NDNF) | F1NIF2_CHICK  (aa19-568)  G1NE09_MELGA  (aa19-568) | 99.6  99.1 | 0e0  0e0 | 9 | a,b,c | 67 | 0.01 | **0.01** |
| **4183**  (aa19-545)  (aa35-545) | Otopetrin-2 (OTOP2) | F1NP44_CHICK  (aa1-577)  G1NAJ2_MELGA  (aa2-564) | 87.9  87.4 | 4.3e-141  1.6e-133 | 2 | a,b,c | 12 | <0.01 | **<0.01** |
| **7816**  (aa13-353)  (aa13-353)  **420** | Similar to receptor-type tyrosine-protein phosphatase gamma (PTPRG) **^1,2^** | G1N9M7_MELGA  (aa152-705)  F1NA27_CHICK  (aa43-596) | 59.4  59.2 | 2.8e-98  3.0e-98 | 3 | a,b | 6 | <0.01 | **<0.01** |
| **4207**  (aa73-543)  (aa116-543**)** | Ribonuclease/angiogenin inhibitor 1 (RNH1) | Q5ZIY8_CHICK  (aa1-456)  G1NAD7_MELGA  (aa5-431) | 95.3  97.4 | 5.9e-196  1.4e-192 | 3 | a,b,c | 5 | <0.01 | **<0.01** |
| **4210**  (aa1-543)  (aa1-543**)** | Similar to dystroglycan (DAG1) **^1,2^** | R4GH71_CHICK  (aa1-849)  G1MW21_MELGA  (aa1-148) | 63.0  62.5 | 6.4e-149  1.9e-146 | 7 | a,b,c | 26 | 0.01 | **0.01** |
| **4217**  (aa1-542) | Similar to signal peptide, CUB and EGF-like domain-containing protein 2 (SCUBE2) | E1C0H6_CHICK  (aa76-959) | 60.1 | 2.2e-148 | 5 | a,b,c | 73 | 0.03 | **0.04** |
| **4244**  (aa51-341)  (aa51-341) | Tissue alpha-L-fucosidase (FUCA1) **^1,2,4^** | F1P4X3_CHICK  (aa128-418)  G1N260_MELGA  (aa49-339) | 98.6  97.3 | 1.2e-129  3.1e-128 | 2 | a,b,c | 5 | <0.01 | **<0.01** |
| **4269**  (aa1-538)  (aa4-537**)** | Similar to glutamyl aminopeptidase (ENPEP) **^1,2^** | E1BXD9_CHICK  (aa1-943)  G1NEU7_MELGA  (aa1-944) | 51.5  50.2 | 1.5e-71  7.2e-68 | 9 | a,b,c | 45 | <0.01 | **<0.01** |
| **4371**  (aa4-529)  (aa4-529) | Alpha-1,2-Mannosidase (MAN1B1) **^1,2^** | G1MQH0_MELGA  (aa42-632)  F1NGN6_CHICK  (aa85-675) | 87.1  87.1 | 2.0e-158  3.8e-158 | 12 | a,b,c | 54 | 0.01 | **0.01** |
| **4382**  (aa12-528)  (aa12-528) | Radixin/moesin/ezrin (MSN/RDX); shares 6 peptides with 4836 and 3 peptides with 4547 | G1MQ39_MELGA  (aa2-577)  F1NQD9_CHICK  (aa4-582) | 89.6  74.7 | 1.3e-36  3.3e-32 | 1 | a,b,c | 26 | <0.01 | **<0.01** |
| **4394**  (aa1-527)  (aa1-527) | Transketolase (TKT) **^3^** | G1N238_MELGA  (aa20-593)  F1P1A5_CHICK  (aa56-627) | 90.4  90.0 | 9.7e-188  1.7e-187 | 3 | a,b | 4 | <0.01 | **<0.01** |
| **4547**  (aa50-516)  (aa50-516) | Similar to ezrin (EZR) **^1,2,4; t^**; shares 17 peptides with 4836 | Q9YGW6_CHICK  (aa5-585)  G1NJR0_MELGA  (aa5-585) | 70.1  70.0 | 3.0e-84  6.7e-84 | 1 | a,c | 6 | <0.01 | **0.02** |
| **4836**  (aa1-494)  (aa1-494) | Ezrin; shares 17 peptides with 4547 | G1NJR0_MELGA  (aa33-585)  Q9YGW6_CHICK  (aa33-585) | 89.0  89.0 | 8.5e-48  8.5e-48 | 27 | a,b,c | 228 | 0.02 |  |
| **4629**  (aa1-510)  (aa1-510) | Elongation factor 1-alpha 1 (EEF1A) **^1,2^**; shares 5 peptides with 5279 | EF1A_CHICK  (aa1-462)  G1NM38_MELGA  (aa1-462) | 90.4  90.4 | 8.5e-107  8.5e-107 | 13 | a,b,c | 121 | 0.02 | **0.03** |
| **4709**  (aa23-504)  (aa23-504) | Chondroitin sulfate glucuronyltransferase 2 (CHPF2); shares 1 peptide with 3144 (CHPF) | E1BV41_CHICK  (aa288-769)  H9H1T6_MELGA  (aa189-670) | 98.3  97.3 | 0e0  1.2e-215 | 3 | a,b,c | 4 | <0.01 | **<0.01** |
| **4728**  (aa1-497) | Beta-1,4-mannosyl-glycoprotein 4-beta-N-acetylglucosaminyltransferase (MGAT3) | R4GJ57_CHICK  (aa1-497) | 98.8 | 0e0 | 2 | a,b | 5 | <0.01 | **<0.01** |
| **4868**  (aa1-491)  (aa1-491) | Carbohydrate sulfotransferase 3 (CHST3); shares 7 peptides with 5773 | E1C8R3_CHICK  (aa1-458)  G3URX1_MELGA  (aa1-458) | 88.0  86.4 | 2.0e-139  3.5e-135 | 1 | a,b,c | 7 | <0.01 | **<0.01** |
| **5773**  (aa1-437)  (aa1-437) | Carbohydrate sulfotransferase 3 (CHST3); shares 7 peptides with 4868 | G3URX1_MELGA  (aa1-458)  E1C8R3_CHICK  (aa1-458) | 90.0  91.5 | 9.3e-130  5.5e-129 | 10 | a,b,c | 41 | <0.01 |  |
| **4967**  (aa1-485)  (aa1-485) | Butyrylcholinesterase (BCHE) **^1^** | F1NV99_CHICK  (aa25-506)  G1NDD8_MELGA  (aa22-505) | 95.9  95.7 | 4.8e-215  2.9e-214 | 13 | a,b,c | 24 | <0.01 | **0.01** |
| **50**  (aa1-2802)  (aa465-2802) | Teneurin-2 (TENM2); shares 1 peptide with 3116 (Teneurin-4) | TEN2_CHICK  (aa1-2802)  G1MUB5_MELGA  (aa10-2346) | 99.0  99.4 | 0e0  0e0 | 2 | a | 2 | <0.01 | **<0.01** |
| **5112**  (aa1-468)  (aa33-473) | Alpha-2-antiplasmin (SERPINF2) **^1,2,4^**; shares 25 peptides with 6916 | G1N0Z7_MELGA  (aa35-462)  F1NAR5_CHICK  (aa17-461) | 83.3  85.6 | 1.1e-144  1.7e-143 | 37 | a,b,c | 1274 | 1.01 | **0.60** |
| **6916**  (aa30-380)  (aa19-385) | Alpha-2-antiplasmin; shares 25 peptides with 5112 | G1N0Z7_MELGA  (aa40-462)  F1NAR5_CHICK  (aa17-461) | 72.1  70.3 | 1.2e-84  7.6e-84 | 7 | a,b,c | 69 | 0.04 |  |
| **5116**  (aa1-424)  (aa1-424) | Fibroleukin/fibrinogen-like 2 (FGL2) | G1N362_MELGA  (aa1-424)  Q5F3I1_CHICK  (aa1-424) | 98.6  97.2 | 3.3e-176  1.2e-174 | 20 | a,b,c | 94 | 0.05 | **0.05** |
| **5141**  (aa1-456)  (aa65-456) | T-complex protein 1 subunit delta (tcp-1 delta/CCT4) | Q9I8D6_CHICK  (aa1-535)  G1N4A2_MELGA  (aa2-383) | 82.4  93.6 | 8.6e-160  8.3e-142 | 4 | a,b,c | 8 | <0.01 | **<0.01** |
| **5183**  (aa6-440) | T-complex protein 1 subunit eta (CCT7) | F1NK38_CHICK  (aa2-435) | 91.6 | 7.1e-162 | 2 | a,b | 3 | <0.01 | **<0.01** |
| **5218**  (aa204-467)  (aa1-467) | Olfactomedin-like 2A (OLFML2A) **^1,2^** | G1N8B3_MELGA  (aa8-271)  F1P0X8_CHICK  (aa27-655) | 99.2  72.5 | 1.9e-104  6.3e-104 | 7 | a,b,c | 47 | 0.01 | **0.01** |
| **5279**  (aa1-463)  (aa1-461) | Elongation factor 1-alpha (EEF1A2) **^1,2^**; shares 5 peptides with 4629 | F1N9H4_CHICK  (aa1-463)  G1NM38_MELGA  (aa1-461) | 99.6  92.2 | 1.3e-122  4.0e-114 | 1 | a | 1 | <0.01 | **<0.01** |
| **5399**  (aa1-455)  (aa1-456) | Radial spoke head protein 4 A (RSPH4A) | G1NFU8_MELGA  (aa153-607)  E1BXJ4_CHICK  (aa94-550) | 88.4  87.3 | 2.0e-136  2.3e-136 | 3 | a,c | 4 | <0.01 | **<0.01** |
| **6206**  (aa1-418)  (aa1-320)  **5403** | Interleukin-1 receptor accessory protein (IL1RAP) **^1,2^** | G1N684_MELGA  (aa32-449)  E1C117_CHICK  (aa34-353) | 97.6  97.5 | 8.1e-193  8.3e-148 | 12 | a,b,c | 46 | 0.01 | **0.01** |
| **5563**  (aa1-446)  (aa1-446) | Similar to alpha-1,3-mannosyl-glycoprotein 4-beta-N-acetylglucosaminyltransferase B  (MGAT4B) **^1,2,4^** | G1N5R7_MELGA  (aa6-511)  F1N901_CHICK  (aa9-514) | 74.8  74.6 | 2.6e-58  2.7e-58 | 4 | a,b,c | 8 | <0.01 | **<0.01** |
| **5568**  (aa1-446) | Tubulin beta-5 chain; shares 9 peptides with 5781 and other tubulins | TBB5_CHICK  (aa1-446) | 99.3 | 3.0e-198 | 3 | a,b,c | 59 | 0.02 | **0.02** |
| **5571**  (aa54-446)  (aa1-446) | Protein disulfide-isomerase (P4HB) **^1,2^** | G1N1E5_MELGA  (aa2-394)  PDIA1_CHICK  (aa1-515) | 98.7  83.3 | 5.5e-148  6.5e-148 | 20 | a,b,c | 76 | 0.01 | **0.01** |
| **5583**  (aa1-445)  (aa54-445) | Matrix metalloproteinase (MMP13) | F1NZ56_CHICK  (aa41-471)  G1NQJ3_MELGA  (aa1-378) | 94.6  94.9 | 3.1e-98  8.1e-89 | 11 | a,b,c | 20 | <0.01 | **<0.01** |
| **5586**  (aa23-445)  (aa23-445) | DnaJ homolog subfamily C member 3 (DNAJC3) **^1,2^** | DNJC3_CHICK  (aa92-503)  G1NPW6_MELGA  (aa82-494) | 89.4  89.0 | 2.6e-87  3.3e-87 | 27 | a,b,c | 146 | 0.04 | **0.05** |
| **5606**  (aa1-444)  (aa1-398) | Tubulin alpha chain (TUBAL3); shares 9 peptides with 4071 and other tubulins | F1P5B0_CHICK  (aa16-459)  G1MWV8_MELGA  (aa17-413) | 99.8  99.7 | 8.6e-196  1.6e-173 | 1 | b | 1 | <0.01 | **<0.01** |
| **5704**  (aa1-362)  (aa109-406) | CASC4 (cancer susceptibility candidate 4) **^1,2^** | F1NDU0_CHICK  (aa1-366)  G1NCY4_MELGA  (aa1-320) | 80.5  71.6 | 4.1e-65  9.8e-36 | 3 | a,b,c | 7 | <0.01 | **<0.01** |
| **5717**  (aa1-438)  (aa1-439) | Antithrombin-III (SERPINC1) **^1,2^** | G1MT40_MELGA  (aa28-466)  F1NLP7_CHICK  (aa14-453) | 95.2  94.8 | 1.3e-180  1.0e-179 | 20 | a,b,c | 57 | 0.01 | **0.01** |
| **5781**  (aa79-437)  (aa79-437) | Tubulin beta-2 chain (TUBB2B) **^1,2^**; shares 11 peptides with 15996 and CON | G3X8P3_MELGA  (aa84-442)  TBB2_CHICK  (aa87-445) | 98.3  98.3 | 3.2e-150  3.3e-150 | 21 | a,b,c | 355 | 0.08 | **0.10** |
| **5860**  **(aa1-432)**  **(aa1-432)** | Pigment epithelium-derived factor (SERPINF1) **^1,2; i^** | G1N131_MELGA  (aa1-421)  E1C7H6_CHICK  (aa1-421) | 93.8  92.4 | 1.8e-110  3.8e-109 | 31 | a,b,c | 494 | 0.29 | **0.31** |
| **5873**  (aa51-432)  (aa1-432) | Plasma alpha-L-fucosidase (FUCA2) **^2^** | G1NJS8_MELGA  (aa2-408)  E1BS94_CHICK  (aa16-471) | 90.9  90.4 | 2.5e-96  1.9e-95 | 9 | a,b,c | 24 | <0.01 | **<0.01** |
| **5910**  (aa12-430)  (aa12-430) | Carbohydrate sulfotransferase 12  (CHST12) **^1,2^** | E1BR90_CHICK  (aa1-419)  G1NR95_MELGA  (aa1-419) | 98.6  98.3 | 1.8e-193  7.0e-193 | 11 | a,b,c | 48 | 0.01 | **0.01** |
| **5924**  (aa273-327)  (aa273-327) | Uncharacterized/Phospholipid scramblase family (PLSCR1) **^1,2^**; domains: scramblase (aa11-185, 273-328); peptides in second scramblase domain | F1NX66_CHICK  (aa232-286)  G1N4E3_MELGA  (aa165-219) | 90.0  89.1 | 2.2e-19  4.3e-19 | 2 | a,b,c | 21 | <0.01 | **<0.01** |
| **5977**  (aa1-428)  (aa1-427) | Tubulin beta-6 chain; shares 7 peptides with 5781 and other tubulins | TBB6_CHICK  (aa20-446)  G1N9A2_MELGA  (aa20-446) | 99.5  99.5 | 5.0e-196  3.7e-195 | 2 | a,b,c | 20 | 0.01 | **0.02** |
| **6002**  (aa22-402)  (aa134-402) | Similar to P2X purinoceptor (P2XCE) | Q9YI70_CHICK  (aa46-347)  G1MYD3_MELGA  (aa74-346) | 62.8  79.9 | 3.9e-100  1.3e-99 | 3 | a,b,c | 11 | <0.01 | **<0.01** |
| **6024**  (aa1-425)  (aa1-425) | Alpha-1-antitrypsin (SERPINA1) **^1,2^** | E1C7T1_CHICK  (aa1-425)  G1NKH9_MELGA  (aa1-427) | 86.8  83.6 | 1.8e-157  7.3e-153 | 7 | a,b,c | 29 | <0.01 | **<0.01** |
| **6069a**  (aa147-311)  (aa147-311) | Ribosomal protein SA/37kDa laminin receptor (RPSA) **^2,4^** | G1NGU2_MELGA  (aa45-209)  RSSA_CHICK  (aa45-209) | 100.0  100.0 | 8.8e-62  8.8e-62 | 3 | a,b,c | 12 | <0.01 | **<0.01** |
| **6140**  (aa1-420)  (aa297-411) | Torsin-1A-interacting protein 2 (TOR1AIP2) | F1P319_CHICK  (aa1-339)  G1NQZ2_MELGA  (aa1-115) | 80.1  95.7 | 3.5e-88  4.0e-37 | 3 | b,c | 6 | <0.01 | **<0.01** |
| **6157**  (aa12-419)  (aa12-419) | alpha-1,2-Mannosidase (EDEM2) | F1NAE0_CHICK  (aa165-572)  G1MUG3_MELGA  (aa85-494) | 98.0  96.8 | 4.4e-186  1.0e-183 | 4 | a,b,c | 6 | <0.01 | **<0.01** |
| **6163**  (aa9-419)  (aa19-419) | UDP-GlcNAc:betaGal beta-1,3-N-acetylglucosaminyltransferase 7  (B3GNT7) **^1,2,4^** | G1N7V1_MELGA  (aa11-494)  F1NU54_CHICK  (aa5-405) | 97.1  97.5 | 1.0e-183  1.4e-182 | 5 | a,b,c | 18 | <0.01 | **<0.01** |
| **621a**  (aa79-605)  (aa5-605) | Similar to semaphorin-3G (SEMA3G) **^1,2; g,t^** | G1MXI6_MELGA  (aa1-647)  F1NQ93_CHICK  (aa2-716) | 77.1  75.2 | 1.1e-86  1.5e-85 | 35 | a,b,c | 631 | 0.09 | **0.22** |
| **6327**  (aa16-408)  (aa16-408) | Serpin E2/Glia-derived nexin (SERPINE2) **^1,2^** | E1BWU2_CHICK  (aa1-414)  G3URX3_MELGA  (aa1-414) | 87.2  90.6 | 5.4e-137  6.4e-134 | 24 | a,b,c | 420 | 0.31 | **0.31** |
| **6385**  (aa1-407)  (aa1-409) | Neuronal pentraxin-1 (NPTX1) | G1N2Z0_MELGA  (aa38-421)  F1NHL5_CHICK  (aa60-453) | 85.5  90.9 | 4.5e-98  6.1e-81 | 10 | a,b,c | 28 | <0.01 | **<0.01** |
| **6406**  (aa1-408)  (aa1-408) | Fibrinogen beta chain (FGB) **^1,2^** | FIBB_CHICK  (aa56-463)  G3UTA1_MELGA  (aa86-493) | 95.3  95.8 | 8.1e-174  2.9e-173 | 2 | a,b,c | 4 | <0.01 | **<0.01** |
| **652**  (aa1-1206)  (aa1-1206) | Connectin (TTN) | G1NAX9_MELGA  (aa1364-3210)  A6BM71_CHICK  (aa3275-5050) | 63.7  66.3 | 1.7e-136  4.5e-135 | 4 | a,b,c | 9 | <0.01 | **0.01** |
| **6528**  (aa207-376)  (aa22-376) | Polypeptide N-acetylgalactosaminyl-transferase (GALNT12) **^1,2^** | F1NMD3_CHICK  (aa1-229)  G1N5V3_MELGA  (aa1-417) | 73.4  83.9 | 3.0e-60  2.7e-59 | 5 | a,b,c | 7 | <0.01 | **<0.01** |
| **6542**  (aa1-402)  (aa1-402) | Serine protease 35 (PRSS35) | E1BQU9_CHICK  (aa12-413)  G1NRW5_MELGA  (aa12-413) | 97.5  97.3 | 1.9e-157  2.8e-156 | 5 | a,b,c | 6 | <0.01 | **<0.01** |
| **6544b**  (aa293-402)  (aa293-402) | Sentan (SNTN) **^1,2^** | G1NA18_MELGA  (aa35-144)  SNTAN_CHICK  (aa35-144) | 94.5  93.6 | 3.2e-37  5.2e-37 | 5 | a,b,c | 41 | 0.01 | **0.03** |
| **6555**  (aa1-401)  (aa30-400) | Galactose-3-O-sulfotransferase 2 (GAL3ST2) **^1,2,4^**; shares 1 peptide with 8093 and 1843 | E1BUG8_CHICK  (aa16-416)  G1MWB3_MELGA  (aa12-382) | 93.8  91.9 | 5.9e-170  2.7e-154 | 22 | a,b,c | 189 | 0.04 | **0.05** |
| **6577**  (aa4-400)  (aa4-391) | Pantetheinase/biotinidase (LOC100542733) **^1,2^** | G1NKC5_MELGA  (aa108-500)  E1BUA6_CHICK  (aa108-491) | 93.2  92.5 | 5.7e-172  3.2e-167 | 5 | a,b,c | 16 | <0.01 | **<0.01** |
| **6635**  (aa1-384)  (aa59-384) | Similar to beta-galactosidase **^1,2^** (GLB1L); shares 3 peptides with 7550 | F1NW93_CHICK  (aa22-410)  H9H1U2_MELGA  (aa2-332 | 58.6  53.2 | 2.1e-91  9.4e-66 | 3 | a,c | 4 | <0.01 | **0.01** |
| **7550**  (aa11-361)  (aa11-359) | Similar to beta-galactosidase (GLB1L); shares 3 peptides with 6635 | F1NW93_CHICK  (aa146-636)  H9H1U2_MELGA  (aa68-556) | 60.1  59.7 | 7.8e-72  7.4e-71 | 7 | a,b,c | 19 | 0.01 |  |
| **6640**  (aa1-384)  (aa1-384) | Glypican-4 (GPC4) **^1,2,4^** | G1MVZ0_MELGA  (aa55-487)  F1NAU1_CHICK  (aa60-492) | 85.7  85.7 | 1.0e-97  1.0e-97 | 22 | a,b,c | 195 | 0.21 | **0.23** |
| **6642**  (aa1-397)  (aa1-397) | UDP-GlcNAc:betaGal beta-1,3-N-acetylglucosaminyltransferase 2  (B3GNT2) **^1,2^** | G1NRV7_MELGA  (aa1-397)  Q5ZK57_CHICK  (aa1-397) | 97.7  93.2 | 3.0e-184  4.9e-178 | 5 | a,b,c | 18 | <0.01 | **<0.01** |
| **6656**  (aa11-396)  (aa23-396) | Lysosomal Pro-X carboxypeptidase (PRCP) | G1NQQ2_MELGA  (aa31-480)  F1NWF2_CHICK  (aa40-479) | 79.9  80.0 | 1.9e-101  2.7e-97 | 2 | a,b,c | 3 | <0.01 | **<0.01** |
| **6729**  (aa1-393)  (aa1-393) | Protein disulfide-isomerase A3 (PDIA3) **^2,4^**; shares 13 peptides with 8258 | PDIA3_CHICK  (aa81-505)  G1NDL0_MELGA  (aa1-425) | 91.1  90.4 | 4.2e-89  4.6e-88 | 17 | a,b,c | 53 | <0.01 | **<0.01** |
| **8258**  (aa1-340)  (aa1-340) | Protein disulfide-isomerase A3 (PDIA3); shares 13 peptides with 6729 | PDIA3_CHICK  (aa81-420)  G1NDL0_MELGA  (aa1-340) | 98.2  99.7 | 2.0e-138  1.2e-137 | 3 | a,b,c | 12 | <0.01 | **<0.01** |
| **6735**  (aa20-393)  (aa20-323) | Carboxypeptidase E (CPE) **^1^** | G1MTM8_MELGA  (aa1-374)  R4GFJ0_CHICK  (aa96-395) | 100.0  99.3 | 1.8e-170  8.1e-137 | 9 | a,b,c | 20 | <0.01 | **<0.01** |
| **6759**  (aa1-392)  (aa95-335) | Angiopoietin-related protein 4 (ANGPTL4) | F1NUQ4_CHICK  (aa1-402)  H9H0Z9_MELGA  (aa1-246) | 96.3  87.7 | 3.1e-91  5.1e-70 | 4 | a,b,c | 8 | <0.01 | **<0.01** |
| **678**  (aa1-1205)  (aa1-1042) | Calcium-transporting ATPase (ATP2B1) **^1,4^**; shares 4 peptides with 2758 (ATP2B4) | F1NX46_CHICK  (aa1-1205)  G1NFH9_MELGA  (aa1-1042) | 100.0  99.8 | 0e0  0e0 | 2 | a,b,c | 7 | <0.01 | **<0.01** |
| **679**  (aa1-1204)  (aa1-996) | Probable cation-transporting ATPase 13A4 (ATP13A4) **^2^** | AT134_CHICK  (aa1-1204)  G1N5S3_MELGA  (aa1-997) | 100.0  88.9 | 0e0  0e0 | 8 | a,b,c | 24 | <0.01 | **<0.01** |
| **6873**  (aa1-387)  (aa1-387) | Biotinidase (BTD) **^1,2^** | G1NED4_MELGA  (aa131-517)  E1C3J7_CHICK  (aa135-521) | 94.6  93.8 | 1.4e-179  1.1e-177 | 5 | a,b,c | 6 | <0.01 | **<0.01** |
| **6907a**  (aa1-232)  (aa38-247) | Stanniocalcin-1 (STC1) **^1,2^** | F1NU46_CHICK  (aa9-240)  G1MR66_MELGA  (aa9-216) | 99.1  91.4 | 6.0e-85  2.5e-70 | 6 | a,b,c | 20 | <0.01 | **<0.01** |
| **6917**  (aa3-385)  (aa3-385) | Similar to heparanase (HPSE) **^2^** | G1N7R6_MELGA  (aa62-547)  F1NYI9_CHICK  (aa39-524) | 65.7  65.5 | 6.3e-47  9.9e-47 | 8 | a,b,c | 25 | <0.01 | **<0.01** |
| **7050**  (aa10-380)  (aa12-380) | Group XV phospholipase A2/1-O-acylceramide synthase (PLA2G15) **^1,2^** | E1C0B0_CHICK  (aa45-415)  G1MXS1_MELGA  (aa46-395) | 98.1  93.8 | 8.8e-173  1.4e-117 | 3 | a,b | 5 | <0.01 | **<0.01** |
| **7084**  (aa3-378)  (aa3-378) | Actin, cytoplasmic type 5 (ACTG1) **^1; i^**; shares 14 peptides with 7902 and other actins | G1NS52_MELGA  (aa1-376)  ACT5_CHICK  (aa1-376) | 100.0  100.0 | 1.4e-164  1.4e-164 | 20 | a,b,c | 463 | 0.25 | **0.36** |
| **7127**  (aa1-377)  (aa1-377) | Anticoagulant protein C (PROC)/Vitamin K-dependent protein C **^2,4^** | F1NGY6_CHICK  (aa24-433)  G1MRY6_MELGA  (aa24-433) | 84.4  84.9 | 1.8e-134  2.5e-134 | 5 | a,b,c | 19 | <0.01 | **<0.01** |
| **713a**  (aa1-227)  (aa1-227) | Similar to Bone sialoprotein 2 (IBSP) | SIAL_CHICK  (aa1-276)  G1N6F0_MELGA  (aa1-262) | 74.3  63.8 | 7.3e-35  3.4e-20 | 3 | a,b,c | 7 |  | **0.01** |
| **713b**  (aa277-991)  (aa300-788) | Ovocleidin-116 **^1,2,4^**; phosphoprotein; combined with 2298 aa1-40 (2298a) | OC116_CHICK  (aa19-732)  G1N6E1_MELGA  (aa41-499) | 80.2  75.1 | 3.2e-143  1.0e-80 | 45 | a,b,c | 8670 | 30.15 | **46.40** |
| **2298a**  (aa1-40)  (aa1-40) | Ovocleidin-116 | OC116_CHICK  (aa1-40)  G1N6E1_MELGA  (aa1-40) | 100.0  82.5 | 6.2e-16  2.0e-11 | 2 | a,b,c | 168 | 1.49 |  |
| **2298b**  (aa41-743)  (aa41-506) | Ovocleidin-116; no peptides shared with 713b! Not combined! | OC116_CHICK  (aa41-743)  G1N6E1_MELGA  (aa41-499) | 98.7  79.4 | 0e0  2.3e-104 | 2 | a,b,c | 4 | - | **<0.01** |
| **713c**  (aa959-1185)  (aa959-1185) | Osteopontin (SPP1) **^1,2,4^**; shares all peptides with Q9I832 and P23498; phosphoprotein | F1NSM8_CHICK  (aa37-264)  G1N6D8_MELGA  (aa37-263) | 83.4  80.3 | 2.1e-40  6.4e-39 | 4 | a,b,c | 140 | - | **0.28** |
| **Q9I832**  (aa1-264)  (aa1-264) | Osteopontin; shares 4 peptides with 713c | G1N6D8_MELGA  (aa1-163)  F1NSM8_CHICK  (aa1-264) | 91.7  91.7 | 2.5e-74  4.1e-74 | 10 | a,b,c | 84 | 0.14 |  |
| **7172**  (aa1-373)  (aa1-373) | Nucleobindin-2 (NUCB2) **^1,2,4^** | F1NGB1_CHICK  (aa20-392)  G1N538_MELGA  (aa20-392) | 99.7  99.2 | 9.6e-100  3.6e-99 | 28 | a,b,c | 188 | 0.07 | **0.07** |
| **7229**  (aa1-373)  (aa1-373) | Beta-1,4-galactosyltransferase  (B4GALT2) **^2^** | F1N8F7_CHICK  (aa1-373)  G1NC93_MELGA  (aa19-316) | 98.7  78.3 | 1.7e-142  5.0e-87 | 4 | a,b,c | 18 | <0.01 | **<0.01** |
| **7257**  (aa1-372)  (aa1-372) | Serine protease 23 (PRSS23) **^1,2^** | G1NRE7_MELGA  (aa1-372)  E1C744_CHICK  (aa1-372) | 98.9  98.7 | 4.6e-171  1.4e-170 | 10 | a,b,c | 22 | <0.01 | **<0.01** |
| **736**  (aa22-1046)  (aa108-1046**)** | Similar to cation-independent mannose-6-phosphate receptor/IGF2R **^1,2^**; shares 1 peptide with 80 | Q90681_CHICK  (aa488-1804)  G1NIM4_MELGA  (aa572-1765) | 66.2  69.9 | 5.7e-188  1.1e-183 | 3 | a,b | 4 | <0.01 | **<0.01** |
| **80**  (aa1-2470)  (aa38-2470) | Cation-independent mannose-6-phosphate receptor; shares 1 peptide with 736 | Q90681_CHICK  (aa1-2470)  G1NIM4_MELGA  (aa38-2470) | 100.0  94.2 | 0e0  0e0 | 2 | a,c | 3 | <0.01 | **<0.01** |
| **7379**  (aa6-367)  (aa6-367) | Urokinase-type plasminogen activator (PLAU) **^2^** | G1N3G3_MELGA  (aa74-434)  UROK_CHICK  (aa74-434) | 92.8  89.8 | 2.4e-163  7.3e-157 | 2 | a,b,c | 5 | <0.01 | **<0.01** |
| **7492**  (aa1-336)  (aa1-179) | Similar to CD14 antigen (CD14) | F1P3I9_CHICK  (aa34-356)  G3USU3_MELGA  (aa34-212) | 65.3  83.2 | 8.9e-77  2.8e-55 | 3 | a,b,c | 7 | <0.01 | **<0.01** |
| **7583**  (aa19-347)  (aa27-352) | Eukaryotic initiation factor 4A-II (EIF4A2) **^1,2^** | G1NAW6_MELGA  (aa1-333)  IF4A2_CHICK  (aa10-338) | 98.5  97.9 | 2.6e-125  8.9e-124 | 8 | a,b,c | 17 | <0.01 | **<0.01** |
| **7643**  (aa1-358)  (aa1-358) | Bifunctional heparan sulfate N-deacetylase/N-sulfotransferase 2 (NDST2) | E1BYQ4_CHICK  (aa522-879)  G1N3Z2_MELGA  (aa522-878) | 99.7  99.2 | 1.2e-171  4.7e-170 | 7 | a,b,c | 26 | <0.01 | **<0.01** |
| **7992**  (aa1-331)  (aa1-331) | Bifunctional heparan sulfate N-deacetylase/N-sulfotransferase 2 (NDST2) | G1N3Z2_MELGA  (aa1-331)  E1BYQ4_CHICK  (aa1-331) | 100.0  99.7 | 9.7e-153  2.4e-152 | 4 | a,b,c | 11 | <0.01 |  |
| **7675**  (aa30-357)  (aa30-257) | GM3 synthase (SIAT9) **^1^** | G1NKA2_MELGA  (aa41-368)  Q6R3Q5_CHICK  (aa41-368) | 96.0  95.7 | 2.8e-149  1.5e-148 | 6 | a,b,c | 17 | <0.01 | **<0.01** |
| **7695**  (aa42-356) | Guanine nucleotide-binding protein G(i) subunit alpha-2 (GNAI2) **^1^** | H9KZ88_CHICK  (aa26-340) | 100.0 | 8.9e-141 | 9 | a,b,c | 19 | 0.01 | **0.01** |
| **7717**  (aa1-347)  (aa1-347) | Amyloid protein (AAP)/Beta-amyloid protein 695 isoform **^1,2^**; shares 9 peptides with 2256 and 1 with 2239 | G3USJ4_MELGA  (aa125-471)  F1P0B2_CHICK  (aa125-477) | 99.7  99.7 | 2.2e-90  2.2e-90 | 1 | c | 2 | <0.01 | **<0.01** |
| **7743b**  (aa191-355)  (aa191-355) | Charged multivesicular body protein 1b (CHMP1B) | F1NVQ6_CHICK  (aa38-185)  G1MSD9_MELGA  (aa35-182) | 89.7  89.7 | 3.5e-36  3.5e-36 | 2 | a,b,c | 4 | <0.01 | **<0.01** |
| **780**  (aa1-1142)  (aa109-1142) | Golgi apparatus protein 1 (GLG1) **^1,2,4^** | GSLG1_CHICK  (aa1-1142)  G1MRQ6_MELGA  (aa1-1034) | 100.0  99.6 | 0e0  0e0 | 59 | a,b,c | 613 | 0.06 | **0.07** |
| **7859**  (aa1-352)  (aa1-338) | Tsukushin (TSKU) **^1,2,4; g,t^** | F1NDH7_CHICK  (aa1-352)  G1NRF2_MELGA  (aa1-343) | 98.3  95.6 | 9.3e-133  1.9e-124 | 32 | a,b,c | 933 | 1.62 | **1.51** |
| **7861**  (aa1-352)  (aa1-352) | Carboxypeptidase (CTSA) **^1,2,4^** | F1NIN2_CHICK  (aa92-471)  G1N8A7_MELGA  (a91-470) | 91.6  91.6 | 1.5e-122  7.9e-122 | 8 | a,b,c | 44 | 0.02 | **0.02** |
| **7869**  (aa1-351)  (aa1-351) | Tubulin alpha-2/8 (TUBA8); shares 5 peptides with 4071 and other tubulins | F1NMP3_CHICK  (aa2-352)  G3UTF6_MELGA  (aa2-352) | 99.1  98.3 | 7.8e-153  4.5e-152 | 1 | c | 2 | <0.01 | **<0.01** |
| **7897**  (aa1-317)  (aa141-317) | Polypeptide N-acetylgalactosaminyltransferase (WBSCR17) | E1BVE9_CHICK  (aa2-352)  G1MTN9_MELGA  (aa1-181) | 89.2  80.0 | 3.7e-120  1.6e-56 | 3 | a,b,c | 5 | <0.01 | **<0.01** |
| **7902**  (aa1-324)  (aa1-324) | Beta-actin (ACTB) **^1,4^**; shares 14 peptides with 7084 and other actins | ACTB_CHICK  (aa1-291)  Q58J72_MELGA  (aa1-291) | 88.0  88.0 | 4.8e-82  4.8e-82 | 2 | a,b | 4 | <0.01 | **<0.01** |
| **7942**  (aa1-349)  (aa1-349) | Lactosylceramide 1,3-N-acetyl-beta-D-glucosaminyltransferase (B3GNT5) **^2^** | F1NRQ5_CHICK  (aa26-374)  G1NS21_MELGA  (aa26-374) | 97.4  96.8 | 9.5e-161  2.6e-160 | 4 | a,b,c | 6 | <0.01 | **<0.01** |
| **7982**  (aa14-348)  (aa14-348) | 45 kDa calcium-binding protein (SDF4) **^1,2^** | G1MW20_MELGA  (aa20-363)  CAB45_CHICK  (aa13-356) | 92.5  91.9 | 4.6e-74  6.7e-74 | 15 | a,b,c | 72 | 0.03 | **0.03** |
| **8009**  (aa14-344) | Polymeric-immunoglobulin receptor (PIGR) **^1,2^** | G3UTH8_MELGA  (aa26-356) | 85.2 | 1.2e-129 | 8 | a,b,c | 47 | 0.01 | **0.02** |
| **8019**  (aa1-347) | Uncharacterized; domains: Glycoside_hydrolase_3_Nterm and Cterm | H9KY26_CHICK  (aa56-584) | 62.0 | 2.2e-54 | 7 | a,b,c | 46 | 0.02 | **0.02** |
| **8027**  (aa1-347)  (aa1-347) | Angiopoietin-related protein 7  (ANGPTL7) **^2^** | E1BRK7_CHICK  (aa1-348)  G1MZY3_MELGA  (aa1-348) | 99.4  98.9 | 1.6e-153  6.7e-153 | 13 | a,b,c | 82 | 0.05 | **0.06** |
| **8047**  (aa11-346)  (aa1-346) | Ovalbumin-related protein Y (SERPINB14B) **^1,2; i^**; shares 1 peptide with 9384 (Ovalbumin-related protein X) | OVALY_CHICK  (aa1-388)  G1MZF4_MELGA  (aa3-388) | 79.1  77.4 | 1.7e-80  8.9e-50 | 13 | a,b,c | 177 | 0.06 | **0.05** |
| **8075**  (aa13-346)  (aa13-346) | Tubulin alpha-3 chain (TUBA3E) **^1,2^** | G1NEY3_MELGA  (aa113-450)  F1N9J7_CHICK  (aa113-450) | 97.0  97.0 | 1.4e-147  1.4e-147 | 4 | a,b,c | 49 | 0.01 | **0.02** |
| **8094**  (aa1-354)  (aa1-345) | Glycosaminoglycan xylosylkinase (FAM20B) **^1,2^** | F1NZJ0_CHICK  (aa1-407)  G1MUV3_MELGA  (aa1-405) | 83.9  83.6 | 1.0e-112  1.0e-112 | 2 | a,b,c | 5 | <0.01 | **<0.01** |
| **8164b**  (aa139-343)  (aa153-342) | Peflin (PEF1) **^1^** | H9KZ63_CHICK  (aa13-223)  G3UT72_MELGA  (aa2-188) | 82.9  87.9 | 1.7e-64  1.6e-61 | 5 | a,c | 7 | <0.01 | **<0.01** |
| **8202**  (aa1-342)  (aa1-342) | Arylsulfatase A (ARSA) **^1,2^** | F1NWF7_CHICK  (aa88-506)  G1MU55_MELGA  (aa14-432) | 79.7  78.5 | 4.6e-104  5.2e-104 | 4 | a,b,c | 8 | <0.01 | **<0.01** |
| **8206**  (aa1-342)  (aa1-342) | Interstitial collagenase (MMP1) | G1NQJ6_MELGA  (aa132-473)  E1C9D9_CHICK  (aa125-466) | 93.6  93.3 | 5.2e-145  8.2e-145 | 14 | a,b,c | 69 | 0.02 | **0.03** |
| **8279**  (aa1-288)  (aa2-288) | PIT 54 **^1,2; g,t^** | Q98TD1_CHICK  (aa131-459)  G1NEQ0_MELGA  (aa132-459) | 84.2  84.8 | 2.0e-81  7.3e-81 | 7 | a,b,c | 41 | 0.01 | **0.01** |
| **8287**  (aa19-339)  (aa2-339) | Serine protease HTRA1 **^2^** | G1NG99_MELGA  (aa1-322)  F1ND64_CHICK  (aa7-345) | 85.7  82.2 | 8.6e-92  9.3e-92 | 2 | a,b,c | 4 | <0.01 | **<0.01** |
| **8300**  (aa3-281)  (aa8-281) | Cytosolic non-specific dipeptidase (CNDP2); shares 2 peptides with 8626 | G3UQW6_MELGA  (aa23-329)  F1P463_CHICK  (aa1-302) | 84.7  84.4 | 2.1e-59  1.7e-57 | 3 | a | 3 | <0.01 | **<0.01** |
| **8626**  (aa1-329)  (aa1-329) | Cytosolic non-specific dipeptidase (CNDP2); shares peptides with 8300 | G1N7Z4_MELGA  (aa69-452)  Q5ZLV5_CHICK  (aa69-452) | 82.0  81.8 | 4.4e-97  4.5e-97 | 1 | a,b,c | 3 | <0.01 |  |
| **8367a**  (aa1-96)  (aa36-96) | Macrophage migration inhibitory factor (MIF) **^1^** | MIF_CHICK  (aa1-97)  G1N6G2_MELGA  (aa1-62) | 99.0  98.4 | 3.0e-29  1.3e-15 | 3 | a,b,c | 9 | <0.01 | **<0.01** |
| **8543**  (aa17-331)  (aa17-331) | Retinoid-inducible serine carboxypeptidase (SCPEP1) **^1,2^** | Q5F3W4_CHICK  (aa95-446)  G1N836_MELGA  (aa88-439) | 86.6  84.4 | 1.2e-96  1.7e-94 | 6 | a,b,c | 31 | 0.03 | **0.03** |
| **8574**  (aa1-324)  (aa1-324) | Gastric intrinsic factor (GIF) | G1MW98_MELGA  (aa4-328)  R4GLQ4_CHICK  (aa35-359) | 76.3  76.0 | 1.3e-103  1.8e-102 | 12 | a,b,c | 215 | 0.39 | **0.42** |
| **871**  (aa575-1096)  (aa559-1095) | Similar to acid alpha-glucosidase (GAA) **^1,2^**; shares 6 peptides with O73632 | G1N308_MELGA  (aa106-762)  E1BU22_CHICK  (aa265-929) | 72.6  42.9 | 4.1e-89  3.8e-42 | 1 | b | 1 | <0.01 | **<0.01** |
| **O73632**  (aa122-873) | Acid alpha glucosidase; shares 6 peptides with 871 | G1N308_MELGA  (aa3-762) | 94.3 | 0e0 | 11 | a,b,c | 19 | <0.01 |  |
| **8715a**  (aa7-53)  (aa7-53) | Regenerating islet-derived  protein 4 (REG4) **^1,2^** | G1MZE6_MELGA  (aa54-100)  E1BZV4_CHICK  (aa54-100) | 91.5  91.5 | 1.6e-16  1.6e-16 | 3 | a,b,c | 50 | 0.03 | **0.11** |
| **9191**  (aa6-314)  (aa1-314)  **8717** | Notum-like protein (NOTUM) **^1,2^** | G1N152_MELGA  (aa40-352)  F1NBY4_CHICK  (aa1-354) | 95.8  88.1 | 6.5e-138  6.5e-138 | 3 | a,b | 3 | <0.01 | **<0.01** |
| **875a**  (aa25-357)  (aa19-357) | GDP-fucose protein O-fucosyltransferase (FUT13/POFUT2) **^1,2^** | Q7T1N6_CHICK  (aa1-369)  H9H0A3_MELGA  (aa48-423) | 83.2  83.6 | 3.0e-79  2.2e-78 | 14 | a,b,c | 40 | <0.01 | **0.01** |
| **8915**  (aa1-291)  (aa1-291) | Polypeptide N-acetylgalactosaminyl-transferase (GALNT10) | G1N294_MELGA  (aa44-334)  F1NX01_CHICK  (aa105-395) | 100.0  100.0 | 3.6e-132  4.1e-132 | 6 | a,b,c | 10 | <0.01 | **<0.01** |
| **9040**  (aa1-318)  (aa1-318) | D-glucuronyl C5-epimerase (GLCE) | G1NCS7_MELGA  (aa288-605)  R4GJP5_CHICK  (aa288-605) | 99.1  98.1 | 2.2e-142  1.2e-141 | 6 | a,b,c | 12 | <0.01 | **<0.01** |
| **9043**  (aa1-318)  (aa1-318) | C1GALT1-specific chaperone 1 (C1GALT1C1) **^2^** | E1BSH4_CHICK  (aa1-318)  G3UUU5_MELGA  (aa1-318) | 97.5  96.5 | 6.9e-147  1.6e-146 | 11 | a,b,c | 24 | <0.01 | **<0.01** |
| **9047**  (aa1-317)  (aa1-317) | Guanine nucleotide-binding protein subunit beta-2-like 1 (GNB2L1) **^2^** | GBLP_CHICK  (aa1-317)  B1N1C2_MELGA  (aa1-317) | 100.0  100.0 | 4.1e-109  4.1e-109 | 4 | b,c | 7 | <0.01 | **<0.01** |
| **9087**  (aa65-288) | Ras-related protein Ral-B (RALB) **^1^** | E1BVT0_CHICK  (aa20-227) | 81.3 | 1.5e-58 | 4 | a,b,c | 7 | <0.01 | **<0.01** |
| **9140**  (aa4-315)  (aa4-184) | Sulfotransferase/Heparan sulfate glucosamine 3-O-sulfotransferase 1 (HS3ST1) **^1,2^** | E1C4I5_CHICK  (aa9-320)  G3UQ78_MELGA  (aa2-172) | 97.1  89.5 | 1.5e-134  7.4e-64 | 16 | a,b,c | 92 | 0.02 | **0.04** |
| **9170**  (aa39-313)  (aa39-313) | Angiopoietin-related protein 1 (ANGPTL1) | E1BYT9_CHICK  (aa1-275)  G1MUI0_MELGA  (aa1-275) | 98.9  98.5 | 1.4e-93  4.5e-93 | 2 | a,b,c | 8 | <0.01 | **<0.01** |
| **9190**  (aa37-314) | Uncharacterized/similar to alpha-1,3-mannosyl-glycoprotein 2-beta-N-acetylglucosaminyltransferase; A0A093CA76_9AVES, aa1-293, 54.0%, 1.8e-60; domain: glycosyl_transferase_13 |  |  |  | 4 | a,c | 10 | <0.01 | **<0.01** |
| **9197**  (aa26-314)  (aa12-314) | Syndecan (SDC1) **^2^** | F1NV24_CHICK  (aa25-313)  G1NMN2_MELGA  (aa11-313) | 95.8  90.1 | 2.0e-92  6.1e-90 | 2 | a,c | 3 | <0.01 | **<0.01** |
| **9238**  (aa1-313)  (aa1-313) | Similar to Solute carrier family 2, facilitated glucose transporter member 3 (SLC2A3) **^1^** | F1NTJ1_CHICK  (aa109-496)  G1NME1_MELGA  (aa110-499) | 71.6  71.8 | 8.0e-51  5.9e-49 | 6 | a,b,c | 27 | 0.01 | **0.01** |
| **9260**  (aa1-312)  (aa1-311) | Alpha-N-acetylgalactosaminidase  (NAGAB) **^1,2^** | NAGAB_CHICK  (aa36-405)  G1UPD1_MELGA  (aa37-405) | 79.2  75.1 | 3.5e-62  3.4e-58 | 5 | a,b,c | 12 | <0.01 | **<0.01** |
| **9318**  (aa1-310)  (aa1-310) | Similar to D-3-phosphoglycerate dehydrogenase (PHGDH) | E1C7Y3_CHICK  (aa1-401)  G1MZB5_MELGA  (aa1-401) | 76.8  76.3 | 6.2e-108  4.2e-107 | 7 | a,b,c | 9 | <0.01 | **<0.01** |
| **935**  (aa1-1072)  (aa42-1072) | Integrin alpha-6 (ITGA6) **^1,2^** | ITA6_CHICK  (aa1-1072)  G1NCA0_MELGA  (aa2-1032) | 100.0  98.2 | 0e0  0e0 | 5 | a,b,c | 6 | <0.01 | **<0.01** |
| **9456**  (aa5-301)  (aa1-301) | Similar to UDP-galactose 4-epimerase (GALE) | G1N202_MELGA  (aa32-319)  F1NWE5_CHICK  (aa4-2969 | 74.5  74.9 | 8.9e-61  6.8e-59 | 4 | a,c | 7 | <0.01 | **<0.01** |
| **9561**  (aa1-304)  (aa1-304) | Serine/threonine-protein phosphatase (PPP1CC) | G1N0K7_MELGA  (aa5-308)  Q5ZL39_CHICK  (aa20-323) | 100.0  100.0 | 4.6e-147  4.8e-147 | 3 | a,b,c | 4 | <0.01 | **<0.01** |
| **9593**  (aa20-303)  (aa20-303) | Di-N-acetylchitobiase (CTBS) **^1,2^**; shares 7 peptides with 8527b | G1NAD9_MELGA  (aa44-367)  F1NRM4_CHICK  (aa53-375) | 82.4  79.3 | 1.2e-66  1.5e-66 | 10 | a,b,c | 69 | 0.03 | **0.02** |
| **8527b**  (aa106-332)  (aa106-332) | Di-N-acetylchitobiase (CTBS); shares 7 peptides with 9593 | F1NRM4_CHICK  (aa53-318)  G1NAD9_MELGA  (aa44-310) | 79.4  82.4 | 3.2e-41  3.6e-41 | 1 | a,b,c | 10 | - |  |
| **9639**  (aa1-302) | Similar to beta-galactosidase (GLB1) **^1,2^** | G1NGW0_MELGA  (aa316-648) | 75.2 | 5.4e-97 | 7 | a,b,c | 51 | 0.04 | **0.07** |
| **9655**  (aa1-273)  (aa1-273) | DnaJ subfamily B member 11 (DNAJB11) **^1^** | G1MUQ1_MELGA  (aa76-337)  F1NVY5_CHICK  (aa82-343) | 94.1  94.5 | 2.4e-61  2.4e-61 | 3 | a,b,c | 8 | <0.01 | **<0.01** |
| **9668**  (aa25-301)  (aa25-301) | Deoxyribonuclease II (DNASE2) **^1^** | Q2XP49_CHICK  (aa21-363)  G1NAH7_MELGA  (aa20-362) | 76.4  76.4 | 1.0e-68  3.8e-68 | 4 | a,b,c | 11 | <0.01 | **0.01** |
| **9750**  (aa46-228)  (aa51-228) | Chloride intracellular channel protein 6 (CLIC6) **^1,4^**; shares 2 peptides with 11834 (CLIC4) | G1NNX6_MELGA  (aa1-183)  F1P2Y2_CHICK  (aa16-193) | 99.5  97.8 | 3.1e-76  8.0e-73 | 1 | a | 1 | <0.01 | **<0.01** |
| **979b**  (aa927-1061)  (aa927-1061) | Uncharacterized/RAC3; shares 4 peptides with 30108 (RAC1) | F6UBD2_CHICK  (aa37-192)  G1N107_MELGA  (aa34-189) | 85.9  85.9 | 2.9e-21  4.3e-21 | 5 | a,b,c | 19 | <0.01 | **0.01** |
| **985b**  (aa316-1054)  (aa316-1054) | Uncharacterized/Similar to membrane-bound transcription factor site-1 protease (MBTPS1) **^2^** | E1C6Y2_CHICK  (aa63-1060)  G1MQ93_MELGA  (aa63-1060) | 73.1  73.0 | 6.9e-117  6.9e-177 | 4 | a,b,c | 11 | - | **<0.01** |
| **9862**  (aa1-297) | Cathepsin L (CTSL2) **^1,2^** | F1NYJ1_CHICK  (aa16-353) | 85.8 | 3.5e-115 | 7 | a,b,c | 42 | 0.02 | **0.02** |
| **9934**  (aa1-295)  (aa1-295) | Actin (ACTG2/ACTA2)**^4^**; shares 6 peptides with 7084 and other actins | G1NIB8_MELGA  (aa46-377)  F1P476_CHICK  (aa46-377) | 88.6  88.6 | 1.7e-106  1.7e-106 | 4 | a,b,c | 31 | 0.08 | **0.09** |
| **A0PA16**  (aa1-646)  (aa1-646)  **5390**  **3087** | HSP-70 (HSPA8); shares 2 peptides with 3436 (HSPA5) **^1,2^** | G1MSW3_MELGA  (aa1-646)  F1NWP3_CHICK  (aa1-646) | 100.0  100.0 | 0e0  0e0 | 25 | a,b,c | 146 | 0.02 | **0.01** |
| **A5HTZ3**  (aa1-450)  (aa1-450) | Axoneme central apparatus protein (SPAG6) | M1T4J8_CHICK  (aa23-472)  A5HTZ4_MELGA  (aa1-450) | 92.9  92.4 | 2.4e-180  4.0e-178 | 3 | a,b,c | 7 | <0.01 | **<0.01** |
| **A5HU02**  (aa1-213)  (aa1-213) | CD9 protein/tetraspannin **^1,2^** | Q9IBC9_CHICK  (aa8-220)  A5HU04_MELGA  (aa1-213) | 91.5  90.1 | 6.2e-90  8.5e-87 | 2 | a,b,c | 28 | 0.01 | **0.01** |
| **A9CTP4**  (aa42-153)  (aa1-153) | Glutathione peroxidase 1 (GPx1) | R4GH86_CHICK  (aa1-111)  G1MTT2_MELGA  (aa10-151) | 90.3  81.8 | 1.2e-46  3.4e-42 | 3 | a,b,c | 11 | <0.01 | **<0.01** |
| **B9A8Q3**  (aa1-238)  (aa1-238) | Riboflavin-binding protein **^1,2^** | RBP_CHICK  (aa1-238)  G1NDJ8_MELGA  (aa3-240) | 89.5  89.1 | 6.0e-103  1.7e-102 | 6 | a,b,c | 30 | 0.01 | **0.01** |
| **I0J150**  (aa1-80)  (aa1-80) | Hep21 **^1,2^** (fragment) | Q8AAV77_CHICK  (aa19-98)  B1N1B6_MELGA  (aa19-98) | 96.2  95.0 | 2.3e-35  1.4e-34 | 2 | a,c | 4 | <0.01 | **<0.01** |
| **O93390**  (aa1-298)  (aa1-240)  **14899** | SPARC/BM-40/osteonectin **^1,2^** | F1P291_CHICK  (aa1-298)  G1N2Z5_MELGA  (aa1-240) | 97.7  99.2 | 6.6e-128  1.2e-102 | 9 | a,b,c | 28 | 0.01 | **0.01** |
| **P00701**  **(aa1-147)**  **(aa1-147)** | Lysozyme C (LYZ) **^1,2; g^** | LYSC_CHICK  (aa1-147)  LYSC_MELGA  (aa1-147) | 95.2  92.5 | 2.8e-68  8.6e-66 | 11 | a,b,c | 178 | 0.13 | **0.14** |
| **P01003**  (aa1-186)  (aa2-186)  **15709** | Ovomucoid **^1,2; i^** | IOVO_MELGA  (aa1-185)  IOVO_CHICK  (aa26-210) | 83.3  76.2 | 1.7e-67  2.1e-62 | 15 | a,b,c | 300 | 0.21 | **0.11** |
| **P14018**  (aa1-451)  (aa1-312)  **6174** | Clusterin (CLU) **^1,2,4; i^** | Q9YGP0_CHICK  (aa1-448)  G1NMV6_MELGA  (aa1-311) | 94.9  95.2 | 7.5e-178  1.2e-123 | 27 | a,b,c | 867 | 0.50 | **0.31** |
| **P24589**  (aa1-142)  (aa1-142**)**  **14194** | Hemoglobin subunit alpha-A (HBAA) **^1,2; g^** | HBA_CHICK  (aa1-142)  HBA_MELGA  (aa1-142) | 93.7  93.0 | 1.9e-55  5.3e-55 | 5 | a,b,c | 13 | <0.01 | **<0.01** |
| **P32918**  (aa1-264)  (aa1-264)  **12041** | Apolipoprotein A-I (APOA1) **^1,2,4; g,t^** | G1MVX1_MELGA  (aa1-264)  APOA1_CHICK  (aa1-264) | 92.8  92.4 | 1.1e-65  1.4e-65 | 41 | a,b,c | 323 | 0.30 | **0.17** |
| **P42642**  (aa1-353)  (aa102-353)  **12505** | Protein NOV **^2^** | F1NYE0_CHICK  (aa1-351)  G1MTF5_MELGA  (aa1-252) | 96.9  99.2 | 1.1e-148  1.3e-105 | 6 | a,b,c | 12 | <0.01 | **<0.01** |
| **P67965**  (aa1-216)  (aa40-216) | Vascular endothelial growth factor A (VEGFA) **^1,2^** | VEGFA_CHICK  (aa1-216)  G1NEJ1_MELGA  (aa1-177) | 100.0  100.0 | 3.6e-91  3.2e-74 | 3 | a,c | 3 | <0.01 | **<0.01** |
| **P79886**  (aa1-105)  (aa1-105) | Apovitellenin-1 **^1,2^** | G1NND5_MELGA  (aa1-106)  APOV1_CHICK  (aa1-106) | 71.7  72.6 | 1.1e-29  1.4e-27 | 3 | a,c | 7 | <0.01 | **0.01** |
| **Q05025**  (aa1-333)  (aa1-333)  **971** | Glyceraldehyde-3-phosphate dehydrogenase (GAPDH) **^1,2^** | G1NMR6_MELGA  (aa1-333)  G3P_CHICK  (aa1-333) | 98.9  98.8 | 4.3e-139  4.3e-139 | 13 | a,b,c | 65 | 0.03 | **0.01** |
| **Q5UAX9**  (aa1-877)  (aa1-466) | Heparan sulfate 6-O endosulfatase 2/Extracellular sulfatase Sulf-2 (Sulf2) **^1,2^** | E1BZH8_CHICK  (aa1-877)  G1MZI0_MELGA  (aa1-466) | 98.6  98.3 | 0e0  5.7e-199 | 7 | a,b,c | 16 | <0.01 | **<0.01** |
| **Q6PRV1**  (aa1-180)  (aa38-181)  **17575**  **26589** | Ferritin | FRIH_CHICK  (aa1-180)  G1NB58_MELGA  (aa2-145) | 98.9  99.3 | 7.7e-77  3.7e-60 | 6 | a,b,c | 17 | <0.01 | **<0.01** |
| **Q7ZUB2**  (aa61-195)  (aa94-195**)**  **23018** | Ribosomal protein S17 | RS17_CHICK  (aa1-135)  G1MRJ1_MELGA  (aa1-102) | 100.0  100.0 | 8.3e-53  6.0e-38 | 2 | a,b | 4 | <0.01 | **<0.01** |
| **Q8JIP4**  (aa4-285)  **15425** | Epimorphin/syntaxin-2 | G1MTV4_MELGA  (aa2-283) | 91.1 | 6.3e-83 | 5 | a,b,c | 9 | <0.01 | **<0.01** |
| **Q9DGI2**  (aa1-227) | IGFBP5 **^1^** | F1ND88_CHICK  (aa45-271) | 99.1 | 1.1e-96 | 17 | a,b,c | 232 | 0.11 | **0.13** |
| **Q9I9P7**  (aa1-178)  (aa1-178) | Extracellular fatty acid-binding protein (EXFAB) **^1,2^** | G1MQ16_MELGA  (aa1-178)  E1C0K1_CHICK  (aa1-178) | 89.3  87.6 | 3.6e-68  5.1e-68 | 12 | a,b,c | 155 | 0.32 | **0.34** |
|  |  |  |  |  |  |  |  |  |  |

^1^, also identified in chicken eggshell matrix proteomes [Mann et al, 2006; Mann et al, 2007;Miksik et al, 2007; Miksik et al, 2010; Rose-Martel et al, 2012; Sun et al, 2013; Miksik et al., 2014; Marie et al., 2015]; ^2^, also identified in turkey eggshell matrix proteome [Mann and Mann, 2013]; ^3^, not identified in chicken eggshell matrix previously, but in chicken eggshell gland fluid [Sun et al, 2013; Marie et al., 2015]. **^4^**, gene expression in chicken uterus up-regulated upon sexual maturation [Dunn et al, 2009] or during shell calcification [Brionne et al, 2014]. Highest abundance in chicken uterus fluid during **^i^**, initial phase, **^g^**, growth phase, **^t^**, terminal phase [Marie et al, 2015]. Protein fragments sharing similarity to the same turkey or chicken protein are tentatively grouped together as indicated by alternate white and grey bachground.
