# Supplementary material for: Proteomic analysis of quail calcified eggshell matrix: a comparison to chicken and turkey eggshell proteomes
Source: Proteome Sci. 2015 Aug 27;13:22. doi: 10.1186/s12953-015-0078-1 (PMC4550075; doi:10.1186/s12953-015-0078-1)
Supplement: Additional file 4: Figure S1. — Alignments of quail accessions predicted to contain sequences (or partial sequences) of different proteins with the most similar chicken or turkey protein sequences. Also included is the location of identified peptides. (DOCX 143 kb) [file 12953_2015_78_MOESM4_ESM.docx]

**FIGURE S1**

**10515**

H9KZB0_CHICK 1 MSGSTSQRAAVFVLLFALLMLLIIYSSSSGTEVFPYSALRGRARRPPNLRHWGVSSGYLP

10515 ------------------------------------------------------------

KAD1_CHICK ------------------------------------------------------------

H9KZB0_CHICK 61 VSGNKSLTAHCHQCAIVTSSSHLLGTHLGAEIDQAECTIRMNDAPTTGYEADVGNKTTFR

10515 ------------------------------------------------------------

KAD1_CHICK ------------------------------------------------------------

H9KZB0_CHICK 121 VVAHSSIYRVLKRPQEFVNKTPETVLIFWGPPAKMQKGLLKIIQRVGASFPNMTAYVVSP

10515 1 ----------------------------------MQKGLLKIIQRVGASFPNMTAYVVSP

KAD1_CHICK 1 ---------------------------------------------------------MST

H9KZB0_CHICK 181 HRMKQFDELFR-GETGKDREKSRSWLSTGWFTMGIAVELCDA-VHVYGMVPPNYCS---Q

10515 27 HR**MKQFDELFR**-GETGKDREKSRSWLSTGWFTMGIAVELCDA-VHVYGMVPPNYCRVMWG

KAD1_CHICK 4 EKLKHHKIIFVVGGPGSGKGTQ-----------------CEKIVHKYGYTHLSTGDLLRA

H9KZB0_CHICK 236 RPQPRRMAYHYYEPKGPDEC-------------TTYIHNERSRRG----NHHRFITEKRV

10515 85 HSGDTAAGMGHIVPHHPDSCMLPLQDTVLDMLRDAMLAKADSSK**GFLIDGYPR**EVKQGEE

KAD1_CHICK 47 EVSSGSERGKKLQAIMEKGELVPL-DTVLDMLRDAMLAKADTSKGFLIDGYPREVKQGEE

H9KZB0_CHICK 279 FASW--------------AAPYNITFSHPTWP----------------------------

10515 145 FEKKVRAVPLVSAVPCCAVPPYTVLAPWPCWVSHLEEPWGPQTHTVTAVSPQIGPPTLLL

KAD1_CHICK 106 FEKK------------------------------------------------IAPPTLLL

10515 205 YVDAGKETMVKRLLKRGETSGRVDDNEETIKKRLETYYKATEPVIAFYKDRGIVRQVSGA

KAD1_CHICK 118 YVDAGKETMVKRLLKRGETSGRVDDNEETIKKRLETYYKATEPVIAFYKGRGIVRQLN-A

10515 265 QGGGKQYDKMA----QLPTPPP

KAD1_CHICK 179 EGTVDEVFQQVCSYLDKL-----

M is the first residue of new entry 10510b

**11366**

C7G541_CHICK 130 KQIQEEDHRFYEYLQHQKKPITANYIPDSHGNIAHDHLQLWGLAIVGSSHIMWKQSTEHT

11366 1 MPGRNALWYIIFLMNSQHSSFLYDSLSDSYGNIDR**AHIALWGLAICGSSKIMFEHSREDL**

F1P0G7_CHICK 1 --------------------RAMAELPPTHGPASRA-------AALATAYISHRHGSPGH

C7G541_CHICK 190 GYLLAQVSSVKQQIRKDNAVAFKFIVLLHEIPTQQLNVCHMYL//

11366 61 **SYNVAQVKAVK**QEDIADVGHKYHLEFILEDVFEKDSTINCTAEVLYHLGNKNSAPDVQFT

F1P0G7_CHICK 34 GWVLRDVRRARREDIDDIGHKYHLEFVLEDIFEKDSTVNCTAEVLYHLGNEKSAPDVQFT

11366 121 IEGDLKNTDEADNAFYNRIKSLKKELEAENIPDRYGNVSPEIEPIRALAWAASGYVIWQN

F1P0G7_CHICK 94 IEGELKNTDEADNAFYNRIQSLKKELEAENIPDSYGNVSPEMQPIRALAWAASGYVIWQN

11366 181 STEDTKYQLAQIKHVKQVKRTDEYLEFDYVILLHENVSQVRNPLPKKVFFLLYCQLCNFE

F1P0G7_CHICK 154 STENTKYQLAQIKHVKQVKRTDEYLEFDYVILLHENVSQEIIPWQMTV---LWHPQHGIE

11366 241 LGHEAGGVSTDLVPGGQQLQWVR

F1P0G7_CHICK 211 VTQNSCQAKHALD----------

E is the first amino acid of the new entry 11366b

**12006**

E1BZE1_CHICK 1 MKALVAFIL-------LVQLPIHRAAPAAPPPPLGCDDPEIEAAAEFAVIYINGHSHHGY

12006 ------------------------------------------------------------

E1BZE1_CHICK 54 RFALNRIEQVRVLFQGPNNEILFLELDLLETTCHILNPTPLVNCSVRTFAEHAVEGDCD-

12006 1 ---------------GPNNEIMFLELDLLETKCHILNPTPLANCS-------AVEGDCD-

E1BZE1_CHICK 113 -VKLQKVNGQFSVLASKCHSHADSAEDIREVCPNCPLLANLNDTDVLAAVSSALNDYNSK

12006 38 -VKLQK**LNGQFSVLASK**CHSNADSAEDIREVCPDCPLLANLNDTDVLTTVSNALNDYNSK

E1BZE1_CHICK 172 NPDV-YLMLLEIGRAVKQYHPVRMVSVEFAVAATNCTSQQAKDNLAACQLLPEDQSNFGF

12006 97 NPDT-YLRLLEIGRAVKQYHPVRMVSVEFAVAATNCTSQQAKDNLEACQLLPEDQS--AL

F1NHT5_CHICK 1 MLLLISVLFGTQAL

E1BZE1_CHICK 231 CTAKMVTEPSQDLIAECQLYGHQPGVTYPHPGQDTSAGLVPSAGQGFTNHNLKISHNNPV

12006 154 CSWAASAPARELPSVLLSPSCHDAAVEEAADLALDQINADR**TEGYILSLYR**IFSVREHPQ

F1NHT5_CHICK 15 CSWAASPPARELPSVLLSPTCHDAAVEEAADLALRQINADRTEGYILSLYRIFSVREHPQ

E1BZE1_CHICK 291 ASESSSSEFPSLLSAKSVAKRAAAEVAQHDKVPHPVGFVPPPPLCPGKIRHFKI

12006 214 EITGSVFYLILDVVDTECHVLSKRPWKNCRVRPAHTT

F1NHT5_CHICK 76 EITGSVFYLILDVVDTECHVLSKQLWKNCTVRPAHTTVYGQCKAIIYINQSRKIAHLNTY

F1NHT5_CHICK ECNLQPVPPRYIWGVCPDCPVDDCPTEPRYLETAVKSLAKFNEESEQTHYFSVLNVTRGS

F1NHT5_CHICK MQWVIGPAHFVEFLIQETSCSKRDPVADISKCKPLSPELAKIGFCKGSVVNSDMEHKQSV

F1NHT5_CHICK TISCEIYSSQDPVTGEGKLQTNQRPEESSQNHQQAFSLHLGKTTGCVKILPPSTEDISFH

F1NHT5_CHICK KLRESQNEHEDIKSVSAKAIECTSTPPAPDGETTHVDEPGLTKPVTGPVILPFPAELSLS

F1NHT5_CHICK DSCPGEAKQTDSILHPLLTRQRAEV

A is first amino acid of new entry 12006b

**1204**

1204 1 MAGMTIGSTPVSAPPPAGLGYGSFGQVRGPQPNYGGAYPGTPNYGSQPGPPPKRLDPDSI

1204 61 PSPIQVIEDDRSNRGSEPFVTGVRGQVPPLVTTNFVVKDQGNASPRYIRCTSYNIPCTSD

1204 121 MAKQSQVPLAAVIKPLATLPPEENNKFPSPPAFIFMIDVSYNAVKSGLVRLICEELKSIL

1204 181 DYLPREGNMEESAIRVGFVTYNKVLHFYNVKSSLAQPQMMVVSDVADMFVPLLDGFLVNV

1204 241 NESRTVITSLLDQIPEMFADTRETETVFGPVIQAGLEALKAAECAGKLFIFHTSLPIAEA

1204 301 PGKLKNRDDKKLINTDKEKTLFQPQTSFYNNLAKDCVAQGCCVDLFLFPNQYLDVATLGV

1204 361 VTYQTGGSIYKYAYFQLEADQDRFLNDLRRDVQKEVGFDAVMRVRTSTGIRATDFFGAFY

1204 421 MSNTTDVEMAGLDCDKTITVEFKHDDKLSEDSGALLQCALLYTSCAGQRRLRIHNLSLNC

1204 481 CTQLADLYRNCETDTLINYLAKYAYRGVL--SSPVKAVRDSLINQCAQILACYRKNCASP

FUT11_CHICK 1 ----------------------------MGGGGPGRAARRG--PTCLWVTLALAWGAGSR

1204 539 SSAGQTKVDVDSDSLPAAIRNSEERLSKGDIYLLENGLNIFLWSTLPVLENPFSRKVRSI

FUT11_CHICK 31 AAAG----DGDGDGEPGPGDAGTPCGAEG-------------WARAAVPPGPAFVAAASY

1204 599 IDMLHLQRSRYMKALPIVLWWSGSLFPHFPGDTERIDCPRGSCLVTRSRRAARHRRTK**AI**

FUT11_CHICK 74 RGPGN-NDTRSNKALPILLWWSGSLFPHFPGDTERIDCPRGSCLVTRSRRAARHRRTKAL

1204 659 **IFYGTDFRAYEAPLPRLPHQTWALFHEESPMNNYLLSHPPGIR**LFNYTATFRRESHYPLT

FUT11_CHICK 133 IFYGTDFRAYEAPLPRLPHQTWALFHEESPMNNYLLSHPPGIRLFNYTATFRRESDYPLT

1204 719 LQWLPGPGYLR**GPALPLADKDAWR**RRGYGPVLYMQSHCDVPSDRDRYVRELMK**YIQVDSY**

FUT11_CHICK 193 LQWLPGAGYLRGPALPLAEKDAWRRRGYGPVLYMQSHCDVPSDRDRYVRELMKYIQVDSY

1204 779 **GK**CLHNRELPSERLRDTVTATAEDPEFMAFIARYKFHLALENAICNDYMTEKLWRPMHLG

FUT11_CHICK 253 GKCLHNRELPSERLRDTSTATTEDPEFMAFIARYKFHLALENAICNDYMTEKLWRPMHLG

1204 839 AVPVYRGSPAVRDWMPNNHSIILIDDFDSPQELAKYLDFLDKNGEEYMKYLEYKNTDGIK

FUT11_CHICK 313 AVPVYRGSPAVRDWMPNNLSIILIDDFDSPQELAKYLDFLDKNGEEYMKYLEYKNLDGIK

1204 899 NQFLLKSLETREWGVNDMTLPNYLNGFECFICDRENARVRAEQEHKKSHGKTPAPAPHVA

FUT11_CHICK 373 NQFLLESLERREWGVNDMTLPNYLNGFECFICDRENARVRAEQEHKKSRGKTPAPSPHIA

1204 959 HFQHMGCPMPTPGFGSVEDLPAEDR-----------------------------------

FUT11_CHICK 433 HFQHMGCPMPTPGFGSVEDLPGEDSWKEMWLQDYWQSLDQGEALTAMIRHNESHQGRFWD

1204 -------------

FUT11_CHICK 493 YMHEIFLKRTRQH

Y is the first amino acid of the new entry 1204b

**1228**

E1BV78_CHICK 1 MAPGEMGPKPGRWVPLGHLLSLLFSTSMAYIATRENCCILDERFGSYCPTTCGIADFFNK

1228 1 ------------------------------------------MAGSYCPTTCGIADFFNK

E1BV78_CHICK YRLTTDGELLEIEGLLQQATNSTGSIEYLIQHIKTIYPSEKQTLPQSIEQLTQKSKKIIE

1228 19 YRLTTDGELLEIERLLQQAVNSTGSIEYLIQHIKTIYPSDKQTLPH--------------

E1BV78_CHICK EIIRYENTILAHENTIQQLTDMHIMNSNKITQLKQKIAQLESHCQEPCKDTAEIQETTGR

1228 ------------------------------------------------------------

E1BV78_CHICK DCQDIANKGARKSGLYFIKPQKAKQSFLVYCEIDTYGNGWTVLQRRLDGSEDFRRNWVQY

1228 65 -CQDIANKGAR**KSGLYFIKPQK**AKQSFLVYCEIDTYGNGWTVLQRRLDGSEDFRRNWVQY

E1BV78_CHICK KEGFGHLSPDDTTEFWLGNEKIHLITTQSTLPYALRIELEDWSGKKGTADYAVFKVGTEE

1228 124 KEGFGHLSPDDTTEFWLGNEKIHLITTQSTLPYTLRIELEDWSGNKGTADYAVFKVGSEE

E1BV78_CHICK DKYRLTYAYFIGGEAGDAFDGFNFGDDPSDKSYTYHNGMRFSTFDNDNDNFEGNCAEQDG

1228 184 DKYRLTYAYFIGGDAGDAFDGFNFGDDPSDKSFTYHNGMRFSTYDNDNDKFEGNCAEQDG

E1BV78_CHICK SGWWMNRCHAGHLNGQYYIGGVYSRDTGTNSYDNGIIWATWRDRWYSMKKTTMKIIPFNR

1228 244 SGWWMNRCHAGHLNGKYYIGGVYSRDGDPDSYDNGIIWATWRDRWYSMKKTTMKIIPFNR

FIBA_CHICK 1 ------------------------------------------------------MIPVTI

E1BV78_CHICK LSIDGQQHSGGLKQVGDS------------------------------------------

1228 304 LSIDGQQHSGGLKQAQDGETTFEKEGAGVRGPRIVEHMQQSSCKYEKNWPICADDDWGIK

FIBA_CHICK 7 LCV---LLCLNLAWAQDGKTTFEKEGGGGRGPRILENMHESSCKYEKNWPICVDDDWGTK

1228 364 CPSGCR**MQGLIDDTDQDNSHR**IDKIR**QQLADSQNNYK**ASNRV------------------

FIBA_CHICK 64 CPSCCRMQGIIDDTDQNYSQRIDNIRQQLADSQNKYKTSNRVIVETINILKPGLEGAQQL

1228 406 ------------------------------------------------DIDIKIRACKGS

FIBA_CHICK 124 DENYGHVSTELRRRIVTLKQRVATQVNRIKALQNSIQEQVVEMKRLEVDIDIKIRACKGS

1228 418 CARSFDYQVDKEGYENIQKQLTQASSIDMNPDFQATALSTLKMRPLKDSNVPEHFKLKPP

FIBA_CHICK 184 CARSFDYQVDKEGYDNIQKHLTQASSIDMHPDFQTTTLSTLKMRPLKDSNVPEHFKLKPS

1228 478 PEMQALNAFNNIKQMQVVLERPETDHVAEGRGDSSPSHSSKLVTSAHGRDNPSLVDLTSS

FIBA_CHICK 244 PEMQAMSAFNNIKQMQVVLERPETDHVAEARGDSSPSHTGKLITSSHRRESPSLVDKTSS

1228 538 ASSVHRCTRTVTKKVITGPDGPREEIVEKMVSSDGSDCSHLQGGREGSTYHFSGAGDFHK

FIBA_CHICK 304 ASSVHRCTRTVTKKVISGPDGPREEIVEKMVSSDGSDCSHLQGGREGSTYHFSGTGDFHK

1228 598 LDRLLPDLESFFTHDSVSTGSRHSMGSSTSSRVTGAGSSHLSTGGKDKFTDLGEEEEDDF

FIBA_CHICK 364 LDRLLPDLESFFTHDSVSTSSRHSIGSSTSSHVTGAGSSHLGTGGKDKFTDLGEEEEDDF

1228 658 GGLQPSGFAASSASHSKTVVTSSSSSFNKGGSTFETKSLKTREISEQLGGVQHDQSAEDT

FIBA_CHICK 424 GGLQPSGFAAGSASHSKTVLTSSSSSFNKGGSTFETKSLKTRETSEQLGGVQHDQSAEDT

1228 718 PDFQARSFRPAAMSTRRDYNGKDCDDIRQKHTFGAKSGIFKIKPEGSNKVLSVYCDQETT

FIBA_CHICK 484 PDFKARSFRPAAMSTRRSYNGKDCDDIRQKHTSGAKSGIFKIKPEGSNKVLSVYCDQETT

1228 778 LGGWLLIQQRMDGSVNFNRTWQDYKRGFGSVDGKGQGELWLGNENIHLLAQNDTLLRVEL

FIBA_CHICK 544 LGGWLLIQQRMDGSVNFNRTWQDYRRGFGSVDGKGQGELWLGNENIHLLTQNDTLLRVEL

1228 838 EDWDGNAAYAEYIVQIGTEAEGYALAVSSYEGTAGDALVAGWLEEGSEYTSHAQMKFSTF

FIBA_CHICK 604 EDWDGNAAYAEYIVQVGTEAEGYALTVSSYEGTAGDALVAGWLEEGSEYTSHAQMQFSTF

1228 898 DRDQDRWEESCAEVYGGGWWYNSCQAANLNGIYYPGGHYDPRYNVPYEIENGVVWIPFRA

FIBA_CHICK 664 DRDQDHWEESCAEVYGGGWWYNSCQAANLNGIYYPGGHYDPRYNVPYEIENGVVWIPFRA

1228 958 SDYSLKVVRMKIRPLETL

FIBA_CHICK 724 SDYSLKVVRMKIRPLETL

Q is the first amino acid of the new entry 1228b

**1311**

E1C7R1_CHICK 188 TFSQADSSSEMKEEMKDLKNRVEALEQKLQLVLAPFHNLMPSAPEDVGTDPISRLSHSLQ

1311 1 MALASLLITRHASLWSHTPSQEPSSKQK**LQLVLAPFHNLMPSAPEDVGTDPISRLSHSLQ**

E1C7R1_CHICK 248 QLDRIDSLSEQISFLEERLETCSCKNEL

1311 61 **QLDRIDSLSEQISFLEER**LETLINLLLHIRTLLLKPAVRRQPLLTGERALAAVSSDMVIS

E1BV78_CHICK 136 IQQLTDMHIMNSNKITQLKQKIAQLESHCQEPC------KDTAEIEETTGRDCQ/aa184-224/

1311 121 ERQLTDMHIMNSNKITQLKQKIAQLESHCQEPC------KDTAEILETTGRGAQNVSCMV

F1P4V1_CHICK 1 ---------------------MIPVTILCVLLCLNLAWAQDGKTTFEKEGGGGRGPRILE

E1BV78_CHICK 225 RRLDGSEDFRRNW-VQYKEGFGHLSPDDTTEFWLGNEKIHLITTQSTLPYALRIELEDWS

1311 175 ERLDGSEDFRRNW-VQYKEGFGHLSPDDTTEFWLGNEK**IHLITTQSTLPYTLRIELEDWS**

F1P4V1_CHICK NMHESSCKYEKNWPICVDDDWGTKCPSGC--------RMQGIIDDTDQNYSQRI------

/aa330-380/

E1BV78_CHICK 284 GKKGTADYAVFKVGTEEDKYRLTYAYFIGGEAGDAFDGFNFGDDPSGVYS-RDTGN-SYD

1311 234 **GNK**GTADYAVFKVGSEEDKYRLTYAYFIGGDAGDAFDGFNFGDDPSGVYSARDGDPDSYD

F1P4V1_CHICK ------DNIRQQLADSQNKYKTSNRVIV-----ETINILK-----PGLEGAQQLD-ENY-

E1BV78_CHICK 394 NGIIWATWRDRWYSMK-KTTMKIIPFNRLSIDGQQHSGGLK--QVGDS

1311 294 NGIIWATWRDRWYSMK-KTTMKIIPFNRLSIDGQQHSGGLK--QVDIDIKIRACKGSCAR

F1P4V1_CHICK -GHVSTELRRRIVTLKQRVATQVNRIKALQNSIQEQVVEMKRLEVDIDIKIRACKGSCAR

1311 351 SFDYQVDKEGYENIQK**QLTQASSIDMNPDFQATALSTLK**MRPLKDSNVPEHFK**LKPPPEM**

F1P4V1_CHICK SFDYQVDKEGYDNIQKHLTQASSIDMHPDFQTTTLSTLKMRPLKDSNVPEHFKLKPSPEM

1311 411 **QALNAFNNIK**QMQVVLERPETASASSRGETFYHVAEGRGDSSPSHSSKLVTSAHGR**DNPS**

F1P4V1_CHICK QAMSAFNNIKQMQVVLERPETD----------HVAEARGDSSPSHTGKLITSSHRRESPS

1311 471 **LVDLT---SSVHR**CTRTVTKK**VITGPDGPREEIVEK**MVSSDGSDCSHLQGGREGSTYHFS

F1P4V1_CHICK LVDKTSSASSVHRCTRTVTKKVISGPDGPREEIVEKMVSSDGSDCSHLQGGREGSTYHFS

1311 528 GAGDFHKLDR**LLPDLESFFTHDSVSTGSR**HSMGSSTTATSSRVTGAGSSHLSTGGKDKFT

F1P4V1_CHICK GTGDFHKLDRLLPDLESFFTHDSVSTSSRHSIGSS---TSSHVTGAGSSHLGTGGKDKFT

1311 588 DLGEEEEDDFGGLQPSGFAASSASHSKTXXXXXXXXXXXXXXXXXXXXXXXXXXXXXXXX

F1P4V1_CHICK DLGEEEEDDFGGLQPSGFAAGSASHSKTVLTSSSSS------------------------

1311 648 XXXXXXXXXXXXFNKGGSTFETKSLKTREISEQLGGVQHDQSAEDTPDFQARSFRPAAMS

F1P4V1_CHICK ------------FNKGGSTFETKSLKTRETSEQLGGVQHDQSAEDTPDFKARSFRPAAMS

1311 708 TRRDYNGKDCDDIRQKHTFGAKSGIFKIKPEGSNKVLSVYCDQETTLGGWLLIQQRMDGS

F1P4V1_CHICK TRRSYNGKDCDDIRQKHTSGAKSGIFKIKPEGSNKVLSVYCDQETTLGGWLLIQQRMDGS

1311 768 VNFNRTWQDYKRGFGSVDGKGQGELWLGNENIHLLAQNDTLLRVELEDWDGNAAYAEYIV

F1P4V1_CHICK VNFNRTWQDYRRGFGSVDGKGQGELWLGNENIHLLTQNDTLLRVELEDWDGNAAYAEYIV

1311 828 QIGTEAEGYALAVSSYEGTAGDALVAGWLEEGSEYTSHAQMKFSTFDRDQDRWEESCAEV

F1P4V1_CHICK QVGTEAEGYALTVSSYEGTAGDALVAGWLEEGSEYTSHAQMQFSTFDRDQDHWEESCAEV

1311 888 YGGGWWYNSCQAANLNGIYYPGGHYDPRYNVPYEIENGVVWIPFRASDYSLKVVRMKIRP

F1P4V1_CHICK YGGGWWYNSCQAANLNGIYYPGGHYDPRYNVPYEIENGVVWIPFRASDYSLKVVRMKIRP

1311 948 LETL

F1P4V1_CHICK LETL

A is first amino acid of new entry 1311b; Q isthe first amino acid of new entry 1311c

**1506**

1506 1 MKTVFYMCLLLAGLHAFAYGQLTANHHNEHDPNEPNDHIHHSAEAAACLK**LVPNNADFAF**

E1BS56_CHICK 1 MKTVFYICLLLAGLHAFAYGQLTASHHNGHNPNEPKDHMHHNAEAAACLKLVPNNADFAF

1506 61 **KFLNEVALEAPNKNIFFSPVSISAAFAMLAMGAK**SVTKTQILEGLAFNLTEIQEQEIHEG

E1BS56_CHICK 61 KFLNEVAQEAPNKNIFFSPVSISAAFAMLALGARSITKTQILEGLAFNLTEIQEKEIHEG

1506 121 FR**NLMHMLSHPEGGVQLSMGNAIFLTEK**MKPLKKFLDDVKPLYQVEVFATDFNSSIEAKK

E1BS56_CHICK 121 FHNLMHMLSHPESGVQLNMGNAIFLTKKLKPLKKFLDDAKPLYQLEVLATDFNNPTEAEK

1506 181 EINDYVEKKTQGKITNLVDEMDPQTIMLLASFVFFR**GNWEKPFKPENTEEREFFVDAETT**

E1BS56_CHICK 181 EINDYTEKKTQGKITNLVKEIDPQTVMLLASFVFFRGNWEKPFKPENTEEREFFVDAETT

1506 241 **VKVPMMYQVGTFDLYFDKDLPCTVVR**LHYSGSATVFLILPAKGKMK**QLEQTLDKEK**VK**KW**

E1BS56_CHICK 241 VKVPMMCRIGTFDLYFDKDLPCTVVRLHYNGSATAFLILPAKGKMKQLEQTLDKERVKKW

1506 301 **SDHLFR**SK**IQLYLPKFSVSGHYEITNILSKMGIVDVFTNQADLSGIAGVPELK**VSKAFHK

E1BS56_CHICK 301 SDHLFKSKIQLYFPKFSISGTYEITNILSKMGIVDVFTNQADLSGISGVPELKVSKVIHK

1506 361 **AALEVNER**GSEA-AEATSPKMMALTLAPVIEFNHPFLMLIFDR**DTNSTLFIGK**ISNPTTT

E1BS56_CHICK 361 AALDVDERGTEASATAATPKIMALSLAPIIEFNRPFLMLIFDRDTNSTLFIGKIANPTTT

1506 420 STKVSCFHWSKFDPSDRNIIIGLHYDEERESTLLDTNMKTGIYLLFLCEICFEISKADIK

E1BS56_CHICK 421 SRTEI-------------------------------------------------------

G1NKG8_MELGA 1 -------------------------------------MKIGIYLLLLCEICFEISKADIK

1506 480 PKSPKKDKWLYFLGKNKNISISEEWHQHKNDSKPLEEQSFEDLTLHNFTEKTANFGFNLY

G1NKG8_MELGA 24 PKSPKKDKRLNFLGKNKNNSISEEWHQHKNYSEPLEDESFEELTLHNFTEKTANFGFNLY

1506 540 RKIAMKTDNNIIISPFSVSTLMATYLLAAEGETQRQIAKALNLHALKDRDRHYLPALFKQ

G1NKG8_MELGA 84 RKIAMKLDNNIIISPLSVSTLMATYLLAAEGETHRQIAKALNLHPLEDRDRHYLPTLFKQ

1506 600 LKDNITTNEEVLFVQGILSFIQKDFVVREAFLNLSKQYFDMEFLCVDFQNITQAKFVINQ

G1NKG8_MELGA 144 LKDNITTNEELLFVQGILSFIQKDFVVREAFLNLSKQYFDMEFLRVNFQNITQAKSVINQ

1506 660 NVKQRTKGKISELFEEVDRHSKLLLLDYIFFKGKWLYPFNSEFTEIETFHINKYRSVQVP

G1NKG8_MELGA 204 NIKQRTKGKISELFEEVDRHSKLLLLDYIFFKGKWLYPFNSEFTEIETFHINKYRSVQVP

1506 720 MMFKSDKINSTYDENLRCYVIKLPYKGKAYMLIVIPEKGEDYVSLEDHLTMELVESWLAN

G1NKG8_MELGA 264 MMFKSDKVNSTYDENLRCNVIKLPYKGKAYMLIVIPEKGEDYVSLEDHLTMELVESWLAN

1506 780 MKSR**NIDISFPK**FKLEQKYKMKKLLYALGIKNLFARTADLSHLTDQKYITVSQVVQKAVI

G1NKG8_MELGA 324 MKSRNTDISFPKFKLEQKYKMKKWLYALGIKNLFARTADLSHLTDQKYVTVSQVVQKAVI

1506 840 EVDEKGTEAAAATGSEIIAFSAPPVIKVDRPFLFMIFEETFKTLLFIGRVVDPTEL

G1NKG8_MELGA 384 EVDEKGTEAAAATGSEIIAFSAPPVIKVDRPFLFMIFEETFKTLLFIGRVVDPTEM

F is the first amino acid of the new entry 1506b

**15258**

F1NCP0_CHICK 353 MSGFTVLHWLAKHGDGTGLQELAEAARQAGLALDVDARSGCGYTPLHLAAIHGHTLVIKV

15258 1 MSGFTVLHWLAKHGDGPGLQELAKAARQAGLALDVDARSGCGYTPLHLAAIHGHTLVIKV

F1NCP0_CHICK 413 LVLQLGCQVQVRDGSGRRPWEYLGSSTSGEIWQLLQAP

15258 61 LVQQMGCQGLVVTQLDVQPGECVKVKGKIPSDAKGFSVNVGK**DSSNLMLHFNPR**FDCHGD

G1NB72_MELGA 1 -----MEQGLVVTQLDVQPGECVKVKGKIPSDAKGFSVNVGKDSSTLMLHFNPRFDCHGD

15258 121 VNTIVCNSKEDGTWGEEDRKADFPFQHGDKVEICISFDAAEVK**VKVPDVEFEFPNR**LGME

G1NB72_MELGA 55 VNTVVCNSKEDGMWGEEDRKADFPFQHGDKVEICISFDAAEVKVKVPEVEFEFPNRLGME

15258 161 K**IQYLSVEGDFK**VKAIKFS

G1NB72_MELGA 115 KIQYLAVEGDFKVKAIKFS

Q is the first residue of the new entry 15258b

**1577**

Q90815_CHICK 243 MPGGNVNITCVAVGSPMPYVKWMQGAEDLTPEDDMPVGRNVLELTDVKDSANYTCVAMSS

1577 1 MPGGNVNITCVAVGSPMPYVKWMQGAEDLTPEDDMPVGRNVLELTDVKDSANYTCVAMSS

Q90815_CHICK 303 LGVIEAVAQITVKSLPKAPGTPVVTETTATSITITWDSGNPDPVSYYVIEYKSKSQDGPY

1577 61 LGVIEAVAQITVKSLPKAPGTPVVTETTATSITITWDSGNPDPVSYYVIEYKSKSQDGPY

Q90815_CHICK 363 QIKEDITTTRYSIGGLSPNSEYEIWVSAVNSIGQGPPSESVVTRTGEQAPASAPRNVQGR

1577 121 QIKEDITTTRYSIGGLSPNSEYEIWVSAVNSIGQGPPSESVVTRTGEQAPASAPRNVQGR

/aa475-555/

Q90815_CHICK 423 MLSSTTMIIQWEEPVEPNGQIRGYRVYYTMEPDQPVSNWQKHNVDDSLLTTVPRNFEPTT

1577 181 MLSSTTMIIQWEEPVEPNGQIRGYRVYYTMEPDQPVSNWQKHNVDDSLLTTVPR**NFEPMT**

Q90815_CHICK 564 SFTVEGLKPNTEYVFRLAARSALGLGAFTPE-------------------VRERTLQ---

1577 241 **SFTVEGLKPNTEYVFR**LAARSALGLGAFTPEWLKEVWSAVAQSDQVIARVQKYQVFSVQQ

Q90816_CHICK 1 --------------------SALGLGAFTPE-------------------VRERTLQSKP

Q90815_CHICK 602 SILPKNF-----------------------------------------------------

1577 301 ETNPSSLQSDLTRSTAILVSWRPPPAESQNGVLAGYSVYYRALDSEDTELKEVNDIPPTT

Q90816_CHICK 22 SAPPQDIKCVSTRSTAILVSWRPPPAESQNGVLAGYSVYYRALDSEDTELKEVNDIPPTT

Q90815_CHICK ------------------------------------------------------------

1577 361 SQILLESLEKWTEYRITVVAHTEVGPGPESSPVIVR**TDEDVPSAPPR**KVEVEVLNSTAIQ

Q90816_CHICK 82 SQILLESLEKWTEYRITVVAHTEVGPGPESSPVIVRTDEDVPSAPPRKVEVEVLNSTAIQ

Q90815_CHICK ------------------------------------------------------------

1577 421 VFWRSPVQNRQHGQIRGYQVHYVRMENGEARGLPQIKDIMLADAQEMVIAGLQPETAYSI

Q90816_CHICK 142 VFWRSPVQNRQHGQIRGYQVHYVRMENGEARGLPQIKDIMLADAQEMVIAGLQPETAYSI

Q90815_CHICK ------------------------------------------------------------

1577 481 TVAAYTMKGDGARSKPKVVTTKGAVPGKPILSVHQTEENTLLVKWEPPLDAEGQVMGYRL

Q90816_CHICK 201 TVAAYTMKGDGARSKPKVVTTKGAVPGKPILSVHQTEENTLLVKWEPPLDAEGQVIGYRL

Q90815_CHICK ------------------------------------------------------------

1577 541 QFGRK**DVDPLATLEFSALEDK**YTAPSIHKGATYVFKLAVKSR**AGFGEEAVQELTTPEDIP**

Q90816_CHICK 261 QFGRKDVDPLATLEFTALEDKYTAPSIHKGATYVFKLAVKSRAGFGEEAVQELTTPEDIP

Q90815_CHICK 609 KVKMVTKTSVLLSWEFP----------ENYNSPTPYKIQYNGLNVDVDGRTTKKLITNLK

1577 601 **K**GYPQILEASNVTSMSVQFGWLPPVLAERNGAIVKYTIQYNGLNVDVDGRTTKKLITNLK

Q90816_CHICK 321 KGYPQILEASNITSMSVQFGWLPPVLAERNGAIVKYTVAYRE-----AGSPGNPLEKDLP

Q90815_CHICK 659 PHTFYNFVLMNRGNSMGGLQQNVAAWTAANMLSRKPEVTHKPDADGNVVVILPDVKSSVA

1577 661 PHTFYNFVLMNRGNSMGGLQQNVAAWTAANMLSRKPEVTHKPDADGNVVVILPDVKSSVA

Q90816_CHICK 376 PSPENSY-------TLNGLKPN-----TAYDVKIRAHTSKGPGP----------------

Q90815_CHICK 719 VQAYYIVVVPLRKSRGGQFLNPLGSPEEMDLEELIQDIARLRRRSLRHSRQLDFPKPYIA

1577 721 VQAYYIVVVPLRKSRGGQFLNPLGSPEEMDLEELVQDIARLRRRSLRHSRQLDFPKPYIA

Q90816_CHICK ----YSPTVQYRTFQLDQVL-----PKNFKVKMVTKTSVLL-------------------

Q90815_CHICK 779 ARFRSLPNHFVLGDMKHYDNFENRALEPGQRYVIFILAVLQEPEATFAASPFSDPIQLDN

1577 781 ARFRSLPNHFILGDMKHYDNFENRALEPGQRYVIFILAVLQEPEATFAASPFSDPIQLDN

Q90815_CHICK 839 PDPQPIIDGEEGLIWVIGPVLAVVFIICIVIAILLYKN/

1577 841 PDPQPIIDGEEGLIWVIGPVLAVVFIICIVIAILLYKK

E is the first residue of new entry 1577b; Y is the first residue of the new entry 1577c

**15969**

15969 1 MRVFCTATMLNEVTIEEMRSGVEKSKTERTEMEYQSNNTNCNSDRIPLRSVLLPVVTSLH

G1N0M4_MELGA ------------------------------------------------------------

15969 61 IWQPNWFYTKLIIYLNCPYFSNLGIRMLWEIVLKALLESSYKMLKKKSHFILQSIGCDDY

G1N0M4_MELGA 1 ------------IFLKYKFISRLAARE------AALLRSSC----------LQSIGCDDY

15969 121 LGSDKVIDKCGICGGDNTACKVVSGVFK**HTLTNLGYHKIVEIPEGATK**INITEMSKSNNY

G1N0M4_MELGA 33 LGSDKVIDKCGICGGDNTACKVVSGVFKHTLTNLGYHKIVEIPEGATKINITEMSKSNNY

15969 181 LVH--------IPEDWEN------------------------------------------

G1N0M4_MELGA 93 LALRSRSGRSIINGNWAIDRPGRYEGGGTMFTYKRPNEISSTAGESFLADGPTNEVLDVY

S is the first residue of the new entry 15969b

**1890**

1890 1 MSRVFELEEKRKKEQLLAKRIELKHDRKARAMASRTKDNFYGYNGIPVEEKPKKRRRTCE

1890 61 NVSQSSEAEYATENEAEQLEFTQTESEYEQEEYDEKPSKAAGKPKAPAKSAPAPLNFADL

1890 121 LRLAEKKQYEPVEIKVVKKIEERPRTAEELREREYLERKNKRVEMQKKKTEREVKNAGIA

1890 181 SSSKKATSLKECADARLSKSAADKHASPKSSLSSLSGIGRPPVGYKRQIDDDDEDDEYDS

1890 241 EMDDFIEDEGEPQEEISKHIREXXXXXXXXXXXXXXXXXXXXXXXXXXXXXXXXXXXXXX

1890 301 XXRKRYKDESDYALRYMESSWREQQKEEARRSDVTPLKITAFSPSKGFTRKIIGETDIKS

1890 361 KSKIGGVRNGGCFNKVTVVGAGDLGIACVLAVVAKGVADKVVLLDLSEGAAKGGTMDLEI

1890 421 FALPNVEISKVEIMTYVSWKLSAFPKSRVIGVGGNLDTERFQYILTKLLQAEAVGKDAWI

1890 481 VGEQGEDKVPSWTSSKSVTNETETVAARNSREKGANRAVEVLKGKGQRSWSVGLSVADLT

1890 541 DSIMKDKRKVHSVSTLAKYKYR**DLTIQETNSVISQYKDLKPVMDSYVFNDGSSRELMSLS**

E1BWW2_CHICK 1 ----MAFSESQLKKMLAKYKYRDLTIQETNSVISQYKDLKPVMDSYVFNDGSSRELMSLS

1890 601 **GTIPVPYR**G---------------------------------------------------

E1BWW2_CHICK 57 GTIPVPYRGNVYNIPICLWLLDTYPFNPPICFVKPTSSMTIKTGKHVDANGKIYLPYLHE

1890 ------------------------------------------------------------

E1BWW2_CHICK 117 WKYPQSDLLELIQVMIVVFGEEPPVFSRPTVSASYPPYQATGPPTTSYVPGMPGISPYPT

1890 610 -----------------------------------------PSR**DGTISEDTIRASLISA**

E1BWW2_CHICK 177 GSTANPSQSSFPSYPYPAGVPFPPTTNVPYYPSQPPVTTVGPSRDGTISEDTIRASLISA

1890 629 **VSDKLR**WRMKEEMDRAQAELNALKRTEEDLKKGHQKLEEMVTR**LDQEVAEVDKNIELLK**K

E1BWW2_CHICK 237 VSDKLRWRMKEEMDRAQAELNALKRTEEDLKKGHQKLEEMVTRLDQEVAEVDKNIELLKK

1890 689 KDEELSSALEK**MESQSENNDIDEVIIPTAPLYK**QILNLYAEENAIEDTIFYFGEALRRGV

E1BWW2_CHICK 297 KDEELSSALEKMESQSENNDIDEVIIPTAPLYKQILNLYAEENAIEDTIFYLGEALRRGV

1890 749 IDLEVFLKDADGTSHSNC---THTNHADLIVGMSVLLFVDIVDEMILERHGAQLKEKQRI

E1BWW2_CHICK 357 IDLDVFLKHVRLLSRKQFQLRALMQKARKTAGLSDLY-----------------------

1890 806 ISAGFGAS

E1BWW2_CHICK --------

M is the first amino acid of the new entry 1890b

**1989**

1989 1 MEPESCRRAAPAEDAVRYRLFALPGAGGRERLRRCNDGIAERLAPLLAAYIWQRQPFRLR

1989 61 YVPPCGDTPEHIGGTTLFGDNVEDEWFIVYLVREISRAFPELVARIDDSDGEFLLIEAAD

1989 121 FLPEWLSPENSENRVFFYKGELHIIPLSETEEQQWEELPAESLTISKALALLSAHSEEFL

1989 181 AAEPIRRAVYKRISGCVRGIVAVLKQRPSLVAAAVQAFYLRDPTDLRACRSFRTFPPEER

1989 241 VMTTVTFTKCLYAQLVQQKFAPDRRSGYTLPPPSHPQYKAHELGMKLGEMEGSAKYLELL

1989 301 RMAEHYFQQSVLRPESWLEISPDALDQMLKEATVESHPTSNEEEQKYDLEAVAESMKAFV

1989 361 SKVSTHKGAEVPWSSDETNVTFDVDSFTKALDRILEELDSDDLDEEEEFDFSDEDEDDLD

1989 421 AENRREDDEVSAHELIGSLKSYMDEMDRELAQTNVGKSFTTQKKAASSAKATISQNGGLD

1989 481 SEEEDAGLTPVDVDMNLVTNLLESYSAQAGLAGPTSNILQSMGVYLPENTDRASSDTVED

1989 541 GQLTCDEDLQPAVFAMSYPGYPPAGGYPPAPPGGSPWGGAAYPPSNPPSNPPPIGLENVA

Q5ZLG6_CHICK 1 ----MPTYPPVPAVNPSMPSYPGPTGPTVSPGAYGNRGTITDALGFDPLKDAEVLRKAMK

1989 601 GYANQ--------------------FNPNYMAGMDLIKDLKSELSGNFEKTILAMMK**TPV**

Q5ZLG6_CHICK 57 GLGTDEQAIIDCLGSRSNKQRQQIILSFKTAYGKDLIKDLKSELSGNFEKTILAMMKTPV

1989 641 **MFDAYEIK**EAVK**GVGTDENCLIEILASR**SNEHIQELNRVYK-------------------

Q5ZLG6_CHICK 117 MFDAYEIKEAIKGIGTDENCLIEILASRSNEHIQELNRVYKAEFKKTLEEAIRSDTSGHF

1989 682 ----------------------------------------------------------**AV**

Q5ZLG6_CHICK 177 QRLLISLSQGNRDESTNVDMSLVQKDVQELYAAGENRLGTDESKFNAILCARSRAHLRAV

1989 684 **FSEYQR**MCNRDIEKSICREMSGDLEKGMLAVVKCLK**NTPAFFAER**LRNAMKGAGTKDRTL

Q5ZLG6_CHICK 237 FSEYQRMCNRDIENSICREMSGDLEKGMLAVVKCLKNTPAFFAERLRNAMKGAGTKDRTL

1989 744 IRIMVSR**SEVDLLDIR**AEYKRMYGK**SLYADITGDTSGDYRK**ILLKLCGGND

Q5ZLG6_CHICK 297 IRIMVSRSEVDLLDIRAEYKRMYGKSLYADITGDTSGDYRKILLKLCGGND

**M is the first amino acid of the new entry 1989b**

**2298**

Protein sequence 1 TPVPPSARAAGNXPQQ.

2298 1 *MRATLFCLCLCLLGTVLP*TPVSLPAR**ARGNCPGQHQILLK**GCNTKHGFYIFQYIYSHLMQ

OC116_CHICK 1 MRATLFCLCLCLLGTVLPTPVSLPARARGNCPGQHQILLKGCNTKHGFYIFQYIYSHLMQ

2298 61 KNQTQVKKEEGDHQGTIHGHWLGKVDGEAPGQGVGSSHVPEDKDSPKPHSHITPASKGEG

OC116_CHICK 61 KNQTQVKKEEGDHQGTIHGHWLGKVDGEAPGQGAGSSHVPEDKDSPKTHSHITPASKGEG

2298 121 RALRPGIGDSNSVYPTSTSVEGSGDMGSILLGEIINGEDGLPHSTHPGGPHGDGDGGNGV

OC116_CHICK 121 RALRPGIGDSNSVYPTSTSVEGSGDMGSILLGEIINGEDGLPQSTHPGGPHGDGDGGNGV

2298 181 LVDGAVTAGRERASGSKGAGSEGGSHATVPDQGQAGTMGTGDSAITSVTDSAITSVTKKE

OC116_CHICK 181 LVDGAVTAGRERASGSEGAGSEGGSHAPVPDQGQAGTMGTGDSAITSVTDSAITSVTKKE

2298 241 DVHVDTEGIDEFAYIPDVDAVTITRGQDGETHISPEDEVKIFIGRANIQVGENDSSVGSA

OC116_CHICK 241 DVHVDTEGIDEFAYIPDVDAVTITRGQDGETHISPEDEVKIFIGRANIQVGENDSSVGSA

2298 301 GATSEANVIPTVVTVRPQGHPEESATMATLHHGDSVTSRPVGHPSVGNSGDGATEIHSGQ

OC116_CHICK 301 GATSEANVIPTVVTVRPQGHPEESATMATLHHGDSVTSRPVGHPSVGNSGDGATEIHSGQ

2298 361 ELEAPSPWESTGGDATVTMAVGVQSGKGRSGQRALGKHSLPATMTTRGGRGTASSGLTTG

OC116_CHICK 361 ELEAPSPWESTGGDATVTMAVGVQSGKGRSGQRALGKHSLPATMTTRGGRGTASSGLTTG

2298 421 DCSTAASTPSRKGSHVVTAGQGESGEVGTAGPERQRSRVQQEVAPARGVVGGMVVPEGHR

OC116_CHICK 421 DCSTAASTPSRKGSHVVSAGQGESGEVGTAGPERQRARVQQEVAPARGVVGGMVVPEGHR

2298 481 ARVQQEVAPSRGVVGGMVVPEGHRARTQPEVASAPSTVGKAAPERHRNRAQQEVAPVPSM

OC116_CHICK 481 ARVQQEVAPARGVVGGMVVPEGHRARTQPEVASAPSTVGKAAPERHRNRAQQEVAPVPSM

2298 541 VVETVAPERHRARVRPESARLGQAARPEVAPAPSTGGRIVAPGGHRARVWPGAAPAPGVV

OC116_CHICK 541 VVETVAPERHRARVRPESARLGQAARPEVAPAPSTGGRIVAPGGHRARVWPGAAPAPGVV

2298 601 GVARPAPSKAYNGDKRVAIGKSTDVPRDPWVWGSAHPQAQHTRGSTVAGGFAHLHRGQRL

OC116_CHICK 601 GVARPAPSKAYNGDKRVAIGKSTDVPRDPWVWGSAHPQAQHTRGSTVAGGFAHLHRGQRL

2298 661 GGLTEMEHSRQVEQVRHADRLRLHERAVYGLSGVGGPLQPPAVHTDPWSADSSQSSEGRW

OC116_CHICK 661 GGLTEMEHSRQVEQVRHADRLRLHERAVYGLSGVGGPLQPPAVHTDPWSADSSQSSEGRW

2298 721 GSHSDSREEDGEVRGYPYGRQSL

OC116_CHICK 721 GSHSDSHEEDGEVRGYPYGRQSL

**G is the first amino acid of the new entry 2298b**

**2308**

2308 1 MAQLSSSNGFKQRSSPSLATARSKDVDKEEALQMEAEALAKMQKERGIAGSLPIYPPVLT

2308 61 PELAKVFDKIASTSEFLRNGKSSTDLEMTAIKSAVSSLPTSEKCRDISKFDWLDLDPLSK

2308 121 PKVDSVETFYKAEDRGEMASSMTAEDPWDAVLLEEKLLVTCHLERKINGKSSGATVTRSQ

2308 181 SLNMRTTQIGKSQGQTSQEEDDRTPIDLTKHLYKVEKPFREPNIRSSIEELLDIYHKQTE

2308 241 IALKNEIAVLWDKAPATLDLITSESYFRDGQFADQEVRSTAVNWIETLSDDELTDFLPQF

2308 301 VQALKYETYLDSALVKFILARALGNIRIAHYLYWLLKDTLYDPKFGIRYEQILGAFLSVC

2308 361 GKGLREELEKQTRLVLLFGMVAEKVKQTSVSGRQVGEDLRQDMLALQMIKIMDKIWLQEG

2308 421 LDLRMVIFKCLSTGKDRGETPDVAYNFCACLSPCPNFYRAHRENFYSKADSGNTGSPKAF

2308 481 LIRNVSTQKDRAPFVLTSDMAYVINGGEKPTIRFQLFVDLCCQAYNLIRKHANLFLNLLS

2308 541 LMLSSGLPELSGVQDLKYVQDALQPQTTDAEATIFFTSFPNKMVLGRTHIKDVAAKRKVE

RS13_CHICK 1 --------------------------------------------MGRMHAPG--------

2308 601 LNSYIQSLMNSSPEVAEWLK**LTSDDVKEQIYK**LAKK**GLTPSQIGVILR**DSHGVAQVRFVT

RS13_CHICK 9 -KGLSQSALPYRRSVPTWLKLTSDDVKEQIYKLAKKGLTPSQIGVILRDSHGVAQVRFVT

2308 641 GNKILRILKSK**GLAPDLPEDLYHLIK**KAVAVRKHLERNRK-RDVVIEINSDEPQQTISVH

RS13_CHICK 68 GNKILRILKSKGLAPDLPEDLYHLIKKAVAVRKHLERNRKDKDAKFRLILIESRIH-RLA

2308 700 GYLQTLTQQSVALRSHCSLPCCALA

RS13_CHICK 127 RYYKTKRVLPPNWKYESSTASALVA

**M is the first amino acid of the new entry 2308b**

**2565**

2565 1 MGNTLRGTREGAEIPGAGDEPLFSPATLIHHYLGIFRIFAKSKLSKLKRFLTTLQQFGND

2565 61 ISPEIGERVRTLVLGLVNSTLTIEEFHAKLQEATNFPLRPFVIPFLKANLPLLQRELLHC

2565 121 ARMAKQSPAQYLAQHEQLLLDANASSPIDSSELLLEVSESGKRRTPDSKRPCTMSPAQRY

2565 181 SPSNGLSHPPNGLPHPPGPPPQHYRLEDMAMAHHYRDAYRHADPRERPRPEEAVNEVKRQ

2565 241 AMSELQKAVSDAERKAHELITTERAKMERALAEAKRQASEDALTVINQQEDSSEELEAIK

2565 301 ARVREMEEEDKRLKELQLEAESCLLMGSEAGLDGSIGLDGAGAVGSAGT-------CWVG

F1NW57_CHICK 1 -------------------------------------LARRAAVAMARAVLVPLWLCWAL

2565 354 GGWDPQESYGKLM---GWGDLGAFGEPSKETPNLDQMASEGMIFLDFYAANPLCSPS---

F1NW57_CHICK 24 GCAAPPNVVLLLMDDMGWGDLGAFGEPSKETPNLDQMASEGMLFLDFYAANPLCSPSRAA

2565 408 ----------------------YTPQDIVGGIQDSEILLPELLKKAGYTNKIVGKWHLGH

F1NW57_CHICK 84 LLTGRLPVRNGFYTTNAHARNAYTPQDIVGGIQDSEILLPELLKKAGYTNKIIGKWHLGH

2565 446 RPQFHPLKHGFDEWFGSPNCHFGPYDNR**ARPNIPVYRDWEMIGR**YYEDFKIDLKTGESNL

F1NW57_CHICK 144 RPQFHPLKHGFDEWFGSPNCHFGPYDNRALPNIPVYRDWEMIGRYYEDFKIDLRTGEANL

2565 506 TQIYLQEALDFISKQQASQQPFFLYWAIDATHAPVYASKHFLGTSQRGRYGDAVREIDDS

F1NW57_CHICK 204 TQIYLQEALDFISKQQASQQPFFLYWAIDATHAPVYASKHFLGTSQRGRYGDAVREIDDS

2565 566 IGKILKHLQKLGISENTFVFFTSDNGAALISAPKEDVRGLAGILGTAYGNKGDKSLRTVA

F1NW57_CHICK 264 VGKILKHLQKLGISENTFVFFTSDNGAALISAPKQGGSNGPFLCGKQTTFEGGMREPAIA

2565 626 W-----------------------------------------------------------

F1NW57_CHICK 324 WWPGHIPAGSVSRQLGSVMDLFTTSLSLVGLQPPSDRQIDGIDLLPAILQGKLIDRPIFY

2565 627 ------------------------------GIDFCPGQNVSGVTTHSQEDHSTLPLLFHL

F1NW57_CHICK 384 YRGNEMMAVRVGLYKAHYWTWSNSWEDYSKGIDFCPGQNVSGVTTHLQEEHSTLPLLFHL

2565 657 GR**DPGEKYPLSFASDEYQGVMR**RISAVVQQHKETMVPGVPQLNVCDKAVMVSS-------

F1NW57_CHICK 444 GRDPGEKYPLSFASDEYQGVMRRISAVVQQHKDTMVPGVPQLNVCDKAVMNWSPPGCEKL

2565 ------------------

F1NW57_CHICK 504 GKCLKPPKSDPKKCFWSH

**M is the first amino acid of the new entry 2565b**

**2574**

2574 1 MSLKAAFLLLLLVVAGTRADVSPDQVATVIWK**YFTELGSNAKETADQLQQAEITKQLNTL**

O93601_CHICK 1 MSPKAALLLVLLAVAGTRADVSPDQVATVLWRYFTELGSSAKETVDQLQQAEITKQLNTL

2574 61 **LQSNLQSMNSYAK**ELQR**SLVPFATELQAQLVQDSQR**LKEQIQRELAELQAK**LAPYADEVH**

O93601_CHICK 61 LQSNLQSANSYAEELQRRLVPFATELQAQLVQDSQRLKKQIQQELAELQAKLAPYADEVH

2574 121 **QQIGTNIR**ELQAK**LSPYADELR**SQVDRGTGELR**KALEPFATELR**ER**LQDNAGSIQASLGS**

O93601_CHICK 121 QQIGTNIRELQAKLSPYADELRSQVDRGTGELRRALEPFATELREKLQDNADSIQASLGP

2574 181 **FSER**LQQK**IDSSVEGLKGQLTPLADDLKEQVAQSVEGLRKGLSPYAQEVQDGLNR**QLESL

O93601_CHICK 181 YAERLQQQIDSSVEGLKGQLTPLADELKEQVAQSVEGLRKGLSPYAQEVQDGLNRQLQSL

2574 241 TTQMERAAEELRSRLAASSEEMRAQLSSQAQELQQALRGDAEAVRQRLTLLAQQLDER**LV**

O93601_CHICK 241 TAQMERAAEELRSRLAASSEEMRAQLSPLAQELQEALRGDAEAMQQRLAPLAQQLDERLA

2574 301 **QTVEAFRQQVAPTSETFGQQLVK**RLEEMK**QKLESGTAGVEDHLDLLEK**ESCSSLAQQIMS

O93601_CHICK 301 QTVEAFRQQAAPISETFRQQLVQRLEEMKQKLESGTAGVEDHLDLLEKEVREKVATFLST

E1BT17_CHICK 1 ----------------------------------------------------------MS

2574 361 LKAALLLTLLATLPVSPAELARSGFWEYLSQLTSDKDSLEQAQGSKLGREFTNLKESLQD

O93601_CHICK 361 TEQAES------------------------------------------------------

E1BT17_CHICK 3 LKAALLLTLLATLPVSPAELARSGFWEYLSQLTSDKDSLEQAQGSKLGREFTNLKESLQD

2574 421 RGSNVGNFLEKLAPLNRGIQPRLYHDSDSLRKLIRKELDSLRVKLSPYVDDVHHRVGKHL

E1BT17_CHICK 63 RVSNVGNFLEKLAPLHRGIHPRLYHDSDSLRKLIRKELESLRVKLSPYVDDVHHRVGKHL

2574 481 EDLRYQLQPFTEELLDQVSLRARELQRHLTPSRDVAAQLLDGVDEVQRFMAHYADKIAFH

E1BT17_CHICK 123 EDLRYQLQPFTEELLDQVSLRARELQRHLTPSRDVAAQLLDGVDEVQRFMAHYADKIAFH

2574 541 TDQVKDIFQPYADRLLSEIQRSVEELHRNVVPHSPASPEQLNQHIGELSAKLTQNARDLH

E1BT17_CHICK 183 TDQVKDIFQPYADRLVSEIQRSVEELHRNVVPHSPGSPEQLNQHIRELSAKLTQNARDLH

2574 601 RNIQRNLEQLKAKLSLHPGGPGEHHAEEMASEVQQRIEEFRRETYRQIQDFTRAVHQETE

E1BT17_CHICK 243 RNIQRNLEQLKAKLSLRPGGPGERYAEEMASEVQRRIEEFRRDTYLQIQDFTRAVHQETE

2574 661 DMRLKLSARPQYLEEAAGSPAPLEDLRASLDALWRDLAYSLSERGGEAA

E1BT17_CHICK 303 DMRLKLSSRPHYPEEAAGSPAPLEDLRASLDALWRDLSHSLSERGGDAP

M is the first amino acid of new entry 2574b

**2706**

2706 1 -----------------------------------------------MASLEESFRK**FAI**

E1C6P4_CHICK 1 MADNKAKSTKPANKTPPRSPSDPTKDRAAKRLSCDSNSSHEGAMAGEISALEEAFRKFAI

2706 14 **YGDTK**ATGQEMNGKNWAKLCKDCKVIDGK**SITGTDVDIVFSK**VKGKTAR**VINYEEFKKAL**

E1C6P4_CHICK 61 HGDTRATGKEMHGKNWSKLCKDCQVIDGKNVTITDVDIVFSKIKGKSSRTITFEQFKEAL

2706 74 **EELAPK**RFKDK**SKEEAYEAICQLVAGKEPINVGVTK**AKNVGAVERLTDTSKYTGSHKERF

E1C6P4_CHICK 121 QELSKKRFKEKSDEEAIQEIYKLIEGKAPIISGVTKAISSPTVSRLTDTSKFTGSHKERF

2706 134 DETGKGKGKSGR**ENIVDNSGYVSAYK**NAGTYDAKVKNIPPEAALVQLVEPLVGKANGKCR

E1C6P4_CHICK 181 DPSGKGKGRAGREDLVDASGYVSGYKHAGTYDHKVQGSK---------------------

2706 194 VQWNIRIKRRVASALRGLHDRTPLGLVDTPLLDTEEEVNYCFLPLDLVRKYPVLVNDDNL

2706 254 LWLLENAALIECDYSEFRFIVNFLRSKKMLLPDDFSNIDALEEEALALGIPELTEAVRIC

2706 314 RDAEWTAEVSVCTLHQIVKVYVGSNWYETYLQTLLKAKPPKMAPQRGGTKRKNAAEGYDE

2706 374 SSPSTAKSSLRLESPPRKRGMRSSLRKQAENKDSSIDILKLLSLVKEWGTLNSKRRDSQH

2706 434 VEVADGCVTGTVPPHGTAEQDGAVRASIACSAGRMSFAIQENEPKCGAGEKQLGQNVSKV

2706 494 SLPVTPSTGMHQFNTIKTTLLTGKSIWNVNREPAEGFATEPRDGQRTRALQEAHRNVGVI

2706 554 LKVQHPPVIGSDGCRTWHEESIIYSTLMGGIQLEKSNPQGLPQDTIFLRFALSHEEMFYA

2706 614 RKCHFFLTDVILDSIRQKDPKEITAKVMTLVNRLWTQQITPKEFVGDLLSTEYFKGDRNI

2706 674 REQLLKWVEVSSTRHKVML

**A is the first amino acid of the new entry 2706b**

**323**

323 1 MDSSAAAASFLVSSRQPWALVGILGGDPLSTVEDKREYHGNGTTGLAVAQREFLTYDGTR

323 61 FTVTAFSGWIKGSPLYLHIGEVIDGIDMRAEVGLLTRNILIQGEMEDSCYGENQCQFFSF

323 121 DTFGGHIKILANFSSVHMSGVELKNMGQQILGSYPVHFHMAADVDERGGYQRPTYLDNLA

323 181 IHHCFSRAVSTFWIANPNNNLIENAAAGAQAGLFIGKGVKTTRASAEDPREYLTVDNARF

323 241 RPHQDADPEKPRVPAVIDGLIAFKNNDHGAWARGGDIIFHNSGAIGFFMKNSWQISPQNN

323 301 VSQILMEKSVGLKVFFGREGQWFGNNDNDGDKMSVFHDLDGSVTGYSNIFVGRADNYLLR

323 361 HPGCVTVPRWNGVMCTGRYAQQSQPVVMLEQAYIIQWDGRAPEDIILYPINFNRGDWLHI

323 421 ALCYPRNTVFHVVKDIYQRRTGMVHSIKHYLMAPSRDSVLNGTGEQLFYFDRALGPGLFF

F1P1G6_CHICK 1 -------------------------------MAPPTSA----------YLAESL---VFF

323 481 VVLDACSGAVVSRRHFDTVTGENVTNSITDYVQTFIKERDIGDKVWSSDMSVFTRLGSAK

F1P1G6_CHICK 17 L-----------------------------FVASFVST----------------------

323 541 PIVFHREEAVVACPDKDPELEIWNPGHNQDNHIEIRNGRK**LLLSSSATVHSIHIMDGGKL**

F1P1G6_CHICK 26 ------AEAAVACPDKDPELEIWNPGHNQENHIEIRNGRKLLLSSSATIHSIHIMDGGKL

323 601 **IIKDDVQPIVLRTRYILIENDGELRIGSEMCPYQGNVVIILYGR**ADDGTELNPYFGRK**YI**

F1P1G6_CHICK 80 IIKDDVQPIILRTRYILIENDGELRIGSEMCPYQGNVVIILYGRADDGSEANPYFGRKYI

323 661 **GVSEGGTLEIHGK**KKLSWTFLNK**TLHPGGMEEGGYYFER**SWGHR**GVIVHVIDPKVGAVVH**

F1P1G6_CHICK 140 GVSEGGTLEIHGKKKLSWTFLNKTLHPGGMEEGGYYFERSWGHRGVIVHVIDPKMGAVVH

323 721 **SDRFDTYR**AKEESKR**LAQYLDRVANGMILSVAVNDEGSR**NLDDSARKAMTKLGSK**HFLHL**

F1P1G6_CHICK 200 SDRFDTYRAKEESKRLAQYLDRVANGMILSVAVNDEGSRNLDDSARKAMTKLGSKHFLHL

323 781 **GFRHPWSFITVKGNPSSSVEDHIEYQGHK**GSAVAKVFKLFKAENGELFNVSSTSEWVQDV

F1P1G6_CHICK 260 GFRHPWSFITVKGNPSSSVEDHIEYHGHKGSAVAKVFKLFKAENGELFNVSSTSEWVQDV

323 841 EWTEWFEKPEKARSKDMEKLSDFKAAHPDKICR**QPIDIQAETLDGTILTTEVFYK**NGHDY

F1P1G6_CHICK 320 EWTEWFEKPEKARSKDMEKLSDFKAAHPDKICRQPIDIQAETLDGTILTTEVFYKNGQDY

323 901 RFLCHGKDQSGEGCQNYRVRFLCGSS----------------------------------

F1P1G6_CHICK 380 QFLCHGKDQTGEGCRNYRVRFLCGSSVKPKLTVTIDTNVNSTVLNLVDDVSSWEPGDRIV

323 927 ------------------------------GK**AAYLHVGEVIDGVDMRAEVGLLSRNVVI**

F1P1G6_CHICK 440 VASTDYSMYQAEEFQVLPCPSCRPTQVKVAGKAAYLHVGEVIDGVDMRAEVGLLSRNVVI

323 957 **MGEMER**QCYEYSSK**LCSFFDFDTFGGHIK------ATHLEGLELKYMGQQTMGHYPIHFH**

F1P1G6_CHICK 500 MGEMEGQCYEYSSKLCSFFDFDTFGGHIKIGLDFKATHLEGLELKYMGQQTMGHYPIHFH

323 1011 **MAGDVDEK**GGYNPPT---------------------------------------------

F1P1G6_CHICK 560 MAGDVDEKGGYNPPTYVKDVSIHHTFSRCVTIHGSNGLLVKDVVGYDALGHCFFTEDGPE

323 ------------------------------------------------------------

F1P1G6_CHICK 620 ERNTFDHCLGLLVKPSTLLPSDRDSRMCKLITEGAYPGYIPKPRQDCSAVSTFWIANPHN

323 ------------------------------------------------------------

F1P1G6_CHICK 680 NLINCAAAGSEETGFWFVLHHVPTGPSAGMYSPGYSEHMPMGRFSNNRAHSNYRAGMIID

323 ------------------------------------------------------------

F1P1G6_CHICK 740 NGVKTTPASAKDKRPILTLISGRYGPHKDADPLKPREPAIIERFIAYKNQDHGAWLRGGD

323 1026 -------FADNGIGLTLASGGTFPHDDGSKQEIK**NSLFVGESGNLGTETMDNEIWGPGGL**

F1P1G6_CHICK 800 VWLDNCQFADNGIGLTLASGGTFPHDDGSKQEIKNSLFVGESGNLGTEMMDNEIWGPGGL

323 1079 **DHRGRTLPIGPNFPIR**GIQFYDGPINVQNCTFR**KFAALDGRHTSALAFR**LNNAWQSCPNN

F1P1G6_CHICK 860 DHRGRTLPISPNFPIRGIQFYDGPINVQNCTFRKFAALDGRHTSALAFRLNNAWQSCPNN

323 1139 NVTDIHFEDVPITSR**VFFGEPGPWFNDLDMDGDKTSVFHDVDGSVSEYPGSYLIKEDNWL**

F1P1G6_CHICK 920 NVTDIHFEDVPITSRVFFGEPGPWFNDLDMDGDKTSVFHDVDGSVSEYPGSYLIKEDNWL

323 1199 **IKHPDCIDVPDWR**GSICSGHYAQ-------PANLRMKIIK**NDYHNHPLYLEGALSKSTHY**

F1P1G6_CHICK 980 IKHPDCIDVPDWRGSICSGHYAQIYIQAYKPANLKMKIIKNDYHNHPLYLEGALSKSTHY

323 1252 **QQYQPVITLRKGYTIHWDKTAPEELTIWLINFNKNDWIQVGFCYPKGTTFSILSDIHNR**L

F1P1G6_CHICK 1040 QQYQPVITLRKGYTIHWDKTAPEELTIWLINFNKNDWIQVGFCYPKGTTFSILSDIHNRL

323 1312 LKKTIKTGTFYR**VSQMEKLEYRYPSKGYYYWDEDTGLLFLK**LKAQNEKEKFAFCSVKGCE

F1P1G6_CHICK 1100 LKKTIKTGTFYRVSQMEKLEYRYPSKGYYYWDEDTGLLFLKLKAQNEKEKFAFCSVKGCE

323 1372 RIRIKAMIPK**TAGVSDCQAMAYPKYIETPVVEVPMPK**KLSSVQLK**TKDHLLEVK**IETYKK

F1P1G6_CHICK 1160 RIRIKAMIPKTAGVSDCQATAYPKYIETPVVEVPMPKKLSSVQLKTKDHLLEVKIETYKK

323 1432 QYFHLK**DDYAYIEVDGVR**YFLTEEGIQLIVIDGHHGK**VVDRVTFRNSILQGIPAQIENYV**

F1P1G6_CHICK 1220 QYFHLKDDYAYIEVDGIRYFLTEEGIQLIVIDGHHGKVVDRVTFKNSILQGIPAQIENYV

323 1492 **NNIKTHSIVLVTSK**GRFISRGPWTKVLEK**LGAEQGFSLKEKMAFVGFKGSFRPVWVKLVT**

F1P1G6_CHICK 1280 NNIKNHSIVLVTSKGRFISRGPWTKVLEKLGAEKGFSLKEKMAFVGFKGSFRPVWVKLVT

323 1552 **NEDSAKIYQALPIPVVKK**MKL

F1P1G6_CHICK 1340 NEDSAKIYQALPIPVVKKMKL

**M is the first residue of the new entry 323b**

**3617**

3617 1 VCLVTFIVSRIVDYIIDLEISLYLLSLLIPTYPALAVMLDSEQPYIHSVVFVFLLRYLEI

E1BU21_CHICK 1 ---------------------------------------------------------AGA

3617 61 KYERPVLRKDPVFRISPIKENSHQHPEEPEEEDEDVKAEREAVKNAIAAPSPEEKSVIIV

E1BU21_CHICK 4 EHRDTPSSQAPLSQGTPVLPEPPRSPPSPRPG-----------LSALPQAGP-QRPLLGA

3617 121 SNLYKEYKIKKAGSIFRKKKKMATKNISFCVKKGEVLGLLGPNGAGKSTVIKMIAGETTL

E1BU21_CHICK 52 REALRYYRRKAAR--WNRRHKLYRQELNL-------------------------------

3617 181 TAGQLCFAVCMLGNPSVLLLDEPSTGMDPNGQRCIWFIGSIQYLKNKFGKGYLLEIKVKD

E1BU21_CHICK 79 ---------------------TASAALLPLRPEASWL---------QFHLGISRD-----

3617 241 PESTDLLHAEILRIFPSAARQERFPSLLVYKVPMEDALPLSQSFSKLE----------EV

E1BU21_CHICK 104 ------------GLYPRSS-----PAVSRLLRDMHDFATISADYSQDEKALLGACDCSQI

3617 291 VKPSGVHLKLVLRFQDFGKAMFKPMRQKREEETPEDFFYFVDFQR**HNAEIAAFHLDR**ILD

E1BU21_CHICK 147 VKPSGVHLKLVLRFQDFGKAMFKPMRQKREEETPEDFFYFVDFQRHNAEIAAFHLDRILD

3617 351 FRRVPPTVGRLINITKEILEVTRNEVLQSVFFVSPASNVCFFAKCPYMCKTEYAVCGNPH

E1BU21_CHICK 207 FRRVPPTVGRLINITKEILEVTRNEVLQSVFFVSPASNVCFFAKCPYMCKTEYAVCGNPH

3617 411 LLEGSLSAFLPSLNLAPRLSIPNPWIRSYSFDEKEEWEVNPLYCDTVREIYPYSSGNRLL

E1BU21_CHICK 267 LLEGSLSAFLPSLNLAPRLSIPNPWIRSYSFDGKEEWEVNPLYCDTVREIYPYSSGNRLL

3617 471 NIVDMAIFDFLIGNMDRHHYEMFTK**FGDDGFLLHLDNAR**G--------------------

E1BU21_CHICK 327 NIVDMAIFDFLIGNMDRHHYEMFTKFGDDGFLLHLDNARGFGRHSHDEISILAPLSQCCV

3617 511 IKRTTLLRLQLLAEPEYQLSAVMRESLLQDPLAPVLTEPHLLALDRRLQLILKAVRKCID

E1BU21_CHICK 387 IKRTTLLRLQLLAEPEYQLSAVMRESLLQDPLAPVLTEPHLLALDRRLQLILKAVRKCID

3617 571 THGEAKVVANDTRQPEAPASDRVKLST

E1BU21_CHICK 447 THGEAKVVANDTRQPEAPASDRVKLST

**V is the first residue of the new entry 3617b**

**3793**

3793 1 MAEPESTVINTDVKQKDPSEALVIAVTTRAIFNLEEEHQLYLEKGKEEYVRHQQANQDKP

CATD_CHICK ------------------------------------------------------------

3793 61 LPPGTAFAFIQAAQYVNKKILESNPTEKGLFDILVLSNNSPESGMRIINSVKHYGLEISK

CATD_CHICK ------------------------------------------------------------

3793 121 FCFVSDEDSTQYLKSHGVKLFLSADRTDVCNALRRGVSAALIFQQEVQATSTPLRVAFDG

CATD_CHICK ------------------------------------------------------------

3793 181 DAVLFSDETDQIFREQGLEGAMQYERAMEAVPIGEASPKVQRGLLHLIL-------AIAS

CATD_CHICK 1 --------------------------------------MAPRGLLVLLLLALVGPCAALI

3793 234 XIPLTKFTSTR**RMLTEVGSEIPDMNAITQFLKFKLGFADLAEPTPEILKNYMDAQYYGEI**

CATD_CHICK 23 RIPLTKFTSTRRMLTEVGSEIPDMNAITQFLKFKLGFADLAEPTPEILKNYMDAQYYGEI

3793 294 **GIGTPPQK**FTVVFDTGSSNLWVPSVHCHLLDIACLLHHKYDASKSSTYVENGTEFAIHYG

CATD_CHICK 83 GIGTPPQKFTVVFDTGSSNLWVPSVHCHLLDIACLLHHKYDASKSSTYVENGTEFAIHYG

3793 354 TGSLSGFLSQDTVT--------------------------FDGILGMAFPR**ISVDKVTPF**

CATD_CHICK 143 TGSLSGFLSQDTVTLGNLKIKNQIFGEAVKQPGITFIAAKFDGILGMAFPRISVDKVTPF

3793 388 **FDNVMQQK**LIEK**NIFSFYLNRDPTAQPGGELLLGGTDPK**YYSGDFSWVNVTRKAYWQVHM

CATD_CHICK 203 FDNVMQQKLIEKNIFSFYLNRDPTAQPGGELLLGGTDPKYYSGDFSWVNVTRKAYWQVHM

3793 448 DSVDVANGLTLCK**GGCEAIVDTGTSLITGPTK**EVK**ELQSAIGAKPLIKGQYVIPCEKISS**

CATD_CHICK 263 DSVDVANGLTLCKGGCEAIVDTGTSLITGPTKEVKELQTAIGAKPLIKGQYVISCDKISS

3793 508 **LPVVTLMLGGKPYQLTGEQYVFK**VSAQGETICLSGFSGLDVPPPGGPLWILGDVFIGPYY

CATD_CHICK 323 LPVVTLMLGGKPYQLTGEQYVFKVSAQGETICLSGFSGLDVPPPGGPLWILGDVFIGPYY

3793 568 TVFDR**DNDSVGFAK**SA

CATD_CHICK 383 TVFDRDNDSVGFAKCV

V is the first amino acid of the new entry 3793b

**4290**

4290 1 ------------KLARLSQK**YPKAAFSDVAKLVHDTK**EIHKECCEGDMVECMDDMAEIIN

F2Z4L6_CHICK 181 LKQFGDRVFQARQLIYLSQKYPKAPFSEVSKFVHDSIGVHKECCEGDMVECMDDMARMMS

4290 49 NMCSRQDAFSSK**IKGCCEKPVVERSQCIMEAEFDEKPADLPSLVEKYIQDKEVCK**SFEAG

F2Z4L6_CHICK 241 NLCSQQDVFSGKIKDCCEKPIVERSQCIMEAEFDEKPADLPSLVEKYIEDKEVCKSFEAG

4290 109 HDAFMSD-----------------------------------------------------

F2Z4L6_CHICK 301 HDAFMAEFVYEYSRRHPEFSIQLIMRIAKGYESLLEKCCKTDNPAECYANAQEQLNQHIK

4290 116 ------------------------ILVRYSK**KMPQVSTETLLEIGKKMTAIGTKCCQLPE**

F2Z4L6_CHICK 361 ETQDVVKTNCDLLHDHGEADFLKSILIRYTKKMPQVPTDLLLETGKKMTTIGTKCCQLPE

4290 152 **DRRMACSEGYLSIVIHDMCKRQETTPINDNVAHCCSDSYAYRRPCFTAMGVDTKYVPPPF**

F2Z4L6_CHICK 421 DRRMACSEGYLSIVIHDTCRKQETTPINDNVSQCCSSSYANRRPCFTAMGVDTKYVPPPF

4290 212 **NPDMFNFDEKLCTAPAEERELGQMKLLINLIKRKPQMTEEQIKTIADGFTSMVDKCCK**QS

F2Z4L6_CHICK 481 NPDMFSFDEKLCSAPAEEREVGQMKLLINLIKRKPQMTEEQIKTIADGFTAMVDKCCKQS

4290 272 DINTCFGEEIDRNVLNDLHSSYSAMIMFAQYVQGNTFGQVVKMAEAVTDFAKRCTDTDRD

F2Z4L6_CHICK 541 DINTCFGEE-GANLIVQSRATLGIGA----------------------------------

4290 332 NPNCQKPLAPKVVKPIREDGLRQEHTCGILKKFGERTIKALKLVQISQKFPKADFFTVTK

4290 392 LVSDVANMHKDCCRGDMLECMSDREEILHYVCTNQEIISSKIKKCCEKPLLQRSECIINA

4290 452 ENDDKPANLSPQVREFIEDKGICERFAQEKDNHLASSSYALRRPCMGKLEIDENYVPLSL

4290 512 TPDLFTFHEDLCTTEEEKLQHKKQE

**N is the first residue of the new entry 4290b**

**4076**

4076 1 MLGIKLLLELFRAVLIITACE---RQK**DTSDQNFDYMFKLLIIGNSSVGK**TSFLFRYADD

E1C8J9_CHICK 1 --------MDYRSKLMASVTDARYRQKDTSDQNFDYMFKLLIIGNSSVGKTSFLFRYADD

4076 58 TFTPAFVSTVGIDFKVKTVYRNDKRVKLQIWDTAGQERYR**TITTAYYR**GAMGFILMYDIT

E1C8J9_CHICK 53 TFTSAFVSTVGIDFKVKTVYRNDKRVKLQIWDTAGQERYRTITTAYYRGAMGFILMYDIT

4076 118 SEDSFNAVQDWATQIKTYSWDNAQVILVGNKCDMEDERIVPLEKGKHLADQLEDEEEEDE

E1C8J9_CHICK 113 SEDSFNAVQDWATQIKTYSWDNAQVILVGNKCDMEDERIIPLEKGKHLADQLGFDYFEAS

4076 178 SEGESDEDDSGAEIEDGREGFDEDCDEGDEDDDSEEVVFMGSSKYPDENGFDAFLKKHGG

E1C8J9_CHICK 173 AKENINVRQVFERL------VDIICEKMSESIESDPS--------RGTSGRNVRLTDNPP

4076 238 SDNASTDCERTVFQFDVQRKYFKEALDRWAQFFIHPLMIRDAIDREVEAVDSEYQLARPS

E1C8J9_CHICK 221 PSQQNCSC----------------------------------------------------

4076 298 DANRKEMLFGSLARPGHPMKKFFWGNADTLKHEPIKNNIDTYTRLRDFWQCHYSAHYMTL

4076 358 VVQSKETLDTLEKWVTEIFSEIPNKIWEEIQKIEANEFHYQEQVFTHCFLLACRDVRLLI

4076 418 LEHGRWSMIDKYQTLMKGLSIEALSSFVKAFKSQLFVEGLVQGNFTSREAKDFLNYVVQK

4076 478 LQFAPLAHPCPVQFRVVDLPNTHLLCKVKTLNRGDANSEVTVYYQSGARSLREYTLMELL

4076 538 VVSYRSCPKAHEMFLPADSA

E is the first amino acid of the new entry 4076b

**4336**

4336 ------------------------------------------------------------

O93601_CHICK 1 MSPKAALLLVLLAVAGTRADVSPDQVATVLWRYFTELGSSAKETVDQLQQAEITKQLNTL

4336 1 -------MNSYAKELQR**SLVPFATELQAQLVQDSQR**LKEQIQRELAELQAK**LAPYADEVH**

O93601_CHICK 61 LQSNLQSANSYAEELQRRLVPFATELQAQLVQDSQRLKKQIQQELAELQAKLAPYADEVH

4336 54 **QQIGTNIR**ELQAK**LSPYADELR**SQVDRGTGELR**KALEPFATELR**ER**LQDNAGSIQASLGS**

O93601_CHICK 121 QQIGTNIRELQAKLSPYADELRSQVDRGTGELRRALEPFATELREKLQDNADSIQASLGP

4336 114 **FSER**LQQK**IDSSVEGLKGQLTPLADDLK**EQ--------------------DGLNR**QLESL**

O93601_CHICK 181 YAERLQQQIDSSVEGLKGQLTPLADELKEQVAQSVEGLRKGLSPYAQEVQDGLNRQLQSL

4336 154 **TMQMER**VAEELRSRLAASSEEMRAQLSSQAQELQQALGGDAEA-----------------

O93601_CHICK 241 TAQMERAAEELRSRLAASSEEMRAQLSPLAQELQEALRGDAEAMQQRLAPLAQQLDERLA

4336 197 -TVEAFR**QQVAPTSETFGQQLVK**RLEEMK**QKLESGTAGVEDHLDLLEK**ESCSSLAQQIMS

O93601_CHICK 301 QTVEAFRQQAAPISETFRQQLVQRLEEMKQKLESGTAGVEDHLDLLEKEVREKVATFLST

4336 255 LKAALLLTLLATLPVSPAELARSGFWEYLSQLTSDKDSLEQAQGSKLGREFTNLKESLQD

O93601_CHICK 361 TEQAES------------------------------------------------------

4336 316 RGSNVGNFLEKLAPLNRGIQPRLYHDSDSLRKLIRKELDSLRVKLSPYVDDVHHRVKDIF

4336 376 QPYADRLLSEIQRSVEELHRNVVPHSPASPEQLNQHIGELSAKLTQNARDLHRNIQRNLE

4336 436 QLKAKLSLHPGEHHEEMASEVQQRIEEFRRETYRQIQDFTRAVHQETEDMRLKLSARPQY

4336 496 LEEAAGSPAPLEDLRASLDALWRDLAYSLSERGGEAA

S is the first residue of the new entry 4336b

**455**

455 1 ------------------------MKGRSCLTNLISSCGQVTHLVGNAEDVDHSVSCVMF

F1NEW8_CHICK 241 NVCALYTYGQPVQGSARINVCQRHFYNPQCQQSQKPRCEAVIGLLENNGCLSTVVSTKTF

455 37 R**V**--------**VTLDSQFR**PVQETVSIHMAN-----QCRGMPESMFASNN---------FI

F1NEW8_CHICK 301 QLYRSYARMYASFNIETIVTENGTGIQMKNYDYVAVSQENDRVMFRNMDQYYRRGIPYFG

455 75 IHDVSKARNQPVKLSLDWVVVLEVNGEYQANYTTDENGTAAFSLDTSNFFDPSVKLR**ATQ**

F1NEW8_CHICK 361 EITVTNADGKPVP---SRVVVLEVNGEYQANYTTDENGTAAFSLDTSNFFNPTVKLRATQ

455 135 **APDGCADPFMWRNDHESEALFYVHR**FYSRTNSFVR**IEPVEEKLSCGQQRTINIHYVLSK**K

F1NEW8_CHICK 418 APDDCADFFMWRNDHESEALFFVRRFYSRTNSFVRIEPVEEKLRCGQQRMINIHYVLSRK

455 195 GYKNATYTNFYYVVMAK**GKIVLSGQK**QVRITP------GKFSTVLTPLLQNIIPSTR---

F1NEW8_CHICK 478 GYRNATHTDFYYVVMAKGKIVLSGQKQVRIAHASTAPWGTFAITLD-VTEKLTPSARLLL

455 246 -------TLVAHIRW---------------------------------------------

F1NEW8_CHICK 537 YTVHPDGEIVADSSWIHSDVCFKNKLQLEFSEKQAYPGSKINIHLEAAANSYCALRAVDQ

455 254 -----DESRELWLNRVYYQLR**LNDLYGYYYNGLNLQDDQPVECTPVK**TTFFDGLYYEPVN

F1NEW8_CHICK 597 SVFLLQPERELSAESVYYRLHLSDLYGYYYNGLNLQDDPPEECTPVKTTFFDGLYYEPVN

455 309 VSHDGDVYR**IFREMGLK**VFTNSTLR**KPVLCNEDKLDEDENRIYFDHSASGGSAFGK**GFSK

F1NEW8_CHICK 657 VSHDGDVYRIFREMGLKVFTNSTLRKPVLCNEDKLDWEENRIYFEHGASGGSAFGKEFSK

455 369 **ITAAGVVNTVR**KYFPETWIWDLVHTDSTGEASIDYTIPDTITEWKASAFCLQDDVGFGIS

F1NEW8_CHICK 717 ITAAGVVNTVRKYFPETWIWDLVHTDSTGEANIFYTVPDTITEWKASAFCLQDDAGFGIS

455 429 SPVSVIGFLPFFVDLSLPYSVIR**GEKLNLIANIFNYLDK**CIQ------------------

F1NEW8_CHICK 777 SPVSLTAFQPFFVDLALPYSVIRGEKINLIANIFNYLDKCIQISAILAESSDYKAEVLSP

455 ------------------------------------------------------------

F1NEW8_CHICK 837 EGNTARVCANERKTYIWAVSPLSLGEVKFTITAEAKLNTKGAKNSTPPEEESIRTDTLTQ

455 ------------------------------------------------------------

F1NEW8_CHICK 897 TLLVEPEGIKKELTQSSLICTKGTTVSEPVLLSLPRNVVQGSARVYFSVIGDILGTALRN

455 ------------------------------------------------------------

F1NEW8_CHICK 957 MENLLHMPYGCGEQNMALFTPNIYVLDYLNKTGQLTEEIRVKSTGYLTTGYQKQLSYKHQ

455 ------------------------------------------------------------

F1NEW8_CHICK 1017 DGSYSSFGTRDKEGNVWLTAFVYKSFAQARRYIYVDENVQSQTLIWLARKQKSDGCFENA

455 471 ---------GGEEGEYTLTAYVVAALLEAGHSVQHPVVSSGMNCLETAFSNGVHNLYNHA

F1NEW8_CHICK 1077 ESHFNNALKGGEEGEYSLTAYIVAALLEAGHSVQHPVVWNGMNCLETAFSNGVHNLYNHA

455 522 LFAYVYGLADKQER**YQYFIEELDRRATRDDGSVYWQREKKPPAEHFPAFYSRAPSAEIET**

F1NEW8_CHICK 1137 LFAYVYGLADKQERYQYFLEKLDKRATRDGGSVYWQRENKPPAEHFPAFYSRAPSAEIEM

455 582 **TSYVLLALLNKAELTPDDLSYSSRIVYWLVKQQNPYGGFSSSQDTVVALQALAQYGYYTF**

F1NEW8_CHICK 1197 TSYVLLALLNKAELTPDDLSYISRIVYWLVKQQNPYGGFSSSQDTVVAIQALAQYGYLTF

455 642 **SK**ETR**NTVKVNFMEIPKKVFEVNDKNR**FLLQQTALPTVPGNYSVEAHGTGCVYMQTTLR**Y**

F1NEW8_CHICK 1257 SKESHNTVKVNFMEIPKKAFQVNDENRFLLQQTSLPTVLGNYSVEVYGTGCVYMQTTLKY

455 702 **NIHLPKK**AAGFFLSVEPANVSCTSNFPPK**FDLVFSASYTGNR**NVSNMAVIDVK**MLSGFIP**

F1NEW8_CHICK 1317 NIHLPKKAAGFFLSVEPANVSCTSNFPLKFDLVFSASYTGNRNVSNMAIIDVKMLSGFIS

F1NU63_CHICK 1 MHCFLGREILS

455 762 **DRSSLKELQYQASVVDHVDIKNDHVFFYLQK**LSQKEVSFSFSVEQSLPVSDIKPAPVHLY

F1NEW8_CHICK 1377 DRSSLKKLQYQASVVDHVDIKNDHVFFYLQKLSQKEVSFSFSVEQSLPVSDIKPAPVHIY

F1NU63_CHICK 12 FFCLTVRKMWLKFILAILLLHAAAGKEPEPQYVLMVPAVLQSDSPSQVCLQFFNLNQTIS

455 822 DYYETVRKMWLKFLLAILLLHATAGKEPEP------------DSPSQVCLQFFNLNQTIS

F1NEW8_CHICK 1437 DYYETDEYAFAEY---------KTPCSPPSN-----------------------------

F1NU63_CHICK 72 VRVVLEYDTINTTIFEKNTTTSNGLQCLNFMIPPVTSVSLAFISFTAKGTTFDLKERRSV

455 870 VRVVLEYGTVNTTIFEKNTTASNGLQCLNFM---------DMVGFQGCGGAL--------

F1NU63_CHICK 132 MIWNMESFVFVQTDKPIYKPGQSVMFRVVALDFNFKPVQEMYPLIAVQDPQNNRIFQWQN

455 913 ---------LAHVQLAIH-PYSEVLFNSVAL-----------------------------

F1NU63_CHICK 192 VTSEINIVQIEFPLTEEPILGNYKIIVTKKSGERTSHSFLVEEYVLPKFDVTVTAPGSLT

455 934 ------------------------------------HSY---------------APQLVL

F1NU63_CHICK 252 VMDSELTVKICAVYTYGQPVEGKVQLSVCRDFDSYGRCKKSPVCQSFTKDLDTDGCLSHI

455 943 IVRIAMT------------------------------------------QLDTDGCLFQV

F1NU63_CHICK 312 LSSKVFELNRIGYKRNLDVKAIVTEKGTGLQLTATQSISITQVMSSLQFENVDHHYRRGI

455 961 ISSKVFELNRVGYRRNLDVK**AIVTEKGTGLQRTATQSISITQVVSSLQFENMDHYYRRGI**

F1NU63_CHICK 372 PYFGQIKLVDKDNSPISNKVIQLFVNNKNTHNFTTDINGIAPFSIDTSKIFDPELSLKAL

455 1021 **PYFGQIKLVDKDNSPISSK**VIQLFVNNENTHNFTTDINGIAPFSIDTSK**MFDPELNLK**AI

F1NU63_CHICK 432 YKTSDQCHSEGWIEPSYPDASLSVQRLYSWTSSFVRIEPLWKDMSCGQKRMITVYYILNT

455 1081 YK**TGDQCHSEGWIEPSYPDAYFSVQRLYSWTSSFVR**IDPLWKYMSCGQKRMITVYYIMNM

F1NU63_CHICK 492 EGYEHINIVNFYYVGMAKGKIVLTGEIKVNIQADQNGTFMIPLVVNEKMAPALRLLVYML

455 1141 EGYEHINIVNFYYVGMAKGK**IVLTGEIK**VNIQADQNGTFTIPLVVNEKMAPALR**LLVYML**

F1NU63_CHICK 552 HPAKELVADSVRFSIEKCFKNKVQLQFSEKQMLTTSNVSLVIEAAANSFCAVRAVDKSML

455 1201 **HPAKELVADSVRFPIEK**CFKNK--------------------------------------

F1NU63_CHICK 612 LLKSETELSAETIYNLHPIQDLQGYIFNGLNLEDDPQDPCVSSDDIFHKGLYYRPLTSGL

455 1223 ------------IYNLHPIQDLQGYIFNGLNLEDDPQDPCVSSENIFHK**GLYYRPLTSGL**

F1NU63_CHICK 672 GPDVYQFLRDMGMKFFTNSKIRQPIVCTRETVRPPSYFLNAGFTASTHHVKLSAEVAREE

455 1272 **GPDVYQFLR**DMGMKFLTNSKVRQPVVCTSETVRPPSFFLNAGFTASTHHV----------

F1NU63_CHICK 732 RGKRHILETIREFFPETWIWDIILINSTGKSVSYTIPDTITEWKASAFCVEELAGFGMSV

455 ------------------------------------------------------------

F1NU63_CHICK 792 PATLTAFQPFFVDLTLPYSIIHGEDFLLRANVFNYLNHCIKINVLLLESLDYQAKLISPE

455 ------------------------------------------------------------

F1NU63_CHICK 852 DDGCVCAKIRKSYVWNIFPKGTGDVLFSITAETNDDEACEEEALRNIRIDYRDTQIRALL

455 1321 -------------------RSSGNVLFSITAETNDDEACEEEALR**NISTDYRDTQIR**TLL

F1NU63_CHICK 912 VEPEGIRREETQNFLICMKDDVISQDVAIDLPTNVVEGSPRPSFSVVGDIMGTAIQNVH//

455 1362 VEV---------LLTALP--------VCPVLPT-MRKGN

V is the first amino acid of the new entry 455b

**4960**

F1P1J6_CHICK 61 TSLFSEDFKLETSNQVIDYDTSHIYTGHIYGEQGSFSHGSVIDGRFEGFIQTHSGTFYVE

4960 1 -------------------------------EQGSFSHGSVIDGR**FEGFIQTHSGTFYIE**

F1P1J6_CHICK 121 PAERYIKDRTLPFHSVIYHEDDIKYPHKYGPQGGCADHSVFERMQKYQMTGIEEPTTEKP

4960 30 **PAER**YIKDR**TLPFHSVIYHEDDIKYPHKYGPQGGCADHSVFER**MQKYQMTGIEEPTTE--

F1P1J6_CHICK 181 SEESDSDGPQLLRKKRATQAEKNTCQLYIQTDHLFYKHYGTREAVIAQISSHVKAIDTIY

4960 88 ------------------------------------------------ISSHVKAIDTIY

F1P1J6_CHICK 241 RSTDFSGIRNISFMVKRIRINTTVDEKDPSNPFRFPNIGVEKFLELNSEQNHDDYCLAYV

4960 100 RSTDFSGIRNISFMVKRIRINTTVDEKDSSNPFR**FPNIGVEK**FLELNSEQNHDDYCLAYV

F1P1J6_CHICK 301 FTDRDFDDGVLGLAWVGAPSGSSGGICEKSKLYSDGKKKSLNTGIITVQNYGSHVPPKVS

4960 160 FTDRDFDDGVLGLAWVGAPSGSSGGICEKSKLYSDGKKKSLNTGIITVQNYGSHVPPKVS

F1P1J6_CHICK 361 HITFAHEVGHNFGSPHDSGMECTPGESKNLGQKENGNYIMYARATSGDKLNNNKFSLCSI

4960 220 HITFAHEVGHNFGSPHDSGMECTPGESKNLGQKENGNYIMYARATSGDKLNNNKFSLCSI

F1P1J6_CHICK 421 RNISQVLEKKRNNCFVESGQPICGNGLVEEGEQCDCGYSDQCKDECCYDANQSDDKKCKL

4960 280 RNISQVLEKKRNNCFV--------------------------------------------

F1P1J6_CHICK 481 KPGKSCSPSQGPCCTAQCNFKLKTDKCRNDSDCAREGMCNGYSALCPASQPKQNFTECNR

4960 ------------------------------------------------------------

F1P1J6_CHICK 541 RTQVCIKGQCTGSICEKYDLEECTCASSDGKDDRELCHVCCMRKMDPDTCASTGSSRWEK

4960 296 -----------------------------------------------------GSSRWEK

F1P1J6_CHICK 601 HFARKTITLQPGSPCNDFRGYCDVFMRCRLVDADGPLARLKKAIFNPELYENIAEWIVA-

4960 303 HFARKTITLQPGSPCNDFRGYCDVFMRCR**LVDADGPLAR**LKKAIFNPELYENIAEWIVHT

F1P1J6_CHICK 660 ----------HWWAVLLMGIALIMLMAGFI------------KICSVHTPSSNPKLPPHK

4960 363 ADSFDFFIRFSWTNAFKLLSAFKIPISTFLPQQTAFAFDNAKSISMVLWKWTSTCIKPAW

F1P1J6_CHICK 698 PLPGTLKRRRPPQTA---------------------------------------------

4960 423 NIDSEAPQRASPYVQLKDSDLEKGVLRLLCNISIQLAAGVAQTAVRGSHSFIPRGTFDEW

F1P1J6_CHICK ---

4960 483 SRH

H is the first residue of new entry 4960b

**5368**

5368 1 --------------------------------DVITQDVAIDLPTNVVEGSPRASFSVVG

OVOS_CHICK 901 IDYRDTQIRALLVEPEGIRREETQNFLICMKDDVISQDVAIDLPTNVVEGSPRPSFSVVG

5368 29 DIMGTAMQNLHQLLQMPFGSGEQNMVLFAPNIYVLDYLNKTR**QLSEDVKSK**TIGYL----

OVOS_CHICK 961 DIMGTAIQNVHQLLQMPFGNGEQNMVLFAPNIYVLDYLDKTRQLSEDVKSKTIGYLVSGY

5368 ------------------------------------------------------------

OVOS_CHICK 1021 QKQLSYKHPDGSYSTFGIRDKEGNTWLTAFVYKSFAEASRFIYIDDNVQAQTLIWLATKQ

5368 85 ------------------GGVDNELSLSAYVTIALLEAGHSMSYTVIR**NAFYCLETASEK**

OVOS_CHICK 1081 KTDGCFQSTGILVNNAMKGGVENELSLSAYITIALLEAGHSMSHTVIRNAFYCLETASEK

5368 127 NISDIYTQALVAYAFCLAGK**AEICESFLR**ELQKSAKDIGGSKYWEQKQRSPTENSHLLDH

OVOS_CHICK 1141 NITDIYTQALVAYAFCLAGKAEICESFLRELQKSAKEVDGSKYWEQNQRSAPEKSHLLDH

5368 187 VPSIEVEITSYVLLALLYKPNRNQEDLTK**ASAIVQWIIRQQNSYGGFSSTQDTVVALQAL**

OVOS_CHICK 1201 VQSTDVEITSYVLLALLYKPNRSQEDLTKASAIVQWIIRQQNSYGGFASMQDTVVALQAL

5368 247 **AAYGEATYNSVTQNVIK**IISKNTFAK**VFTVNNENRLLLQQTPLPEVPGK**YSLTVNGTGCV

OVOS_CHICK 1261 AAYGAATYNSVTQNVIKINSKNTFEKVFTVNNENRLLLQQTPLPQVPGKYSLTVNGTGCV

E1C544_CHICK 1 ------------------------------------------------------MGKDGS

5368 307 LIQDSFPGDPTQEQQFLEQLEFCFPEAQGPDSTFCQDCIPPEHKLHQGMIIAA----PAA

OVOS_CHICK 1321 LIQTAL-----RYNIHLPEGAFGFSLSVQTSNASCPRDQPGKFDI---VLISSYTGKRSS

E1C544_CHICK 7 SKLNIFILLFFLSGDASPVTEP-QYMVLLPFLIHTDSPEKICVQLTHL----NESVTLNA

5368 363 SNL-YFSNAFFLIG----EHHIQQYMVLLPFLIHTDSPEKVCVQLAHL----NESVTLSA

OVOS_CHICK 1374 SNMVIIDVK-MLSGFVPVKSSLDQLIDDHTVMQVEYKKNHVLLYLGNILQKRRKEVTFSV

E1C544_CHICK 64 AIEYQGENRSLIDDVVSEKDLFTCIPFTIPKSRSQSTVTFITVTVKGETLQFRNRKSVL

5368 414 TLEYQGENR**SLIDDVVTEK**DMFACIPHSVSNLILLGVSFVFIAKMR

OVOS_CHICK 1433 EQDFVVTHPK--PAPVQIYDYYETEEYAVAEYMSLCRGVV--EEMG

**L is the first amino acid of the new entry 5368b**

**6069**

6069 1 MHPVLKAFVCGSISGTCSTLLFQPLDLLKTRLQTLQPAVGGLFCIIVGSLFCVADQLDPV

6069 61 LMPVVNFGCGIFAGILASLATQPADVIKTHMQLPPDKYRRTSQAIAFIYKGFVLETYVSL

RSSA_CHICK 1 -----------MSGGLDVLQMKEEDVL---------KFLAAGTHLGGTNLDFQMEQYI--

6069 121 KASKFYEQKWTCTETVLITLNILSPVGIYIINLKRTWEKLLLAAR**AIVAIENPADVSVIS**

RSSA_CHICK 39 -----YKRK---------------SDGIYIINLKRTWEKLLLAARAIVAIENPADVSVIS

6069 181 **SR**NTGQRAVLK**FAAATGATPIAGRFTPGTFTNQIQAAFR**EPRLLVVTDPRADHQPLTEAS

RSSA_CHICK 79 SRNTGQRAVLKFAAATGATPIAGRFTPGTFTNQIQAAFREPRLLVVTDPRADHQPLTEAS

6069 241 YVNIPTIALCNTDSPLRYVDIAIPCNNKGAHSVGLMWWMLAREVLRMRGTISREHPWEVM

RSSA_CHICK 139 YVNIPTIALCNTDSPLRYVDIAIPCNNKGAHSVGLMWWMLAREVLRMRGTISREHPWEVM

6069 301 PDLYFYRDPEEGSALGWVLFNTIINDTDREFECTLSKAADLIKLSGAIDTIEERDAIQGD

RSSA_CHICK 199 PDLYFYRDPEE-----------IEKEEQAAAEKAVTKEEFQTEWTAPAPEFTAPPQ----

6069 361 LDRLQKWTHENLMRQGVRIVAVLSLFQGMVPLGVLLARAVLAEGMVPKCLASVAVSPVRV

RSSA_CHICK 244 -PEVADWS------EGVQVPS--------VPIQQFPTEDWSAQPATED-WSAAPTAQATE

6069 421 VVG------

RSSA_CHICK 288 WVGTTTEWS

**G_147_ is the first amino acid of the new entry 6069b; G_312_ is the first amino acid of the new entry 6069c**

**6544**

6544 1 MGIKVVRAQCKELSQSLFKSTCLIIRLLNPFADLPLFAIFAFATCGGYSGGLRLSVDCAN

6544 61 KSESDLNIDIAFAYPFRLHQVNFDAPTCEGKRRETLSLIGDFSSSAEFFVTIAVFAFLYS

6544 121 LAATVVYIFFQNKYRENNRGPLIDFIVTVVFSFLWLVSSSAWAKGLSDVKIATDPDEVLL

6544 181 LMSACKQQSNKCLPVRSPVMSSLNTSVVFGFLNFILWAGNIWFVFKETGWHSSGQRHAAD

6544 241 TMEKQSSGYNQSGYNQDSYG--PSGGYNQPGSYGQISRVTPEKGLFLIQDQGWNIPISKQ

SNTAN_CHICK 1 -----MCGCRASVPSTKHYSVNPAPTTRSPPAAAGMPK---------------RIPIAKQ

6544 299 LASIKALEKGSDLEK**AFATAALVYNNSADPEGK**LSKAETK**NLLQTQFSRFIQGQEDKPKY**

SNTAN_CHICK 41 LASIKALEKGSDLEKAFATAALVYNNSADPEGKLSKAETKSLLQTQFSRFIQGQENKPKY

6544 359 **QEIISALDEEPEKK**IDFEDFMVSLVSLALLSDLLQEIRNVKCTK

SNTAN_CHICK 101 QEMISALDEEPEKKIDFEDFMISLVSLALLSDLLQEIKNVKSTK

**G is the first residue of the new entry 6544b**

**6907**

6907 1 --------MLLKSGLLLLLVISASASYEAEQNDSVSPRKPR**VAAQNSAEVVR**CLNSALQV

F1NU46_CHICK 1 HLAETCQRMLLKSGLLLLLVISASASYEAEQNDSVSPRKPRVAAQNSAEVVRCLNSALQV

6907 53 GCGAFACLENSTCDTDGMYDICKSFLYSAAKFDTQGKAFVKESLKCIANGVTSKVFLAIR

F1NU46_CHICK 61 GCGAFACLENSTCDTDGMYDICKSFLYSAAKFDTQGKAFVKESLKCIANGVTSKVFLAIR

6907 113 RCSTFQR**MISEVQEECYSKLDMCGIAK**R**NPEAITEVVQLPNQFSNR**YYNKLVR**SLLECDE**

F1NU46_CHICK 121 RCSTFQRMISEVQEECYSKLDMCGIAKRNPEAITEVVQLPNQFSNRYYNKLVRSLLECDE

6907 173 ETVSTIKDSLMEK**IGPNMASLFHLLQTDHCAQGHPR**TDFTRRRITEPQKLKLYFRNLRGQ

F1NU46_CHICK 181 ETVSTIKDSLMEKIGPNMASLFHLLQTDHCAQGHPRTDFARRRITEPQKLKLYFRNLRGE

6907 233 CLLSQPLQSDQQQSDPCHHPKQPQRRKPRVLFSQTQVLELERRFKQQKYLSALEREHLAK

F1NU46_CHICK 241 GSI--PAHAKRTSAESA-------------------------------------------

6907 293 VLQLTSTQVKIWFQNRRYKCKRQRQDRSLEMATYPLPPRRVAVPVLVRDGKPCFGGSQPH

6907 353 LVPYGLTVSPYSYSTYYGAYGVNYGVGYTGVLTP

**Q is the first residue of the new entry 6907b**

**713**

SIAL_CHICK 1 MRSALLLACLLATASAFSVRSWLRRARAGDSEENAVLKSRHRYYLYRYAYPPLHRYKGSD

713 1 MRTALLLACLLATASAFSVRSWLRRAR**AGDSEENAVLK**SRHRYYLYR**YAYPPLHR**YKG--

SIAL_CHICK 61 SSEEEGDGSEEEEEGGAPSHAGTQAAGEGLTLGDVGPGGDAASAHQDCKGGQKGTRGDSG

713 59 -----------------------------------------------CKGGQKGTQGDSG

SIAL_CHICK 121 DEDSDEEEEEEEEEEEEEEVEEQDVSVNGTSTNTTAETPHGNNTVAAEEEEDDDEEEEEE

713 72 DEDSDEEEEEEEEEEEEEEVEEQDVSVNGTSTNTTAETPRGNSTAAAEEEEEEEEEEGEE

SIAL_CHICK 181 EEEEEEAEATTAAATTAQDEVTTLGDEQRSEVTTAGEQWEYEVTVGARGDEGPTESSYGD

713 132 EEEEEEAEATTAAATTAQDEATTLGDDQRFEATTAGEQWEYEVTAGAR**GDEGPTEGSYGD**

SIAL_CHICK 241 QEEPARGDSYRAYEDEYGYYKGHGYDMYGQDYYYNQ------------------------

713 192 **QEGPPR**GDTYRNYEDEYGYYKGHGYDIYGQDYYYSHSFSINYPFNYLSNTPSNTLLANTS

OC116_CHICK 1 ---------------------------------------------------MRATLFCLC

713 252 VNILGYPIIYPTDYFIDHPIEYSSNTDTFLPAGATSSQGKRKLPWTAPGCNTNHGFFVFK

OC116_CHICK 10 LCLLG--TVLPT------PVSLPARARGNCP-------GQHQILLK--GCNTKHGFYIFQ

713 312 FMHSYLMRRNQTQVK**KEEGDHQGTIHGR**WLGKVDGEALGQGVGSSHVPEDK**DSPKPHSHI**

OC116_CHICK 53 YIYSHLMQKNQTQVKKEEGDHQGTIHGHWLGKVDGEAPGQGAGSSHVPEDKDSPKTHSHI

713 372 **TPVSKVGGR**ALRPSTGDSISVYPTSTSMEGSGDMGSMLLGEIINGEDGLPQSTRPGEPHG

OC116_CHICK 113 TPASKGEGRALRPGIGDSNSVYPTSTSVEGSGDMGSILLGEIINGEDGLPQSTHPGGPHG

713 432 DGNGGNGVLVDGAVTAGRERATNSEGAGGEGSSHATVPDQGQAGTMVTGDSAITSVTDSA

OC116_CHICK 173 DGDGGNGVLVDGAVTAGRERASGSEGAGSEGGSHAPVPDQGQAGTMGTGDSAITSVTDSA

713 492 ITSVTK**KEDVHVDAEDIGEFAYIPYVDAATITRGQDGETHVSPKDEVQIFIGRANIQVGE**

OC116_CHICK 233 ITSVTKKEDVHVDTEGIDEFAYIPDVDAVTITRGQDGETHISPEDEVKIFIGRANIQVGE

713 552 **NDGSAGSTGATSEANVIPAAVNAR**PQGYPEESASMATVHHGDSATSRPGDHPSAGNSGDG

OC116_CHICK 293 NDSSVGSAGATSEANVIPTVVTVRPQGHPEESATMATLHHGDSVTSRPVGHPSVGNSGDG

713 612 ATAIHSGQELEAPSPR**ESTDGEATVTTAAGSQSGK**GR**SGQRPVGKHSLPATMTTQGAR**GT

OC116_CHICK 353 ATEIHSGQELEAPSPWESTGGDATVTMAVGVQSGKGRSGQRALGKHSLPATMTTRGGRGT

713 672 ASSGLTMGDCSTAASTPSWK**GSQVVTAGQEGSREVGTAGPKR**QRARIQQEVAPAPDVEGG

OC116_CHICK 413 ASSGLTTGDCSTAASTPSRKGSHVVSAGQGESGEVGTAGPERQRARVQQEVAPARGVVGG

713 732 MVVPEGHR**ARIQQEVAPAPDVEGGMVVPEGHGAR**TQPGVASAPSTVETEAPERHR**SRAQQ**

OC116_CHICK 473 MVVPEGHRARVQQEVAPARGVVGGMVVPEGHRARTQPEVASAPSTVGKAAPERHRNRAQQ

713 792 **GVAPVHSMERETVTPERQRARMQPGSAR**MQQAAR**PEVAPAPSTAGGIVAPEGHRARVWPG**

OC116_CHICK 533 EVAPVPSMVVETVAPERHRARVRPESARLGQAARPEVAPAPSTGGRIVAPGGHRARVWPG

F1NSM8_CHICK 1 ------------------------------------------------------MKLAFL

713 852 **AAPAPGVIRVARPAPSRVYNGDKRDAMGKSTDVPRDPWVWGSTSPQTQHTRGSMVAGSFS**

OC116_CHICK 593 AAPAPGVVGVARPAPSKAYNGDKRVAIGKSTDVPRDPWVWGSAHPQAQHTRGSTVAGGFA

F1NSM8_CHICK 7 CL-------CFISIAAAWPVSKSRQ-HAISASSEEKY---------DPRSHHTHRYHQDH

713 912 **HLHGGQRMGSLAELERSRQVEQVRQADRLR**LHERAVYGLSGVGGPLQPLAAHTDPWSADS

OC116_CHICK 653 HLHRGQRLGGLTEMEHSRQVEQVRHADRLRLHERAVYGLSGVGGPLQPPAVHTDPWSADS

F1NSM8_CHICK 50 VDSQSQEHLQQTQNDLASLQQTHYSSEENADVPEQPDFPDIPSKSQEAVDDDDDDDNDSN

713 972 SQS-SEGRWGSRSNSGEEDGETHYSSEENADVPEQPDFPDVPSKSQETVDDDDDDDNDSN

OC116_CHICK 713 SQS-SEGRWGSHSDSHEEDGEVRGYPYGRQSL----------------------------

F1NSM8_CHICK 110 DTDESDEVVTDFPTEAPVTPFNRGDNAGRGDSVAYGFRAKAHVVKASKLRKAARKLIEDD

713 1031 DTDESDEVFTDFPTEAPVTPFNR**GDNAGRGDSVAYGFR**AKAHVVKASKIRKAARKLIEDD

F1NSM8_CHICK 170 ATAEVGDSQLAGLWLPKESREQDSRELAQHQSVENDSRPRFDSPEVDGGDSKASAGVDSR

713 1091 ATTEVGDSQPAGLWWPKESR**EQNSRELPQHQSVENDSRPK**FDSPEVDGGDSKASAGVDSR

F1NSM8_CHICK 230 ESLASASAVDTSNQTLESAEDAEDRHSIENNEVTR

713 1151 ESLASAPAVDTSNQTLESAEDAEDRHSIENNEVTR

**S is the first amino acid of the new entry 713b; T is the first amino acid of the new entry 713c**

**7743**

7743 1 MPCFPALVSQVEALKEENDSLRWQLDAYRNEVELLKQEHGKASRDEDTTKEQQLKLLQQA

7743 61 LQGMQQAKYQEFRISNCAERAVNCLTFLFQHLLKVQDEYKKKEAELEKVKEDKLKIETLL

F1NVQ6_CHICK 1 ----------------------------------------------------MSNMEKHL

7743 121 ETLKEQESSMPRVCACSQEKECPVEKTLSNSPVKSEREALLVGIISTFLHVHPFGASIEY

F1NVQ6_CHICK 9 FNLKFAAKELNR-----NSKRCDKEEKAEKAKIK--------------------------

7743 181 ICSYLQRLDSKAIQKGNMEVARIHAENAIR**QKNQAINFLR**MSARVDAVAARVQTAVTMGK

F1NVQ6_CHICK 38 ----------KAIQKGNMEVARIHAENAIRQKNQAINFLRMSARVDAVAARVQTAVTMGK

7743 241 VTKSMAGVVKSMDATLKSMNLEKISALMDKFEHQFETLDVQTQQMEDTMSNTTTLTTPQN

F1NVQ6_CHICK 88 VTKSMAGVVKSMDATLKSMNLEKISALMDKFEHQFETLDVQTQQMEDTMSNTTTLTTPQN

7743 301 QVDMLLQEMADEAGHLCLTFVLVLYFSVIYSLDLNMELPQGQTGSVGTSVASAEQ-----

F1NVQ6_CHICK 148 QVDMLLQEMADEAG-----------------LDLNMELPQGQTGSVGTSVASAEQDELSQ

7743 ---------

F1NVQ6_CHICK 191 RLARLRDQV

**K is the first amino acid of new entry 7743b**

**8164**

8164 1 MGTMRGLWALILVGLIGACDGMEMTAEEGAPCPQLWDEDLVGNKYENVTGFNLIKRFDLL

8164 61 KISSVKKVRGSRGAVLLRLGAVPLVQPTRDLQGSKVPWVPLGHQGPASLGHQVTVANLVL

8164 121 LGTLAVLGQSDKRKQEVRGRKQEVRGGRGDMAAFPGQ------AGPYSGAHYGGGAPHGV

H9KZ63_CHICK 1 MAGFPGQGYPGA------GQAGPYSGGPYGGGAAPGQPYGAPPAGPYGGAAPGGGAPQGV

8164 175 DPEALSWFQAVDTDRSGSISVKELKQALLNNNWSAFNDETCLLMINTFDSSRSGR**MDVYG**

H9KZ63_CHICK 55 DPEAFSWFQAVDADRSGYISVKELKQALLNSNWSAFNDETCLLMINMFDRTRSGRIDVYG

8164 235 **FSALLR**FIQQWR**SLFQQYDR**DQSGSISCTELQQAFSQMGYNLSPQFSQLLLSRYAQR**SSN**

H9KZ63_CHICK 115 FAALLRFIQQWKNLFQQYDRDQSGSISFSELQQAFSQMGYNLSPQFSQLLLSRYAQRSSN

8164 295 **PSIQLDRFIHICMQLQSLTDAFR**EKDTGMVGNVR**LGYEDFLTMVITR**ML

H9KZ63_CHICK 175 PSIQLDRFIHICMQLQSLTDAFREKDTGMVGNVRLGYEDFLTMVMTRML

**M is the first residue of the new entry 8164b**

**8367**

MIF_CHICK 1 MPMFTIHTNVCKDAVPDSLLGELTQQLAKATGKPAQYIAVHIVPDQMMSFGGSTDPCALC

8367 1 M**PMFTIHTNVCKDAVPDSLLGELTQQLAK**ATGKPAQYIAVHIVPDQMMSFGGSTDPCALC

MIF_CHICK 61 SLYSIGKIGGQQNKTYTKLLCDMIAKHLHVSADRVYINYFDINAANVGWNGSTFA-----

8367 61 SLYSIGKIGGQQNKTYTK**LLCDMIAK**HLHVSADR-YIGE-EEDADDTAQEERARALLERL

8367 121 REQAKARQLRRQQELGGEGLEDEAEQGRLRKEREGGSAVKRRKDGELGHGAVQAEVIPAV

8367 181 LQSAANGYLLGQGGYRPRDICVSAPTGSGKTLSFVIPIVQIIDEADRMIDDMHQNWLNQV

8367 241 VKAAFQAENEAGSNRLFQRTKPGPVTAARSSIMGLELYLDLLSQPCR**AVYIFAR**CNNIPF

E1BUB6_CHICK 1 ------------PTRRLRDRTDG--TGARSGSMGLELYLDLLSQPCRAVYIFARCNSIPF

8367 301 EFKRVELMKGQQRTEEFRKVNVLMKVPALKDGSFTLAE----------------------

E1BUB6_CHICK 47 EFKRVELMKGEHRTEEFRKVNVLMKVPALRDGSFTLAESVAILMYLTRKFKTPDHWYPSD/

**A is the first amino acid of the new entry 8367b; M is the first amino acid of the new entry 8367c**

**8527**

F1NRM4_CHICK 1 --------------------------------------------EAEVAAGSDMGCPGCA

8527 1 ---MITTFNGVEFNYSMPANCYHILVQDCSPELKFLVMMKR**LEESADLTAISVR**LASHKV

E1BYN5_CHICK 1501 SQNMITTFNGVEFNYSMPANCYHILAQDCSPELKFLVMMKRLGESADLTAISVRLASHEV

F1NRM4_CHICK 17 GLVLLLGLLQLLGRTAACPCQDPRLCQP-------IAGTD------RFE------VFVFD

8527 61 DMYVSNGLIQLKINGVQTPTDVPYTSKS-------DSTLELEEEQVRLDQKSEDLVFVFD

E1BYN5_CHICK 1561 DMYVSNGLIQLKINGVQTPTDVPYTSKSGLLISSEKEGLSLKAPEYGVE------KLYYD

F1NRM4_CHICK 58 VGKEAWKSYDWSKITTVAAFGKYDPELLCFAHSKGARVVLKGDVPLKEVVDPAKRAAWIS

8527 111 VGKEAWKSYDWSK**ITTVAAFGKYDPDLVCFAHSK**GAR**VVLKGDVPLQEVVDPANRAAWIS**

E1BYN5_CHICK 1615 RRKLEIRVAFWMVGKTCGICGKYDAE-------KKREYQMPSGYLAKDAVSFAQ--SWVI

F1NRM4_CHICK 118 **QQVDLAK**KQYMDGINIDIEQEVNETSPEYYALTELVKETTDAFHKEIPGSQVTFDVAWSP

8527 171 QQVDLAKKQYMDGINIDIEQEVNETSPVYYALTELVKETADAFHKEIPGS----------

F1NRM4_CHICK 178 ACIDKRCYNYTGIAAACDFLFVMSYDEQSQIWTDCIAKANAPYLQTLFELGYEEYITMGI

8527 221 ---------------------------QSQIWNDCIAK**ANAPYLQTL--VGYEDYIGMGI**

F1NRM4_CHICK 238 DPKKLVMGVPWYGYDYVCLNLSEDHVCSLPKVPFRGAPCSDAAGHQVPYGEIMKQVNSSI

8527 252 **DPK**KLVMGVPWYGYDYVCLNLTEDHVCSLPKVPFRGAPCSDAAGRQVPYGAIMKQVNSSI

F1NRM4_CHICK 298 SGALWDEVQNSPFYEYKDSLGHFHQVWYDDPRSISLKAAYVKNRGLKGIGMWNGNS/

8527 312 SGALWDEVQSSPFYEYKCSKG------------------------------------

**S is the first amino acid of the new entry 8527b**

**8715**

8715 1 -----------------------------------------------MPRISLQAQCQSS

E1BZV4_CHICK 1 RMMAARCALLLLCCMVLLPVVGARYLLSCPKGWSYYKQSCFRYFRQHRTWEEAEAQCQNS

8715 14 YSGAHLVWVEDPK**EAATLSRVIMYYQRTQPVWLGLHYLR**QYVEQSELERFDGVEAGKYTR

E1BZV4_CHICK 61 YSGAHLAWVEEPKEAATLSRVIMYYQRTQPVWLGLHYLRQSRDW------------RWTH

8715 74 GLGQQQMGFCAAHEDINSLCLTVVQRLVERGQLSWDS---------IGRLEVGTETVIDK

E1BZV4_CHICK 109 GVKFDDLRTLPGNGAQGGSCALLTR---SSSFTVWSSADCDRQHHFICKFTPSQ------

8715 125 SKAVKTVLMELFRESGNTDVEGIDTTNACYGGTASLFNAASWVESSAWDGRYAVVVCGDI

8715 185 AVYATGNARPTGGAGAIAMLVGPNAPLVLERGLRGTHMEHAYDFYKPDLSSEYPVVDGPL

8715 245 SIQCYLRALDRCYAVYRRKAESQWHQAGMKRPFTLDDFKFIIFHTPFCKLVQKSVGRLLL

8715 305 NDFLAAPNPDTTAGLYKGLQPF

**F is the first amino acid of the new entry 8715b**

**875**

875 1 MDCVTCLATSTTQRQNGFR**YLLYDVNPPEGFNLR**RDVYIRIASLMKTLLK**SENWVLVLPP**

Q7T1N6_CHICK 1 ------------------------VNPPEGFNLRRDVYIRIASLMKTLLKSENWVLVLPP

875 61 **WGRLYHWQSPDILQVRIPWSEFFDLPSLNR**NIPVIEYEQFLAESGGPFIDQIYVLQGYEE

Q7T1N6_CHICK 37 WGRLYHWQSPDILQVRIPWSEFFDLPSLNKNIPVIEYEQFLAESGGPFIEQIYVLQGYEE

875 121 GWKEGTWEEK**IDERPCIDQLMYSK**DKHQYYR-------------TGC-------------

Q7T1N6_CHICK 97 GWKEGTWEEKIDERPCIDQLMYSKDKHQYYRGWFWGYEETRGLNVSCLSVQGSASVVAPI

875 155 ------SEKLRLEMFKAWLDGAWSNQ----TRRSMVFAKHLRVAGDEFRNK**YLQSTDEAD**

Q7T1N6_CHICK 157 LLKNTSAQSVMLDRAENLLHDHYGGKDYWNTRRSMVFAKHLRVAGDEFRNKYLQSTDEAD

875 205 **RTHYNEDWTQMK**VK**MGTALGGPYLGVHLR**RK**DFIWGHR**EDVPSLHGAAKKIHSLLKTHKL

Q7T1N6_CHICK 217 RTHYNEDWTQMKVKTGTALGGPYLGVHLRRKDFIWGHREDVPSLHGAAKKIHSLLKTHKL

875 265 EK**VFIATDAVEDEIALLKKLVPEMVRFEPTWEELELYKDGGLAVIDQWICAHARYFIGTS**

Q7T1N6_CHICK 277 EKVFIATDAVEDEIELLKKLVPEMVRFEPTWEELELYKDGGMAVIDQWICAHARYFIGTS

875 325 **VSTFSFR**IHEEREILGFDPKTTYNRFCGEKERNWAAEVRLVNGNMRCAGRVEVKYNNEWG

Q7T1N6_CHICK 337 VSTFSFRIHEEREILGFDPKTTYNRFCGEKEKN-------------C------EQPTHWK

875 385 TVCGFFWDMKDAAVVCKELNCGSAIEALHYAHFGAGSGPIWLHDLKCNGSEAALSNCTHM

Q7T1N6_CHICK 378 IVY---------------------------------------------------------

875 445 GGYQRYCIHSVDAGVICSGLVRLVGDSDCSGHVEIHDGKQWKSVCDSHFGPKAADVVCRD

875 505 LLCGKALLLPRETPLDEEVSPICDRDLQEVMNPSVLISCLKKSSASQACTHPNGTHVTCT

875 565 RSRRVRLVDGAGRCAGRVEIYYQGKWGTVCDDAWDLADADVVCRQLSCGRAVEVAGSARF

875 625 GEGSGQIWLDGVNCSGTEAALWDCHAQAWGQHDCGHKEDAGVVCSEFMALRLENSDGCSG

875 685 RLQVFYNGTWGSVCSNSMTVDTVPLVCKQLGCGDSGTLEMSSRYDKLSGIAWLDRIECGK

875 745 SSRSFWQCPSAPWDPQSCDDLRDVTNITCSGRQPERPLVSVCPNSTTCTGKNSFSTWLLA

875 805 WQGCPDCAGATREDSASADREKIRAVGGKDRCSGRVEVWHHGSWGTVCDDSWDMQDAEVV

875 865 CRQLGCGPAVSAPGEAAFGEGTGFIWLEQVECRGTELSLQDCWARPGDSGVCRHKEDVGV

875 925 QCLGSRRTEEPFPEVVYEEIGYSSAWEKQTTFDHSDIAIVPRDDPEEGYDDAKEVSEPGN

875 985 DPDSGQGHSEVPRTQKEEEEPMDAVRGMSLHSQRNTGGSGAGGDTLSLFLRSTGYDDVEE

875 1045 VSLAHPHGDMSTVKLEWDTKGSLSPEQGEPISAMSPSAVAREEKLVLLREL

**C is the first amino acid of new entry 875b**

**887**

887 1 MISIQKGQKEEQASKSAQHTETCSNLESGICKNCLIDRTSRNRCQHCRLQKCLAVGMSRD

887 61 AVKFGRMSKKQRDSLYAEVQKHRMQQQQRDHQQQPGEAEPLTPTYNITTNGLTELHDDLS

887 121 NYIDGHTPEGSKADSAVSSFYLDIQPSPDQSGLDINGIKPEPICDYTPASGFFPYCSFTN

887 181 GETSPTVSMAELEHLAQNISKSHMETCQYLREELQQITWQTFLQEEIENYQNKQREVMWQ

887 241 LCAVKITEAIQYVVEFAKRIDGFMELCQNDQIVLLKAGSLEVVFIRMCRAFDSQNNTVYF

887 301 DGKYASPEVFKSLGCEDFISFVFEFGKSLCSMHLTEDEIALFSAFVLMSADRSWLQEKVK

887 361 IEKLQQKIQLALQHVLQKNHREDGILTKLICKVSTLRALCGRHTEKLIAFKAIYPDIVRL

887 421 HFPPLYKELFTSEFEPAMQIDGLAKKGVETSANTSENKQTGNIQATNAVEVEAKATINDD

887 481 PFPEPRLPYPFTSCLTEKEQKTYLYLMTKFSKTPNNFPLSAASQRELLTYLKMKEIVNNE

887 541 VAEFMKFAQNAAKSCTQDYDSISEDALLYTEELFRACIGHVEKYPEFYTLHEIMSIMGGK

887 601 FNTQLTFKLEKNLLVMDISSDSNAEKLALKYSPQVVLSNQSLFTLLNNHGLNYKEQWEIP

887 661 VCIKMIPTAGKLPDELQMIVEDPGEYKAPQDSNLVYKLFSLDDLLLLVRCNVQKLIPIFL

887 721 LPKLEYQAYYGVEALTESEVCQLWTESLLHSECIFYIDPVKMSTVHEILSK**LSLEGDHSL**

ANXA2_CHICK 1 -----------------------------------------MSTVHEILSKLSLEGDHSL

887 781 **PPSAYATVKAYSNFDADRDAAALETAIKTKGVDEVTIINILTNR**SNEQR**QDIAFAYQR**RT

ANXA2_CHICK 20 PPSAYATVKAYSNFDADRDAAALEAAIKTKGVDEVTIINILTNRSNEQRQDIAFAYQRRT

887 841 KKELSAALK**SALSGHLEAVILGLLKTPSQYDASELK**AAMK**GLGTDEDTLIEIICSRTNQE**

ANXA2_CHICK 80 KKELSAALKSALSGHLEAVILGLLKTPSQYDASELKAAMKGLGTDEDTLIEIICSRTNQE

887 901 **LYEINR**VYREMYK**TELEKDIISDTSGDFRKLMVALAK**GK**RCEDTSVIDYELIDQDAR**ELY

ANXA2_CHICK 140 LNEINRVYREMYKTELEKDIISDTSGDFRKLMVALAKGKRCEDTSVIDYELIDQDARELY

887 961 DAGVKRKGTDVPKWINIMTERSVPHLQKVFERYK**SYSPYDMLESIKKEVKGDLENAFVNL**

ANXA2_CHICK 200 DAGVKRKGTDVPKWINIMTERSVPHLQKVFERYKSYSPYDMLESIKKEVKGDLENAFLNL

887 1021 **VQCIQNKQLYFADR**LYDSMKGKGTRDKVLIRIMVSRCEVDMLKIKSEFKRKYGKSLYYFI

ANXA2_CHICK 260 VQCIQNKQLYFADRLYDSMKGKGTRDKVLIRIMVSRCEVDMLKIKSEFKRKYGKSLYYFI

887 1081 Q-----------VQFFSADDC

ANXA2_CHICK 320 QQDTKGDYQRALLNLCGGED-

**M is the first residue of the new entry 887b**

**979**

979 1 MAGGSMRAVKHRQLQAEGLDLLLVGVRFQRNVLDLTLQWRFARLPNNAKLEMVPVSCNKV

979 61 GTGNTVRIALQLDDGSRLQDTFLCQQTLWELLNHFAKTREFLEQHGEFCLVCIYMRDEIS

979 121 GKDALEKTTLKSLGLTGGSAIVRVVVKKCSLPGQEEAVDTTTQCDEPVVRPGSTEGAVDV

979 181 PLPQTQTFPTVLDHGDEALSLNNCTDKQDSIRESHASTEELCPPSEPKSSPFVPFIGGGQ

979 241 RLGDSVSAEPLELDMPSSELPTTFSSPGGPSKPKKSKNSQELQKEQEQLVEREPLLCHPD

979 301 LLEPIPADLEELPDEFFEVTVDDVRKRLAQLQSERQVPFLLMTVFVSYPVAEHTHTVPLL

979 361 LGSGASEIHMSPQMGLKIALPLVGLEQLVVQMKRLEEAPLLTRSLREAQLKEKLERYPKV

979 421 VLRVHFPDRHILQGFFRPTETGKSSLCFRDSCLYALVDLERRVKQTAVKVLASCIRESRM

979 481 VQWQERKRDLSGSVVSQKTVLLLPDRNVNTYVSAKLFPAAVIHFGSEERRGDLILEWNSL

979 541 GVWEEGFSFFCQGLGANNFLQRLDLRNNQINHHGAGELAVALKRNSSLQELDLRWNNIGL

979 601 LGGRALLNCLQSNKTLKKLELAGNNVPSDILKAVEQAMDHNRDRQTILSESQNRTSVLSK

979 661 EILNLKDEKAKQFLDLMDTIDKQREEIARSGRLQMTEAALALSEQKVHNLGELLSAMKQE

979 721 QANMAERHFAELQQQKQVDHLTQHMEASERSMQDRVQRLESIRIALEEQQRLEHLEEKLR

979 781 LMTEARDEAQNCCLKQKQMVSEAQVKANQLNLHADGLKRRIEELQQELNSKEQEKVTEVN

979 841 KVKVELQEQIGHLQAERTAQEGLREKIAALERQLKVLSSNHREALLDKEGEISMLMEKLR

979 901 MKEADISRMREEEAQRASIL----QNAI------MAFDNYSANVMVDGK**PVNLGLWDTAG**

F6UBD2_CHICK 1 MQAIKCVVVGXXAVGKTCLLISYTTNAFPGEYIPTVFDNYSANVMVDGKPVNLGLWDTAG

979 951 **QEDYDR**LRPLSYPQT---------------------WYPEVR**HHCPNTPIILVGTK**LDLR

F6UBD2_CHICK 61 QEDYDRLRPLSYPQTDVFLICFSLVSPASFENVRAKWYPEVRHHCPNTPIILVGTKLDLR

979 989 DDKDTIERLRDKKLAPITYPQGLAMARGIGSVK**YLECSALTQR**GLK**TVFDEAIRAVLCPP**

F6UBD2_CHICK 121 DDKDTIERLRDKKLAPITYPQGLAMAREIGSVKYLECSALTQRGLKTVFDEAIRAVLCPP

979 1049 **PVK**KPGKKCTVF

F6UBD2_CHICK 181 PVKKPGKKCTVF

**M is the first residue of the new entry 979b**

**985**

985 1 MAAVDRFNLLYREISRSCSVYIEALAIVGAWYTVRKCLTLVFDTYSMLRLHVIPKLVGEI

985 61 DIVKRYGRWAVVTGGTDGIGKAYAEELAKRGVNVILISRSKEKLKAVCRSISETYKVETD

985 121 FIVADFSKGREAYQAIKEGLKDREIGILVNNVGLFYTYPDYFTNLSEDMLWDMININIAS

985 181 ANMMVHIVLPGMVEKKKGAIVNVSSASCCQPTPMLTIYGASKAYLDYFSRALYYEYASKG

E1C6Y2_CHICK 1 ------------------------------------------------------MKPVSS

985 241 IFVQSLTPFVIATKMVSCSSVTSKRSFFFPSAEEYASHAISTLGLSKRTPGYWKHSIEAM

E1C6Y2_CHICK 7 MTMKFANIWFVLVVILLCGKK------HFSTRLGNSSHE------AHVCPGCSRLTLKV-

985 301 CLHKHGSNYLQCCLA-EYIVAFNGYFTAKARSKFISSALKSSGIENWRIVPR**NNPASDYP**

E1C6Y2_CHICK 54 -------EFTSAVVEHEYIVAFNGYFTAKARSKFISSALKSSDIENWRIVPRNNPASDYP

985 361 **SDFEVIQINEKQKDGVLTLEDHPNIK**RVTPQRKVFRSLKYSD------C-----------

E1C6Y2_CHICK 106 SDFEVIQINEKQKDGVLTLEDHPNIKRVTPQRKVFRSLKYSDSDPTLHCNETRWTQKWQS

985 403 ---------------------------------------------------AGVR**VAVFD**

E1C6Y2_CHICK 166 SRPLRRASLSLGSGFWHATGRHSSRRLLRAIPRQVAQTLQADVLWQMGYTGAGVRVAVFD

985 412 **TGLSEK**HPHFKNVKERTNWTNERTLDDGELLCTVGLGHGTFVAGVIASMR**ECQGFAPNAE**

E1C6Y2_CHICK 226 TGLSEKHPHFKNVKERTNWTNERTLDDG-------LGHGTFVAGVIASMRECQGFAPNAE

985 472 **LHIFR**VFTNNQVSYTSWFLDAFNYAILKKIDVLNLSIGGPDFMDHPFVDKVWELTANNVI

E1C6Y2_CHICK 280 LHIFRVFTNNQVSYTSWFLDAFNYAILKKIDVLNLSIGGPDFMDHPFVDKVWELTANNVI

985 532 MVSAIGNDGPLYGTLNNPADQMDVIGVGGIDFEDNIARFSSRGMTTWELPGGYGRVKPDI

E1C6Y2_CHICK 340 MVSAIGNDGPLYGTLNNPADQMDVIGVGGIDFEDNIARFSSRGMTTWELPGGYGRVKPDI

985 592 VTYGSGVRGSGMKGGCRSLSGTSVASPVVAGAVTLLVSTVQKREMVNPASMKQALIASAR

E1C6Y2_CHICK 400 VTYGSGVRGSGMKGGCRSLSGTSVASPVVAGAVTLLVSTVQKREMVNPASMKQALIASAR

985 652 RLPGVNMFEQGHGKLDLLRAYQILNSYKPQASLSPSYIDLTECPYMWPYCSQPIYYGGMP

E1C6Y2_CHICK 460 RLPGVNMFEQGHGKLDLLRAYQILNSYKPQASLSPSYIDLTECPYMWPYCSQPIYYGGMP

985 712 TIVNVTILNGMGVTGRIIDK----------------------------------------

E1C6Y2_CHICK 520 TIVNVTILNGMGVTGRIIDKPDWQPYLPQNGDNIEVAFSYSPVLWPWSGYLAISISVAKK

985 732 -----------------------SKNGAEQTSTVKLPIKVKIIPTPPRSKRVLWDQYHNL

E1C6Y2_CHICK 580 AASWEGIAQGHVMITVSSPAENKSKNGAEQTSTVKLPIKVKIIPTPPRSKRVLWDQYHNL

985 768 RYPPGYFPRDNLRMKNDPLDWNGDHIHTNFRDMYQHLRSMGYFVEVLGSPFTCFDASQYG

E1C6Y2_CHICK 640 RYPPGYFPRDNLRMKNDPLDWNGDHIHTNFRDMYQHLRSMGYFVEVLGSPFTCFDASQYG

985 828 TLLMVDSEEEYFPEEITKLRRDVDNGLSLIVFSDWYNTSVMRKVKFYDENTRQWWMPDTG

E1C6Y2_CHICK 700 TLLMVDSEEEYFPEEITKLRRDVDNGLSLIVFSDWYNTSVMRKVKFYDENTRQWWMPDTG

985 888 GANIPALNDLLSVWNMAFSDGLYEGDFTMASHEMNYASGCSIAKFPEDGIVIAQTFKDQG

E1C6Y2_CHICK 760 GANIPALNDLLSVWNMAFSDGLYEGDFTMASHEMNYASGCSIAKFPEDGIVIAQTFKDQG

985 948 LEVLKQETAVIENVPILGLYQVPSEGGGRIVLYGDSNCLDDSHRQK--------------

E1C6Y2_CHICK 820 LEVLKQETAVIENVPILGLYQVPSEGGGRIVLYGDSNCLDDSHRQKDCFWLLDSLLQYTS

985 ------------------------------------------------------------

E1C6Y2_CHICK 880 YGVMPPSLSHSENRQRPPSGEGCPLPERMEGNHLHRYSKVLEAHLGDPKPRSLPACPHLS

985 995 --------------------------------------------------------GIMP

E1C6Y2_CHICK 940 WAKPQPLNETAPSNLWKHQKLLSIDLDKVALPSFRQNRPQVRPLSPGESGAWDIPGGIMP

985 999 GRYNQDVGQTIPVFAFLGAMVVLAFFVVQINKAKSRPKRRKPRVKRPQLMQQVHPAKTPS

E1C6Y2_CHICK 1000 GRYNQDVGQTIPVFAFLGAMVVLAFFVVQINKAKSRPKRRKPRVKRPQLMQQVHPPKTPS

985 1059 V

E1C6Y2_CHICK 1061 V

**E is the first amino acid of the new entry 985b**
